# Investigation of a Quadruplex-Forming Repeat Sequence Highly Enriched in *Xanthomonas* and *Nostoc* sp.

**DOI:** 10.1371/journal.pone.0144275

**Published:** 2015-12-22

**Authors:** Charlotte Rehm, Lena A. Wurmthaler, Yuanhao Li, Tancred Frickey, Jörg S. Hartig

**Affiliations:** 1 Department of Chemistry and Konstanz Research School Chemical Biology (KoRS-CB), University of Konstanz, Universitätsstr. 10, 78457 Konstanz, Germany; 2 Department of Biology, University of Konstanz, Universitätsstr. 10, 78457 Konstanz, Germany; Belgian Nuclear Research Centre SCK•CEN, BELGIUM

## Abstract

In prokaryotes simple sequence repeats (SSRs) with unit sizes of 1–5 nucleotides (nt) are causative for phase and antigenic variation. Although an increased abundance of heptameric repeats was noticed in bacteria, reports about SSRs of 6–9 nt are rare. In particular G-rich repeat sequences with the propensity to fold into G-quadruplex (G4) structures have received little attention. In silico analysis of prokaryotic genomes show putative G4 forming sequences to be abundant. This report focuses on a surprisingly enriched G-rich repeat of the type GGGNATC in *Xanthomonas and* cyanobacteria such as *Nostoc*. We studied in detail the genomes of *Xanthomonas campestris pv*. *campestris* ATCC 33913 (*Xcc*), *Xanthomonas axonopodis pv*. *citri* str. 306 (*Xac*), and *Nostoc sp*. strain PCC7120 (*Ana*). In all three organisms repeats are spread all over the genome with an over-representation in non-coding regions. Extensive variation of the number of repetitive units was observed with repeat numbers ranging from two up to 26 units. However a clear preference for four units was detected. The strong bias for four units coincides with the requirement of four consecutive G-tracts for G4 formation. Evidence for G4 formation of the consensus repeat sequences was found in biophysical studies utilizing CD spectroscopy. The G-rich repeats are preferably located between aligned open reading frames (ORFs) and are under-represented in coding regions or between divergent ORFs. The G-rich repeats are preferentially located within a distance of 50 bp upstream of an ORF on the anti-sense strand or within 50 bp from the stop codon on the sense strand. Analysis of whole transcriptome sequence data showed that the majority of repeat sequences are transcribed. The genetic loci in the vicinity of repeat regions show increased genomic stability. In conclusion, we introduce and characterize a special class of highly abundant and wide-spread quadruplex-forming repeat sequences in bacteria.

## Introduction

Non-B DNA structures have been identified in eukaryotes as well as prokaryotes [1, 2]. Z-DNA is formed by alternating purine/pyrimidine patterns [3, 4] and A- or H-DNA by oligo-purine or—pyrimidine runs [5, 6]. Other examples of sequences that can give rise to non-canonical DNA structures include palindromes and close inverted repeats [7], simple sequence repeats (SSRs) [8, 9] as well as G-quadruplex (G4) forming sequences [10, 11]. Among these different structural elements mutagenic effects on DNA have been associated especially to SSRs [12]. These perfect (or near-perfect) direct iterations of short DNA tracts in a head-to-tail manner with a motif size of 1–9 nt are also termed ‘tandem repeats’ [9]. In bacteria next to SSRs a number of other small repeat classes have been identified primarily in intergenic regions, e.g. Miniature Inverted-repeat Transposable Elements (MITEs) [13, 14], Repetitive Extragenic Palindromic sequences (REPs) [15] and Clustered Regularly Interspaced Short Palindromic Repeats (CRISPRs) [16, 17]. All three belong to the general class of inverted repeats. In addition to genomic instability there is increasing evidence for non-canonical nucleic acid structures to directly or indirectly influence replication, recombination, transcription and translation on the DNA or RNA level [1, 10, 18–23].

So far research on tandem repeats has primarily been focused on short 1–4 nt repeats of which every possible combination has been found to be vastly over-represented in the human genome [8]. In particular trinucleotide expansions in open reading frames (ORFs), introns or untranslated regions (UTRs) have been identified to give rise to human neurodegenerative disorders such as Huntington disease [24], spinobulbar muscular atrophy [25] and Fragile X syndrome [26]. Although microsatellites have been found in prokaryotes as well, they are present at lower numbers [27]. Especially longer repeat sequences are less abundant than in eukaryotes [8]. The distribution of SSRs across bacterial species has been shown to vary greatly even among close relatives [28–30]. In general, SSRs with smaller unit sizes of 1–4 nt are found more abundantly in smaller genomes, especially those of host-adapted pathogens and of low G+C content [31–33]. In contrast, longer repeat runs were more frequently found in non-pathogens and bacteria with large genomes (> 4Mb) and high G+C content (> 60%) [33]. Major differences were detected in the distribution of SSRs in coding and non-coding regions. In *Escherichia coli* (*E*. *coli*) most repeat sequences were found to concentrate in intergenic regions up to 200 nt upstream of the start codon, the region containing proximal regulators of gene expression. Investigation of tandem repeats in *E*. *coli* by Gur-Arie et al. also showed them to be under-represented in ORFs when exceeding a unit size of 3 nt [34]. SSRs play a role in bacterial evolution, where they allow for local sequence variation and thereby enable accelerated adaption to changing environmental conditions [35, 36]. By inducing local genetic instability SSRs have been shown to act as cis-regulatory motifs enabling the modulation of gene expression in a reversible manner, especially in phase and antigenic variation [22, 23, 37]. Both processes allow the switching of phenotypes in a bacterial population and thereby are thought to increase their fitness.

Research conducted on SSRs with longer repeat units of 5–9 nt is rare. In 1999 van Belkum et al. presented a study on the occurrence of pentameric tandem repeats in bacterial genomes [38]. Although heptameric repeats were found to be over-represented among SSRs in many bacterial genomes in 2007 [33] no detailed literature focusing on heptameric repeats is available to date. Van Belkum et al. report one example of a heptanucleotide 5’-GTGATTA-3’ in *Helicobacter pylori* [38]. The presence of three different tandemly repetitive heptanucleotides has also been reported for the cyanobacterium *Calothrix* sp. strain PCC7601 [39]. However, no further characterization of these repeats has been carried out. Recently, Mrázek and Huang presented an extensive assessment of local sequence patterns with the potential to form non-canonical DNA conformations from 1424 bacterial chromosomes [20]. A different representation of short versus long SSRs was reported with longer tandem repeats showing normal or slight over-representation. When analyzing Mrázek and Huang’s data for γ-proteobacteria and cyanobacteria only, we noted a strong over-representation of heptameric SSRs in intergenic regions in *Xanthomonas* and *Nostoc* species, while other long SSRs in the range of 4–11 nt were normally represented. Furthermore, a slight over-representation of intergenic G4 forming sequences is present in xanthomonads, strong over-representation is evident for *Nostoc* species. G4s are four-stranded helical complexes that are assembled from multiple stacked guanine tetrads. These specialized secondary structures can be formed either by DNA or RNA consisting of consecutive runs of guanines. G-rich repeats are of special interest as in addition to being SSRs they also represent potential G4 forming sequences. G4 structures have been shown to be able to carry out a variety of cellular functions in eukaryotes, e.g. in replication and recombination [10] or as transcriptional regulators [40–42]. However, much less is known about their function in the eubacterial kingdom of life. In an earlier study Chowdhury and co-workers identified potential G4 forming sequences in 18 bacterial strains and report them to be over-represented in regulatory regions [43]. We have previously shown that G4s can be used as translational repressors in an artificial system in bacteria [44]. Recently, we have studied the multifaceted effects of G4s as potent transcriptional and translational regulators in *E*. *coli*. The influence of G4 sequences proved to depend strongly on strand orientation and the exact location within the promoter region, 5´-UTR or 3´-UTR [21]. In this report we focus on G-rich heptameric repeats of the type GGGAATC in the plant pathogens *Xanthomonas campestris pv*. *campestris* ATCC 33913 (*Xcc*) [45] and *Xanthomonas axonopodis pv*. *citri* str. 306 (*Xac*) [46]. In addition we studied similar GGGGA(T/C)T repeats in the cyanobacterium *Nostoc sp*. strain PCC7120 (*Ana*) [47].

## Materials and Methods

### Identification and characterization of repeat patterns

Potential G4 forming sequences were initially obtained from ProQuad Database (http://quadbase.igib.res.in/) [48]. Using the following query parameters for *Xcc*: pattern G (or C for minus strand), stem size G3 (or C3) and loop size L1-5, genomic location: all. For further studies the chromosomal sequences of *Xcc* (NC_003902), *Xac* (AE008923), plasmids pXAC33 (NC_003921) and pXAC64 (NC_003922) and Ana (NC_003272) were downloaded from the NCBI website. *Xcc* and *Xac* genomes were manually searched for repeats comprising at least two units and containing at least once the heptamer “GGGAATC” using the software Clone Manager 9 (Scientific & Educational Software). For *Ana* stem size G3-5 (or C3-5) and loop size L1-7 was used in the ProQuad search. From this set all patterns of the type G_4-_L_1-4_ containing at least twice the units GGGGA(C/T)T were selected. Frequency plots showing the consensus nucleotide sequence of a heptameric unit were created with WebLogo (http://weblogo.berkeley.edu/logo.cgi) [49]. Distances from the respective start or end point of the repeat to the start or stop codon to the next neighboring ORF were calculated. Subsequently repeats were grouped into three categories of increasing distance between the repeat motif and the start or stop codon of the neighboring ORF of 0–50 bp, 50–100 bp and > 100 bp. Functions of annotated genes and their positions on the genome were collected from KEGG (http://www.kegg.jp/), NCBI as well as Cyanobase (http://genome.microbedb.jp/cyanobase) [50]. Repeat associated genes were sorted into functional categories using the KEGG pathway mapper (http://www.genome.jp/kegg/mapper.html) [51].

### Circular Dichroism (CD) Measurements

Oligonucleotides (Table A in S3 File) for CD measurements and melting assays were synthesized by Sigma Aldrich (Steinheim, Germany) at the 1 μmol scale with HPLC purification. CD spectra were recorded on a JASCO-J815 spectropolarimeter equipped with a MPTC-490S/15 multicell temperature unit using quartz cells with 1 cm optical path. Oligonucleotides were prepared in a reaction volume of 600 μL as a 5 μM solution in 10 mM Tris-HCl or 10 mM sodium acetate for C-rich oligonucleotides and adjusted to the indicated pH 4.5–7.5 with HCl. If noted, the solution was supplemented with either KCl, NaCl or LiCl to the indicated concentration. Oligonucleotides were denatured by heating to 98°C for 5 min, followed by slow cooling to 20°C over night. Scans were performed at 20°C over a wavelength range of 220–320 nm (5 accumulations) with a scanning speed of 500 nm/min, 0.5 s response time, 0.5 nm data pitch and 1 nm bandwidth. The buffer spectrum was subtracted and all spectra zero-corrected at 320 nm. For thermal denaturation oligonucleotides were prepared as previously described. Due to the temperature dependent pH change of tris buffer, melting experiments of C-rich oligonucleotides were carried out in sodium acetate buffer only. Samples were heated from 20°C to 100°C at a rate of 0.5°C/min. The CD signal was recorded every 0.5°C at the indicated wavelength. The temperature of the half-maximal decay of ellipticity T_1/2_ was obtained from the normalized ellipticity decrease using the Boltzmann sigmoidal fit.

### Analysis of sequence homology between *Xcc* and *Xac* in repeat containing regions

Nucleotide BLAST [52] (http://blast.ncbi.nlm.nih.gov/Blast.cgi) was used to compare sequence similarity between *Xcc* and *Xac* applying the following parameters: algorithm: blastn (somewhat similar sequences), database: NCBI genomes (chromosome), organism: *Xanthomonas axonopodis pv*. *citri str*. 306 (taxid: 190486). The entire repeat containing intergenic region and the next up- and downstream neighboring ORFs or the entire ORF containing an intragenic repeat of *Xcc* were used as query sequence. Presence of the repeat was assessed. Sites where the alignments showed less homology or gaps were then checked directly in Clone Manager for repeat presence and compared for general changes in the intergenic regions and neighboring genes. 260 intergenic regions that did not contain GGGAATC repeats including the next neighboring ORFs were randomly chosen from the *Xcc* genome and subjected to the same blast analysis. Control sets were randomly assembled from this pool of controls to contain 117 queries each. From the same pool sequences for control 4 were chosen to show the same distribution along the *Xcc* genome and sequences for control 5 were chosen to show the same orientation of neighboring ORFs as the intergenic repeat containing sequences. One-sample t-tests were carried out using R (version 3.0.2) for each category. Distribution of the orientation of the neighboring genes relative to the repeats was analyzed for all controls.

### Analysis of whole transcriptome sequencing data of *Xac*

Paired-end reads of *Xac* (referred to as XccA306 by Jalan et al. [53]) of NB sample 2 were downloaded from Gene Expression Omnibus database of NCBI (accession number GSE41519). Read quality was first checked with FastQC (http://www.bioinformatics.babraham.ac.uk/projects/fastqc/) (version 0.11.2) and then trimmed with Trimmomatic [54] (version 0.32). Trimmed reads were then mapped to the *Xac* genome using bowtie2 [55] (version 2.2.3). Uniquely mapped reads were assembled by Trinity [56, 57] (version r20140717). In total, 4266 transcripts were assembled, and their expression levels were calculated by aligning reads to each assembled transcript and normalizing them by Fragments Per Kilobase of exon per Million fragments mapped (FPKM). Assembled transcripts were then mapped to the *Xac* genome using blat [58] to obtain their respective coordinates on the genome. Number and orientation of repeat-containing transcripts was determined. Repeats were further classified as potential G4 forming repeats (at least 4 G-tracts without mutations) or short repeats unable to form G4s (controls). Assembled transcripts were then sorted into the three categories according to the location of the repeat within the assembled transcript: start, middle or end. Reads mapping to repeats located in coding regions were excluded from the final analysis.

## Results

### GGGAATC **repeat sequences in xanthomonads**

**Fig 1:**
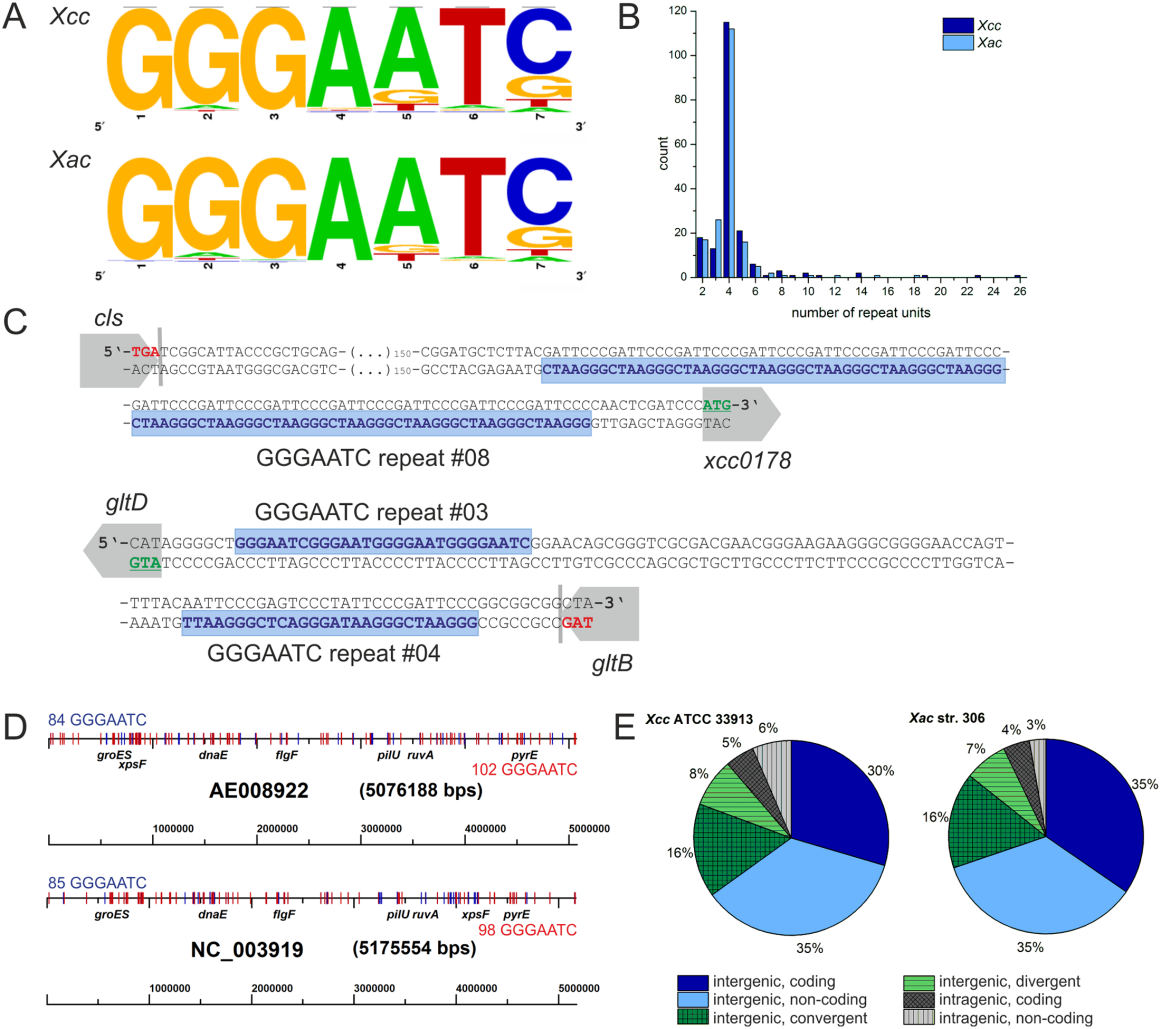
Overview of repeats in *Xanthomonas* species *Xcc* and *Xac*. (A) Frequency plot [49] shows the consensus nucleotide sequence of a heptameric repeat unit in *Xcc* (top) and *Xac* (bottom). (B) Histogram shows the count of repeat iterations per repeat sequence in *Xcc* (dark blue) and *Xac* (light blue). (C) Examples of GGGAATC patterns in *Xcc*. Repeat #08 located upstream of the hypothetical gene xcc0178 is the longest, perfect repeat present (top). Repeat #03 and #04 represent an inverted repeat with two short repeat sequences located in convergent orientation on the plus and on the minus strand of the genome (bottom). (D) Distribution of GGGAATC on the *Xcc* (AE008922, top) and *Xac* (NC_003919, bottom) genomes. Repeats located on the plus strand are marked in blue (84 *Xcc*, 85 *Xac*), repeats on the minus strand in red (102 *Xcc*, 98 *Xac*). Locations of repeat associated genes *groES*, *dnaE*, *flgF*, *pilU*, *ruvA*, *pyrE* and *xpsF* have been marked for orientation. (E) Orientation of neighboring genes relative to repeat sequences in *Xcc* (left) and *Xac* (right). Intergenic repeats can be located on the same strand that will serve as the coding strand of the aligned ORFs (dark-blue) or on the non-coding strand (light blue), between convergent (dark green) or divergent (light green) ORFs. Intragenic repeats can be located on the coding strand (dark gray) or non-coding strand (light gray).

The findings of Mrázek and Huang motivated us to investigate potential G4 forming sequences in the plant pathogen *Xanthomonas* in more detail. First we used the ProQuad Pattern Search [48] to gain an overview of potential G4 folding sequences in the *Xcc* genome (total of 270 potential G4s with G_3_-L_1-5_, Table A and B in S1 File). We hereby noticed an intriguing over-representation of GGGAATC repeat patterns among the putative quadruplex patterns, which led us to screen the genomes of *Xcc* and the related species *Xac* for GGGAATC-containing tandem repeats. The following parameters were used to define these G-rich SSRs: the total length must be ≥14 bp (at least 2 units) and contain at least once the GGGAATC heptamer. Repeats can be either perfect repeats (GGGAATC)_n_ or heterogeneous (GGGANTN)_n_. In total we identified 186 G-rich repeat patterns in *Xcc* and 183 in *Xac* (Table A and B in S2 File). The frequency plot in Fig 1A shows the consensus motif of a heptamer unit, in both organisms position 1–4 and 6 show high sequence conservation. Although extensive length variation was noted with repeats ranging from 2 to 26 units in *Xcc* and 2 to 18 units in *Xac*, the majority of the sequence motifs comprise four repeat units, as shown in the histogram in Fig 1B. 56% of all repeats in *Xcc* and 42% in *Xac* are made up of ≥4 units and have no point mutations in the G-tract, which would prevent G4 formation. An example for the longest perfect repeat with 14 GGGAATC units from *Xcc* is given in Fig 1C (top). Remarkably, in 70 cases in *Xcc* and 75 cases in *Xac* we found two repeat sites with convergent orientation in close proximity to each other, always located once on the plus and once on the minus strand of the genome. An example for such an inverted repeat is shown in Fig 1C (bottom). This rearrangement is of particular interest as inverted repeats have the potential to give rise to stem-loops or cruciform structures.

We found that GGGAATC repeat sequences are dispersed all over the genome in both species and do not show preference for a defined region on the chromosome, such as the origin or terminus of replication. Repeats are about equally distributed on the plus and minus strand of the chromosome and show no preference in regard to presence in the leading or lagging strand during replication (Fig 1D). In contrast to *Xcc*, *Xac* carries two plasmids, pXAC33 and pXAC64. No repeat sequences were identified on these plasmids. The repeats were most often found in intergenic regions (89% *Xcc*, 93% *Xac*) (Fig 1E) and are almost exclusively located at a shorter distance to the next 5’ neighboring ORF (average distance 28 nt) than to the next downstream ORF (average distance 160 nt). Regarding the orientation of the neighboring ORFs to the intergenic repeats, we found that the majority of ORFs were oriented in the same direction, with the repeats localized in the intergenic region. In *Xcc* 30% of the G-rich patterns are present on the same strand as the aligned ORFs, and in 35% of cases are present on the opposite strand than the aligned ORFs. In *Xac* there are 35% of all repeats assigned to each of these categories. In both xanthomonads 16% of repeats were located between convergent ORFs, while only 8% in *Xcc* and 7% in *Xac* were located between divergent ORFs. Intragenic repeats are similarly rare, accounting to 11% in *Xcc* and 7% in *Xac* with at least partly overlap with the ORF (Fig 1E). Interestingly, although a high degree of sequence homology exists between *Xcc* and *Xac* [46, 59], repeats of similarly prominent length are not found in association with the same genes in the two species (Table B and C in S3 File).

### GGGGA(C/T)T **repeat sequences in *Nostoc***

**Fig 2:**
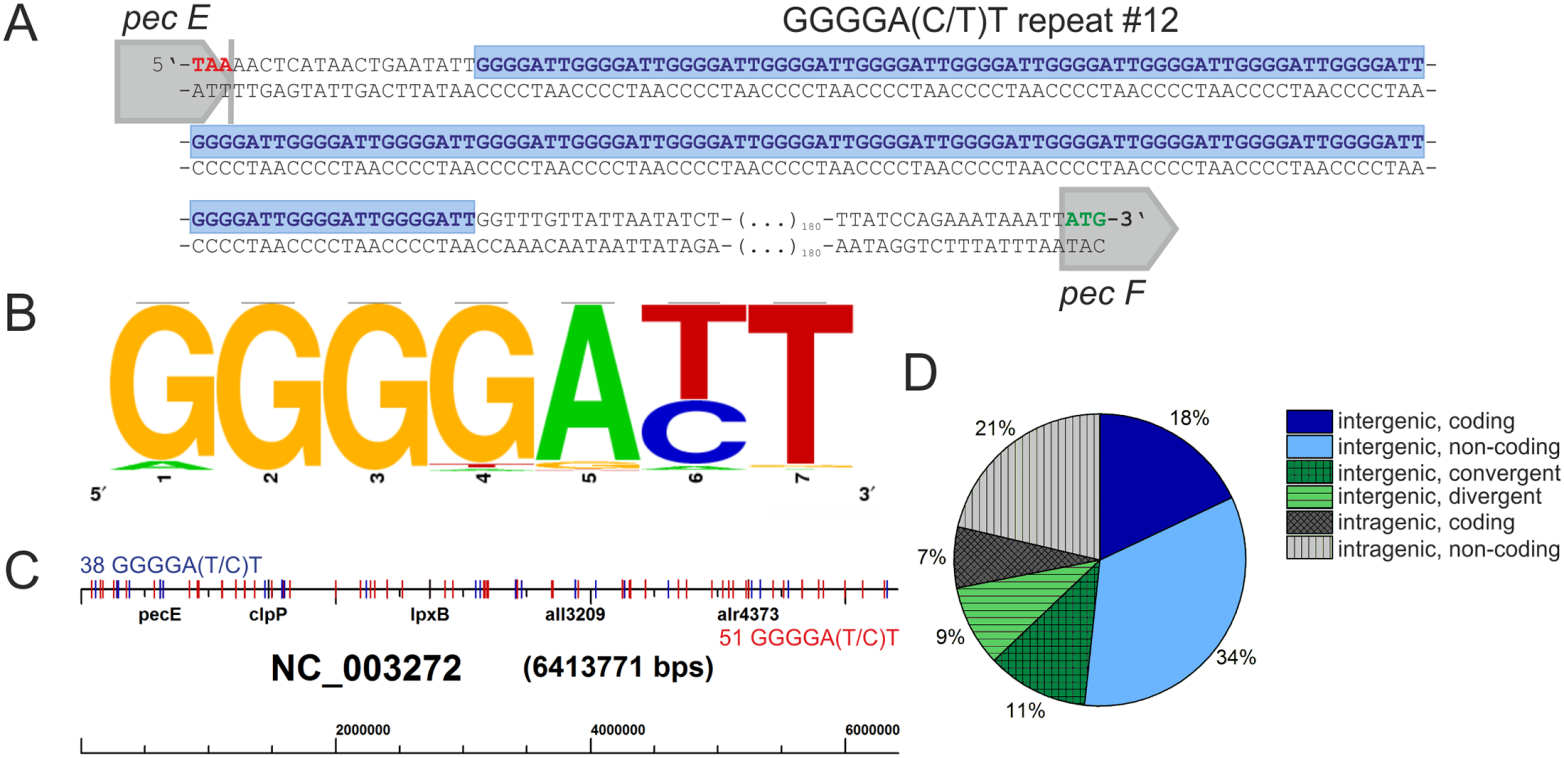
Overview of repeats in *Ana*. (A) Examples of a perfect 26mer GGGGATT repeat patterns in *Ana*. Repeat #12 is located in an intergenic region in the pec operon between *pecE* and *pecF*. (B) Frequency plot [49] shows the consensus nucleotide sequence of heptameric repeat units in Ana. (C) Distribution of GGGGA(T/C)T repeats on the *Ana* chromosome (NC_003272). Repeats located on the plus strand are marked in blue [38], repeats on the minus strand in red [51]. Locations of repeat associated genes *pecE*, *clpP*, *lpxB*, *all3209* and *alr4373* have been marked for orientation. (D) Orientation of neighboring genes relative to repeat sequences. Intergenic repeats can be located on the same strand that will serve as the coding strand of the aligned ORFs (dark-blue) or on the non-coding strand (light blue), between convergent (dark green) or divergent (light green) ORFs. Intragenic repeats can be located on the coding strand (dark gray) or non-coding strand (light gray).

Generally, a very high repeat-coverage was found for cyanobacterial genomes [33]. Mrázek and Huang not only reported a strong over-representation of long SSRs, but in a later publication particularly of potential G4 forming sequences [20]. Even earlier, Swanson et al. noticed a long stretch of G-rich heptamer repeats in the *pec* (phycoerythrocyanin) locus of *Nostoc sp*. strain PCC7120 (*Ana*) (Fig 2A) [60], however to the best of our knowledge no further studies concerning this DNA pattern have been carried out to date. We therefore chose *Ana* for a more detailed examination. Despite its low G+C content of 41.3%, ProQuad pattern search (G_3-5-_L_1-5_) yielded 471 hits (Table C and D in S2 File). Because of the high abundance of G-rich patterns we chose to focus our analysis on only repeat sequences containing at least twice the runs 5’-GGGGATT-3’ or 5’-GGGGACT-3’, similar to the patterns observed in xanthomonads. The analysis yielded 89 SSRs in total (Table C in S2 File). The frequency plot in Fig 2B shows the consensus nucleotide sequence GGGGA(T/C)T. The identified repeat patterns again varied strongly in length from 39 to 179 nt. The longest perfect GGGGATT pattern is a 26mer located within the *pec* operon (Fig 2A). Repeat patterns were again distributed all over the *Ana* chromosome, not restricted to specific genomic locations (Fig 2C) and almost equally distributed between the plus (43%) and minus strand (57%). Although the majority of repeats are located intergenically (68%), a significantly higher fraction of repeats is located intragenically than was the case for the xanthomonads. This is especially remarkable as the average codon usage in *Ana* shows a lower G+C content than codons used in xanthomonads (coding GC-content 42.34% in *Ana*, 65.58% *Xcc*, 65.06% *Xac*, http://www.kazusa.or.jp/codon). Regarding the orientation of the neighboring ORFs to the repeat only 18% of all repeats were oriented in the same direction with the neighboring ORFs and 34% are present on the opposite strand between aligned ORFs (Fig 2D). 11% of repeats were located between convergent ORFs and 9% between divergent ORFs. In contrast to *Xanthomonas* we found only a few paired repeats that could form inverted repeats. In addition to the chromosome *Ana* carries six plasmids, but repeat patterns were not found on the plasmids.

### Oligonucleotides derived from repeat sequences form G4s in vitro

**Fig 3:**
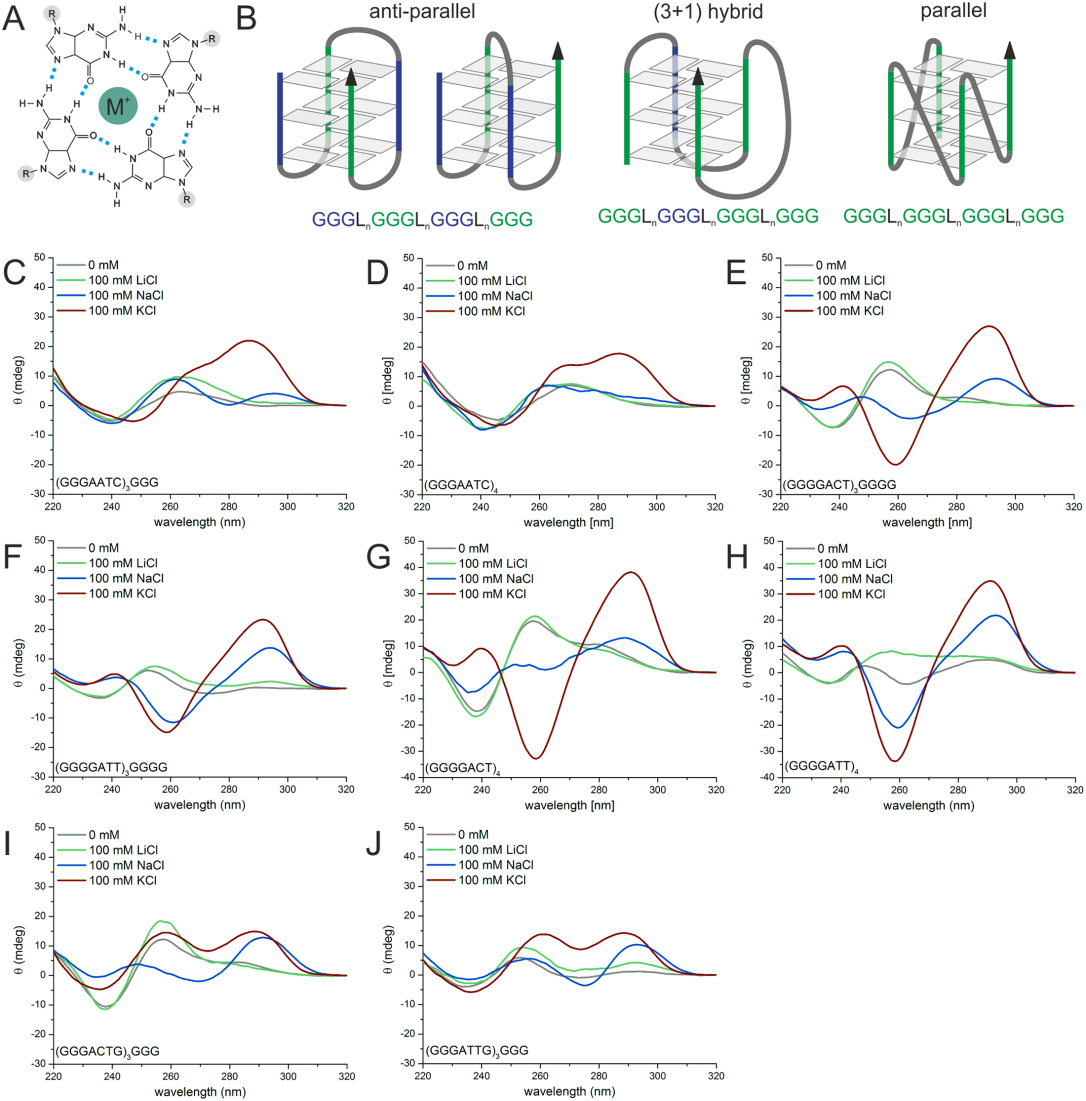
Circular dichroism analysis of G-rich repeat derived oligonucleotides. (A): Top view of a guanine tetrad formed by Hoogsteen base-pairing. Hydrogen bonds are depicted by light blue dashed lines. Monovalent cations M^+^ (green) in the central cavity or between tetrads stabilize the structure. Sugar-phosphate backbone of the nucleic acid is depicted by R (highlighted in gray). (B): Schemes of different G4 topologies with three tetrads, from left to right: anti-parallel chair and anti-parallel basket structure, (3+1) hybrid structure and all-parallel propeller structure. Guanines forming a tetrad are represented by gray rectangles. General nucleic acids sequence is shown underneath, with Ln representing the nucleotides in the loop. Different strand orientations are indicated by blue (top to bottom) and green (bottom to top), the arrow indicates the 3’ end. (C-J): CD spectra recorded from 220 to 320 nm of 5 μM oligonucleotide in 10 mM Tris-HCl (pH 7.5) in the presence of 100 mM LiCl (green), 100 mM NaCl (blue), 100 mM KCl (red) or tris buffer only (gray), (GGGAATC)_3_GGG (C), (GGGAATC)_4_ (D), (GGGGACT)_3_GGGG (E), (GGGGAAT)_3_GGGG (F), (GGGACTG)_3_GGG (G), (GGGATTG)_3_GGG (H), (GGGGACT)_4_ (I) and (GGGGATT)_4_ (J).

The majority of the sequence motifs comprise four (or more) repeat units. These consecutive runs of guanosines can give rise to G4s on the level of DNA as well as RNA. Hoogsteen base-pairing between the guanines arranges them in a square tetrameric formation, also called a tetrad. The quadruplex is then made up by several such tetrads stacking upon each other; stabilization of the compact structure is achieved by coordination of metal cations in the central cavity (Fig 3A) [61, 62]. We employed circular dichroism (CD) spectroscopy to study putative G4-formation of the repeat-derived DNA oligonucleotides *in vitro*. Stabilization of G4s by monovalent cations is dependent on the nature of the cation, in general the order of the degree of stabilization is K^+^ > Na^+^ > Li^+^ [61]. We analyzed both the minimal motif needed to form a G4 consisting only of the four G-tracts and three loop regions, e.g. 5’-(GGGAATC)_3_GGG-3’, as well as the respective extended repeat motif 5’-(GGGAATC)_4_−3’. In case of the *Ana* sequences different G4 conformations are possible with the fourth guanine either being part of the loop sequence, e.g. 5’-(GGGACTG)_3_GGG-3’, or being located in the G-tract 5’-(GGGGACT)_3_GGGG-3’. Different G4 structures can be distinguished according to their signature in CD, a typical spectrum of an anti-parallel G4 shows a minimum at 260 nm and a maximum at 290 nm, while a G4 with parallel strand orientation shows a minimum at 240 nm and a maximum at 260 nm [63]. Different possible G4 topologies are shown in Fig 3B. For the G-rich motif from *Xcc* 5’-(GGGAATC)_3_GGG-3’ CD spectra in presence of K^+^ showed a minimum in ellipticity at 240–250 nm, a shoulder at 270 nm and a maximum at 290 nm indicative for a (3+1) hybrid structure (Fig 3B **middle**, Fig 3C). The spectral change for the respective repeat motif is less pronounced (Fig 3D). As a control no structural changes could be observed in CD upon introduction of G to T mutations at the second position in the G-tract for the *Xcc* derived oligonucleotides (Fig A in S3 File). Possible quadruplex forming oligonucleotides from *Ana* showed clear formation of an antiparallel structure in the presence of KCl for 5’-(GGGGACT)_3_GGGG-3’ (Fig 3E), 5’-(GGGGATT)_3_GGGG-3’ (Fig 3F) and the repeat motifs 5’-(GGGGACT)_4_−3’ (Fig 3G) and 5’-(GGGGATT)_4_−3’ (Fig 3H). Peaks at 290 nm are also present in the spectra of 5’-(GGGACTG)_3_GGG-3’ (Fig 3I) and 5’-(GGGATTG)_3_GGG-3’ (Fig 3J) in solution with KCl. For these oligo types four guanines are present in the second and third G-tract which enables formation of a variety of G4 structures with three guanines in the G-tract. Spectra of these different structures formed may then overlap in CD. In all cases NaCl did not result to equally pronounced quadruplex formation as KCl and spectra in the presence of LiCl were similar to the unfolded state.

In order to assess thermodynamic stabilities of the structures formed in the presence of KCl and NaCl we performed thermal denaturation experiments. Melting temperatures T_1/2_ are listed in Table D in S3 File. Melting profiles are shown in Fig B in S3 File. We determined moderate melting temperatures T_1/2_ of 50.4°C for the *Xcc* quadruplex 5’-(GGGAATC)_3_GGG-3’ in the presence of 100 mM KCl. All sequences from *Ana* showed to be more stable than the *Xcc* quadruplex with T_1/2_ higher than 74°C; in fact species with G-tracts comprising four guanines 5’-(GGGGACT)_3_GGGG-3’, 5’-(GGGGATT)_3_GGGG-3’ and 5’-(GGGGACT)_4_−3’ could not be fully denatured in presence of KCl with T_1/2_ >95°C. In all cases structures folded in the presence of 100 mM NaCl were less stable than their K^+^ stabilized counterparts.

**Fig 4:**
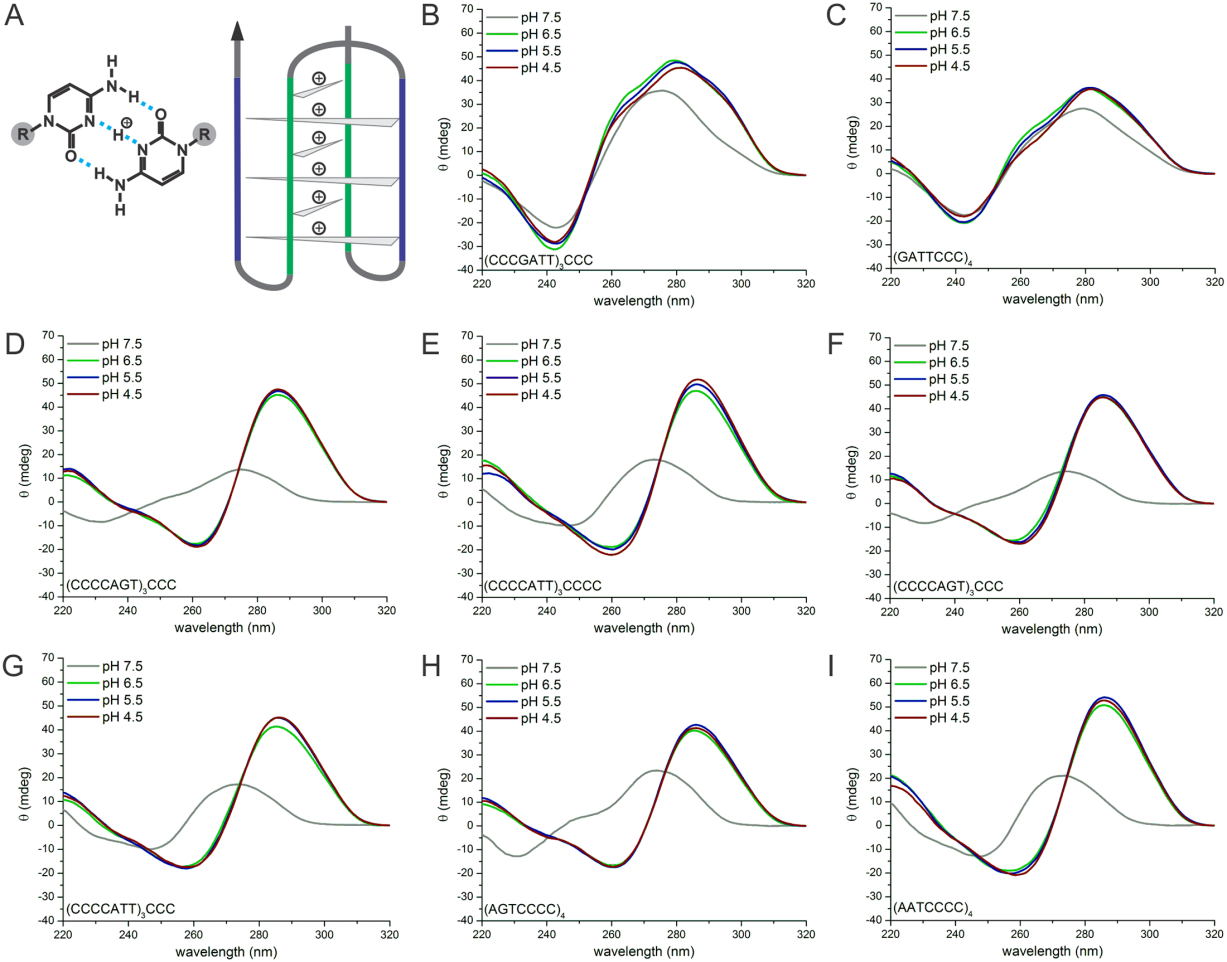
Circular dichroism analysis of C-rich complementary repeat oligonucleotides. (A) Left: Hemiprotonated cytosine-cytosine+ base pair. Hydrogen bonds are depicted by light blue dashed lines, sugar-phosphate backbone of the nucleic acid is depicted by R (highlighted in gray). Right: Scheme of an i-motif formed by a duplex between parallel oriented strands intercalated with anti-parallel duplex. Gray triangles represent cytosine-cytosine+ base pair. Different strand orientations are indicated by blue (top to bottom) and green (bottom to top), the arrow indicates the 3’ end. (B-I) CD spectra recorded from 220 to 320 nm of 5 μM oligonucleotide in 10 mM Tris-HCl pH 7.5 (gray). pH 6.5 (green), pH 5.5 (blue) pH 4.5 (red) for (CCCGATT)_3_CCC (B), (GATTCCC)_4_ (C), (CCCCAGT)_3_CCCC (D), (CCCCAAT)_3_CCCC (E), (CCCCAGT)_3_CCC (F), (CCCCAAT)_3_CCC (G), (AGTCCCC)_4_ (H) and (AATCCCC)_4_ (I).

Since the presence of a G-rich genomic repeat pattern is accompanied by the presence of a C-rich pattern on the complementary strand, we investigated the formation of a four-stranded structure of the C-rich motif. The so-called i-motif structure is formed from C-rich oligonucleotides at mild acidic conditions, which enables the formation of hemiprotonated cytosine-cytosine^+^ base pairs (Fig 4A) [64]. Formation of the i-motif is favored at lower pH, although some sequences are able to stably fold i-motif structures even at neutral pH [65]. CD spectra show a characteristic minimum at about 260 nm and a maximum at around 290 nm [66]. We determined the folding behavior of the complementary C-rich repeat strands while decreasing pH from pH 7.5 to 4.5. CD spectra of the C-rich oligonucleotides derived from *Xcc* already showed a minimum at 240 nm and a maximum at about 270 nm suggesting a folded structure of unknown nature at neutral pH. As the pH of the buffer is decreased the spectrum shifts showing a minimum at 240 nm, shoulder at 260–270 nm and maximum at 280 nm at pH 4.5 suggesting overlapping spectra of different conformations, possibly including an i-motif at 290 nm (Fig 4B and 4C). I-motif signatures were readily detectable in all C-rich oligonucleotides derived from *Ana* (Fig 4D–4I). Remarkably all observed structures persisted even at the elevated pH of 6.5.

We also assessed the thermodynamic stability of the structures formed under acidic conditions (**Table E, Fig C** CD spectra and **Fig D** melting profiles in S3 File). At pH 4.5 all structures are fairly stable with T_1/2_ ranging between 60–72°C. I-motifs have been reported to be destabilized by increased ion concentrations [67, 68], however we found that addition of 100 mM NaCl or KCl did not disturb i-motif formation at pH 4.5. Raising pH to 6.5 lead to a destabilization of the formed structures with T_1/2_ dropping by 15–29°C in comparison to the T_1/2_ determined at pH 4.5, except for (AGTCCCC)_4_, which showed a weaker decrease of only 4°C.

In summary, characteristic changes in ellipticity and enhanced thermodynamic stability were indeed observed under conditions favoring either G4 or i-motif formation. K^+^ has been reported to be the major cation in the bacterial cell, cytosolic concentrations of about 200 mM were determined for *E*. *coli* [69]. A concentration of 100 mM K^+^ therefore represents a concentration likely to be achieved in a cellular environment to stabilize potential G4s.

### Repeats in intergenic regions

**Fig 5:**
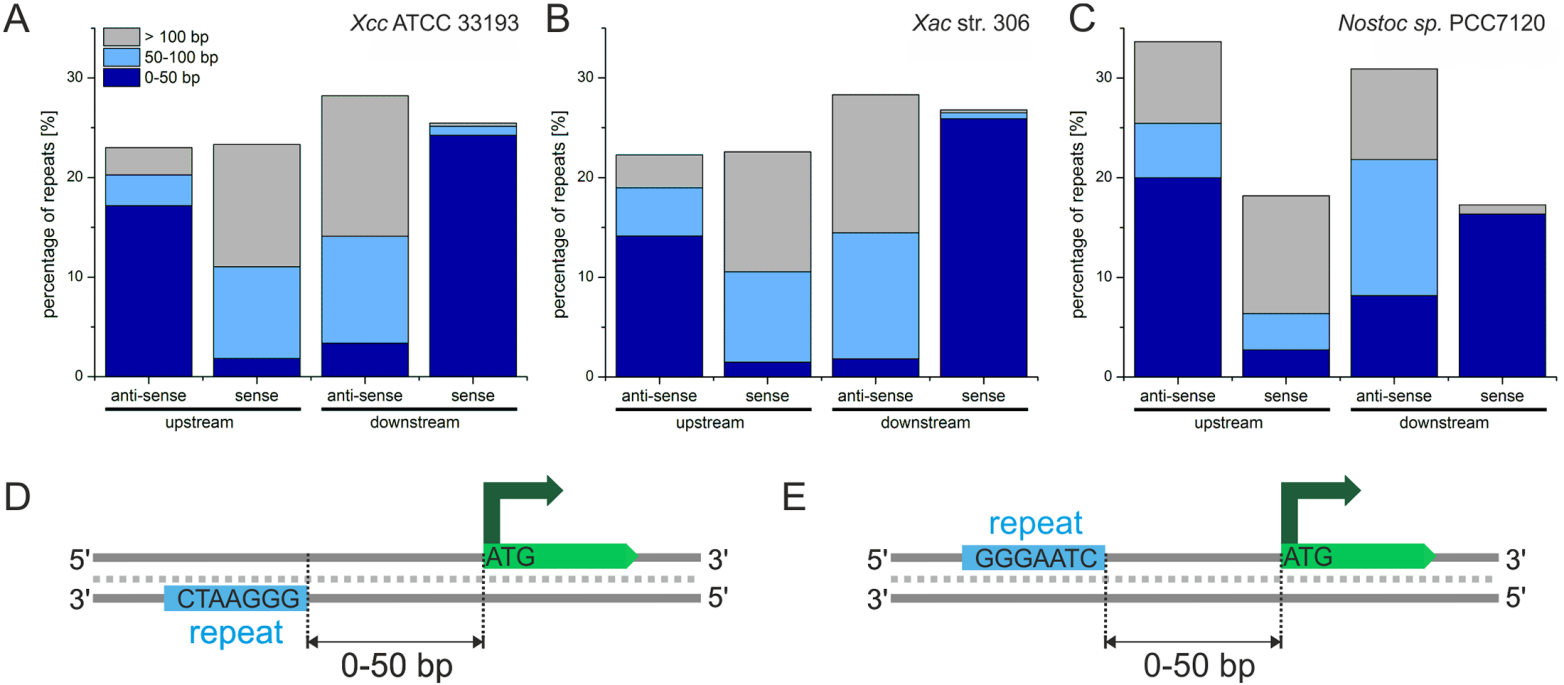
Distances of repeats to neighboring ORFs. (A-C) Analysis of the distance of repeat sequences relative to adjacent ORFs for *Xcc* (A), *Xac* (B) and *Ana* (C). Repeats can be either located upstream or downstream of the next neighboring ORF. Repeats were grouped into three categories according to increasing distance from the respective: distance of > 100 bp (gray), 50–100 bp (light blue) and repeats overlapping with ORFs or located in a distance of up to 50 bp from the respective start or stop codon are grouped together (dark blue). (D) Schematic of a repeat being located in close proximity, upstream of the neighboring ORF on the non-coding strand. (E) Schematic of a repeat being located in close proximity, downstream of the neighboring ORF on the non-coding strand.

During mapping of the repeat sequences we noticed that intergenic repeats are almost exclusively located at a shorter distance to the next 5’ neighboring ORF than to the next downstream ORF irrespective of the ORFs orientation on the genome. We therefore decided to analyze the distance distribution of intergenic repeats in relation to the next neighboring ORF in more detail. We distinguished between a repeat’s position upstream on the coding or non-coding strand of an ORF as well as downstream on the coding or non-coding strand. Repeats were grouped according to increasing distance from the ORF. In all three species intergenic repeats patterns showed a similar distribution (Fig 5A–5C): Upstream of the ORF the greatest fraction is localized within 0–50 bp from the ORF on the non-coding strand (Fig 5D). If the pattern is located on the coding strand the distance to the start codon increases. Downstream of the ORF the situation is reversed: most repeats are located within a distance of 0–50 bp from the stop codon on the coding strand. This includes all repeats overlapping with the stop codon (Fig 5E). When localized on the non-coding strand, the distance to the end of the ORF again increases. When considering only repeats able to form G4s for *Xcc*, we found the same distribution as when also taking into account shorter and mutated repeats (Fig E in S3 File). A preference for the non-coding strand can be observed for *Ana*.

G4s have been shown to be potent modulators of gene expression in eukaryotes and bacteria [10, 21, 40–42, 44, 70] when they are located in close proximity to an ORF, e.g. in the promoter region or UTR. To gain further insight into a potential biological role of the repeat patterns we classified the neighboring genes according to functional classes using the KEGG database [51]. Many of the genes associated to the repeats sequences are hypothetical genes with no further functional description (55% in *Xcc* and *Xac*, 69% in *Ana*). The remaining genes belong mainly to general metabolism pathways. All three organisms show a similar distribution across the gene functional classes (overview and detailed lists in S4 File). Repeats are not exclusively associated to known cell surface structures or genes involved in adaption processes, making a possible function similar to SSRs in phase variation unlikely. In addition we generally did not find them associated with genetically mobile elements such as insertion sequences or transposable elements. However, in order to characterize whether the motif results in increased genetic instability we analyzed the genetic variability in repeat-containing regions.

### Analysis of sequence homology in repeat-containing regions in xanthomonads

**Fig 6:**
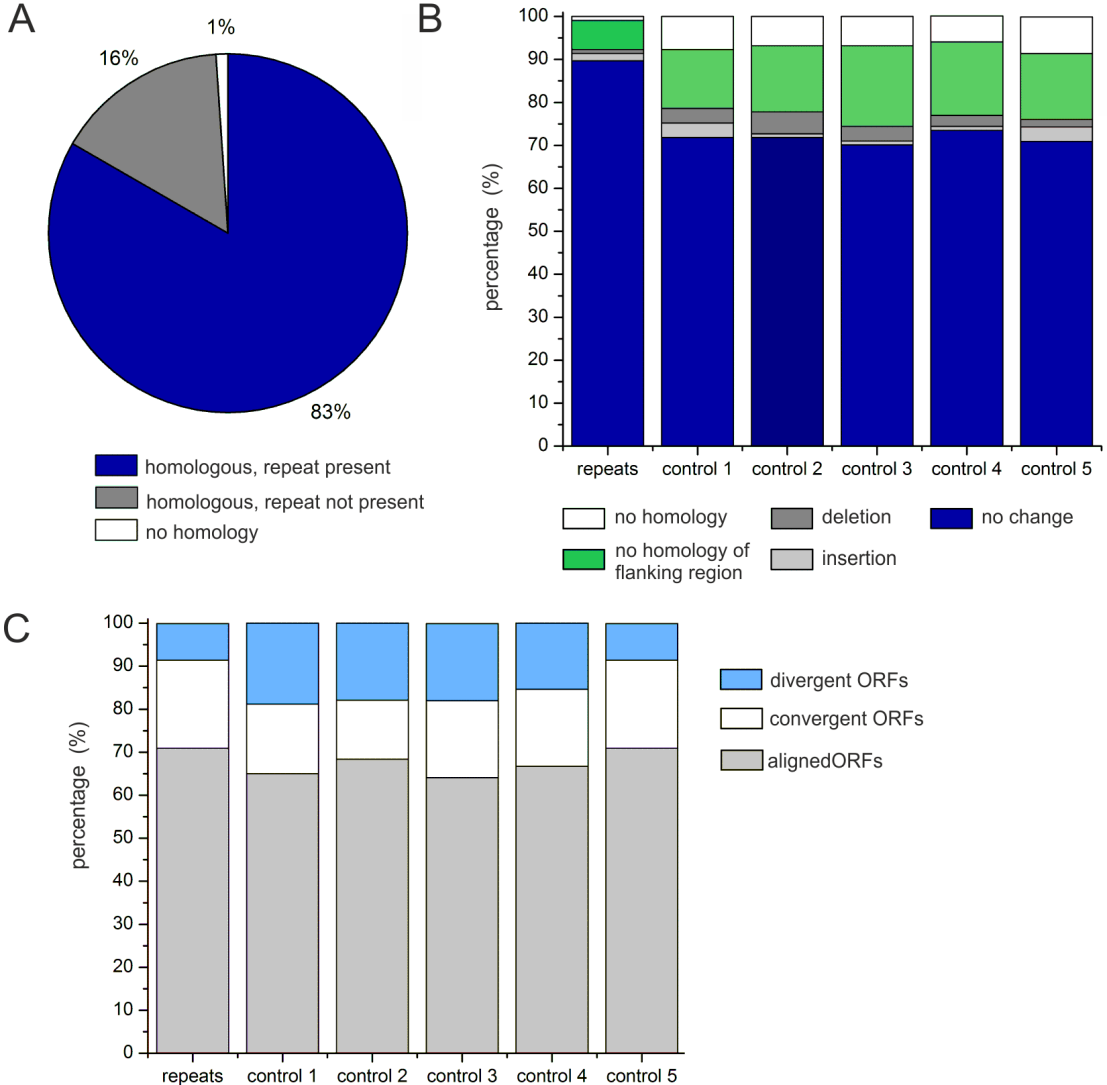
Sequence comparison between repeat containing regions in *Xcc* and *Xac*. (A): Presence of repeats patterns in *Xac* for repeat containing sequences from *Xcc*. Homologous repeats are depicted in blue, absent or mutated repeats depicted in gray, non-homologous alignments in white. (B) Analysis of changes of the identity of the neighboring genes for intergenic repeats from *Xcc* in comparison to *Xac*. Perfect alignments are grouped as “no change” (blue). Deletions (dark gray) or insertions (light gray) into intergenic regions were detected. Alignments showing only homology for one neighboring ORFs were grouped as “flanking region changes” (green). Non-homologous alignments are shown in white. (C) Orientation of neighboring genes relative to intergenic regions are shown for the repeat-containing intergenic regions from *Xcc* and the randomly chosen control sets 1–5. Sequences of control 5 were chosen to reflect the orientation of genes as found for the repeat containing intergenic regions in *Xcc*. ORFs can be either aligned (gray), convergent (white) or divergent (blue).

SSRs have been implicated as locations of genomic instability [1, 9, 37, 71, 72]. We used nucleotide blast (algorithm: blastn) to compare sequence similarity between the close relatives *Xcc* and *Xac* in repeat containing regions. Therefore all intergenic region containing a repeat and the complete neighboring ORFs, or complete ORFs containing an intragenic repeat of *Xcc* were aligned against the *Xac* genome (Table A in S5 File). We first assessed whether repeats from *Xcc* were also represented by G-rich repeat patterns at the same position in the *Xac* genome. 83% of the repeats were also present in the same gene context in *Xac*. For 16% we could not detect a G-rich pattern in the alignment or the G-rich stretch was strongly mutated. In two cases no alignment was possible between *Xcc* and *Xac* (Fig 6A). Furthermore we noticed differences in the length of the repeats between the two organisms, however the type of the repeat (singular repeat or inverted repeat pair) was usually preserved.

Next, we assessed changes of the identity of the neighboring genes for the repeats located in intergenic regions only (117 regions) (Fig 6B). Sequences were therefore grouped into the following categories according to their degree of sequence variability: “No homology” refers to all cases in which sequence alignment was impossible, “no homology of flanking region” refers to all cases in which one ORF was homologous, but the other neighboring ORF including the intergenic region was not homologous. We further distinguished between insertions of fragments comprising one to several genes in the intergenic region and deletions of neighboring genes. Finally, alignments with high similarity throughout were grouped as “no change”. For comparison we carried out the same analysis with 260 randomly chosen intergenic regions from *Xcc* that did not contain GGGAATC repeats (Table B in S5 File). From this pool of controls we randomly assembled three control sets with 117 sequences each (control 1 –control 3). In addition we assembled a fourth control set that mimics the overall distribution of the repeats along the *Xcc* genome (control). We found that 90% of the repeats were located between the same ORFs in *Xcc* and *Xac* (Fig 6B). Deletions or insertions in the intergenic regions, changes in flanking regions as well as no homology in the overall alignment were rare, altogether accounting to 10%. In contrast, these fractions of genomic changes were considerably higher in the random control sets accounting to roughly 30%. We analysed the statistical relevance of the data presented in Fig 6B by carrying out one-sample t-tests for each category. Using the 5 frequency values for each category in the five controls as background, the probability of observing a value equal to, higher or lower than the repeat group was calculated. The t-test for the category "no change" between the repeat group and the five control groups shows significance with a p-value = 2.857e-06.

This indicates that overall the investigated repeats are located at more conserved genomic locations. This finding is in contrast to the genomic instability of many previously characterized SSRs. When analyzing the orientation of the neighboring ORFs of the repeat set and the control 1–4, we noticed a bias for the control groups containing more intergenic regions located between divergent ORFs. To rule out an effect of this arrangement on our analysis in Fig 6B, we assembled a fifth control in which the orientation of the neighboring ORFs with respect to the intergenic region is the same as for the repeat sample (control 5) (Fig 6C). Also for control 5 we found a higher fraction of deletions, insertions and changes in the flanking regions in comparison to the repeat set (Fig 6B).

### Analysis of whole transcriptome sequencing data of *Xac*

Xanthomonads are plant pathogens. Since we identified the heptameric G-rich repeats in the genus *Xanthomonas* but not in other γ-proteobacteria, we considered a possible role of these putative G4-forming sequences in controlling a pathogenesis-related mechanism. Recently, whole transcriptome sequencing data became publicly available for *Xac* grown in full medium “NB” and hypersensitive response-elicitating medium “XVM2”, the latter mimicking plant infection [53]. Jalan et al. identified 229 differentially expressed genes (≥3 fold up- or down-regulation) in XVM2 in comparison to NB. Reviewing this data we found that among the 173 up-regulated genes in XVM2 only 5 genes were associated with repeats (*aroG*, *kdpC*, *asnC*, *suc1*, *fecA*). Likewise of the 119 down-regulated genes 6 were connected with repeats /*cheA*, *flhB*, *cheV*, *flgA*, *cysJ*, *xac3999*). However, these genes did not show drastic changes in expression levels, nor do they exclusively feature very prominent members of repeats or show a trend regarding orientation of the differentially expressed gene to the respective repeat.

In addition to a clear preference for 4 units, a strong bias for repeats downstream of ORFs to be localized in very close proximity of the stop codon or even overlapping with the ORF had been noticed (see Fig 5). In order to gain insight into whether the repeats are transcribed and whether they play a role in transcription termination we assessed the location of the repeat sequences on transcripts by investigating the available RNA sequencing data of *Xac* grown in NB full medium (sample NB_2) [53] (S6 File).

**Fig 7:**
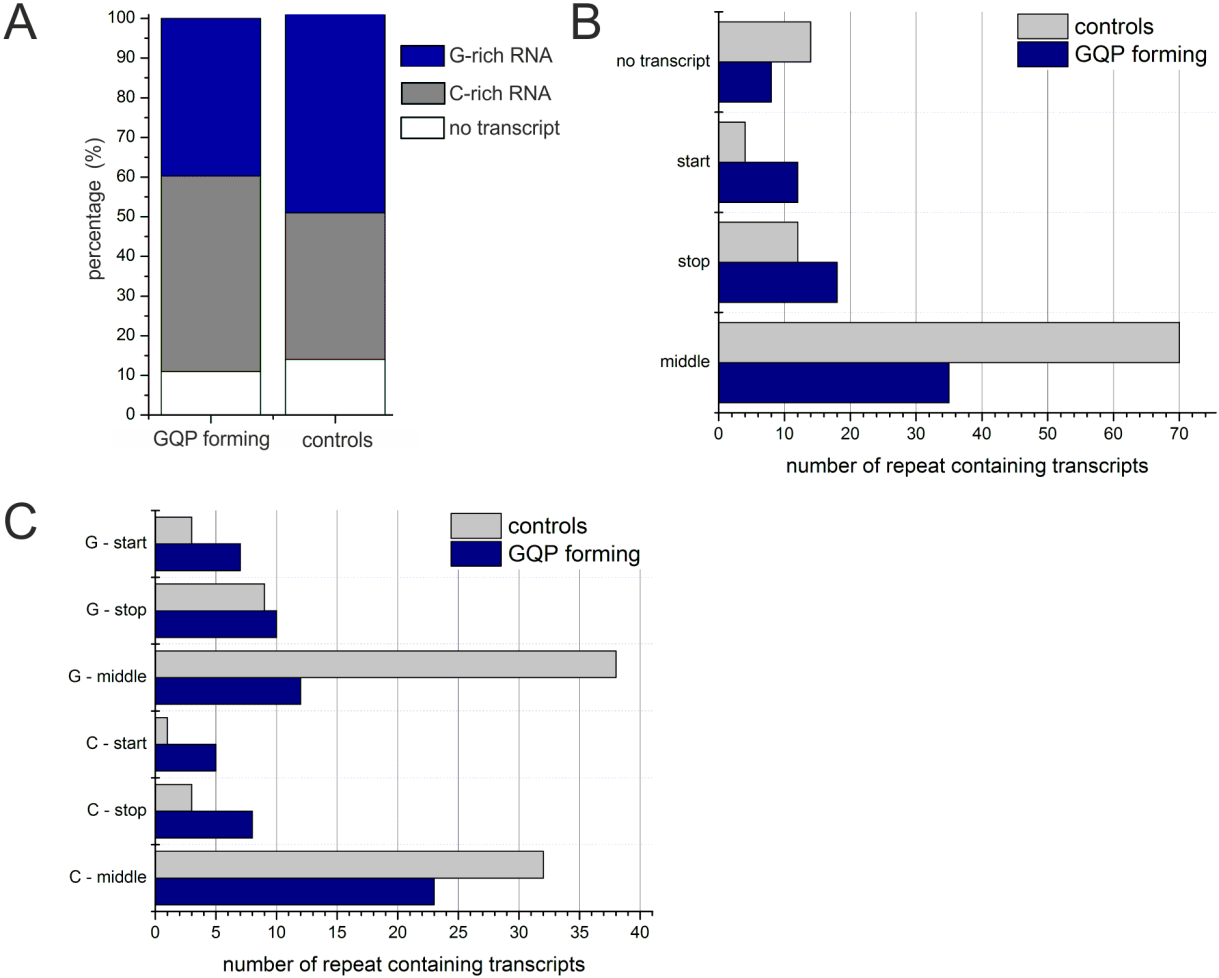
Analysis of repeat containing transcripts of *Xac* grown in NB medium. Analysis of assembled transcripts of *Xac* grown in NB medium that mapped to repeat containing regions. Control repeats are shown in gray, putative G4 forming repeats are shown in blue. The G4 forming set contains 65 transcripts, the control set contains 86 transcripts. In addition 8 G4 forming repeats and 14 control repeats are shown in A, for which no transcript could be assembled. (A): The overall distribution of a repeat’s location on a transcript is shown. No transcript refers to repeats for which no transcript could be assembled. If a transcript was assembled, it may start within a repeat sequence (start), stop within a repeat sequence or maximum 30 nt after the repeat (stop) or the repeat may be located somewhere in the middle of transcript (middle). (B) The analysis of A is further split up to show whether the G- or C-rich strand was found in the respective transcript.

First it was determined whether all repeat sequences are part of assembled transcripts. Of the 183 repeat sequences in *Xac* 24 repeats could not be assigned to a transcript in the analyzed sample. All transcripts mapping to repeats within coding regions were excluded from the following analysis and all repeats unable to fold putative G-quadruplexes with a G-tract of 3 guanines were allocated to a control set. In case of tandemly inverted repeats each repeat was analyzed individually. In the G4 group 49.3% showed the C-rich sequence on the transcript, 39.7% the G-rich sequence and for 11% of the repeats no transcript had been assembled. 37% of the control set showed the C-rich sequence on the transcript, 50% the G-rich sequence and for 14% of the repeats no transcript had been assembled (Fig 7A).

Next the assembled transcripts were sorted into the following groups according to the location of the repeat sequence within the transcript: 1) the transcript starts within the repeat, 2) the transcript ends within the repeat sequence or shortly thereafter (max. 30 nt) and 3) the repeat sequences is located anywhere in the middle of the transcript. Generally, putative G4 forming sequences were under-represented in the middle of transcripts (Fig 7B). Interestingly, a transcript started or stopped more often in the G4 forming group than in the control group (Fig 7B). This effect was observed no matter if the G-rich or C-rich strand was found on the transcript (Fig 7C).

## Discussion

GGGAATC / GGGGA(C/T)T repeat sequences are very abundant in xanthomonads and cyanobacteria. We investigated the occurrence of these motifs in the *Xcc*, *Xac* and *Ana* genomes. They represent a special type of SSR as in addition to being repetitive sequences they also have the capacity to form G4 structures. We found these repetitive patterns to be present all over the respective genomes with a strong bias for non-coding regions. Remarkably, a clear preference for a unit size of four was detected, which corresponds to the minimum number of G-tracts needed for G4 formation. Using CD spectroscopy we were able to show that repeat-comprising DNA oligonucleotides readily formed secondary structures with moderate to very high thermodynamic stabilities and a clear preference for K^+^, demonstrating that the adopted structures in presence of K^+^ are G4s. In addition we observed characteristic spectral changes that suggest i-motif formation of the complementary C-rich oligonucleotide even at mildly acidic pH of 6.5. Increasing ionic strength did not disturb i-motif formation. In case of inverted repeats there is the possibility of formation of stem-loop structures as well as G4s, both secondary structures may also compete with each other. It is unclear whether such possible non-canonical nucleic acid structures are formed at the DNA or RNA level in the bacteria. However, analysis of RNA sequencing data published by Jalan et al. [53] showed that the majority of the repeat sequences in *Xac* are in fact transcribed. The G- as well as the C-rich strand was found to be part of transcripts. While DNA as well as RNA G4s exist, formation of an i-motif on RNA level is much less favored compared to G4s [73] as RNA i-motifs have been shown to be less stable than their DNA counterparts [74, 75].

A preference for these G-rich repeats to be located in close proximity to the ORF either upstream on the non-coding strand or downstream on the coding strand was detected in all three organisms. These locations are prone to allow for gene regulatory effects. A variety of possible cellular functions have been attributed to G4s as has been reviewed by Bochman et al. [10]. For instance putative regulative roles of G4 structures formed during transcription involve blocking of transcription via inhibition of the polymerase, facilitating transcription by keeping the DNA strands separated, or even promotion or repression of transcription by recruitment of G4 binding proteins that may in turn interact with the RNA polymerase. Recently, we showed hat in *E*. *coli* G4 sequences can have activating as well as inhibitory effects on gene expression that largely depend on the exact location of the quadruplex-forming sequence element within the promoter region or at the ribosomal binding site [21]. Gene regulatory effects have also been observed for SSRs involved in phase variation, e.g. by overlapping with binding sites of regulatory proteins or variation of spacing between promoter elements [76, 77]. However, we were not able to identify a role of the studied repeats in gene regulation.

Generally, we found repeats located between divergent ORFs to be under-represented. In this case G-rich repeats may overlap with promoter regions of several genes. Possible secondary structure formation or repeat expansion in this region may interfere with the promoter function of both genes. Under-representation of G-rich motifs at such a position may indicate that formation of non-canonical nucleic acid structures by the repeats might well be possible in vivo and therefore be avoided in this particular region. This goes hand in hand with repeats being underrepresented on the coding strand within ORFs in all three organisms. Apart from restrictions due to the coding function of the ORF, G4 formation may cause ribosome stalling or induce frame-shifts.[78, 79] Generally, Lin and Kussell found SSRs to be suppressed in the middle of coding regions in prokaryotes, but enriched near the termini. SSRs were especially over-representated close to the N-terminus indicating involvement in phase variation by frame-shifting [80].

Analysis of the repeat-associated genes in all three organisms showed them to be randomly distributed across the different functional gene classes. Repeats involved in phase variation have been shown to be associated with cell surface structures such as antigens [22, 23, 77, 81]. In addition a G4 sequence in *Neisseria gonorrhoeae* has been shown to promote antigenic variation [82–84]. While genes encoding cell wall and pili components were among the repeat-associated genes, the great number of genes belonging to general metabolism pathways makes a role of GGGAATC and GGGGA(C/T)T repeats in phase variation unlikely. The genus *Xanthomonas* shows a high degree of host plant specificity and may even show tissue specificity. In addition to infecting different dicotyledonous hosts, *Xcc* invades the vascular system of the plant while *Xac* infects the mesophyll tissue [45]. However repeats were often found associated to similar genes in *Xac* and *Xcc* and not exclusive to pathogenicity-related genes. This makes a role of the repeats in pathogenicity or pathogen-host interactions unlikely.

While the majority of repeats are found between the same genes in *Xcc* and *Xac*, we found extensive length and sequence variation of the intergenic patterns even between these closely related organisms. It was hypothesized that the increased abundance of heptameric repeats in bacteria might be related to the size of the DNA segment that interacts with the active site of the DNA polymerase, which may lead to increased occurrence of polymerase slippage for this pattern type [33]. Joukhadar and Jighly hypothesized that microsatellites may even grant more stable flanking genes. SSRs may be able to discard weak DNA polymerases, thereby increasing the opportunity of the flanking genes to be replicated by more stable DNA polymerases [85]. In contrast to other SSRs, the sequences investigated here seem to be associated with genomic regions with increased genomic stability. While the over-representation of GGGAATC and GGGGA(C/T)T repeats in *Xcc*, *Xac* and *Ana*, respectively, is a remarkable feature of these prokaryotes, a potential functional role of these peculiar repeat motifs still remains to be elucidated.

## Supporting Information

S1 File G4 sequences in *Xanthomonas campestris pv*. *campestris* ATCC 33913 on plus strand (**Table A**). G4 sequences in *Xanthomonas campestris pv*. *campestris* ATCC 33913 on minus strand (**Table B**). G4 sequences in *Nostoc* sp. PCC7120 on plus strand (**Table C**). G4 sequences in *Nostoc* sp. PCC7120 on minus strand (**Table D**).
(DOCX)

S2 File GGGAATC Repeats in *Xanthomonas campestris pv*. *campestris* ATCC 33913 (**Table A**). GGGAATC Repeats in *Xanthomonas axonopodis pv*. *ctri* str. 306 (**Table B**). GGGGA(C/T)T Repeats in *Nostoc* sp. PCC7120 (**Table C**).
(DOCX)

S3 File DNA Oligonucleotides (**Table A**). Longest Repeats in *Xcc* (**Table B** *)*. Longest Repeats in *Xac* (**Table C** *)*. CD spectra of (GGGAATC)_3_GGG variants with G to T mutations in G-tract (**Fig A**). Melting temperatures of G-quadruplex structures (**Table D**). Melting profiles of G-rich repeat oligos at pH 7.5 (**Fig B**) Melting temperatures of structures formed by C-rich repeat oligonucleotides (**Table E**). CD spectra of C-rich repeat oligos in Na-acetate buffer (**Fig C**). Melting profiles of C-rich repeat oligos in Na-acetate buffer (**Fig D**). Distance of repeats to neighboring ORFs for potential quadruplex forming sequences in *Xcc* (**Fig E**).
(DOCX)

S4 File Classification of repeat associated genes according to KEGG Pathways. (DOCX)

S5 File Sequence comparison between repeat containing regions in *Xcc* and *Xac* (**Table A**). Control sequences used for sequence comparison between *Xcc* and *Xac* (**Table B**).
(DOCX)

S6 File Analysis of RNA sequencing data for repeat-containing transcripts. (DOCX)
