# Supplementary material for: Investigation of a Quadruplex-Forming Repeat Sequence Highly Enriched in Xanthomonas and Nostoc sp
Source: PLoS One. 2015 Dec 22;10(12):e0144275. doi: 10.1371/journal.pone.0144275 (PMC4692102; doi:10.1371/journal.pone.0144275)
Supplement: S1 File — G4 sequences in Xanthomonas campestris pv. campestris ATCC 33913 on plus strand (Table A). G4 sequences in Xanthomonas campestris pv. campestris ATCC 33913 on minus strand (Table B). G4 sequences in Nostoc sp. PCC7120 on plus strand (Table C). G4 sequences in Nostoc sp. PCC7120 on minus strand (Table D). (DOCX) [file pone.0144275.s001.docx]

**Supporting Information 1:**

**Contents:**

S1 Table A: G4 sequences in *Xanthomonas campestris pv. campestris* ATCC 33913 on plus strand

S1 Table B: G4 sequences in *Xanthomonas campestris pv. campestris* ATCC 33913 on minus strand

S1 Table C: G4 sequences in *Nostoc* sp. PCC7120 on plus strand

S1 Table D: G4 sequences in *Nostoc* sp. PCC7120 on minus strand

S1 Table A: G4 sequences in Xanthomonas campestris pv. campestris ATCC 33913 on plus strand

Table shows the potential G4 forming sequences on the plus strand of the chromosome of *Xcc* obtained from ProQuad Database (http://quadbase.igib.res.in/). The following query parameters had been used: pattern G, stem size G3 and loop size L1-5, genomic location: all. Sequence of the G4 pattern, position on the chromosome and total length are stated.

| **sequence name** | **sequence (5‘-3‘)** | **start** | **end** | **length** | **strand** |
| --- | --- | --- | --- | --- | --- |
| Xcc_QFind_001 | GGGCGGGGAATCGGGAGTGGGGAATCGGG | 18075 | 18103 | 29 | plus |
| Xcc_QFind_002 | GGGCGGGCTGCGGGTCGGG | 27023 | 27041 | 19 | plus |
| Xcc_QFind_003 | GGGCGGGCTGCGGGTCGGG | 27023 | 27041 | 19 | plus |
| Xcc_QFind_004 | GGGGCTGGGAATCGGGAATGGGGAATGGGG | 39485 | 39514 | 30 | plus |
| Xcc_QFind_005 | GGGAGCGGGGTGAGGGTAGGG | 59774 | 59794 | 21 | plus |
| Xcc_QFind_006 | GGGCTGGGCCTGTGGGCCCTGGG | 94445 | 94467 | 23 | plus |
| Xcc_QFind_007 | GGGCGGGCAGGGCGGGG | 197009 | 197025 | 17 | plus |
| Xcc_QFind_008 | GGGAGAGGGGTTGGGGTGAGGG | 198664 | 198685 | 22 | plus |
| Xcc_QFind_009 | GGGAGAGGGGTTGGGGTGAGGG | 198862 | 198883 | 22 | plus |
| Xcc_QFind_010 | GGGCAAGCGGGCCGGGCTCGGG | 244362 | 244383 | 22 | plus |
| Xcc_QFind_011 | GGGAGTGGGCATTCGGGATTGGGG | 253701 | 253724 | 24 | plus |
| Xcc_QFind_012 | GGGAGGGGGAAGGGTCCGGG | 299602 | 299621 | 20 | plus |
| Xcc_QFind_013 | GGGCTGGGGACGTGGGGAGGG | 322964 | 322984 | 21 | plus |
| Xcc_QFind_014 | GGGAGAGGGGTTGGGGTGAGGG | 358845 | 358866 | 22 | plus |
| Xcc_QFind_015 | GGGAGAGGGGTTGGGGTGAGGGTACGGG | 530840 | 530867 | 28 | plus |
| Xcc_QFind_016 | GGGAGAGGGGTTGGGGTGAGGG | 535727 | 535748 | 22 | plus |
| Xcc_QFind_017 | GGGTGACCGGGGTGGGCTCGGG | 550482 | 550503 | 22 | plus |
| Xcc_QFind_018 | GGGAGAGGGGTTGGGGTGAGGG | 557604 | 557625 | 22 | plus |
| Xcc_QFind_019 | GGGAATAGGGAGTCGGGAATGGGGAATGGGTAGATGCGGG | 622544 | 622583 | 40 | plus |
| Xcc_QFind_020 | GGGAATCGGGAGTAGGGAATCGGG | 630440 | 630463 | 24 | plus |
| Xcc_QFind_021 | GGGAAACGGGAATCGGGAATCGGG | 671018 | 671041 | 24 | plus |
| Xcc_QFind_022 | GGGGAATGGGGAATCGGGAATCGGGAATGGG | 873070 | 873100 | 31 | plus |
| Xcc_QFind_023 | GGGAATCGGGAATCGGGAATCGGG | 875248 | 875271 | 24 | plus |
| Xcc_QFind_024 | GGGCGGGAGTCGGGATTGGG | 883219 | 883238 | 20 | plus |
| Xcc_QFind_025 | GGGGTGGGGAAGGGAGTCGGGAATGGGGGTTGGGG | 920684 | 920718 | 35 | plus |
| Xcc_QFind_026 | GGGCCTGGGCTTCGGGAGGCTGGG | 945424 | 945447 | 24 | plus |
| Xcc_QFind_027 | GGGCTCGGGCGGGAAGCGGG | 980516 | 980535 | 20 | plus |
| Xcc_QFind_028 | GGGAATGGGCAAGGGGAGGG | 992034 | 992053 | 20 | plus |
| Xcc_QFind_029 | GGGACCTTGGGACAGGGAAGCGGG | 996517 | 996540 | 24 | plus |
| Xcc_QFind_030 | GGGGAGGGCGGGACGGG | 1004176 | 1004192 | 17 | plus |
| Xcc_QFind_031 | GGGAGAGGGGTTGGGGTGAGGG | 1024610 | 1024631 | 22 | plus |
| Xcc_QFind_032 | GGGCCCGGGGTGGGCACGGGG | 1098788 | 1098808 | 21 | plus |
| Xcc_QFind_033 | GGGGCGGGATCGTGGGGGAGGG | 1118599 | 1118620 | 22 | plus |
| Xcc_QFind_034 | GGGAATCGGGAGTAGGGAATCGGG | 1152989 | 1153012 | 24 | plus |
| Xcc_QFind_035 | GGGAGAGGGGTTGGGGTGAGGG | 1241786 | 1241807 | 22 | plus |
| Xcc_QFind_036 | GGGCTGCGGGAACTGGGTGAGCGGG | 1246341 | 1246365 | 25 | plus |
| Xcc_QFind_037 | GGGAATGGGGAATCGGGAGTGGGG | 1253794 | 1253817 | 24 | plus |
| Xcc_QFind_038 | GGGAATCGGGAGTAGGGAGTCGGG | 1338603 | 1338626 | 24 | plus |
| Xcc_QFind_039 | GGGGCCGGGATTCGGGAATCGGG | 1394250 | 1394272 | 23 | plus |
| Xcc_QFind_040 | GGGAATCGGGAGTTGGGAGTGGGG | 1487167 | 1487190 | 24 | plus |
| Xcc_QFind_041 | GGGCGGGCCGGGCGAGGG | 1518521 | 1518538 | 18 | plus |
| Xcc_QFind_042 | GGGCAGCGGGCCAGGGCAGTGGGTCGGG | 1523820 | 1523847 | 28 | plus |
| Xcc_QFind_043 | GGGGTGGCGGGGTGGGGCGTGGG | 1551430 | 1551452 | 23 | plus |
| Xcc_QFind_044 | GGGGCGGGGAATCGGGAATCGGGAGTTGGGAATGGG | 1556024 | 1556059 | 36 | plus |
| Xcc_QFind_045 | GGGATGGGAATGGGGAATGGGGAATCGGGAATGGGG | 1572753 | 1572788 | 36 | plus |
| Xcc_QFind_046 | GGGACTCGGGAGTCGGGAATGGGG | 1583930 | 1583953 | 24 | plus |
| Xcc_QFind_047 | GGGAATCGGGAGTGGGGAATGGGG | 1585223 | 1585246 | 24 | plus |
| Xcc_QFind_048 | GGGAGTTGGGAATCGGGAATCGGGAATCGGG | 1648354 | 1648384 | 31 | plus |
| Xcc_QFind_049 | GGGCATGGGCGGGGTGGGCTCGTGGG | 1657903 | 1657928 | 26 | plus |
| Xcc_QFind_050 | GGGAGTAGGGAATTGGGAATGGG | 1735666 | 1735688 | 23 | plus |
| Xcc_QFind_051 | GGGAATGGGGAGTAGGGAATGGGG | 1737257 | 1737280 | 24 | plus |
| Xcc_QFind_052 | GGGTAGCAGGGAGGGGGAGGG | 1783065 | 1783085 | 21 | plus |
| Xcc_QFind_053 | GGGCGGCTGGGTGGGCGGCGGG | 1804610 | 1804631 | 22 | plus |
| Xcc_QFind_054 | GGGAGAGGGGTTGGGCTGAGGGTACGGGG | 1830927 | 1830955 | 29 | plus |
| Xcc_QFind_055 | GGGCGGGGCGCGGGCATAGGG | 1862299 | 1862319 | 21 | plus |
| Xcc_QFind_056 | GGGAGCGGGCGAGGGCGGG | 1942699 | 1942717 | 19 | plus |
| Xcc_QFind_057 | GGGCAGGGCAGGGCCCTGGG | 1972630 | 1972649 | 20 | plus |
| Xcc_QFind_058 | GGGAATTGGGAATGGGCAGTCGGG | 1985700 | 1985723 | 24 | plus |
| Xcc_QFind_059 | GGGTGGGGCAGGGCTGGG | 2058074 | 2058091 | 18 | plus |
| Xcc_QFind_060 | GGGGCTGGGATTCGGGATTCGGGATTCGGGATTGGGGATTGGGG | 2126832 | 2126875 | 44 | plus |
| Xcc_QFind_061 | GGGAATCGGGAGTTGGGAATGGGG | 2133319 | 2133342 | 24 | plus |
| Xcc_QFind_062 | GGGGCCGGGGTGCTGGGAGTGGG | 2133356 | 2133378 | 23 | plus |
| Xcc_QFind_063 | GGGAGAGGGGTTGGGGTGAGGG | 2213758 | 2213779 | 22 | plus |
| Xcc_QFind_064 | GGGAATGGGGAATCGGGATTCGGG | 2237555 | 2237578 | 24 | plus |
| Xcc_QFind_065 | GGGAATCGGGAATGGGGAATCGGG | 2283899 | 2283922 | 24 | plus |
| Xcc_QFind_066 | GGGAATGGGGAATCGGGAATCGGGAATGGGG | 2291127 | 2291157 | 31 | plus |
| Xcc_QFind_067 | GGGGGAGGGGCGGGAGCGGGAGCGGGAGCGGG | 2389302 | 2389333 | 32 | plus |
| Xcc_QFind_068 | GGGCAGGGGCAGGGGCAGGG | 2423162 | 2423181 | 20 | plus |
| Xcc_QFind_069 | GGGGCCCGGGTCAAGGGGTAGGGGATAGGGG | 2436173 | 2436203 | 31 | plus |
| Xcc_QFind_070 | GGGCACCGGGCTGGGCGTGGG | 2522047 | 2522067 | 21 | plus |
| Xcc_QFind_071 | GGGCGGGGTAAAGGGCGTGGGCTTGGG | 2578839 | 2578865 | 27 | plus |
| Xcc_QFind_072 | GGGTCGCTGGGTCGCTGGGTCGCTGGGTCGCCGGGTCGCTGGGTCGCCGGGTCGCCGGGTCGCCGGG | 2653586 | 2653652 | 67 | plus |
| Xcc_QFind_073 | GGGCTGTGGGCTGTGGGCTGTGGGCTGTGGGCTGTGGGCAGTGGGCTGTGGG | 2657700 | 2657751 | 52 | plus |
| Xcc_QFind_074 | GGGAATCGGGAGTTGGGAATCGGGAATGGGGGGTCCATCAGGG | 2782275 | 2782317 | 43 | plus |
| Xcc_QFind_075 | GGGAATGGGGAATGGGGAATCGGGAGTCGGG | 2812110 | 2812140 | 31 | plus |
| Xcc_QFind_076 | GGGAGAGGGGTTGGGGTGAGGG | 2822739 | 2822760 | 22 | plus |
| Xcc_QFind_077 | GGGAATCGGGAAGCGGGAAGCGGGAAGCGGGAAGCGGGAAGCGGG | 2927343 | 2927387 | 45 | plus |
| Xcc_QFind_078 | GGGAGAGGGGTTGGGGTGTGGGTACGGG | 2938442 | 2938469 | 28 | plus |
| Xcc_QFind_079 | GGGGAATCGGGAGTCGGGAGTCGGG | 3095822 | 3095846 | 25 | plus |
| Xcc_QFind_080 | GGGGCGGGAATCGGGAATCGGGAATCGGG | 3103829 | 3103857 | 29 | plus |
| Xcc_QFind_081 | GGGGCGGGGATTGGGGATTCGGGAGTTGGG | 3195766 | 3195795 | 30 | plus |
| Xcc_QFind_082 | GGGAATTGGGAATTGGGAATTGGGAATTGGGAATTGGGAATTGGGAATCGGGAATTGGGAATTGGGAATTGGGAATTGGGAATTGGGAATTGGGAATTGGGAATCGGGAATCGGGAATCGGGAATCGGGAATCGGGAATCGGGAATCGGG | 3257029 | 3257178 | 150 | plus |
| Xcc_QFind_083 | GGGATTGGGGAATCGGGATTCGGGAATCGGGAATCGGGAATCGGG | 3312014 | 3312058 | 45 | plus |
| Xcc_QFind_084 | GGGGGTGGGCGGGATCAGGG | 3435952 | 3435971 | 20 | plus |
| Xcc_QFind_085 | GGGAGAGGGGTTGGGGTGAGGG | 3507362 | 3507383 | 22 | plus |
| Xcc_QFind_086 | GGGGATTGGGGAATCGGGAGTCGGG | 3567830 | 3567854 | 25 | plus |
| Xcc_QFind_087 | GGGGCGCCGGGAATCGGGAATCGGGAATGGGG | 3572830 | 3572861 | 32 | plus |
| Xcc_QFind_088 | GGGCCGGGAGTGGGGAATCGGGAATCGGGAATGGG | 3573457 | 3573491 | 35 | plus |
| Xcc_QFind_089 | GGGAGAGGGGTTGGGGTGAGGG | 3646986 | 3647007 | 22 | plus |
| Xcc_QFind_090 | GGGAGAGGGGTTGGGGTGAGGG | 3647140 | 3647161 | 22 | plus |
| Xcc_QFind_091 | GGGAGAGGGGTTGGGGTGAGGG | 3647294 | 3647315 | 22 | plus |
| Xcc_QFind_092 | GGGAGAGGGGTTGGGGTGAGGG | 3647447 | 3647468 | 22 | plus |
| Xcc_QFind_093 | GGGGCCGGGAATTGGGAGTGGGGAATCGGG | 3671162 | 3671191 | 30 | plus |
| Xcc_QFind_094 | GGGATTGGGGAGTGGGGAATCGGG | 3765772 | 3765795 | 24 | plus |
| Xcc_QFind_095 | GGGAATCGGGAGTGGGGAATCGGGAATGGG | 3768821 | 3768850 | 30 | plus |
| Xcc_QFind_096 | GGGGGAATGGGGAATGGGGAATGGGGAATGGGGAATGGGG | 3788315 | 3788354 | 40 | plus |
| Xcc_QFind_097 | GGGTGGGCTGGGTCATGGG | 3825428 | 3825446 | 19 | plus |
| Xcc_QFind_098 | GGGATGGGCATGGGTGGGG | 3885086 | 3885104 | 19 | plus |
| Xcc_QFind_099 | GGGAATGGGGAATCGGGAATGGGG | 3995713 | 3995736 | 24 | plus |
| Xcc_QFind_100 | GGGATTCGGGATTCGGGATTCGGGATTCGGGATTCGGGATTCGGGATTCGGGATCGGGAATCGGGAATCGGGAATCGGGAATCGGGAATCGGGAATCGGGAATCGGGAATCGGGAATCGGGAATCGGG | 3998009 | 3998136 | 128 | plus |
| Xcc_QFind_101 | GGGAATCGGGAGTCGGGAGTCGGG | 4130504 | 4130527 | 24 | plus |
| Xcc_QFind_102 | GGGCCGGGATTGGGGAATTGGGAGTGGGG | 4131830 | 4131858 | 29 | plus |
| Xcc_QFind_103 | GGGAGAGGGGTTGGGGTGAGGG | 4162354 | 4162375 | 22 | plus |
| Xcc_QFind_104 | GGGAATCGGGAATCGGGAATCGGG | 4223226 | 4223249 | 24 | plus |
| Xcc_QFind_105 | GGGAGAGGGGTTGGGGTGAGGG | 4274614 | 4274635 | 22 | plus |
| Xcc_QFind_106 | GGGAGTCGGGATTGGGGATTGGGG | 4330647 | 4330670 | 24 | plus |
| Xcc_QFind_107 | GGGAATCGGGATTGGGGAATCGGG | 4350196 | 4350219 | 24 | plus |
| Xcc_QFind_108 | GGGTTAGCGGGTTAGCGGGTTAGCGGGTTAGCGGG | 4350252 | 4350286 | 35 | plus |
| Xcc_QFind_109 | GGGGATTGGGGAGTTGGGAATCGGG | 4355522 | 4355546 | 25 | plus |
| Xcc_QFind_110 | GGGCTGGGATTTGGGAGTCGGGATTGGGG | 4456910 | 4456938 | 29 | plus |
| Xcc_QFind_111 | GGGAATCGGGAGTTGGGAATAGGGAATAGGG | 4535056 | 4535086 | 31 | plus |
| Xcc_QFind_112 | GGGTCGTTGGGTCGTTGGGTCGTTGGGTCGTTGGG | 4606201 | 4606235 | 35 | plus |
| Xcc_QFind_113 | GGGAATCGGGAATCGGGAATCGGGAGTTGGGAATCGGG | 4631079 | 4631116 | 38 | plus |
| Xcc_QFind_114 | GGGAGTGGGGAATGGGGATTGGGGATTGGGGATTGGG | 4644872 | 4644908 | 37 | plus |
| Xcc_QFind_115 | GGGAGAGGGGTTGGGGTGAGGG | 4703163 | 4703184 | 22 | plus |
| Xcc_QFind_116 | GGGAGAGGGGTTGGGGTGAGGGTACGGGG | 4774956 | 4774984 | 29 | plus |
| Xcc_QFind_117 | GGGATTGGGGATTGGGGATTGGGGATTGGGAAGCGGGAAGCGGG | 4795013 | 4795056 | 44 | plus |
| Xcc_QFind_118 | GGGAGTGGGGAATCGGGAGTGGGG | 4818828 | 4818851 | 24 | plus |
| Xcc_QFind_119 | GGGAGAGGGGTTGGGGTGAGGG | 4828056 | 4828077 | 22 | plus |
| Xcc_QFind_120 | GGGAATCGGGAATCGGGAATCGGGAATCGGGAATGGGGAATGGGGAGTCGGG | 4841550 | 4841601 | 52 | plus |
| Xcc_QFind_121 | GGGAGAGGGGTTGGGGTGAGGG | 4885389 | 4885410 | 22 | plus |
| Xcc_QFind_122 | GGGCGCTGGGCATTGGGATGGG | 4951658 | 4951679 | 22 | plus |
| Xcc_QFind_123 | GGGTAGTGGGGTTATGGGGGATGGG | 4956893 | 4956917 | 25 | plus |
| Xcc_QFind_124 | GGGAAAGGGGTTGGGGTGAGGGTACGGGG | 4957045 | 4957073 | 29 | plus |
| Xcc_QFind_125 | GGGAGAGGGGTTGGGTGAGGGTACTGGG | 4957220 | 4957247 | 28 | plus |
| Xcc_QFind_126 | GGGAATTGGGAATTGGGAATTGGGAATTGGGAATTGGGAATTGGGGATTGGGGATTGGGGATTGGGGATGCGGGATGCGGGATGCGGG | 4960617 | 4960704 | 88 | plus |

S1 Table B: G4 sequences in *Xanthomonas campestris pv. campestris* ATCC 33913 on minus strand

Table shows the potential G4 forming sequences on the minus strand of the chromosome of *Xcc* obtained from ProQuad Database (http://quadbase.igib.res.in/). The following query parameters had been used: pattern C, stem size C3 and loop size L1-5, genomic location: all. Sequence of the G4 pattern, position on the chromosome and total length are stated.

| **sequence name** | **sequence (5‘-3‘)** | **start** | **end** | **length** | **strand** |
| --- | --- | --- | --- | --- | --- |
| Xcc_QFind_127 | CCCGAGTCCCTATTCCCGATTCCC | 39573 | 39596 | 24 | plus |
| Xcc_QFind_128 | CCCTCACCCCAACCCCTCTCCC | 59949 | 59970 | 22 | plus |
| Xcc_QFind_129 | CCCGAATCCCCACTCCCGATTCCC | 110162 | 110185 | 24 | plus |
| Xcc_QFind_130 | CCCGATTCCCGATTCCCGATTCCCGATTCCCGATTCCCGATTCCCGATTCCCAATCCCCAATCCCCAATCCCC | 131225 | 131297 | 73 | plus |
| Xcc_QFind_131 | CCCACCCAGCCCGAACTCCC | 138060 | 138079 | 20 | plus |
| Xcc_QFind_132 | CCCGATTCCCGATTCCCGATTCCCGATTCCCGATTCCCGATTCCCGATTCCCGATTCCCGATTCCCGATTCCCGATTCCCGATTCCCGATTCCCC | 232448 | 232542 | 95 | plus |
| Xcc_QFind_133 | CCCGCCCGCCCGCCCGCCC | 237156 | 237174 | 19 | plus |
| Xcc_QFind_134 | CCCGCCCCACGCCCCTCAGCCCC | 245161 | 245183 | 23 | plus |
| Xcc_QFind_135 | CCCTCACCCCAACCCCTCTCCC | 283592 | 283613 | 22 | plus |
| Xcc_QFind_136 | CCCCAATCCCGATTCCCCAATCCCTGCCCC | 284323 | 284352 | 30 | plus |
| Xcc_QFind_137 | CCCCAATCCCCAATCCCCAATCCCCAATCCCCAATCCCCAATCCCC | 529751 | 529796 | 46 | plus |
| Xcc_QFind_138 | CCCTCACCCCAACCCCTCTCCC | 536114 | 536135 | 22 | plus |
| Xcc_QFind_139 | CCCGATTCCCGATTCCCGATTCCCGACTCCCGACTCCCGACTCCCGATTCCCGACTCCCGACTCCCGATTCCCGATTCCCGACTCCCGATTCCCAATTCCCAATTCCCAATTCCCAATTCCCGAATCCCGAATCCCGAATCCCGAATCCCGAATCCC | 619000 | 619156 | 157 | plus |
| Xcc_QFind_140 | CCCAATCCCGAATCCCGTTTCCCCAATCCC | 669513 | 669542 | 30 | plus |
| Xcc_QFind_141 | CCCCACTCCCGATTCCCGATTCCC | 671117 | 671140 | 24 | plus |
| Xcc_QFind_142 | CCCTTGCCCGCCCACCC | 714097 | 714113 | 17 | plus |
| Xcc_QFind_143 | CCCATTCCCTATTCCCCACTCCC | 786171 | 786193 | 23 | plus |
| Xcc_QFind_144 | CCCGATTCCCGACTCCCGATTCCC | 786842 | 786865 | 24 | plus |
| Xcc_QFind_145 | CCCAAATCCCAAATCCCGACCCCAAAATCCC | 800973 | 801003 | 31 | plus |
| Xcc_QFind_146 | CCCCATTCCCGATTCCCAATTCCCAATTCCCGGCCTCCC | 801021 | 801059 | 39 | plus |
| Xcc_QFind_147 | CCCTATTCCCCTCTCCCTATTCCC | 834023 | 834046 | 24 | plus |
| Xcc_QFind_148 | CCCGATTCCCCTCTCCCGATTCCC | 840266 | 840289 | 24 | plus |
| Xcc_QFind_149 | CCCGACTCCCTATTCCCGATTCCCGATTCCCAACCC | 841527 | 841562 | 36 | plus |
| Xcc_QFind_150 | CCCGATTCCCGATTCCCGATTCCCGATTCCCGATTCCCGATTCCCGATTCCCGATTCCCGATTCCC | 848739 | 848804 | 66 | plus |
| Xcc_QFind_151 | CCCCACTCCCGATTCCCGATTCCC | 861770 | 861793 | 24 | plus |
| Xcc_QFind_152 | CCCTATTCCCGATTCCCCATTCCC | 873132 | 873155 | 24 | plus |
| Xcc_QFind_153 | CCCGATTCCCGATTCCCGATTCCCGATTCCCGATTCCCCAATCCCCAATCCCCAATCCCCAATCCCC | 924072 | 924138 | 67 | plus |
| Xcc_QFind_154 | CCCCAATCCCGATTCCCGAATCCC | 974517 | 974540 | 24 | plus |
| Xcc_QFind_155 | CCCGCAACCCGCAACCCGCAACCCGCAACCC | 1086822 | 1086852 | 31 | plus |
| Xcc_QFind_156 | CCCCATTCCCCATTCCCCATTCCC | 1117056 | 1117079 | 24 | plus |
| Xcc_QFind_157 | CCCCAATCCCGATTCCCGATTCCC | 1126981 | 1127004 | 24 | plus |
| Xcc_QFind_158 | CCCCAATCCCCATTCCCGATTCCC | 1153049 | 1153072 | 24 | plus |
| Xcc_QFind_159 | CCCGATTCCCGATTCCCGATTCCCGATTCCCGATTCCCGATTCCCGATTCCC | 1181203 | 1181254 | 52 | plus |
| Xcc_QFind_160 | CCCCGGCCCGATCCCGTTCCC | 1187109 | 1187129 | 21 | plus |
| Xcc_QFind_161 | CCCTGGCCCGGTCCCATCCCGCGGCCCC | 1212016 | 1212043 | 28 | plus |
| Xcc_QFind_162 | CCCCATTCCCTATTCCCCATTCCC | 1338724 | 1338747 | 24 | plus |
| Xcc_QFind_163 | CCCGAATCCCGAATCCCGAATCCC | 1354818 | 1354841 | 24 | plus |
| Xcc_QFind_164 | CCCGAATCCCGAATCCCGAATCCCGAATCCCGAATCCC | 1367116 | 1367153 | 38 | plus |
| Xcc_QFind_165 | CCCCATTCCCCATTCCCCATTCCCCATTCCC | 1367193 | 1367223 | 31 | plus |
| Xcc_QFind_166 | CCCCATTCCCAGTCCCGATTCCCGATTCCCC | 1401172 | 1401202 | 31 | plus |
| Xcc_QFind_167 | CCCGATTCCCCACTCCCGATTCCC | 1556153 | 1556176 | 24 | plus |
| Xcc_QFind_168 | CCCGATTCCCCACTCCCAATTCCC | 1572932 | 1572955 | 24 | plus |
| Xcc_QFind_169 | CCCGATTCCCGATTCCCGATTCCCAATTCCC | 1584033 | 1584063 | 31 | plus |
| Xcc_QFind_170 | CCCGTACCCTCACCCCAACCCCTCTCCC | 1620106 | 1620133 | 28 | plus |
| Xcc_QFind_171 | CCCATTCCCGAATCCCAATTCCCGATTCCCGCCC | 1648464 | 1648497 | 34 | plus |
| Xcc_QFind_172 | CCCTTCTCCCGATTCCCGATTCCC | 1722828 | 1722851 | 24 | plus |
| Xcc_QFind_173 | CCCAATTCCCCACTCCCGATTCCC | 1735780 | 1735803 | 24 | plus |
| Xcc_QFind_174 | CCCGACTCCCGATTCCCGATTCCC | 1769134 | 1769157 | 24 | plus |
| Xcc_QFind_175 | CCCTAATCCCGACTGCCCAATCCCCGCCCACTCACCC | 1782434 | 1782470 | 37 | plus |
| Xcc_QFind_176 | CCCCAATCCCCAATCCCCAATCCCCAATCCCCAATCCCCAATCCC | 1808614 | 1808658 | 45 | plus |
| Xcc_QFind_177 | CCCCGTACCCTCACCCCAACCCCTCTCCC | 1823440 | 1823468 | 29 | plus |
| Xcc_QFind_178 | CCCGATTCCCCAATCCCGATTCCC | 1824542 | 1824565 | 24 | plus |
| Xcc_QFind_179 | CCCTCACCCCAACCCCTCTCCC | 1976201 | 1976222 | 22 | plus |
| Xcc_QFind_180 | CCCTCACCCCAACCCCTCTCCC | 1976411 | 1976432 | 22 | plus |
| Xcc_QFind_181 | CCCGTACCCTCACCCCAACCCCTCTCCC | 2173029 | 2173056 | 28 | plus |
| Xcc_QFind_182 | CCCGATTCCCGATTCCCGATTCCCGATTCCC | 2224288 | 2224318 | 31 | plus |
| Xcc_QFind_183 | CCCGAATCCCAATCCCCAATCCCCAATCCCC | 2227242 | 2227272 | 31 | plus |
| Xcc_QFind_184 | CCCTACTCCCGATTCCCCATTCCC | 2284005 | 2284028 | 24 | plus |
| Xcc_QFind_185 | CCCGATTCCCCACTCCCGATTCCCC | 2288362 | 2288386 | 25 | plus |
| Xcc_QFind_186 | CCCCACTCCCGATTCCCTATTCCC | 2291250 | 2291273 | 24 | plus |
| Xcc_QFind_187 | CCCCACCCTCGGCCCTCCC | 2519788 | 2519806 | 19 | plus |
| Xcc_QFind_188 | CCCTCACCCCAACCCCTCTCCC | 2526775 | 2526796 | 22 | plus |
| Xcc_QFind_189 | CCCTCACCCCAACCCCTCTCCC | 2527045 | 2527066 | 22 | plus |
| Xcc_QFind_190 | CCCTCACCCCAACCCCTCTCCC | 2527315 | 2527336 | 22 | plus |
| Xcc_QFind_191 | CCCACGCCCATCCCGCGCGCCCGCATGCCC | 2607649 | 2607678 | 30 | plus |
| Xcc_QFind_192 | CCCCCAATCCCAATCCCGACTCCCCAATCCCCGCCC | 2614865 | 2614900 | 36 | plus |
| Xcc_QFind_193 | CCCCAATCCCGATTCCCGAATCCC | 2615823 | 2615846 | 24 | plus |
| Xcc_QFind_194 | CCCTGTCCCGCCCTTCCC | 2617149 | 2617166 | 18 | plus |
| Xcc_QFind_195 | CCCGTACCCTCACCCCAACCCCTCTCCC | 2645393 | 2645420 | 28 | plus |
| Xcc_QFind_196 | CCCGTACCCTCACCCCAACCCCCGCGCCCCGGCCC | 2650644 | 2650678 | 35 | plus |
| Xcc_QFind_197 | CCCTCACCCCAACCCCTCTCCC | 2657598 | 2657619 | 22 | plus |
| Xcc_QFind_198 | CCCTCACCCCAACCCTCTCCC | 2657899 | 2657919 | 21 | plus |
| Xcc_QFind_199 | CCCGAATCCCGATTCCCCATTCCCC | 2669604 | 2669628 | 25 | plus |
| Xcc_QFind_200 | CCCGCCCTGCTGCCCCTGCCC | 2735287 | 2735307 | 21 | plus |
| Xcc_QFind_201 | CCCGAATCCCGACTCCCAACTCCCCACCC | 2744870 | 2744898 | 29 | plus |
| Xcc_QFind_202 | CCCCATTCCCGATTCCCCACTCCC | 2812194 | 2812217 | 24 | plus |
| Xcc_QFind_203 | CCCCGTACCCTCACCCCAACCCCTCTCCC | 2813457 | 2813485 | 29 | plus |
| Xcc_QFind_204 | CCCGATTCCCCACTCCCGATTCCCTGCCCC | 2813531 | 2813560 | 30 | plus |
| Xcc_QFind_205 | CCCGATCCCGCCCTGCCCC | 2857208 | 2857226 | 19 | plus |
| Xcc_QFind_206 | CCCAGGCCCGTGCCCTGCCCC | 2879851 | 2879871 | 21 | plus |
| Xcc_QFind_207 | CCCACCCCGCCCTTACCCGAGCCC | 3047539 | 3047562 | 24 | plus |
| Xcc_QFind_208 | CCCAGCCCCGCCCTCGCCCC | 3084315 | 3084334 | 20 | plus |
| Xcc_QFind_209 | CCCCATTCCCGATTCCCGATTCCCC | 3089495 | 3089519 | 25 | plus |
| Xcc_QFind_210 | CCCCACTCCCGATTCCCGATTCCC | 3089582 | 3089605 | 24 | plus |
| Xcc_QFind_211 | CCCCGTACCCTCACCCCAACCCCTCTCCC | 3120310 | 3120338 | 29 | plus |
| Xcc_QFind_212 | CCCTCACCCCAACCCCTCTCCC | 3120469 | 3120490 | 22 | plus |
| Xcc_QFind_213 | CCCCGTACCCTCACCCCAACCCCTCTCCC | 3120613 | 3120641 | 29 | plus |
| Xcc_QFind_214 | CCCCCTACCCTTACCCCAACCCCTCTCCC | 3120764 | 3120792 | 29 | plus |
| Xcc_QFind_215 | CCCTGCCCGGCCCGCCC | 3185066 | 3185082 | 17 | plus |
| Xcc_QFind_216 | CCCCATTCCCGATTCCCTATTCCC | 3266449 | 3266472 | 24 | plus |
| Xcc_QFind_217 | CCCTCACCCCAACCCCTCTCCC | 3276311 | 3276332 | 22 | plus |
| Xcc_QFind_218 | CCCGATTCCCGATTCCCGATTCCCGACTCCCGATTCCCGATTCCCGATTCCCGACTCCCGACTCCCGATTCCCGATTCCCGATTCCCGATTCCC | 3279743 | 3279836 | 94 | plus |
| Xcc_QFind_219 | CCCGGCTACCCAACCACCCTGCCC | 3464804 | 3464827 | 24 | plus |
| Xcc_QFind_220 | CCCGATTCCCGATTCCCGATTCCC | 3572899 | 3572922 | 24 | plus |
| Xcc_QFind_221 | CCCTGACCCACCCCCGCCC | 3582159 | 3582177 | 19 | plus |
| Xcc_QFind_222 | CCCCATCCCCGATTCCCGATTCCC | 3594311 | 3594334 | 24 | plus |
| Xcc_QFind_223 | CCCCGTACCCTCACCCCAACCCCTCTCCC | 3631418 | 3631446 | 29 | plus |
| Xcc_QFind_224 | CCCCACTCCCGATTCCCAATTCCC | 3671549 | 3671572 | 24 | plus |
| Xcc_QFind_225 | CCCCATTCCCGACTCCCCATTCCC | 3732759 | 3732782 | 24 | plus |
| Xcc_QFind_226 | CCCCTTTCCCGATTCCCGATTCCCATGACCC | 3768901 | 3768931 | 31 | plus |
| Xcc_QFind_227 | CCCTGCCGCCCTACCCCACCC | 3770579 | 3770599 | 21 | plus |
| Xcc_QFind_228 | CCCCACTCCCAAATCCCCAATCCCC | 3846663 | 3846687 | 25 | plus |
| Xcc_QFind_229 | CCCCAATCCCGATTCCCTAATCCC | 3876290 | 3876313 | 24 | plus |
| Xcc_QFind_230 | CCCCAATCCCGACTCCCCAATCCC | 3992821 | 3992844 | 24 | plus |
| Xcc_QFind_231 | CCCCACTCCCGATTCCCCATTCCC | 3995821 | 3995844 | 24 | plus |
| Xcc_QFind_232 | CCCCGCCCGCACCCGCCACCCC | 3999909 | 3999930 | 22 | plus |
| Xcc_QFind_233 | CCCACCTCCCCGACCCGCCC | 4034053 | 4034072 | 20 | plus |
| Xcc_QFind_234 | CCCAGCGCCCTGCCCTCACCC | 4092042 | 4092062 | 21 | plus |
| Xcc_QFind_235 | CCCACCCGTCCCCAGCCC | 4094421 | 4094438 | 18 | plus |
| Xcc_QFind_236 | CCCAATTCCCGACTCCCCATTCCCAGCCCCC | 4130943 | 4130973 | 31 | plus |
| Xcc_QFind_237 | CCCCACTCCCGATTCCCGATTCCCGTCCCC | 4132126 | 4132155 | 30 | plus |
| Xcc_QFind_238 | CCCTCACCCCAACCCCTCTCCCC | 4163833 | 4163855 | 23 | plus |
| Xcc_QFind_239 | CCCAAATCCCGACTCCCCAATCCCCGCCCC | 4168057 | 4168086 | 30 | plus |
| Xcc_QFind_240 | CCCGATTCCCGACTCCCCATTCCC | 4223304 | 4223327 | 24 | plus |
| Xcc_QFind_241 | CCCCGCCCTCCCCCGCCC | 4351341 | 4351358 | 18 | plus |
| Xcc_QFind_242 | CCCCACTCCCGAATCCCCAATCCCAGCCC | 4356689 | 4356717 | 29 | plus |
| Xcc_QFind_243 | CCCCACTCCCAATTCCCAATTCCC | 4360243 | 4360266 | 24 | plus |
| Xcc_QFind_244 | CCCGCCAGCCCGCGCCCACCC | 4368304 | 4368324 | 21 | plus |
| Xcc_QFind_245 | CCCGTGCCCAACTACCCGCGCCC | 4398115 | 4398137 | 23 | plus |
| Xcc_QFind_246 | CCCATCCCCTCCCATCCCCCTCCCATCTCCCC | 4414745 | 4414776 | 32 | plus |
| Xcc_QFind_247 | CCCGATTCCCCATTCCCGATTCCCCATTTCCCATTCCCGACTCCCCATTCCCCC | 4414802 | 4414855 | 54 | plus |
| Xcc_QFind_248 | CCCTTGCCCAGCAGCCCCGCTCCC | 4490030 | 4490053 | 24 | plus |
| Xcc_QFind_249 | CCCACTCCCGATTCCCGATTCCCAATTCCC | 4506871 | 4506900 | 30 | plus |
| Xcc_QFind_250 | CCCGGCCCAGACCCAAGGCCC | 4527198 | 4527218 | 21 | plus |
| Xcc_QFind_251 | CCCGATTCCCGATTCCCGACTCCC | 4535152 | 4535175 | 24 | plus |
| Xcc_QFind_252 | CCCGATTCCCTATTCCCCATTCCCGCCC | 4570734 | 4570761 | 28 | plus |
| Xcc_QFind_253 | CCCTCTCCCCAACCCCTCTCCC | 4636698 | 4636719 | 22 | plus |
| Xcc_QFind_254 | CCCTCACCCCAACCCCTCTCCC | 4694133 | 4694154 | 22 | plus |
| Xcc_QFind_255 | CCCCGTACCCTCACCCCTACCCCTTTCCC | 4782696 | 4782724 | 29 | plus |
| Xcc_QFind_256 | CCCCGCACCCTCACCCCTACCCCTCTCCC | 4782919 | 4782947 | 29 | plus |
| Xcc_QFind_257 | CCCCGTACCCTCACCCCTACCCCTTTCCC | 4783140 | 4783168 | 29 | plus |
| Xcc_QFind_258 | CCCCGTACCCTCACCCCTCTCCC | 4783363 | 4783385 | 23 | plus |
| Xcc_QFind_259 | CCCCGCACCCTCACCCCTCTCCC | 4783578 | 4783600 | 23 | plus |
| Xcc_QFind_260 | CCCGATTCCCCACTCCCGATTCCCGGCCCC | 4819020 | 4819049 | 30 | plus |
| Xcc_QFind_261 | CCCTCACCCCAACCCCTCTCCC | 4828319 | 4828340 | 22 | plus |
| Xcc_QFind_262 | CCCCGCCCCACGGCCCGCCC | 4854780 | 4854799 | 20 | plus |
| Xcc_QFind_263 | CCCCGTACCCTCACCCCAACCCC | 4858938 | 4858960 | 23 | plus |
| Xcc_QFind_264 | CCCTCACCCCAACCCCTCTCCC | 4861972 | 4861993 | 22 | plus |
| Xcc_QFind_265 | CCCTCACCCCAACCCCTCTCCC | 4912012 | 4912033 | 22 | plus |
| Xcc_QFind_266 | CCCGTACCCTCACCCGCCC | 5008533 | 5008551 | 19 | plus |
| Xcc_QFind_267 | CCCTCACCCCAACCCCTCTCCC | 5009078 | 5009099 | 22 | plus |
| Xcc_QFind_268 | CCCCAACCCAGGCCCGCACCC | 5017538 | 5017558 | 21 | plus |
| Xcc_QFind_269 | CCCTCACCCCAACCCCTCTCCC | 5039683 | 5039704 | 22 | plus |
| Xcc_QFind_270 | CCCCACTCCCGATTCCCGATTCCC | 5052381 | 5052404 | 24 | plus |
| Xcc_QFind_271 | CCCCAATCCCGATTCCCCAATCCC | 5062169 | 5062192 | 24 | plus |

S1 Table C: G4 sequences in *Nostoc* sp. PCC7120 on plus strand

Table shows the potential G4 forming sequences on the plus strand of the chromosome of *Ana* obtained from ProQuad Database (http://quadbase.igib.res.in/). The following query parameters had been used: pattern G, stem size G3-5 and loop size L1-7, genomic location: all. Sequence of the G4 pattern, position on the chromosome and total length are stated.

| **sequence name** | **sequence (5'-3')** | **start** | **end** | **length** | **strand** |
| --- | --- | --- | --- | --- | --- |
| Nos_Qfind_001 | GGGTACTGGGTACTGGGTACTGGG | 19188 | 19211 | 24 | plus |
| Nos_Qfind_002 | GGGGATTAGGGACTGGGGATTAGGGATTGGG | 19250 | 19280 | 31 | plus |
| Nos_Qfind_003 | GGGGACTGGGGACTGGGTATGGGGACTGGGGAAGTGGAGGG | 111837 | 111877 | 41 | plus |
| Nos_Qfind_004 | GGGTAGACTTGGGAACGGGTAGTGGGG | 117834 | 117860 | 27 | plus |
| Nos_Qfind_005 | GGGGGATTGGGTAATGGGTAATGGG | 121146 | 121170 | 25 | plus |
| Nos_Qfind_006 | GGGTGTAAGGGTATAGGGGTGTAAGGG | 144493 | 144519 | 27 | plus |
| Nos_Qfind_007 | GGGGGCAGGGGGCAGGGGAAGCAGGGGGCAGGGGGCAGGGAGCAGGGGGCAGGGGG | 169561 | 169616 | 56 | plus |
| Nos_Qfind_008 | GGGCTACGGGGAAGTGGGTGAGGGCTTGGGCGATGGGTAGGG | 172675 | 172716 | 42 | plus |
| Nos_Qfind_009 | GGGGTGTGGGGGTATAGGGGTATGGGGG | 186460 | 186487 | 28 | plus |
| Nos_Qfind_010 | GGGAACGGGGGACTGGGGATTGGGGATTGGG | 227599 | 227629 | 31 | plus |
| Nos_Qfind_011 | GGGGGTATAGGGGTGTAGGGGTATGGGGG | 232561 | 232589 | 29 | plus |
| Nos_Qfind_012 | GGGTTTTGGAGGGTTTGGGGTTTGGGG | 237958 | 237984 | 27 | plus |
| Nos_Qfind_013 | GGGTGTAAGGGTATAGGGGTGTAGGGG | 247969 | 247995 | 27 | plus |
| Nos_Qfind_014 | GGGTATAGGGGTTTAAGGGTATAGGGG | 251717 | 251743 | 27 | plus |
| Nos_Qfind_015 | GGGGACTGGGGATCGGGGATTGGGAAGGTGGGGACTGGGG | 279040 | 279079 | 40 | plus |
| Nos_Qfind_016 | GGGGTGTAGAGGGGTAGGGGTGTAGGGGTGTAAGGG | 287651 | 287686 | 36 | plus |
| Nos_Qfind_017 | GGGGATTGGGGATTGGGGACTGGGGATTGGGGACTGGG | 290772 | 290809 | 38 | plus |
| Nos_Qfind_018 | GGGATAAGGGGTAATGGGGAGTGGGGAGAGACGGGG | 313607 | 313642 | 36 | plus |
| Nos_Qfind_019 | GGGGATTGGGGATTGGGGATTGGGTGCTGGGGATTGGGGATTGGGGATTGGG | 377483 | 377534 | 52 | plus |
| Nos_Qfind_020 | GGGTGTAGGGGTGTAAGGGTGTAAGGGTGTGAGGGTGTAAGGGTGTGAGGGTGTAGGGTGTAAGGGTGTAGGGTGTAGGGTGTGAGGG | 440446 | 440533 | 88 | plus |
| Nos_Qfind_021 | GGGGTGTGGGGGTGTAGGGGTGTGGGGGTGTGGGGG | 510668 | 510703 | 36 | plus |
| Nos_Qfind_022 | GGGAAAATAGGGGTGTAAGGGTTTAGGGGTGTAAGGG | 524997 | 525033 | 37 | plus |
| Nos_Qfind_023 | GGGGAGTAGGGATTGGGGACTGGGGAGCAGGGG | 575259 | 575291 | 33 | plus |
| Nos_Qfind_024 | GGGTATAGGGGTTGTAGGGGTTGTAGGGGTATAGGGGTTGTAGGGG | 577372 | 577417 | 46 | plus |
| Nos_Qfind_025 | GGGTAGGGCGGGCCGTCGGGGG | 584834 | 584855 | 22 | plus |
| Nos_Qfind_026 | GGGGATTGGGGATTGGGGATTGGGGATTGGGGATTGGGGATTGGGGATTGGGGATTGGGGATTGGGGATTGGGGATTGGGGATTGGGGATTGGGGATTGGGGATTGGGGATTGGGGATTGGGGATTGGGGATTGGGGATTGGGGATTGGGGATTGGGGATTGGGGATTGGGGATTGGGG | 619299 | 619477 | 179 | plus |
| Nos_Qfind_027 | GGGGATTGGGGACTGGGGATTGGGGATTGGGGATTAGGGATTGGGGATTGGGGATTAGGGATTGGGGATTGGGGATTAGGGATTGGGGATTAGGGATTGGGGATTAGGGATTGGGGATTAGGGATTGGG | 643135 | 643263 | 129 | plus |
| Nos_Qfind_028 | GGGGTTGGGGATTAGGGATTAGGGATGGGG | 735378 | 735407 | 30 | plus |
| Nos_Qfind_029 | GGGGAGCAGGGGAGCAGGGAGCAGGGG | 811864 | 811890 | 27 | plus |
| Nos_Qfind_030 | GGGAGCAGGGGGCGGGGAGCGGAGGGG | 884561 | 884587 | 27 | plus |
| Nos_Qfind_031 | GGGGCTGGGGACTGGGGACTGGGGGCTGGG | 911056 | 911085 | 30 | plus |
| Nos_Qfind_032 | GGGGATAAGGGGTAATGGGGATTGGG | 925146 | 925171 | 26 | plus |
| Nos_Qfind_033 | GGGATGTGGGTGATGTGGGCGATGGGGGCGATGTAGGG | 973086 | 973123 | 38 | plus |
| Nos_Qfind_034 | GGGTAATTAGGGGAGGGAGCAGGG | 986310 | 986333 | 24 | plus |
| Nos_Qfind_035 | GGGGACTGGGGACTGGGAAGGGGGAAATGGGGGG | 994299 | 994332 | 34 | plus |
| Nos_Qfind_036 | GGGGGTGTAAGGGGATGAGGGTATAGGGG | 1043929 | 1043957 | 29 | plus |
| Nos_Qfind_037 | GGGTAATGGGTAATGGGTAATGGG | 1094102 | 1094125 | 24 | plus |
| Nos_Qfind_038 | GGGGTGTAAGGGTGTAAGGGTGTAAGGGTGTAGGGG | 1107146 | 1107181 | 36 | plus |
| Nos_Qfind_039 | GGGAACTGATGGGACAAAGGGGACTGGGG | 1155921 | 1155949 | 29 | plus |
| Nos_Qfind_040 | GGGGAAGCAGGGGAAGCATGGGAAGCAGGGGGGCAGGGGG | 1195637 | 1195676 | 40 | plus |
| Nos_Qfind_041 | GGGAGCAGGGGGCAGGGAGCAGGGGG | 1201666 | 1201691 | 26 | plus |
| Nos_Qfind_042 | GGGAACGGGGATTAGGGACTGGGG | 1213009 | 1213032 | 24 | plus |
| Nos_Qfind_043 | GGGAGCAGGGGGAGCAGGGGAAGCAGGGGGAGTAGGGGAAGCAGGGGG | 1213052 | 1213099 | 48 | plus |
| Nos_Qfind_044 | GGGATTTAGGGGTGTAGGGGTGTAGGGGTTTAGGGGTTTAGGGGTTTAGGGG | 1214511 | 1214562 | 52 | plus |
| Nos_Qfind_045 | GGGTAATGGGTAGTGGGTAATGGGTAATGGG | 1217470 | 1217500 | 31 | plus |
| Nos_Qfind_046 | GGGGACTGGGGAGTGGGTACTGGGGAGTGGGTACTGGGGAGTGGGG | 1219856 | 1219901 | 46 | plus |
| Nos_Qfind_047 | GGGTTTAGGGGCTTAGGGGTTTAGGGG | 1230448 | 1230474 | 27 | plus |
| Nos_Qfind_048 | GGGTGTAAGGGTGTAAGGGTGTAGGGGGG | 1240046 | 1240074 | 29 | plus |
| Nos_Qfind_049 | GGGGGTGTGGGGGTATAGGGGTTTAGGGGTTTAGGGGTTTAGGGG | 1262563 | 1262607 | 45 | plus |
| Nos_Qfind_050 | GGGTGGCGCGGGAAGTAGGGACGGAAGGG | 1313844 | 1313872 | 29 | plus |
| Nos_Qfind_051 | GGGTAGCGCGGGAGGTGGGAACAGAAGGGAAATTGGG | 1314948 | 1314984 | 37 | plus |
| Nos_Qfind_052 | GGGTGGCGCGGGAGGTGGGAACCGAGGG | 1315500 | 1315527 | 28 | plus |
| Nos_Qfind_053 | GGGATTGGGGATTGGGTACTGGGGATTGGGTACTGGGGATTGGGTAATGGGGATTGGG | 1441553 | 1441610 | 58 | plus |
| Nos_Qfind_054 | GGGACTGGGGACTGGGGACTGGGGATTGGGGGAGCAGGGG | 1470074 | 1470113 | 40 | plus |
| Nos_Qfind_055 | GGGTGTAAGGGTGTAGGGGTGTAAGGG | 1479285 | 1479311 | 27 | plus |
| Nos_Qfind_056 | GGGTATGGGTAATGGGTACTGGGTAATAGGGG | 1480386 | 1480417 | 32 | plus |
| Nos_Qfind_057 | GGGTAAGGGTATAGGGGTGTAAGGGTGTGAGGGTGTGAGGG | 1551672 | 1551712 | 41 | plus |
| Nos_Qfind_058 | GGGAATTAGGGACTGGGGACTGGGG | 1552219 | 1552243 | 25 | plus |
| Nos_Qfind_059 | GGGGGTTGGGTATCGGGGATTGGGGGTTGGGGATTGGGGGTTGGGGATTGGGGATTGGGGACTGGGGATTAGGGACTAGGGATTGGGGATTGGGGATTGGGGACTGGGGATTGGGGACTGGGGATTGGGGGTTAGGGACTGGG | 1575364 | 1575506 | 143 | plus |
| Nos_Qfind_060 | GGGGTGTAGGGGTATGGGGGTTTAGGGG | 1588631 | 1588658 | 28 | plus |
| Nos_Qfind_061 | GGGGATTAGGGACTGGGGATTAGGGACTGGGG | 1590989 | 1591020 | 32 | plus |
| Nos_Qfind_062 | GGGTATTGGGCGAAGCAGGGGAGGG | 1592914 | 1592938 | 25 | plus |
| Nos_Qfind_063 | GGGATTAGGGACTGGGGACTGGGGACTAGGGACTGGGGACTAGGG | 1595025 | 1595069 | 45 | plus |
| Nos_Qfind_064 | GGGGTTTAGGGGTTTAGGGGTGTAGGGG | 1624955 | 1624982 | 28 | plus |
| Nos_Qfind_065 | GGGTTTTAGGGGGATTGGGGACTGGGGATTGGGGACTGGGGATTGGGGACTGGG | 1638008 | 1638061 | 54 | plus |
| Nos_Qfind_066 | GGGGTTTAGGGGTTTAGGGGTGTGGGGGTGTAGGGG | 1663255 | 1663290 | 36 | plus |
| Nos_Qfind_067 | GGGTGTAGGGGTATAGGGGTGTAGGGGTATAGGGG | 1698500 | 1698534 | 35 | plus |
| Nos_Qfind_068 | GGGGGATTGGGTACTGGGGGCTGGGTACTGGGAATTGGG | 1715439 | 1715477 | 39 | plus |
| Nos_Qfind_069 | GGGTACTGGGGATTGGGTATTGGGGACTGGGG | 1808772 | 1808803 | 32 | plus |
| Nos_Qfind_070 | GGGTATAGGGGTGTAAGGGTGTAGGGG | 1885146 | 1885172 | 27 | plus |
| Nos_Qfind_071 | GGGCGCGGGTCATGGGTTTGTTGGG | 1904133 | 1904157 | 25 | plus |
| Nos_Qfind_072 | GGGAGATTTTGGGGTTTTTGGGGCCGAATGGGGCGGGG | 1907027 | 1907064 | 38 | plus |
| Nos_Qfind_073 | GGGTGTAGGGGTGTGAGGGTGTAAGGG | 1943961 | 1943987 | 27 | plus |
| Nos_Qfind_074 | GGGGGCAGGGAAGTAGGGAGCAGGGAAGGCTGGGGAGTAGGGGGACTGGGG | 2040265 | 2040315 | 51 | plus |
| Nos_Qfind_075 | GGGAGGGGGAGATAGGGGATGGGGGAGATAAAGGG | 2092793 | 2092827 | 35 | plus |
| Nos_Qfind_076 | GGGTAAAAGGGGTGTAAGGGTATAAGGGTATAGGGG | 2225641 | 2225676 | 36 | plus |
| Nos_Qfind_077 | GGGGATTGGGGACTGGGGACTGGGGACTGGGGATTGGGGATTGGGGATAAGGGGTAATGGGGATTTTGGG | 2236798 | 2236867 | 70 | plus |
| Nos_Qfind_078 | GGGTGTAAGGGGGTAAGGGTATAGGGGGGTAAGGG | 2238484 | 2238518 | 35 | plus |
| Nos_Qfind_079 | GGGGGTGTGAGGGTGTGAGGGTGTGAGGGTGTAAGGGTGTAAGGGTGTGAGGGTGTGAGGG | 2243140 | 2243200 | 61 | plus |
| Nos_Qfind_080 | GGGTGGCATGGGCGGCATGGGTGGTATGGGCGGTATGGGTGGCATGGGCGGTATGGGTGGTATGGG | 2267174 | 2267239 | 66 | plus |
| Nos_Qfind_081 | GGGAGCAGGGGGCAGGGAGCAAGGGGG | 2267259 | 2267285 | 27 | plus |
| Nos_Qfind_082 | GGGGCAGAGGGCAGGGGAGGCAGGGGG | 2270446 | 2270472 | 27 | plus |
| Nos_Qfind_083 | GGGGTACAGGGGTATAGGGGTATAGGGGTACAGGGGTACAGGGATACAGGGGTATAGGGG | 2310430 | 2310489 | 60 | plus |
| Nos_Qfind_084 | GGGACTGGGGATTGGGTACTGGGTATTGGGGATTGGGG | 2384108 | 2384145 | 38 | plus |
| Nos_Qfind_085 | GGGGGCAGGGGGCAGGGGGCAGGGG | 2390011 | 2390035 | 25 | plus |
| Nos_Qfind_086 | GGGGAATGGGGACTGGGGACTGGGGATTGGGGATTGGGTAATCGGG | 2401236 | 2401281 | 46 | plus |
| Nos_Qfind_087 | GGGGAATGGGTAATGGGTAATGGGTAATGGG | 2405984 | 2406014 | 31 | plus |
| Nos_Qfind_088 | GGGGGAGTGGGGAGTAGGGGAGCAGGGG | 2478134 | 2478161 | 28 | plus |
| Nos_Qfind_089 | GGGGAGTTGGGCGCGGGTTTCTGGG | 2520477 | 2520501 | 25 | plus |
| Nos_Qfind_090 | GGGATTGGGGATTGGGGATTGGGGACTGGG | 2521330 | 2521359 | 30 | plus |
| Nos_Qfind_091 | GGGTGTAAGGGGGTAAGGGTATAGGGG | 2630997 | 2631023 | 27 | plus |
| Nos_Qfind_092 | GGGGAAGGGGGCAGGGAGCAGGGGG | 2632274 | 2632298 | 25 | plus |
| Nos_Qfind_093 | GGGTTTAGGGGTATAAGGGTTTAGGGGTGTAGGGG | 2665841 | 2665875 | 35 | plus |
| Nos_Qfind_094 | GGGTGGCAGGGGGCAGGGGGCAGGGGAGCAGGGG | 2736428 | 2736461 | 34 | plus |
| Nos_Qfind_095 | GGGGGTGTAGGGGTGTGGGGGTGTAGGGG | 2740257 | 2740285 | 29 | plus |
| Nos_Qfind_096 | GGGTGTAGGGGTATGGGGGTATAAGGGTGTAGGG | 2795656 | 2795689 | 34 | plus |
| Nos_Qfind_097 | GGGGATTAGGGACTGGGAACTGGG | 2816288 | 2816311 | 24 | plus |
| Nos_Qfind_098 | GGGGACTAGGGACTGGGGACTGGG | 2817697 | 2817720 | 24 | plus |
| Nos_Qfind_099 | GGGGTGTAGGGGTATGGGGTTTAGGGGTTTAGGGG | 2857993 | 2858027 | 35 | plus |
| Nos_Qfind_100 | GGGATTAGGGATTAGGGATTGGGGATTGGGGACTGGGGATTGGGGATTGGGGATGAAGGGG | 2916857 | 2916917 | 61 | plus |
| Nos_Qfind_101 | GGGGCAGGGAGCAGGGAGCAGGG | 2945867 | 2945889 | 23 | plus |
| Nos_Qfind_102 | GGGGCTGGGCTGGGTTCATCTGGG | 2967173 | 2967196 | 24 | plus |
| Nos_Qfind_103 | GGGGAGCAGGGAGCAGGGAGCAGGGG | 3047814 | 3047839 | 26 | plus |
| Nos_Qfind_104 | GGGGGTAAGGGTATAGGGGTGTAAGGG | 3070748 | 3070774 | 27 | plus |
| Nos_Qfind_105 | GGGGACTGGGGACTGGGGACTGGGGACTGGGGACTGGGG | 3096498 | 3096536 | 39 | plus |
| Nos_Qfind_106 | GGGATTGGGGATTGGGGATTGGGGATTGGG | 3131530 | 3131559 | 30 | plus |
| Nos_Qfind_107 | GGGGTAATTGGGTGGGTTCGGGG | 3149838 | 3149860 | 23 | plus |
| Nos_Qfind_108 | GGGGACTGGGGATTGGGGACTGGG | 3151507 | 3151530 | 24 | plus |
| Nos_Qfind_109 | GGGATTGGGGATTGGGGATTGGGGACTGGGGATTGGGGATTGGGGATTGGGG | 3160483 | 3160534 | 52 | plus |
| Nos_Qfind_110 | GGGGAGTAGGGATAGGGCTGAGGGGG | 3161192 | 3161217 | 26 | plus |
| Nos_Qfind_111 | GGGGACTGGGGACTGGGGACTGGGGATTGGGGACTGGGGATTGGGGTGTCTAGGGAAATTATGGG | 3169803 | 3169867 | 65 | plus |
| Nos_Qfind_112 | GGGGTGTAGGGGTGTAGGGGTGTAGGGGTGTAGGGGGG | 3176455 | 3176492 | 38 | plus |
| Nos_Qfind_113 | GGGACTGGGGACTGGGGACTGGGGACTGGGGACTGGG | 3185044 | 3185080 | 37 | plus |
| Nos_Qfind_114 | GGGAGTAGGGAGTAGGGAGTAGGGAGTAGGG | 3187608 | 3187638 | 31 | plus |
| Nos_Qfind_115 | GGGCATGGGGTATGGGGCATGGGGCATGGGG | 3195367 | 3195397 | 31 | plus |
| Nos_Qfind_116 | GGGGGAGCAGGGGGAGAAGGGAGTGAGGG | 3244450 | 3244478 | 29 | plus |
| Nos_Qfind_117 | GGGGATGGGGTTGGGTAAGGGGTGTGGGG | 3247799 | 3247827 | 29 | plus |
| Nos_Qfind_118 | GGGCGTTGGGGTTGAGGGGTGGCGGG | 3277413 | 3277438 | 26 | plus |
| Nos_Qfind_119 | GGGGATTAGGGATTGGGGATTAGGG | 3304753 | 3304777 | 25 | plus |
| Nos_Qfind_120 | GGGGTATGGGGGTATAGGGGTATAGGGGTATAGGGGTATAGGGG | 3331081 | 3331124 | 44 | plus |
| Nos_Qfind_121 | GGGGACTGGGGACTGGGTATTAGGG | 3376351 | 3376375 | 25 | plus |
| Nos_Qfind_122 | GGGAGCAGGGGGCAGGGGGCAGGGGG | 3422017 | 3422042 | 26 | plus |
| Nos_Qfind_123 | GGGGAAGCAGGGGAGGCAGGGGAGGCAGGGGAGACAGGG | 3422053 | 3422091 | 39 | plus |
| Nos_Qfind_124 | GGGGCAGGGAGCAGGGGAGGCAGGGGAGGCAGGGGG | 3422725 | 3422760 | 36 | plus |
| Nos_Qfind_125 | GGGTAATAGTGGGATGTTGGGGATGCCGGG | 3462865 | 3462894 | 30 | plus |
| Nos_Qfind_126 | GGGTGTAAGGGTTTAAGGGGACAGGGGTGTAAGGGTGTAAGGGTGTAAGGG | 3471806 | 3471856 | 51 | plus |
| Nos_Qfind_127 | GGGTGTAAGGGGGTAAGGGTATAGGGG | 3542960 | 3542986 | 27 | plus |
| Nos_Qfind_128 | GGGGTGTAAGGGTGTGGGGGTGTAAGGG | 3585820 | 3585847 | 28 | plus |
| Nos_Qfind_129 | GGGGACTGGGGACTGGGGACTGGGGACTGGG | 3585960 | 3585990 | 31 | plus |
| Nos_Qfind_130 | GGGGTATAAGGGTGTAAGGGTGTAGGGGG | 3624990 | 3625018 | 29 | plus |
| Nos_Qfind_131 | GGGTAAAAATGGGAGGGCATAAAGGGAGCAGGGGGGG | 3625742 | 3625778 | 37 | plus |
| Nos_Qfind_132 | GGGGTGTAAGGGTGTGGGGGTGTAAGGGTATAGGGG | 3653465 | 3653500 | 36 | plus |
| Nos_Qfind_133 | GGGTTTAGGGGTGTAGGGGTATAGGGG | 3680289 | 3680315 | 27 | plus |
| Nos_Qfind_134 | GGGGTGTTGGGGTATAGGGGTTTAGGGGTATAGGGG | 3702701 | 3702736 | 36 | plus |
| Nos_Qfind_135 | GGGGAGATTGGGGAGTAGGGGAAGCAGGG | 3752253 | 3752281 | 29 | plus |
| Nos_Qfind_136 | GGGGAATGGGGATTAGGGATTAGGGAATGGGGATTAGGGATTAGGGATTAGGG | 3805354 | 3805406 | 53 | plus |
| Nos_Qfind_137 | GGGGGAGTAGGGAAGCAGGGGAGGCAGGGGG | 3847308 | 3847338 | 31 | plus |
| Nos_Qfind_138 | GGGGATTGGGGATTGGGGATTGGGGATTGGG | 3853236 | 3853266 | 31 | plus |
| Nos_Qfind_139 | GGGGATTGGGGATTGGGGATTGGGGATTGGGGATTGGGGATTGGGG | 3878954 | 3878999 | 46 | plus |
| Nos_Qfind_140 | GGGGTGTAGGGGTATAAGGGTGTAAGGGTGTAGGGG | 3885980 | 3886015 | 36 | plus |
| Nos_Qfind_141 | GGGGATTGGGGATTGGGTACTAGGGATTAGGGACTGGGG | 3919349 | 3919387 | 39 | plus |
| Nos_Qfind_142 | GGGTGTAGGGGTGCAAGGGTATAGGGG | 4009398 | 4009424 | 27 | plus |
| Nos_Qfind_143 | GGGGCAGGGGGAGCAGGGCAGGGGAGGTGGGG | 4014472 | 4014503 | 32 | plus |
| Nos_Qfind_144 | GGGGGCAGGGGGCAGGGGGCAGGGGGCAGGGGG | 4035466 | 4035498 | 33 | plus |
| Nos_Qfind_145 | GGGTATAGGGGTATAGGGGTATAGGG | 4036922 | 4036947 | 26 | plus |
| Nos_Qfind_146 | GGGGATTGGGGACTGGGGACTGGGTATTGGGGACTGGGGACTGGGTATTGGGTGTTGGG | 4038623 | 4038681 | 59 | plus |
| Nos_Qfind_147 | GGGTGTAAGGGTGTAGGGGTGTAAGGG | 4074307 | 4074333 | 27 | plus |
| Nos_Qfind_148 | GGGAAATAGGGATTAGGGAATAGGG | 4123598 | 4123622 | 25 | plus |
| Nos_Qfind_149 | GGGGCAGTTTGGGCTTGGGTGCGGG | 4173293 | 4173317 | 25 | plus |
| Nos_Qfind_150 | GGGGATAAGGGGTAATGGGGATTGGG | 4229894 | 4229919 | 26 | plus |
| Nos_Qfind_151 | GGGTGTGGGGGTTTAGGGATTTAGGGG | 4235405 | 4235431 | 27 | plus |
| Nos_Qfind_152 | GGGGATAAGGGGTAATGGGGATTGGG | 4239330 | 4239355 | 26 | plus |
| Nos_Qfind_153 | GGGGTGTACGGGTGTAAGGGTATAGGGGG | 4247372 | 4247400 | 29 | plus |
| Nos_Qfind_154 | GGGGATCGGGTATTGGGTATTGGG | 4262837 | 4262860 | 24 | plus |
| Nos_Qfind_155 | GGGGATTGGGGATTGGGGATTGGGGACTGGGG | 4265346 | 4265377 | 32 | plus |
| Nos_Qfind_156 | GGGTATAGGGGTGTAAGGGTGTAAGGGG | 4283660 | 4283687 | 28 | plus |
| Nos_Qfind_157 | GGGTGGGAAAATTTGGGTAGGG | 4299206 | 4299227 | 22 | plus |
| Nos_Qfind_158 | GGGACAGGGATTGGGGATTGGGGACTGGGGATTGGGGACTGGGGACTGGG | 4302919 | 4302968 | 50 | plus |
| Nos_Qfind_159 | GGGATTGGGGGTTGGGGACTGGGGACTGGGGGTTGGGTAAAGGGGATTGGGGATTGGG | 4304910 | 4304967 | 58 | plus |
| Nos_Qfind_160 | GGGGATTGGGGATTGGGGATTGGG | 4308402 | 4308425 | 24 | plus |
| Nos_Qfind_161 | GGGGATTGGGTACTGGGGAAGGTGGGGGCCCCTCTGGGGATAAGGGG | 4395567 | 4395613 | 47 | plus |
| Nos_Qfind_162 | GGGAGTCGTCGGGTTTATGGGTTTAATGGGAGGGTGAATGGGG | 4424245 | 4424287 | 43 | plus |
| Nos_Qfind_163 | GGGGATTAGGGATTAGGGATTGGGGATTGGGGATTGGGGATTGGGG | 4429252 | 4429297 | 46 | plus |
| Nos_Qfind_164 | GGGAGTGGGAATTGGGGATTGGGGACTAGGG | 4450180 | 4450210 | 31 | plus |
| Nos_Qfind_165 | GGGTTTAGGGAAGGGTATAGGGG | 4458149 | 4458171 | 23 | plus |
| Nos_Qfind_166 | GGGACTGGGGACTGGGGACTGGGG | 4498167 | 4498190 | 24 | plus |
| Nos_Qfind_167 | GGGGTGTGGGGGTGTGAGGGTGTAGGGGTGTGGGGGTGTGGGGGTGTAAGGG | 4557188 | 4557239 | 52 | plus |
| Nos_Qfind_168 | GGGAATGAGAGGGGTGTAGGGGTGTGGGG | 4570176 | 4570204 | 29 | plus |
| Nos_Qfind_169 | GGGGAGGGAAGCAGGGGGTAGGGAGCAGGGGAAGCAGGGGAAGCAGGGG | 4589725 | 4589773 | 49 | plus |
| Nos_Qfind_170 | GGGAGCAGGGATGCAGGGGGTGCAGGGG | 4591560 | 4591587 | 28 | plus |
| Nos_Qfind_171 | GGGTGTGTGGGTTTAGGGGTTTAGGGGG | 4593165 | 4593192 | 28 | plus |
| Nos_Qfind_172 | GGGTTTGGGCGAGGGTATAGGGGTATAGGGGTGTAGGGG | 4597168 | 4597206 | 39 | plus |
| Nos_Qfind_173 | GGGGTGTAGGGGTTTAGGGGTGTAGGGG | 4598119 | 4598146 | 28 | plus |
| Nos_Qfind_174 | GGGGGCTGGGGACTGGGTAACGGGGACTGGGGACTGGG | 4609185 | 4609222 | 38 | plus |
| Nos_Qfind_175 | GGGGTTTAGGGGTGTAAGGGTTTAGGGGTGTAGGGG | 4642749 | 4642784 | 36 | plus |
| Nos_Qfind_176 | GGGGTATAGGGGTATAGGGGTGTAGGGGTGTGGGGG | 4662517 | 4662552 | 36 | plus |
| Nos_Qfind_177 | GGGGTGTAAGGGTGTAGGGGTGTAAGGGAGCAGGGG | 4663572 | 4663607 | 36 | plus |
| Nos_Qfind_178 | GGGGTTGGGTACTGGGTACTGGGGGTTGGGTACTGGGGGTTGGGTACTGGGGGTTGGGG | 4676163 | 4676221 | 59 | plus |
| Nos_Qfind_179 | GGGGAGCAGGGAGCAGGGAGCAAGGGAAGCAGGGGGAGTAGGGGG | 4683996 | 4684040 | 45 | plus |
| Nos_Qfind_180 | GGGCTAGGGGGTAGGGAGTAGGG | 4687699 | 4687721 | 23 | plus |
| Nos_Qfind_181 | GGGAGCAGGGAGCAGGGAGCAGGGGG | 4729872 | 4729897 | 26 | plus |
| Nos_Qfind_182 | GGGGGCAGGGAGCAGGGAGCAGGGAGCAGGGGGCAGGGAGCAGGGGAGGCAGGGGAGGCAGGGGG | 4751612 | 4751676 | 65 | plus |
| Nos_Qfind_183 | GGGGTGTAAGGGTGTAGGGGTGTAAGGGTGTGAGGG | 4838945 | 4838980 | 36 | plus |
| Nos_Qfind_184 | GGGGAGTTGGGTACTGGGGATTGGGTACTGGGGATGGGGGATTGGG | 4840605 | 4840650 | 46 | plus |
| Nos_Qfind_185 | GGGATTGGGTATTGGGTATTGGGTATTGGGG | 4865626 | 4865656 | 31 | plus |
| Nos_Qfind_186 | GGGATTAGGGATTAGGGAGTAGGG | 4900992 | 4901015 | 24 | plus |
| Nos_Qfind_187 | GGGTATTGGGTATGGGGGATTAGGGATTGGG | 4950596 | 4950626 | 31 | plus |
| Nos_Qfind_188 | GGGGAATTGGGGGTTGGGAACTGGGGATTAGGG | 4990285 | 4990317 | 33 | plus |
| Nos_Qfind_189 | GGGAACTGGGGATTGGGTACTGGGG | 5041128 | 5041152 | 25 | plus |
| Nos_Qfind_190 | GGGTGTAAGGGTTTAAGGGTACAGGGGTGTAAGGG | 5055159 | 5055193 | 35 | plus |
| Nos_Qfind_191 | GGGGTGTAAGGGTGTAGGGGTGTAAGGG | 5071119 | 5071146 | 28 | plus |
| Nos_Qfind_192 | GGGTGTAAGGGGGTAAGGGTATAGGGGTGTAAGGG | 5087253 | 5087287 | 35 | plus |
| Nos_Qfind_193 | GGGACTGGGGATTGGGGATTGGGGACTGGG | 5105733 | 5105762 | 30 | plus |
| Nos_Qfind_194 | GGGGACTGGGTATTGGGGATTGGGTATTGGG | 5114064 | 5114094 | 31 | plus |
| Nos_Qfind_195 | GGGATTGGGGATTAGGGACTGGGG | 5140297 | 5140320 | 24 | plus |
| Nos_Qfind_196 | GGGATTGGGGATTGGGGACTGGGGATTGGGGACTGGGGACTGGGGATTGGGGATTGGGGACTGGGGACTGGGGACTGGG | 5219352 | 5219430 | 79 | plus |
| Nos_Qfind_197 | GGGATTGGGGACTGGGGATTGGGGACTGGGGACTAGGGATTGGGGACTAGGGATTGGGGACTGGGGATTGGGGACTGGGGATTGGGGACTGGGGACTGGGGACTAGGGATTGGGGACTGGG | 5238394 | 5238514 | 121 | plus |
| Nos_Qfind_198 | GGGGATTGGGGACTGGGGATTGGGG | 5263152 | 5263176 | 25 | plus |
| Nos_Qfind_199 | GGGATTAGGGATTGGGGATCGGGGATTGGGGACTGGG | 5263874 | 5263910 | 37 | plus |
| Nos_Qfind_200 | GGGATTGGGGATTAGGGATTGGGGATTGGGGGTTGGGGACTGGGGATTGGGGATTGGGTATTGGGGATTGGG | 5330538 | 5330609 | 72 | plus |
| Nos_Qfind_201 | GGGAAATAGGGAAAGGGCAAATAGGG | 5398090 | 5398115 | 26 | plus |
| Nos_Qfind_202 | GGGGCGTTGGGGAGTGGGGATTAGGGACTGGGG | 5418434 | 5418466 | 33 | plus |
| Nos_Qfind_203 | GGGTTTAGGGGTATAGGGGTGTAGGGG | 5428988 | 5429014 | 27 | plus |
| Nos_Qfind_204 | GGGGTGTAAGGGTGTGAGGGTGTGAGGG | 5486364 | 5486391 | 28 | plus |
| Nos_Qfind_205 | GGGTGGGGGAATGGGTAATGGGTAATGGGG | 5516202 | 5516231 | 30 | plus |
| Nos_Qfind_206 | GGGGTGTAAGGGTGTAAGGGTATAAGGGTGTAGGGGTGTAGGGGTGTAGGGGTGTAGGGG | 5519626 | 5519685 | 60 | plus |
| Nos_Qfind_207 | GGGAGCGGGTTGGGAATCAAAGGGATTGGGTTTGGCGGGG | 5529335 | 5529374 | 40 | plus |
| Nos_Qfind_208 | GGGGACTGGGAACTGGGAATTGGGGATTGGGGACTGGGGG | 5551214 | 5551253 | 40 | plus |
| Nos_Qfind_209 | GGGGGTAATGGGTAATGGGTAATGGGTAATGGG | 5639064 | 5639096 | 33 | plus |
| Nos_Qfind_210 | GGGGTGCAGGGGGCAGGGTGCAGGGGGG | 5642066 | 5642093 | 28 | plus |
| Nos_Qfind_211 | GGGGCAGGGGGCAGGGAGCAAGGGGG | 5653813 | 5653838 | 26 | plus |
| Nos_Qfind_212 | GGGTATAGGGGTGTAAGGGTGTAGGGGTGTAAGGG | 5660321 | 5660355 | 35 | plus |
| Nos_Qfind_213 | GGGGATAAGGGATTGGGTATTGGG | 5661851 | 5661874 | 24 | plus |
| Nos_Qfind_214 | GGGATTGGGTATTGGGTATTGGGGG | 5750309 | 5750333 | 25 | plus |
| Nos_Qfind_215 | GGGGACTGGGGGCTGGGGATTGGGG | 5789425 | 5789449 | 25 | plus |
| Nos_Qfind_216 | GGGGACGGGAACGGGGTATGGG | 5822552 | 5822573 | 22 | plus |
| Nos_Qfind_217 | GGGGTGTAAGGGTGTAAGGGTGTGGGGGTGTAGGGG | 5825932 | 5825967 | 36 | plus |
| Nos_Qfind_218 | GGGACTGGGTATTGGGGACTGGGGACTGGGGACTGGG | 5842288 | 5842324 | 37 | plus |
| Nos_Qfind_219 | GGGCCTCCTTGGGCAAAGGGGCGGGATAAAAGGG | 5845005 | 5845038 | 34 | plus |
| Nos_Qfind_220 | GGGTGTAGGGGTGTCAGGGTGCAGGGG | 5974145 | 5974171 | 27 | plus |
| Nos_Qfind_221 | GGGGATTGGGGATTGGGGATTGGG | 6026163 | 6026186 | 24 | plus |
| Nos_Qfind_222 | GGGGTGTAGGGGTTTAAGGGTATAAGGGTGTAGGG | 6067550 | 6067584 | 35 | plus |
| Nos_Qfind_223 | GGGTTTAGGGGTTTAAGGGTATAGGGGTGTAGGGGTTTAAGGGTTTAAGGGTTTAAGGGTTTAAGGG | 6123176 | 6123242 | 67 | plus |
| Nos_Qfind_224 | GGGTGTAGGGGTGCAGGGGTCTAAGGGTGTAGGG | 6125293 | 6125326 | 34 | plus |
| Nos_Qfind_225 | GGGGGTGTAGGGGTGTAAGGGTTTAGGGGTGTGAGGG | 6132391 | 6132427 | 37 | plus |
| Nos_Qfind_226 | GGGATGATGGGAGTGGGGAGTGGGGAGTTGGGAGTGGGG | 6133010 | 6133048 | 39 | plus |
| Nos_Qfind_227 | GGGACTGGGGACTGGGGACTGGGGGG | 6162159 | 6162184 | 26 | plus |
| Nos_Qfind_228 | GGGGTATGGGGGTATAGGGGTATAGGGGTTTGGGGGTATAGGGGTATAGGGGTTTGGG | 6170071 | 6170128 | 58 | plus |
| Nos_Qfind_229 | GGGGACTGGGGACTGGGGACTGGG | 6273635 | 6273658 | 24 | plus |
| Nos_Qfind_230 | GGGGTATAGGGGTATAGGGGGATGGGGG | 6289137 | 6289164 | 28 | plus |
| Nos_Qfind_231 | GGGGACTGGGGATTGGGAATCGGGGATTAGGG | 6294095 | 6294126 | 32 | plus |
| Nos_Qfind_232 | GGGGCTGGGGACTGGGGCCTGGGG | 6305173 | 6305196 | 24 | plus |
| Nos_Qfind_233 | GGGAATGGGGGATGGGTAATGGGGGATGGG | 6308241 | 6308270 | 30 | plus |
| Nos_Qfind_234 | GGGATTAGGGATTGGGGACTGGGGATTGGGGATAAGGGG | 6327021 | 6327059 | 39 | plus |
| Nos_Qfind_235 | GGGGGTTGGGGATTGGGGACTGGGG | 6327071 | 6327095 | 25 | plus |
| Nos_Qfind_236 | GGGATAGGTAGGGATAGGGATTGGG | 6334223 | 6334247 | 25 | plus |
| Nos_Qfind_237 | GGGTGTAAGGGTATAGGGGTGTGAGGGTGTAAGGGTGTAAGGGTGTAAGGG | 6376481 | 6376531 | 51 | plus |

S1 Table D: G4 sequences in *Nostoc* sp. PCC7120 on minus strand

Table shows the potential G4 forming sequences on the minus strand of the chromosome of *Ana* obtained from ProQuad Database (http://quadbase.igib.res.in/). The following query parameters had been used: pattern C, stem size C3-5 and loop size L1-7, genomic location: all. Sequence of the G4 pattern, position on the chromosome and total length are stated.

| **sequence name** | **sequence (5'-3')** | **start** | **end** | **length** | **strand** |
| --- | --- | --- | --- | --- | --- |
| Nos_Qfind_236 | CCCAGTCCCCGGTACCCAATCCC | 19297 | 19319 | 23 | plus |
| Nos_Qfind_237 | CCCCTGCTCCCTGCCCCCTTCCCC | 60082 | 60105 | 24 | plus |
| Nos_Qfind_238 | CCCTTATCCCCCTACACCCTTACACCC | 63476 | 63502 | 27 | plus |
| Nos_Qfind_239 | CCCCAGTCCCCAGTCCCCAATCCCCAATCCCC | 80938 | 80969 | 32 | plus |
| Nos_Qfind_240 | CCCTTGCACCCTGCTCCCTGCTCCC | 94571 | 94595 | 25 | plus |
| Nos_Qfind_241 | CCCAGTCCCTAGTCCCTATTCCCCAGTCCCC | 123706 | 123736 | 31 | plus |
| Nos_Qfind_242 | CCCTAAACCCCTAAACCCCTATACCCCTACCCCC | 129556 | 129589 | 34 | plus |
| Nos_Qfind_243 | CCCAGTCCCCAGTCCCCAGTCCC | 141723 | 141745 | 23 | plus |
| Nos_Qfind_244 | CCCCAATTCCCAATTCCCAGTCCCCAGTCCCCAGTCCCCAGTCCCCAATCCC | 149820 | 149871 | 52 | plus |
| Nos_Qfind_245 | CCCAATCCCCAGTCCCCAATCCCCAGTCCCCAATCCCCAGTCCCCAATCCCC | 169639 | 169690 | 52 | plus |
| Nos_Qfind_246 | CCCCTAAACCCCTACACCCCTACACCCCTACACCCTTAAACCC | 183244 | 183286 | 43 | plus |
| Nos_Qfind_247 | CCCATTCCCCACTCCCAACTCCCC | 203745 | 203768 | 24 | plus |
| Nos_Qfind_248 | CCCCTACACCCTTACACCCCTAAACCC | 228561 | 228587 | 27 | plus |
| Nos_Qfind_249 | CCCACCTTCCCCAGTCCCCAGTCCCCAATCCCCC | 254145 | 254178 | 34 | plus |
| Nos_Qfind_250 | CCCTCACACCCTTACACCCTTACACCCTCACACCC | 255781 | 255815 | 35 | plus |
| Nos_Qfind_251 | CCCAATCCCCAATCCCCAGTCCCCAATCCCCAGTCCCCAGTCCCC | 287777 | 287821 | 45 | plus |
| Nos_Qfind_252 | CCCTAAACCCCTACACCCTTACACCC | 290822 | 290847 | 26 | plus |
| Nos_Qfind_253 | CCCAGTTCCCCCTGCTCCCAGCTCCCTAATCCCC | 316458 | 316491 | 34 | plus |
| Nos_Qfind_254 | CCCCCTGCCCCCTGCCCCCTGCCCCCTGCCTCTTCCCTCTTCCCTCTTCCCACTCCCC | 318921 | 318946 | 26 | plus |
| Nos_Qfind_255 | CCCCAATCCCCAGTCCCCAATCCCCAGTCCCCAGTCCCCAGTCCCCAGTCCC | 353414 | 353465 | 52 | plus |
| Nos_Qfind_256 | CCCCTGCACCCTACACCCTGCACCCTGCTTTCCCTAGTCCCCAGTCCCCAGACCC | 377561 | 377585 | 25 | plus |
| Nos_Qfind_257 | CCCAGTACCCAGTCCCCAATCCCCAATCCCC | 447928 | 447958 | 31 | plus |
| Nos_Qfind_258 | CCCTTACACCCTCATACCCTTACACCCTCATACCCTTACACCCTCATACCCTTACACCCTCATACCCTTACACCCTCATACCCTTACACCCTCATACCCTTATACCCTCATACCCTTATACCCTTATACCCCTACACCC | 449427 | 449565 | 139 | plus |
| Nos_Qfind_259 | CCCCTGCCCCCTGCTCCCTGCCCCCC | 458600 | 458625 | 26 | plus |
| Nos_Qfind_260 | CCCTTACACCCCTATACCCCTACACCC | 499097 | 499123 | 27 | plus |
| Nos_Qfind_261 | CCCCAATCCCTAATCCCCAATCCCCAATCCCC | 519863 | 519894 | 32 | plus |
| Nos_Qfind_262 | CCCTACACCCCTAGACCCCCATACCCCTACACCC | 525167 | 525200 | 34 | plus |
| Nos_Qfind_263 | CCCCGTTCCCCAGTCCCCAATCCCGGTTCCCCAATCCCC | 573037 | 573075 | 39 | plus |
| Nos_Qfind_264 | CCCTAATCCCAATCCCCAATCCCTAGTACCCAATCCCTAATCCCCATTACCCCTTATCCCC | 641741 | 641801 | 61 | plus |
| Nos_Qfind_265 | CCCAGTCCCCAGTCCCCAATCCCCAATCCC | 643775 | 643804 | 30 | plus |
| Nos_Qfind_266 | CCCTTAAACCCCTATACCCTTACACCC | 659434 | 659460 | 27 | plus |
| Nos_Qfind_267 | CCCTCACACCCCTATACCCCTACACCC | 734026 | 734052 | 27 | plus |
| Nos_Qfind_268 | CCCTTACACCCTTATACCCTTACACCC | 748019 | 748045 | 27 | plus |
| Nos_Qfind_269 | CCCCTACACCCTTACACCCCCATACCC | 760729 | 760755 | 27 | plus |
| Nos_Qfind_270 | CCCCAGTCCCCAGTCCCCAGTCCCC | 848085 | 848109 | 25 | plus |
| Nos_Qfind_271 | CCCCTACACCCCTATACCCTTCTCCCAACCC | 869539 | 869569 | 31 | plus |
| Nos_Qfind_272 | CCCTCATCCCCGTCCTCCCTACCC | 874300 | 874323 | 24 | plus |
| Nos_Qfind_273 | CCCCCAATCCCCATTACCCCTTATCCCC | 909560 | 909587 | 28 | plus |
| Nos_Qfind_274 | CCCCTACACCCCCATACCCCTATACCC | 910330 | 910356 | 27 | plus |
| Nos_Qfind_275 | CCCAGTCCCCAATCCCCAGTCCCCAATCCCCAGTCCCCAATCCCCAGTCCC | 923147 | 923197 | 51 | plus |
| Nos_Qfind_276 | CCCCTACACCCCTAAACCCTTACACCCCTAAACCC | 925179 | 925213 | 35 | plus |
| Nos_Qfind_277 | CCCCTACACCCTTACACCCTTACACCC | 932280 | 932306 | 27 | plus |
| Nos_Qfind_278 | CCCAGTCCCCAGCCCCCAATCCCTAACCC | 961259 | 961287 | 29 | plus |
| Nos_Qfind_279 | CCCTCACACCCTTACACCCTTATACCCCTAAACCC | 971371 | 971405 | 35 | plus |
| Nos_Qfind_280 | CCCTTACACCCCTACCCCCTTATACCCTTATCCCC | 1018965 | 1018999 | 35 | plus |
| Nos_Qfind_281 | CCCTTATACCCCTATACCCTCATACCCTTACACCC | 1084175 | 1084209 | 35 | plus |
| Nos_Qfind_282 | CCCTTACACCCTTAAACCCTCACACCC | 1092543 | 1092569 | 27 | plus |
| Nos_Qfind_283 | CCCCAGTCCCCAGCCCCCAATCCCCAATCCC | 1102823 | 1102853 | 31 | plus |
| Nos_Qfind_284 | CCCCAATCCCCATTCCCCAACCCCCAGTCCCCAATCCCCAGTCCCCAATCCCCAATCCCCAATCCCC | 1107227 | 1107293 | 67 | plus |
| Nos_Qfind_285 | CCCCCAATCCCCAATCCCTACTCCC | 1129391 | 1129415 | 25 | plus |
| Nos_Qfind_286 | CCCCAATCCCCAGTACCCAATCCCCAGTACCCAATCCCCAATCCCCAACCCCCAATCCCCAATCCCCAACCCCC | 1213110 | 1213183 | 74 | plus |
| Nos_Qfind_287 | CCCTCACACCCTCACGCCCTCACACCC | 1214584 | 1214610 | 27 | plus |
| Nos_Qfind_288 | CCCCTACTCCCTGCCCCCTTGCCCCC | 1219519 | 1219544 | 26 | plus |
| Nos_Qfind_289 | CCCTTACACCCTCACACCCTCACACCCTCACACCCTCACACCCTCACACCC | 1230531 | 1230581 | 51 | plus |
| Nos_Qfind_290 | CCCCTGCTCCCTGCTCCCCTGCTCCCTGCTCCCTGCCCCC | 1231392 | 1231431 | 40 | plus |
| Nos_Qfind_291 | CCCCAGTACCCAATCCCCAATCCCAATCCCCAATCCCCAATCCCCAGTCCCCAGTCCCC | 1281880 | 1281938 | 59 | plus |
| Nos_Qfind_292 | CCCCAGTCCCCAGTCCCCAATCCCCAGTCCCC | 1284283 | 1284314 | 32 | plus |
| Nos_Qfind_293 | CCCTTACACCCCTAAACCCTTACACCC | 1301449 | 1301475 | 27 | plus |
| Nos_Qfind_294 | CCCTACATCCCTACATCCCTACACCCCTACACCC | 1320203 | 1320236 | 34 | plus |
| Nos_Qfind_295 | CCCTTACACCCCTATACCCTTACACCCTTACACCC | 1344206 | 1344240 | 35 | plus |
| Nos_Qfind_296 | CCCCTACACCCCCATACCCTTACACCC | 1360183 | 1360209 | 27 | plus |
| Nos_Qfind_297 | CCCAGTCCCCCATCCCCAATCCCCCCAGTCCCC | 1360405 | 1360437 | 33 | plus |
| Nos_Qfind_298 | CCCAATACCCAGTCCCCGATCCCCATACCCC | 1361693 | 1361723 | 31 | plus |
| Nos_Qfind_299 | CCCCTACACCCTTACACCCTTACACCC | 1424929 | 1424955 | 27 | plus |
| Nos_Qfind_300 | CCCATTACCCGTTACCCATTACCC | 1433873 | 1433896 | 24 | plus |
| Nos_Qfind_301 | CCCAATACCCAATACCCAATACCCAATACCCAATACCC | 1470162 | 1470199 | 38 | plus |
| Nos_Qfind_302 | CCCCTACACCCCTATACCCCTATACCC | 1479327 | 1479353 | 27 | plus |
| Nos_Qfind_303 | CCCCAGTCCCCAATCCCCAGTCCCCAGTCCCCAGTCCCCAATCCCCAACCCCC | 1494691 | 1494743 | 53 | plus |
| Nos_Qfind_304 | CCCAAGCCCCAGACCCTAATCCCCAGTCCCC | 1526794 | 1526824 | 31 | plus |
| Nos_Qfind_305 | CCCTACCCCTAAACCCCTATACCCCCATACCCC | 1551730 | 1551762 | 33 | plus |
| Nos_Qfind_306 | CCCAATCCCCAATACCCAATACCCAACACCCAATACCCAATACCCAATACCCAACACCCAATCCC | 1552282 | 1552346 | 65 | plus |
| Nos_Qfind_307 | CCCAGTACCCAGTCCCAAGTCCCC | 1563840 | 1563863 | 24 | plus |
| Nos_Qfind_308 | CCCCATCCCCAATCCCCAATCCCTCATCCCC | 1587576 | 1587606 | 31 | plus |
| Nos_Qfind_309 | CCCCTACACCCTTACACCCCTACACCCTTACACCCCTACACCC | 1588745 | 1588787 | 43 | plus |
| Nos_Qfind_310 | CCCCGATACCCAATCCCCGATACCCAATACCCAATCCCC | 1595113 | 1595151 | 39 | plus |
| Nos_Qfind_311 | CCCAATCCCCAATCCCCAATCCCC | 1627203 | 1627226 | 24 | plus |
| Nos_Qfind_312 | CCCATTACCCATTACCCATTACCC | 1695037 | 1695060 | 24 | plus |
| Nos_Qfind_313 | CCCAATACCCAATCCCCAGTCCC | 1698684 | 1698706 | 23 | plus |
| Nos_Qfind_314 | CCCCACGCCCCGCCCAGATTCCC | 1772414 | 1772436 | 23 | plus |
| Nos_Qfind_315 | CCCATCCCCCATCCCCAATCCC | 1783972 | 1783993 | 22 | plus |
| Nos_Qfind_316 | CCCCTACACCCTTACACCCTTACACCCTTACACCC | 1804509 | 1804543 | 35 | plus |
| Nos_Qfind_317 | CCCCTAAACCCCTACACCCCTATTCCC | 1805044 | 1805070 | 27 | plus |
| Nos_Qfind_318 | CCCAACCCACCCAACCC | 1860394 | 1860410 | 17 | plus |
| Nos_Qfind_319 | CCCCATTCCCCGCTACCCAATCCCCGATCCCTTTTTCCC | 1866387 | 1866425 | 39 | plus |
| Nos_Qfind_320 | CCCTACACCCTTACACCCCTAAACCCCTACACCCCTTCTCCC | 1893223 | 1893264 | 42 | plus |
| Nos_Qfind_321 | CCCAGTCCCCAATCCCTAGTCCCCAGTCCCCAGTCCC | 1893795 | 1893831 | 37 | plus |
| Nos_Qfind_322 | CCCCCAGTCCCCAGTCCCCAGTCCCC | 1997674 | 1997699 | 26 | plus |
| Nos_Qfind_323 | CCCCTATACCCTTACCCCCTTACACCC | 2007637 | 2007663 | 27 | plus |
| Nos_Qfind_324 | CCCCCATCCCCAATCCCCTCTCCC | 2018061 | 2018084 | 24 | plus |
| Nos_Qfind_325 | CCCCAATCCCCAATCCCCAATCCCTAATCCCTAATCCCCAATCCC | 2040328 | 2040372 | 45 | plus |
| Nos_Qfind_326 | CCCTTATACCCTCACACCCCTACACCC | 2049400 | 2049426 | 27 | plus |
| Nos_Qfind_327 | CCCAGTACCCAGTCCCTAATCCC | 2099828 | 2099850 | 23 | plus |
| Nos_Qfind_328 | CCCAATCCCCAATCCCCAGTCCC | 2111754 | 2111776 | 23 | plus |
| Nos_Qfind_329 | CCCTTCCCCCAATCCCCAATCCCCAGTCCCCAATCCCC | 2191248 | 2191285 | 38 | plus |
| Nos_Qfind_330 | CCCCTACACCCCTACACCCTCATACCCCTACACCC | 2238714 | 2238748 | 35 | plus |
| Nos_Qfind_331 | CCCTCACACCCTTACACCCTTACACCCACATACCCCTATACCCCTATACCC | 2259396 | 2259446 | 51 | plus |
| Nos_Qfind_332 | CCCAGTACCCAATCCCCAATCCCCAGTACCC | 2267309 | 2267339 | 31 | plus |
| Nos_Qfind_333 | CCCCAATCCCCAATCCCCAGTCCCCAATCCCCAGTCCCCAGTCCCCAATCCCCAATCCCCAGTCCCCAATCCCC | 2270479 | 2270552 | 74 | plus |
| Nos_Qfind_334 | CCCCTCCCCCAACCCCTCCCC | 2272267 | 2272287 | 21 | plus |
| Nos_Qfind_335 | CCCCTCCCCCAACCCCTCCCC | 2273084 | 2273104 | 21 | plus |
| Nos_Qfind_336 | CCCCCAATCCCCAGTCCCCAGTCCCCAATCCCCAGTCCC | 2303740 | 2303778 | 39 | plus |
| Nos_Qfind_337 | CCCTTACACCCTTACACCCTTACACCCTCACACCC | 2310513 | 2310547 | 35 | plus |
| Nos_Qfind_338 | CCCCAGTCCCTAATCCCTAGTCCCCAACCCCTACTCCC | 2393899 | 2393936 | 38 | plus |
| Nos_Qfind_339 | CCCAGTCCCCAATCCCCCATCCCC | 2394930 | 2394953 | 24 | plus |
| Nos_Qfind_340 | CCCCAGTCCCCAGTCCCCAATCCCCAATACCCAATCCCTACCCC | 2401429 | 2401472 | 44 | plus |
| Nos_Qfind_341 | CCCTTGCTCCCTGCTCCCTGCCCCCC | 2521395 | 2521420 | 26 | plus |
| Nos_Qfind_342 | CCCTTACACCCCTACACCCTTACACCC | 2529055 | 2529081 | 27 | plus |
| Nos_Qfind_343 | CCCCTGCACCCCTACACCCCTGCACCCCTACACCCCCATACCCC | 2666584 | 2666627 | 44 | plus |
| Nos_Qfind_344 | CCCCTTCCCCAGTCCCCAATCCCCAATCCCTAATCCCCAATCCCTAATCCCCAATCCCTAATCCCCAATCCCTAATCCC | 2736492 | 2736570 | 79 | plus |
| Nos_Qfind_345 | CCCATTACCCATTCCCCATTCCC | 2838300 | 2838322 | 23 | plus |
| Nos_Qfind_346 | CCCCAACCCCCAACCCCCAATCCCCAACCCTCAATCCCCAATCCCCAATCCCC | 2855541 | 2855593 | 53 | plus |
| Nos_Qfind_347 | CCCTTACACCCTCACACCCTTACACCCTCACACCCTTACACCCTCATACCCTTACACCCTTACACCCTCACACCCTTACACCCTTACACCCTCACACCCTCACACCC | 2858036 | 2858142 | 107 | plus |
| Nos_Qfind_348 | CCCTAATCCCCATTCCCTAGTCCCC | 2901420 | 2901444 | 25 | plus |
| Nos_Qfind_349 | CCCTTACACCCTTACACCCCTACACCC | 2928815 | 2928841 | 27 | plus |
| Nos_Qfind_350 | CCCCAATCCCCAATCCCCAATCCC | 3048712 | 3048735 | 24 | plus |
| Nos_Qfind_351 | CCCAGTCCCTCATCCCTCCC | 3084228 | 3084247 | 20 | plus |
| Nos_Qfind_352 | CCCAATCCCCAGTCCCCAATACCCAGTCCC | 3125729 | 3125758 | 30 | plus |
| Nos_Qfind_353 | CCCTATTCCCCGTTCCCTATTCCCGGTTCCC | 3140723 | 3140753 | 31 | plus |
| Nos_Qfind_354 | CCCCTCCCCAACCCTCCCC | 3158375 | 3158393 | 19 | plus |
| Nos_Qfind_355 | CCCAGTCCCCAGTCCCCAGTCCCCAGTCCCCAGTCCC | 3160830 | 3160866 | 37 | plus |
| Nos_Qfind_356 | CCCCAGTCCCCAGTCCCCAGTCCCCAGTCCCCAGTCCCCC | 3164289 | 3164328 | 40 | plus |
| Nos_Qfind_357 | CCCAGTCCCCAGTCCCCAGTCCCCAGTCCCC | 3192231 | 3192261 | 31 | plus |
| Nos_Qfind_358 | CCCAACCTCCCACCCTTCCC | 3257164 | 3257183 | 20 | plus |
| Nos_Qfind_359 | CCCCACCCCAACCCTCCCC | 3264721 | 3264739 | 19 | plus |
| Nos_Qfind_360 | CCCTTACACCCTTACACCCATATACCCCTATACCCTTACACCC | 3345327 | 3345369 | 43 | plus |
| Nos_Qfind_361 | CCCAATCCCCATTACCCCTTATCCCC | 3365417 | 3365442 | 26 | plus |
| Nos_Qfind_362 | CCCACCTTCCCCAGTCCCCAGTCCC | 3365452 | 3365476 | 25 | plus |
| Nos_Qfind_363 | CCCCAGTCCCCAGTCCCCAGTCCCCAGTCCCC | 3410722 | 3410753 | 32 | plus |
| Nos_Qfind_364 | CCCCAGTCCCCAGTCCCCAATCCCCAATCCCCAATCCCC | 3422100 | 3422138 | 39 | plus |
| Nos_Qfind_365 | CCCCAATCCCCAATCCCCAATCCCCAGTCCCCAATCCCCAATCCCCAGTCCCTAGTCCC | 3422767 | 3422825 | 59 | plus |
| Nos_Qfind_366 | CCCTTACACCCTCACACCCTTACACCC | 3437439 | 3437465 | 27 | plus |
| Nos_Qfind_367 | CCCCAATCCCCAATCCCCAATCCCCAATCCCCAGTCCCCAATCCCCAATCCC | 3456196 | 3456247 | 52 | plus |
| Nos_Qfind_368 | CCCCTAAACCCCTACACCCTTATACCCCTAAACCCCTACACCC | 3459356 | 3459398 | 43 | plus |
| Nos_Qfind_369 | CCCAGTCCCTAGTCCCCAGTCCCC | 3466990 | 3467013 | 24 | plus |
| Nos_Qfind_370 | CCCCTATACCCCTATACCCCTATACCCCTATACCCCTATACCCCTATACCC | 3471872 | 3471922 | 51 | plus |
| Nos_Qfind_371 | CCCTAGCACCCCTACCCCCTCCTCCC | 3562099 | 3562124 | 26 | plus |
| Nos_Qfind_372 | CCCAGTCCCCAGTCCCCAGTCCCC | 3592447 | 3592470 | 24 | plus |
| Nos_Qfind_373 | CCCACTCCCCAGTCCCTAGTCCCCAGTCCCC | 3610941 | 3610971 | 31 | plus |
| Nos_Qfind_374 | CCCGACATCCCGCCAGCCCACCCCC | 3614581 | 3614605 | 25 | plus |
| Nos_Qfind_375 | CCCCAATCCCCAATTCCCAATCCCC | 3619406 | 3619430 | 25 | plus |
| Nos_Qfind_376 | CCCTAATCCCAGTCCCCAGTCCCC | 3620096 | 3620119 | 24 | plus |
| Nos_Qfind_377 | CCCATCCCCGATACCCAGTCCC | 3621664 | 3621685 | 22 | plus |
| Nos_Qfind_378 | CCCCAATTCCCAATCCCCAATCCC | 3638023 | 3638046 | 24 | plus |
| Nos_Qfind_379 | CCCCCACACCCCCACACCCCCACACCCCCACACCCCTAAACCCTTACACCC | 3653641 | 3653691 | 51 | plus |
| Nos_Qfind_380 | CCCCTACACCCCCACACCCTTACACCCCTATACCC | 3668113 | 3668147 | 35 | plus |
| Nos_Qfind_381 | CCCCAGTCCCCAGTCCCCAATCCCCAACCC | 3693064 | 3693093 | 30 | plus |
| Nos_Qfind_382 | CCCCAATCCCCAGTCCCCAATCCCCAATCCCC | 3702770 | 3702801 | 32 | plus |
| Nos_Qfind_383 | CCCCAATCCCTAATCCCCAATCCCC | 3726368 | 3726392 | 25 | plus |
| Nos_Qfind_384 | CCCAGTCCCCAGCCCCCAATCCCAAGTCCCC | 3733285 | 3733315 | 31 | plus |
| Nos_Qfind_385 | CCCTCTCCCCATCTCCCTGCCCCCTGCCCC | 3754557 | 3754586 | 30 | plus |
| Nos_Qfind_386 | CCCCCAGTCCCCAATCCCTAGACCCTAATCCC | 3785040 | 3785071 | 32 | plus |
| Nos_Qfind_387 | CCCAGTCCCCAGTCCCCAGTCCC | 3853322 | 3853344 | 23 | plus |
| Nos_Qfind_388 | CCCTTTTCCCCCTGCACCCTGCCCCTCTGCCCATTACCCATTCCCCATTCCCC | 3868763 | 3868815 | 53 | plus |
| Nos_Qfind_389 | CCCCGTCCCCAGTCCCCAGTCCCCAATTCCC | 3900877 | 3900907 | 31 | plus |
| Nos_Qfind_390 | CCCCCACACCCCTATACCCTTATACCC | 3907718 | 3907744 | 27 | plus |
| Nos_Qfind_391 | CCCAATCCCCAGTCCCCAGTCCC | 3935504 | 3935526 | 23 | plus |
| Nos_Qfind_392 | CCCTTACACCCTTACACCCCTACACCC | 4017082 | 4017108 | 27 | plus |
| Nos_Qfind_393 | CCCTCACACCCTTATACCCTTACACCCTCACACCC | 4036962 | 4036996 | 35 | plus |
| Nos_Qfind_394 | CCCTCACACCCCTAAACCCTCACACCC | 4110122 | 4110148 | 27 | plus |
| Nos_Qfind_395 | CCCAGTCCCCAGTCCCCAGTCCCTAATTCCC | 4114258 | 4114288 | 31 | plus |
| Nos_Qfind_396 | CCCTGCTTCCCCTGCTCCCTTGCCCC | 4136984 | 4137009 | 26 | plus |
| Nos_Qfind_397 | CCCAGTCCCCAGTCCCCAGTCCCC | 4235532 | 4235555 | 24 | plus |
| Nos_Qfind_398 | CCCCAGTCCCCAGTCCCCAGTCCC | 4235594 | 4235617 | 24 | plus |
| Nos_Qfind_399 | CCCATTACCCATTACCCATTGCCC | 4244828 | 4244851 | 24 | plus |
| Nos_Qfind_400 | CCCAATCCCCAATCCCCAATCCCCAATCCCCAATCCCCAATCCCCAATCCCCAATCCCAATCCCCAATCCCCAATCCCCAGTCCCCAATCCC | 4247409 | 4247500 | 92 | plus |
| Nos_Qfind_401 | CCCCAGTCCCCAGTCCCCAGTCCCCAGTCCCTAATCCCCAGTCCC | 4262898 | 4262942 | 45 | plus |
| Nos_Qfind_402 | CCCTATACCCCTAAACCCCTAAACCCCTACACCC | 4265390 | 4265423 | 34 | plus |
| Nos_Qfind_403 | CCCCAATACCCAATACCCAATACCCAATACCCAATCCCCGATCCCCAGTACCCGATCCCCAATCCCC | 4282716 | 4282782 | 67 | plus |
| Nos_Qfind_404 | CCCTTACACCCCTGTACCCTTAAACCCTTACACCC | 4294397 | 4294431 | 35 | plus |
| Nos_Qfind_405 | CCCAATCCCCAATCCCCAATCCCCAGTCCCCAATCCCTACTCCCC | 4312049 | 4312093 | 45 | plus |
| Nos_Qfind_406 | CCCCTATACCCCATACCCCTATACCCC | 4357542 | 4357568 | 27 | plus |
| Nos_Qfind_407 | CCCTCACACCCCTACACCCTTACACCC | 4358366 | 4358392 | 27 | plus |
| Nos_Qfind_408 | CCCTTACACCCCTATACCCTTACCCCCTTATACCC | 4459554 | 4459588 | 35 | plus |
| Nos_Qfind_409 | CCCCTACACCCCTACACCCCTACACCCCTATACCCCTATCCCCC | 4557551 | 4557594 | 44 | plus |
| Nos_Qfind_410 | CCCATTACCCATTACCCATCTTCCC | 4578091 | 4578115 | 25 | plus |
| Nos_Qfind_411 | CCCTTACACCCTTACACCCTCACACCCTCACACCC | 4593208 | 4593242 | 35 | plus |
| Nos_Qfind_412 | CCCTCACACCCCCAAACCCCTACACCC | 4597330 | 4597356 | 27 | plus |
| Nos_Qfind_413 | CCCTTACACCCTCATACCCCTACACCCCCACACCC | 4662665 | 4662699 | 35 | plus |
| Nos_Qfind_414 | CCCCTACACCCTTACACCCTTACACCC | 4667057 | 4667083 | 27 | plus |
| Nos_Qfind_415 | CCCAGTCCCTATTCCCCAATCCC | 4676277 | 4676299 | 23 | plus |
| Nos_Qfind_416 | CCCATTACCCATCACCCATTACCC | 4686473 | 4686496 | 24 | plus |
| Nos_Qfind_417 | CCCAATCCCCAGCCCCCAGTCCCCCAATCCCCAGTCCCC | 4687775 | 4687813 | 39 | plus |
| Nos_Qfind_418 | CCCCCTTCCCCCTGCCCCCTGCCCCC | 4699832 | 4699857 | 26 | plus |
| Nos_Qfind_419 | CCCAGTCCCCAGTCCCCAATCCC | 4729920 | 4729942 | 23 | plus |
| Nos_Qfind_420 | CCCAGTCCCCAATCCCCAGTCCC | 4729964 | 4729986 | 23 | plus |
| Nos_Qfind_421 | CCCAATCCCCAATCCCCAATCCCCAATCCCCAATCCCCAGTCCCCCATCCCCC | 4751699 | 4751751 | 53 | plus |
| Nos_Qfind_422 | CCCCTATACCCTTACCCCCTTACACCC | 4766592 | 4766618 | 27 | plus |
| Nos_Qfind_423 | CCCCTATACCCTTACCCCCTTACACCC | 4803659 | 4803685 | 27 | plus |
| Nos_Qfind_424 | CCCCTACACCCTTACACCCTTACACCC | 4812407 | 4812433 | 27 | plus |
| Nos_Qfind_425 | CCCCTATACCCTTACACCCTCACACCC | 4820475 | 4820501 | 27 | plus |
| Nos_Qfind_426 | CCCAACTCCCCAACGCCCCACACCCCACACCCCACACCCC | 4824141 | 4824180 | 40 | plus |
| Nos_Qfind_427 | CCCCTACACCCCTCTACCCCTACACCC | 4827104 | 4827130 | 27 | plus |
| Nos_Qfind_428 | CCCCAGTCCCCATTCCCCATTCCC | 4839017 | 4839040 | 24 | plus |
| Nos_Qfind_429 | CCCCTGCTCCCTACTCCCTGCCCCCC | 4840669 | 4840694 | 26 | plus |
| Nos_Qfind_430 | CCCTTACACCCAGTCCCCAATCCCCAGTCCC | 4859688 | 4859718 | 31 | plus |
| Nos_Qfind_431 | CCCCAGTCCCCAACCCCTAGTCCCCAGTCCCC | 4950636 | 4950667 | 32 | plus |
| Nos_Qfind_432 | CCCCTACACCCTTACACCCCTAAACCCCCACACCCTTACACCC | 4952867 | 4952909 | 43 | plus |
| Nos_Qfind_433 | CCCCTGCACCCTGCACCCTGCCCCCC | 4989728 | 4989753 | 26 | plus |
| Nos_Qfind_434 | CCCAATCCCCAGTCCCCAGTACCCAATCCCC | 5036064 | 5036094 | 31 | plus |
| Nos_Qfind_435 | CCCAATCCCCAGTCCCCAATCCCCAATCCCCAGTCCC | 5083820 | 5083856 | 37 | plus |
| Nos_Qfind_436 | CCCAGTCCCCAATCCCCAATCCCCAATCCCCAATCCCCAATCCCC | 5117131 | 5117175 | 45 | plus |
| Nos_Qfind_437 | CCCTTATACCCCTATACCCCTATACCC | 5146328 | 5146354 | 27 | plus |
| Nos_Qfind_438 | CCCTTACACCCCTATACCCTTACACCC | 5214792 | 5214818 | 27 | plus |
| Nos_Qfind_439 | CCCCCGCTCCCCTGCTCCCTGCTCCCTGCTCCCCTGCTCCCTGTTCCCTGCTCCCTGCTCCCCTGCTCCC | 5238546 | 5238615 | 70 | plus |
| Nos_Qfind_440 | CCCTGCCCCTGCTCCCTGCCCCC | 5263207 | 5263229 | 23 | plus |
| Nos_Qfind_441 | CCCCAATCCCTAATACCCAGTACCC | 5263975 | 5263999 | 25 | plus |
| Nos_Qfind_442 | CCCCTATACCCCTATACCCCTATACCCCTACACCC | 5265033 | 5265067 | 35 | plus |
| Nos_Qfind_443 | CCCCTACACCCCTACACCCTTAAACCCCTACACCC | 5282095 | 5282129 | 35 | plus |
| Nos_Qfind_444 | CCCCAATCCCCAGTCCCCAATCCCCAGTCCCC | 5431003 | 5431034 | 32 | plus |
| Nos_Qfind_445 | CCCAATACCCAATCCCTAGTCCCCAATCCCC | 5435296 | 5435326 | 31 | plus |
| Nos_Qfind_446 | CCCCTACACCCCTATACCCCTATACCC | 5439655 | 5439681 | 27 | plus |
| Nos_Qfind_447 | CCCCAGTACCCAATCCCCAACCCCCAGTACCCAATCCCCAATCCCCAGTACCC | 5479118 | 5479170 | 53 | plus |
| Nos_Qfind_448 | CCCCAGTCCCCAGTCCCCAGTCCCCAGTCCCC | 5514050 | 5514081 | 32 | plus |
| Nos_Qfind_449 | CCCAGCACCCCCACAACCCCAACCCCACCCC | 5553025 | 5553055 | 31 | plus |
| Nos_Qfind_450 | CCCATACACCCATACACCCCTAAACCCTTACACCC | 5566343 | 5566377 | 35 | plus |
| Nos_Qfind_451 | CCCCATTCCCCATTCCCCATTCCCCATTCCCCATTCCCC | 5604942 | 5604980 | 39 | plus |
| Nos_Qfind_452 | CCCAATCCCCAATCCCCAGTCCCCAATCCCCAGTCCCCAATCCCCAGTCCCCAATCCCCAATCCCCAATCCCC | 5658729 | 5658801 | 73 | plus |
| Nos_Qfind_453 | CCCCCTGCCCCCTGCCCCCTGCCCAATCCCAAATCCCCAATCCCCAATCCCC | 5660368 | 5660419 | 52 | plus |
| Nos_Qfind_454 | CCCCAGTTCCCCATCCCAACTCCCC | 5697088 | 5697112 | 25 | plus |
| Nos_Qfind_455 | CCCCTACACCCCTACACCCCTACACCCCTATCCCCC | 5704053 | 5704088 | 36 | plus |
| Nos_Qfind_456 | CCCTTACACCCCTGTACCCGGAAACCCTTACACCC | 5704700 | 5704734 | 35 | plus |
| Nos_Qfind_457 | CCCTTTTCCCCCTGCACCCTGCCCCTCTGCCC | 5724937 | 5724968 | 32 | plus |
| Nos_Qfind_458 | CCCATTACCCATTACCCATTACCC | 5745426 | 5745449 | 24 | plus |
| Nos_Qfind_459 | CCCTTACACCCTTAAACCCTTACACCC | 5776640 | 5776666 | 27 | plus |
| Nos_Qfind_460 | CCCCAGTCCCCAGTCCCCAGTCCCCAATCCCCAATCCCCAGTCCCCAATCCCC | 5825998 | 5826050 | 53 | plus |
| Nos_Qfind_461 | CCCCATTCCCAATCCCCAATACCCAATACCC | 5829782 | 5829812 | 31 | plus |
| Nos_Qfind_462 | CCCTTACACCCCTATACCCTTTTCCCC | 5883077 | 5883103 | 27 | plus |
| Nos_Qfind_463 | CCCTGCTTCCCAGTCCCCAATCCCCAGTCCC | 5885357 | 5885387 | 31 | plus |
| Nos_Qfind_464 | CCCATTACCCATTACCCATTACCCATTACCC | 5959844 | 5959874 | 31 | plus |
| Nos_Qfind_465 | CCCCAGTCCCTAATCCCCAATCCCCAGTCCCCAATCCCCAGTCCCCAATCCCCAATCCCC | 5995480 | 5995539 | 60 | plus |
| Nos_Qfind_466 | CCCTTACACCCCTACACCCCCATACCCCTACACCC | 6026206 | 6026240 | 35 | plus |
| Nos_Qfind_467 | CCCCTACACCCCTACACCCCCATACCCCTACACCCCCATACCCCTACACCCCCATACCCCTACACCCCCATACCCC | 6123342 | 6123417 | 76 | plus |
| Nos_Qfind_468 | CCCCCAGTCCCCAATCCCCAGTCCCCAATCCC | 6134786 | 6134817 | 32 | plus |
| Nos_Qfind_469 | CCCAGTACCCAATACCCAATACCC | 6267935 | 6267958 | 24 | plus |
| Nos_Qfind_470 | CCCCTGCTCCCTGCTCCCTTGCTCCCCTGCTCCC | 6327105 | 6327138 | 34 | plus |
| Nos_Qfind_471 | CCCCTATACCCCTACACCCCTACACCCACCCC | 6393111 | 6393142 | 32 | plus |
