# Supplementary material for: Investigation of a Quadruplex-Forming Repeat Sequence Highly Enriched in Xanthomonas and Nostoc sp
Source: PLoS One. 2015 Dec 22;10(12):e0144275. doi: 10.1371/journal.pone.0144275 (PMC4692102; doi:10.1371/journal.pone.0144275)
Supplement: S2 File — GGGAATC Repeats in Xanthomonas campestris pv. campestris ATCC 33913 (Table A). GGGAATC Repeats in Xanthomonas axonopodis pv. ctri str. 306 (Table B). GGGGA(C/T)T Repeats in Nostoc sp. PCC7120 (Table C). (DOCX) [file pone.0144275.s002.docx]

**Supporting Information 2:**

**Contents:**

S2 Table A: GGGAATC Repeats in *Xanthomonas campestris pv. campestris* ATCC 33913

S2 Table B: GGGAATC Repeats in *Xanthomonas axonopodis pv. citri* str. 306

S2 Table C: GGGGA(C/T)T Repeats in *Nostoc* sp. PCC7120

S2 Table A: GGGAATC Repeats in Xanthomonas campestris pv. campestris ATCC 33913

Table shows the occurrence of GGGAATC repeat sequences in the Xcc ATCC 33913 genome (NC_003902). Sequence of the repeat, its total length and the number of repeat units are stated. Mutations deviating from the consensus sequences are marked in red. G-tracts are underscored. The location on plus or minus strand as well as start and end point of the sequence on the genome is given. Participation in inverted repeat (inv rep) formation is stated with the number of the partnering repeat and the distance between the two repeats (dis inv reps). Other indicates occurrence of another G-rich sequence or another repeat type in place of the partnering GGGAATC repeat. The next upstream and downstream genes are listed, including locus tags, location on the plus or minus strand of the genome and the distance between the neighboring gene and the repeat (dis [nt]). Distances are negative in case a repeat overlaps with the ORF. [n.a. = not available]

| **#** | **sequence (5’-3’)** | **strand** | **length [nt]** | **number units** | **start** | **end** | **inv repwith** | **dis inv reps** | **upstream gene** | **locus tag** | **strand** | **dis [nt]** | **downstream gene** | **locus tag** | **strand** | **dis [nt]** |
| --- | --- | --- | --- | --- | --- | --- | --- | --- | --- | --- | --- | --- | --- | --- | --- | --- |
| 1 | GGGAATCGGGAGTGGGGAATCGGGATTC | plus | 28 | 4 | 18080 | 18107 | 2 | 42 | conserved | [XCC0015](http://www.genome.jp/dbget-bin/www_bget?xcc:XCC0015) | plus | 3 | conserved, minus | [XCC0016](http://www.genome.jp/dbget-bin/www_bget?xcc:XCC0016) | minus | 62 |
| 2 | GGGAATAGGGAATCGGCAATGGAGAATC | minus | 28 | 4 | 18177 | 18150 | 1 | 42 | conserved | [XCC0016](http://www.genome.jp/dbget-bin/www_bget?xcc:XCC0016) | minus | -8 | conserved, plus | [XCC0015](http://www.genome.jp/dbget-bin/www_bget?xcc:XCC0015) | plus | 74 |
| 3 | GGGAATCGGGAATGGGGAATGGGGAATC | plus | 28 | 4 | 39491 | 39518 | 4 | 50 | gltD | [XCC0031](http://www.genome.jp/dbget-bin/www_bget?xcc:XCC0031) | minus | 7 | gltB, minus strand | [XCC0032](http://www.genome.jp/dbget-bin/www_bget?xcc:XCC0032) | minus | 86 |
| 4 | GGGAATCGGGAATAGGGACTCGGGAATT | minus | 28 | 4 | 39596 | 39569 | 3 | 50 | gltB | [XCC0032](http://www.genome.jp/dbget-bin/www_bget?xcc:XCC0032) | minus | 8 | gltD, minus strand | [XCC0031](http://www.genome.jp/dbget-bin/www_bget?xcc:XCC0031) | minus | 85 |
| 5 | GGGAATCGGGAGTGGGGATTCGGGAATC | minus | 28 | 4 | 110185 | 110158 | no | n.a. | tldD | [XCC0093](http://www.genome.jp/dbget-bin/www_bget?xcc:XCC0093) | minus | 9 | conserved, minus | [XCC0092](http://www.genome.jp/dbget-bin/www_bget?xcc:XCC0092) | minus | 97 |
| 6 | GGGGATTGGGGATTGGGGATTGGGAATCGGGAATCGGGAATCGGGAATCGGGAATCGGGAATCGGGAATCGGGAATC | minus | 77 | 11 | 131297 | 131221 | no | n.a. | lctD | [XCC0106](http://www.genome.jp/dbget-bin/www_bget?xcc:XCC0106) | minus | 11 | ATP, plus | [XCC0105](http://www.genome.jp/dbget-bin/www_bget?xcc:XCC0105) | plus | 1218 |
| 7 | GTGAATCGGGAGTGGGGAATC | minus | 21 | 3 | 146039 | 146019 | no | n.a. | kdgK | [XCC0118](http://www.genome.jp/dbget-bin/www_bget?xcc:XCC0118) | minus | -4 | conserved | [XCC0117](http://www.genome.jp/dbget-bin/www_bget?xcc:XCC0117) | minus | 138 |
| 8 | GGGAATCGGGAATCGGGAATCGGGAATCGGGAATCGGGAATCGGGAATCGGGAATCGGGAATCGGGAATCGGGAATCGGGAATCGGGAATCGGGAATC | minus | 98 | 14 | 232541 | 232444 | no | n.a. | conserved | [XCC0178](http://www.genome.jp/dbget-bin/www_bget?xcc:XCC0178) | plus | 11 | cls | [XCC0177](http://www.genome.jp/dbget-bin/www_bget?xcc:XCC0177) | plus | 182 |
| 9 | GGGATTGGGGAATCGGGATTGGGGATTC | minus | 28 | 4 | 284346 | 284319 | no | n.a. | conserved | [XCC0226](http://www.genome.jp/dbget-bin/www_bget?xcc:XCC0226) | minus | 6 | pseudouridylate | [XCC0225](http://www.genome.jp/dbget-bin/www_bget?xcc:XCC0225) | plus | 791 |
| 10 | GGGCGGTGGCGCGAAGGGGAATC | minus | 23 | 3 | 499593 | 499571 | no | n.a. | intragenic: glgY | [XCC0412](http://www.genome.jp/dbget-bin/www_bget?xcc:XCC0412) | plus |  | malQ | [XCC0411](http://www.genome.jp/dbget-bin/www_bget?xcc:XCC0411) | plus | -2 |
| 11 | GGGAATCGGGTCGG | plus | 14 | 2 | 552617 | 552630 | no | n.a. | intragenic: purC | [XCC0453](http://www.genome.jp/dbget-bin/www_bget?xcc:XCC0453) | minus |  |  |  |  |  |
| 12 | GGGAATGGGGAATGGAGAATCGGGAATCGGGAATCGGGATTC | plus | 42 | 6 | 557497 | 557538 | no | n.a. | conserved | [XCC0458](http://www.genome.jp/dbget-bin/www_bget?xcc:XCC0458) | plus | 4 | trpE | [XCC0459](http://www.genome.jp/dbget-bin/www_bget?xcc:XCC0459) | plus | 436 |
| 13 | GGGAATCGGGAATC | minus | 14 | 2 | 614863 | 614850 | no | n.a. | fis | [XCC0508](http://www.genome.jp/dbget-bin/www_bget?xcc:XCC0508) | minus | 4 | ice | [XCC0507](http://www.genome.jp/dbget-bin/www_bget?xcc:XCC0507) | plus | 396 |
| 14 | GGGATTCGGGATTCGGGATTCGGGATTCGGGATTCGGGAATTGGGAATTGGGAATTGGGAATTGGGAATCGGGAGTCGGGAATCGGGAATCGGGAGTCGGGAGTCGGGAATCGGGAGTCGGGAGTCGGGAGTCGGGAATCGGGAATCGGGAATCGGGAGTCGGCGATTGGGGATTTGGGATTC | minus | 183 | 26 | 619156 | 618974 | no | n.a. | conserved | [XCC0513](http://www.genome.jp/dbget-bin/www_bget?xcc:XCC0513) | plus | 103 | prmA | [XCC0512](http://www.genome.jp/dbget-bin/www_bget?xcc:XCC0512) | minus | 200 |
| 15 | GGGAATCGGGAGTGGAGAATCGGGAATC | minus | 28 | 4 | 622983 | 622956 | no | n.a. | bccP | [XCC0517](http://www.genome.jp/dbget-bin/www_bget?xcc:XCC0517) | minus | 6 | conserved | [XCC0516](http://www.genome.jp/dbget-bin/www_bget?xcc:XCC0516) | overlap with start codon, -17 | -17 |
| 16 | GGGAATCGGGAGTAGGGAATCGGGAATC | plus | 28 | 4 | 630440 | 630467 | 17 | 60 | groES | [XCC0522](http://www.genome.jp/dbget-bin/www_bget?xcc:XCC0522) | plus | 16 | groEL | [XCC0523](http://www.genome.jp/dbget-bin/www_bget?xcc:XCC0523) | plus | 101 |
| 17 | GGAAATTGGGAATGGGGAATGGGGAATC | minus | 28 | 4 | 630555 | 630528 | 16 | 60 | groEL | [XCC0523](http://www.genome.jp/dbget-bin/www_bget?xcc:XCC0523) | plus | 13 | groES | [XCC0522](http://www.genome.jp/dbget-bin/www_bget?xcc:XCC0522) | plus | 104 |
| 18 | GGGAATCGGGAATAGAGAATCGGGAATC | plus | 28 | 4 | 668493 | 668520 | 19 | 36 | atpA | [XCC0552](http://www.genome.jp/dbget-bin/www_bget?xcc:XCC0552) | plus | 10 | atpG | [XCC0553](http://www.genome.jp/dbget-bin/www_bget?xcc:XCC0553) | plus | 74 |
| 19 | GGGAATGGGGAATC | minus | 14 | 2 | 668570 | 668557 | 18 | 36 | atpG | [XCC0553](http://www.genome.jp/dbget-bin/www_bget?xcc:XCC0553) | plus | 24 | atpA | [XCC0552](http://www.genome.jp/dbget-bin/www_bget?xcc:XCC0552) | plus | 74 |
| 20 | GGGAAACGGGAATCGGGAATCGGGAATC | plus | 28 | 4 | 671018 | 671045 | 21 | 67 | atpD | [XCC0554](http://www.genome.jp/dbget-bin/www_bget?xcc:XCC0554) | plus | 30 | atpC | [XCC0555](http://www.genome.jp/dbget-bin/www_bget?xcc:XCC0555) | plus | 115 |
| 21 | GGGAATCGGGAATCGGGAGTGGGGAATC | minus | 28 | 4 | 671140 | 671113 | 20 | 67 | atpC | [XCC0555](http://www.genome.jp/dbget-bin/www_bget?xcc:XCC0555) | plus | 20 | atpD | [XCC0554](http://www.genome.jp/dbget-bin/www_bget?xcc:XCC0554) | plus | 30 |
| 22 | GGGAATCGGGCGAG | plus | 14 | 2 | 699304 | 699317 | no | n.a. | intragenic: conserved | [XCC0579](http://www.genome.jp/dbget-bin/www_bget?xcc:XCC0579) | minus |  |  |  |  |  |
| 23 | GGGTAGAGGGAATC | plus | 14 | 2 | 726307 | 726320 | no | n.a. | wxcB | [XCC0602](http://www.genome.jp/dbget-bin/www_bget?xcc:XCC0602) | plus | 3 | wxcC | [XCC0603](http://www.genome.jp/dbget-bin/www_bget?xcc:XCC0603) | plus | 17 |
| 24 | GGGAATCGGGAGAGGAGAGTTGGGAATG | plus | 28 | 4 | 777027 | 777054 | 25 | 53 | holC | [XCC0648](http://www.genome.jp/dbget-bin/www_bget?xcc:XCC0648) | minus | 7 | pepA | [XCC0649](http://www.genome.jp/dbget-bin/www_bget?xcc:XCC0649) | minus | 79 |
| 25 | GGGAATTGAAAATCGGGAATAGGGAATCGTGAAAG | minus | 35 | 5 | 777142 | 777108 | 24 | 53 | pepA | [XCC0649](http://www.genome.jp/dbget-bin/www_bget?xcc:XCC0649) | minus | -9 | holC | [XCC0648](http://www.genome.jp/dbget-bin/www_bget?xcc:XCC0648) | minus | 88 |
| 26 | GCGAATCGGGAATCGGGAATCGGGAATC | plus | 28 | 4 | 786134 | 786107 | no |  | intragenic: purL | [XCC0656](http://www.genome.jp/dbget-bin/www_bget?xcc:XCC0656) | plus |  |  |  |  |  |
| 27 | GGGAATGGAGAATCGGGAATCGGGAATGGTGAAAC | plus | 35 | 5 | 786782 | 786816 | 28 | 21 | intragenic: purL | [XCC0656](http://www.genome.jp/dbget-bin/www_bget?xcc:XCC0656) | plus |  |  |  |  |  |
| 28 | GGGAATCGGGAGTCGGGAATCGGGAATG | minus | 28 | 4 | 786865 | 786838 | 27 | 21 | intragenic: purL | [XCC0656](http://www.genome.jp/dbget-bin/www_bget?xcc:XCC0656) | plus |  |  |  |  |  |
| 29 | GGGAGGCCGGGAATTGGGAATTGGGAATCGGGAATGGGGTGAG | minus | 43 | 6 | 801059 | 801017 | no | n.a. | xpsF | [XCC0661](http://www.genome.jp/dbget-bin/www_bget?xcc:XCC0661) | plus | 6 | xpsE | [XCC0660](http://www.genome.jp/dbget-bin/www_bget?xcc:XCC0660) | plus | 166 |
| 30 | GGGAATCGGGAATCGGGAATC | minus | 21 | 3 | 811710 | 811690 | no | n.a. | transcriptional regulator | [XCC0672](http://www.genome.jp/dbget-bin/www_bget?xcc:XCC0672) | minus | 4 | conserved | [XCC0671](http://www.genome.jp/dbget-bin/www_bget?xcc:XCC0671) | plus | 232 |
| 31 | GGGAATAGGGAGAGGGGAATAGGGAATC | minus | 28 | 4 | 834046 | 834019 | other | 33 | conserved, nrdR transcriptional regulator | [XCC0691](http://www.genome.jp/dbget-bin/www_bget?xcc:XCC0691) | plus | 9 | glyA | [XCC0690](http://www.genome.jp/dbget-bin/www_bget?xcc:XCC0690) | plus | 64 |
| 32 | GGGAATCGGGAGAGGGGAATCGGGAATC | minus | 28 | 4 | 840289 | 840262 | other | 40 | thiL | [XCC0699](http://www.genome.jp/dbget-bin/www_bget?xcc:XCC0699) | plus | 6 | nusB | [XCC0698](http://www.genome.jp/dbget-bin/www_bget?xcc:XCC0698) | plus | 76 |
| 33 | GGGAATCGGGAATCGGGAATAGGGAGTCGGGACGA | minus | 35 | 5 | 841557 | 841523 | no | n.a. | conserved | [XCC0700](http://www.genome.jp/dbget-bin/www_bget?xcc:XCC0700) | plus | 114 | thiL | [XCC0699](http://www.genome.jp/dbget-bin/www_bget?xcc:XCC0699) | plus | 252 |
| 34 | GGGAATCGGGAATCGGGAATCGGGAATCGGGAATCGGGAATCGGGAATCGGGAATCGGGAATCGGGAATC | minus | 70 | 10 | 848804 | 848735 | no | n.a. | kdpD | [XCC0705](http://www.genome.jp/dbget-bin/www_bget?xcc:XCC0705) | plus | 2 | kdpC | [XCC0704](http://www.genome.jp/dbget-bin/www_bget?xcc:XCC0704) | plus | 26 |
| 35 | GGGAATGGAGAATCGGGAATCGGGAATG | plus | 28 | 4 | 861737 | 861710 | 36 | 28 | intragenic: mraW | [XCC0718](http://www.genome.jp/dbget-bin/www_bget?xcc:XCC0718) | plus |  |  |  |  |  |
| 36 | GGGAATCGGGAATCGGGAGTGGGGAATC | minus | 28 | 4 | 861793 | 861766 | 35 | 28 | intragenic: mraW | [XCC0718](http://www.genome.jp/dbget-bin/www_bget?xcc:XCC0718) | plus |  |  |  |  |  |
| 37 | GCGAATCGGGAATCGAGAATCGGGAATCGGGTGAA | minus | 35 | 5 | 870659 | 870625 | other | 87 | intragenic: murG | [XCC0725](http://www.genome.jp/dbget-bin/www_bget?xcc:XCC0725) | plus |  |  |  |  |  |
| 38 | GGGAATGGGGAATCGGGAATCGGGAATG | plus | 28 | 4 | 873071 | 873098 | 39 | 23 | ddl | [XCC0727](http://www.genome.jp/dbget-bin/www_bget?xcc:XCC0727) | plus | 4 | ftsQ | [XCC0728](http://www.genome.jp/dbget-bin/www_bget?xcc:XCC0728) | plus | 68 |
| 39 | GGGAATGGGGAATCGGGAATAGGGAATC | minus | 28 | 4 | 873155 | 873128 | 38 | 23 | ftsQ | [XCC0728](http://www.genome.jp/dbget-bin/www_bget?xcc:XCC0728) | plus | 17 | ddl | [XCC0727](http://www.genome.jp/dbget-bin/www_bget?xcc:XCC0727) | plus | 61 |
| 40 | GCGAATGGAGAATGGGGAATCGGGAATG | plus | 28 | 4 | 873856 | 873883 | other | 83 | intragenic: ftsQ | [XCC0728](http://www.genome.jp/dbget-bin/www_bget?xcc:XCC0728) | plus |  |  |  |  |  |
| 41 | GGGAATCGGGAATCGGGAATCGGGAATC | plus | 28 | 4 | 875248 | 875275 | other | 131 | ftsA | [XCC0729](http://www.genome.jp/dbget-bin/www_bget?xcc:XCC0729) | plus | 6 | ftsZ | [XCC0730](http://www.genome.jp/dbget-bin/www_bget?xcc:XCC0730) | plus | 261 |
| 42 | GGGAATCGGGAATGGGGAATC | minus | 21 | 3 | 875508 | 875488 | no | n.a. | ftsZ | [XCC0730](http://www.genome.jp/dbget-bin/www_bget?xcc:XCC0730) | plus | 28 | ftsA | [XCC0729](http://www.genome.jp/dbget-bin/www_bget?xcc:XCC0729) | plus | 246 |
| 43 | GGGAATCGGGCATG | minus | 14 | 2 | 883412 | 883399 | other | 162 | hypothetical protein | [XCC0736](http://www.genome.jp/dbget-bin/www_bget?xcc:XCC0736) | plus | -13 | conserved | [XCC0735](http://www.genome.jp/dbget-bin/www_bget?xcc:XCC0735) | plus | 178 |
| 44 | GGGATTGGGGATTGGGGATTGGGGATTGGGGAATCGGGAATCGGGAATCGGGAATCGGGAATCGGGTGCA | minus | 70 | 10 | 924137 | 924068 | no | n.a. | voltage-gated potassium channel beta subunit | [XCC0766](http://www.genome.jp/dbget-bin/www_bget?xcc:XCC0766) | plus | 74 | yeiM | [XCC0765](http://www.genome.jp/dbget-bin/www_bget?xcc:XCC0765) | minus | 452 |
| 45 | GGGATTCGGGAATCGGGATTGGGGAATC | minus | 28 | 4 | 974540 | 974513 | no | n.a. | conserved | [XCC0816](http://www.genome.jp/dbget-bin/www_bget?xcc:XCC0816) | minus | 5 | gfo | [XCC0815](http://www.genome.jp/dbget-bin/www_bget?xcc:XCC0815) | plus | 234 |
| 46 | GGGAGACGGGAATC | minus | 14 | 2 | 1036433 | 1036420 | no | n.a. | tufA | [XCC0880](http://www.genome.jp/dbget-bin/www_bget?xcc:XCC0880) | plus | 161 | ychF | [XCC0876](http://www.genome.jp/dbget-bin/www_bget?xcc:XCC0876) | plus | 320 |
| 47 | GGGAATCGGGAGTGGGGAATCGGGAATCGGGAATC | plus | 35 | 5 | 1116950 | 1116984 | other | 67 | gtrB | [XCC0959](http://www.genome.jp/dbget-bin/www_bget?xcc:XCC0959) | minus | 83 | ppx | [XCC0960](http://www.genome.jp/dbget-bin/www_bget?xcc:XCC0960) | minus | 118 |
| 48 | GGGAATCGGGAATCGGGATTGGGGAATC | minus | 28 | 4 | 1127004 | 1126977 | no | n.a. | conserved | [XCC0968](http://www.genome.jp/dbget-bin/www_bget?xcc:XCC0968) | minus | 3 | icd | [XCC0967](http://www.genome.jp/dbget-bin/www_bget?xcc:XCC0967) | plus | 110 |
| 49 | GGGAATCGGGAGTAGGGAATCGGGAATC | plus | 28 | 4 | 1152989 | 1153016 | 50 | 28 | conserved | [XCC1002](http://www.genome.jp/dbget-bin/www_bget?xcc:XCC1002) | plus | 6 | recR | [XCC1003](http://www.genome.jp/dbget-bin/www_bget?xcc:XCC1003) | plus | 62 |
| 50 | GGGAATCGGGAATGGGGATTGGGGAATC | minus | 28 | 4 | 1153072 | 1153045 | 49 | 28 | recR | [XCC1003](http://www.genome.jp/dbget-bin/www_bget?xcc:XCC1003) | plus | 6 | conserved | [XCC1002](http://www.genome.jp/dbget-bin/www_bget?xcc:XCC1002) | plus | 62 |
| 51 | GGGAATCGTAAGTCGTGAATGGGGAATC | plus | 28 | 4 | 1153678 | 1153705 | 52 | 39 | recR | [XCC1003](http://www.genome.jp/dbget-bin/www_bget?xcc:XCC1003) | plus | 5 | histidine triad-like protein | [XCC1004](http://www.genome.jp/dbget-bin/www_bget?xcc:XCC1004) | plus | 72 |
| 52 | GGGAATCGAGAATCGGGATTGGGGAATC | minus | 28 | 4 | 1153772 | 1153745 | 51 | 39 | histidine triad-like protein | [XCC1004](http://www.genome.jp/dbget-bin/www_bget?xcc:XCC1004) | plus | 5 | recR | [XCC1003](http://www.genome.jp/dbget-bin/www_bget?xcc:XCC1003) | plus | 72 |
| 53 | GGGAATCGGGAATCGGGAATCGGGAATCGGGAATCGGGAATCGGGAATCGGGTAGA | minus | 56 | 8 | 1181254 | 1181199 | no | n.a. | acnA | [XCC1033](http://www.genome.jp/dbget-bin/www_bget?xcc:XCC1033) | plus | 38 | prpC | [XCC1032](http://www.genome.jp/dbget-bin/www_bget?xcc:XCC1032) | plus | 104 |
| 54 | GGGCTGCGGGAACTGGGTGAG | plus | 21 | 3 | 1246341 | 1246361 | no |  | intragenic: oxidoreductase | [XCC1079](http://www.genome.jp/dbget-bin/www_bget?xcc:XCC1079) | minus |  |  |  |  |  |
| 55 | GGGAATGGGGAATCGGGAGTGGGGAATC | plus | 28 | 4 | 1253794 | 1253821 | no | n.a. | conserved | [XCC1085](http://www.genome.jp/dbget-bin/www_bget?xcc:XCC1085) | plus | 18 | conserved | [XCC1086](http://www.genome.jp/dbget-bin/www_bget?xcc:XCC1086) | minus | 217 |
| 56 | GGGAATCGGGAGTAGGGAGTCGGGAATC | plus | 28 | 4 | 1338603 | 1338630 | 57 | 89 | rpmA | [XCC1150](http://www.genome.jp/dbget-bin/www_bget?xcc:XCC1150) | plus | 93 | GTPase ObgE | [XCC1151](http://www.genome.jp/dbget-bin/www_bget?xcc:XCC1151) | plus | 129 |
| 57 | GGGAATGGGGAATAGGGAATGGGGAATC | minus | 28 | 4 | 1338747 | 1338720 | 56 | 89 | GTPase ObgE | [XCC1151](http://www.genome.jp/dbget-bin/www_bget?xcc:XCC1151) | plus | 12 | rpmA | [XCC1150](http://www.genome.jp/dbget-bin/www_bget?xcc:XCC1150) | plus | 210 |
| 58 | GGGTCACGGGAATCT | minus | 14 | 2 | 1343221 | 1343207 | no |  | ileS | [XCC1155](http://www.genome.jp/dbget-bin/www_bget?xcc:XCC1155) | plus | -7 | ribF | [XCC1154](http://www.genome.jp/dbget-bin/www_bget?xcc:XCC1154) | plus | -2 |
| 59 | GGGATTCGGGAATCGGGATTC | plus | 21 | 3 | 1394256 | 1394276 | no | n.a. | conserved | [XCC1193](http://www.genome.jp/dbget-bin/www_bget?xcc:XCC1193) | minus | 28 | ffh | [XCC1194](http://www.genome.jp/dbget-bin/www_bget?xcc:XCC1194) | plus | 241 |
| 60 | GGGAATCGGGAATCGGGACTGGGAATGGGGAGAG | minus | 34 | 5 | 1401201 | 1401168 | no | n.a. | trmD | [XCC1201](http://www.genome.jp/dbget-bin/www_bget?xcc:XCC1201) | plus | 2 | rimM | [XCC1200](http://www.genome.jp/dbget-bin/www_bget?xcc:XCC1200) | plus | 28 |
| 61 | GGGAATCGGGAGTTGGGAGTGGGGAATC | plus | 28 | 4 | 1487167 | 1487194 | 62 | 41 | mucD | [XCC1269](http://www.genome.jp/dbget-bin/www_bget?xcc:XCC1269) | plus | 6 | lepA | [XCC1270](http://www.genome.jp/dbget-bin/www_bget?xcc:XCC1270) | plus | 131 |
| 62 | GGGATTGGGGAATCGGGAATG | minus | 21 | 3 | 1487256 | 1487236 | 61 | 41 | lepA | [XCC1270](http://www.genome.jp/dbget-bin/www_bget?xcc:XCC1270) | plus | 69 | mucD | [XCC1269](http://www.genome.jp/dbget-bin/www_bget?xcc:XCC1269) | plus | 75 |
| 63 | GGGAATGGTCAATCGGGAATCGGGAATC | plus | 28 | 4 | 1489134 | 1489161 | 64 | 28 | lepA | [XCC1270](http://www.genome.jp/dbget-bin/www_bget?xcc:XCC1270) | Plus | 2 | lepB | [XCC1271](http://www.genome.jp/dbget-bin/www_bget?xcc:XCC1271) | plus | 73 |
| 64 | GGGAATCGGGAATCGGGAATCGAGAATC | minus | 28 | 4 | 1489217 | 1489190 | 63 | 28 | lepB | [XCC1271](http://www.genome.jp/dbget-bin/www_bget?xcc:XCC1271) | plus | 17 | lepA | [XCC1270](http://www.genome.jp/dbget-bin/www_bget?xcc:XCC1270) | plus | 58 |
| 65 | GGGAATCGGGAATCGGGAGTTGGGAATG | plus | 28 | 4 | 1556030 | 1556057 | 66 | 91 | cicA | [XCC1338](http://www.genome.jp/dbget-bin/www_bget?xcc:XCC1338) | minus | 7 | metS / metG | [XCC1339](http://www.genome.jp/dbget-bin/www_bget?xcc:XCC1339) | minus | 123 |
| 66 | GGGAATCGGGAGTGGGGAATCGGGAATC | minus | 28 | 4 | 1556176 | 1556149 | 65 | 91 | metS / metG | [XCC1339](http://www.genome.jp/dbget-bin/www_bget?xcc:XCC1339) | minus | 4 | cicA | [XCC1338](http://www.genome.jp/dbget-bin/www_bget?xcc:XCC1338) | minus | 126 |
| 67 | GGGAATGGGGAATGGGGAATCGGGAATGGGGAATT | plus | 35 | 5 | 1572758 | 1572792 | 68 | 135 | conserved | [XCC1350](http://www.genome.jp/dbget-bin/www_bget?xcc:XCC1350) | minus | 183 | conserved | [XCC1351](http://www.genome.jp/dbget-bin/www_bget?xcc:XCC1351) | plus | 233 |
| 68 | GGGAATTGGGAGTGGGGAATCGGGAATC | minus | 28 | 4 | 1572955 | 1572928 | 67 | 135 | conserved | [XCC1351](http://www.genome.jp/dbget-bin/www_bget?xcc:XCC1351) | plus | 70 | conserved | [XCC1350](http://www.genome.jp/dbget-bin/www_bget?xcc:XCC1350) | minus | 353 |
| 69 | GGGAAATGGGGAATC | plus | 14 | 2 | 1579401 | 1579414 | 70 | 32 | accA | [XCC1357](http://www.genome.jp/dbget-bin/www_bget?xcc:XCC1357) | minus | 84 | dnaE1 | [XCC1358](http://www.genome.jp/dbget-bin/www_bget?xcc:XCC1358) | minus | 55 |
| 70 | GTGAATCGGGAATCGAGAATGGGGAATC | minus | 28 | 4 | 1579447 | 1579474 | 69 | 32 | dnaE1 | [XCC1358](http://www.genome.jp/dbget-bin/www_bget?xcc:XCC1358) | minus | -4 | accA | [XCC1357](http://www.genome.jp/dbget-bin/www_bget?xcc:XCC1357) | minus | 130 |
| 71 | GGGACTCGGGAGTCGGGAATGGGGAATC | plus | 28 | 4 | 1583930 | 1583957 | 72 | 71 | intragenic: rnhB | [XCC1359](http://www.genome.jp/dbget-bin/www_bget?xcc:XCC1359) | minus |  | lpxB | [XCC1360](http://www.genome.jp/dbget-bin/www_bget?xcc:XCC1360) | minus | 59 |
| 72 | GGGAATTGGGAATCGGGAATCGGGAATCGGGAGGA | minus | 35 | 5 | 1584063 | 1584029 | 71 | 71 | intragenic: lpxB | [XCC1360](http://www.genome.jp/dbget-bin/www_bget?xcc:XCC1360) | minus |  | rnhB | [XCC1359](http://www.genome.jp/dbget-bin/www_bget?xcc:XCC1359) | minus | 8 |
| 73 | GGGAATCGGGAGTGGGGAATGGGGAATC | plus | 28 | 4 | 1585223 | 1585250 | 74 | 57 | lpxB | [XCC1360](http://www.genome.jp/dbget-bin/www_bget?xcc:XCC1360) | minus | 9 | lpxA | [XCC1361](http://www.genome.jp/dbget-bin/www_bget?xcc:XCC1361) | minus | 79 |
| 74 | CGGAATCGGGAATCGGGAACC | minus | 21 | 3 | 1585328 | 1585308 | 73 | 57 | lpxA | [XCC1361](http://www.genome.jp/dbget-bin/www_bget?xcc:XCC1361) | minus | 1 | lpxB | [XCC1360](http://www.genome.jp/dbget-bin/www_bget?xcc:XCC1360) | minus | 94 |
| 75 | GGGAGTTGGGAATCGGGAATCGGGAATCGGGAATC | plus | 35 | 5 | 1648354 | 1648388 | 76 | 71 | fpr | [XCC1414](http://www.genome.jp/dbget-bin/www_bget?xcc:XCC1414) | minus | 77 | msbA | [XCC1415](http://www.genome.jp/dbget-bin/www_bget?xcc:XCC1415) | minus | 111 |
| 76 | GGGAATCGGGAATTGGGATTCGGGAATGGGTAGA | minus | 34 | 5 | 1648493 | 1648460 | 75 | 71 | msbA | [XCC1415](http://www.genome.jp/dbget-bin/www_bget?xcc:XCC1415) | minus | 6 | fpr | [XCC1414](http://www.genome.jp/dbget-bin/www_bget?xcc:XCC1414) | minus | 183 |
| 77 | GGGAATCGGGATTCGGGATTC | minus | 21 | 3 | 1714108 | 1714088 | other | 173 | smpB | [XCC1466](http://www.genome.jp/dbget-bin/www_bget?xcc:XCC1466) | minus | 6 | serine peptidase | [XCC1465](http://www.genome.jp/dbget-bin/www_bget?xcc:XCC1465) | plus | 197 |
| 78 | GGGAATCGGGAATCGGGAGAAGGGAATC | minus | 28 | 4 | 1722851 | 1722824 | other | 81 | dnaJ | [XCC1475](http://www.genome.jp/dbget-bin/www_bget?xcc:XCC1475) | plus | 4 | dnaK | [XCC1474](http://www.genome.jp/dbget-bin/www_bget?xcc:XCC1474) | plus | 106 |
| 79 | GGGAATCGGGAGTGGGGAATTGGGAATC | minus | 28 | 4 | 1735803 | 1735776 | other | 89 | ldp | [XCC1485](http://www.genome.jp/dbget-bin/www_bget?xcc:XCC1485) | minus | 3 | conserved | [XCC1484](http://www.genome.jp/dbget-bin/www_bget?xcc:XCC1484) | minus | 29 |
| 80 | GGGAATGGGGAGTAGGGAATGGGGAATC | plus | 28 | 4 | 1737257 | 1737284 | 81 | 72 | ldp | [XCC1485](http://www.genome.jp/dbget-bin/www_bget?xcc:XCC1485) | plus | 13 | sucB | [XCC1486](http://www.genome.jp/dbget-bin/www_bget?xcc:XCC1486) | minus | 105 |
| 81 | GGGAATCGGGAATGGAGATTCGGGAATG | minus | 28 | 4 | 1737384 | 1737357 | 80 | 72 | sucB | [XCC1486](http://www.genome.jp/dbget-bin/www_bget?xcc:XCC1486) | minus | 5 | ldp | [XCC1485](http://www.genome.jp/dbget-bin/www_bget?xcc:XCC1485) | minus | 113 |
| 82 | GGGAATCGGGAATCGGGAGTCGGGATTG | minus | 28 | 4 | 1769157 | 1769130 | other | 51 | intragenic: transcriptional | [XCC1513](http://www.genome.jp/dbget-bin/www_bget?xcc:XCC1513) | minus |  | metH1 | [XCC1512](http://www.genome.jp/dbget-bin/www_bget?xcc:XCC1512) | minus | 38 |
| 83 | GGGAATCGGGATTGGGGAATCGGGAATC | minus | 28 | 4 | 1824565 | 1824538 | no | n.a. | rpl1 | [XCC1565](http://www.genome.jp/dbget-bin/www_bget?xcc:XCC1565) | plus | 71 | rpsR | [XCC1564](http://www.genome.jp/dbget-bin/www_bget?xcc:XCC1564) | plus | 193 |
| 84 | GGGAGAGGGGAATC | minus | 14 | 2 | 1830895 | 1830882 | no | n.a. | hisC | [XCC1569](http://www.genome.jp/dbget-bin/www_bget?xcc:XCC1569) | minus | 169 | zipA | [XCC1568](http://www.genome.jp/dbget-bin/www_bget?xcc:XCC1568) | plus | -2 |
| 85 | GGGAATCGGGCAGT | plus | 14 | 2 | 1841107 | 1841120 | no |  | intragenic: gcd | [XCC1575](http://www.genome.jp/dbget-bin/www_bget?xcc:XCC1575) | plus |  |  |  |  |  |
| 86 | GGGAATTGGGAATGGGCAGTCGGGAATC | plus | 28 | 4 | 1985700 | 1985727 | other | 110 | nlpD | [XCC1709](http://www.genome.jp/dbget-bin/www_bget?xcc:XCC1709) | plus | 4 | conserved | [XCC1710](http://www.genome.jp/dbget-bin/www_bget?xcc:XCC1710) | minus | 135 |
| 87 | GGGCACGGCGTGTGGGGAATC | minus | 21 | 3 | 1993548 | 1993528 | no | n.a. | conserved | [XCC1718](http://www.genome.jp/dbget-bin/www_bget?xcc:XCC1718) | plus | 6 | hflX | [XCC1717](http://www.genome.jp/dbget-bin/www_bget?xcc:XCC1717) | plus | 384 |
| 88 | GGGAATCGGGAGTT*GGG*AATGGGGAATT | plus | 28 | 4 | 2133319 | 2133346 | 89 | 42 | yeiP | [XCC1830](http://www.genome.jp/dbget-bin/www_bget?xcc:XCC1830) | minus | 0 | hadH2 | [XCC1831](http://www.genome.jp/dbget-bin/www_bget?xcc:XCC1831) | minus | 72 |
| 89 | GGGGATGGGGAATCGAGAATGAGGAATC | minus | 28 | 4 | 2133416 | 2133389 | 88 | 42 | hadH2 | [XCC1831](http://www.genome.jp/dbget-bin/www_bget?xcc:XCC1831) | minus | 2 | yeiP | [XCC1830](http://www.genome.jp/dbget-bin/www_bget?xcc:XCC1830) | minus | 70 |
| 90 | GGGAATCGGGAATCGGGAATCGGGAATCGGGAATC | minus | 35 | 5 | 2224318 | 2224284 | no | n.a. | conserved | [XCC1900](http://www.genome.jp/dbget-bin/www_bget?xcc:XCC1900) | plus | 120 | IS1481 | [XCC1899](http://www.genome.jp/dbget-bin/www_bget?xcc:XCC1899) | plus | 177 |
| 91 | GGGAATGGGGAATCGGGATTCGGGAATC | plus | 28 | 4 | 2237582 | 2237555 | no | n.a. | diguanylate cyclase | [XCC1911](http://www.genome.jp/dbget-bin/www_bget?xcc:XCC1911) | minus | 363 | flhB | [XCC1910](http://www.genome.jp/dbget-bin/www_bget?xcc:XCC1910) | minus | 55 |
| 92 | GGGAATCGGGAATGGGGAATCGGGAATT | plus | 28 | 4 | 2283899 | 2283926 | 93 | 74 | flgF | [XCC1948](http://www.genome.jp/dbget-bin/www_bget?xcc:XCC1948) | minus | 8 | flgE | [XCC1949](http://www.genome.jp/dbget-bin/www_bget?xcc:XCC1949) | minus | 107 |
| 93 | GGGAATGGGGAATCGGGAGTAGGGAATC | minus | 28 | 4 | 2284028 | 2284001 | 92 | 74 | flgE | [XCC1949](http://www.genome.jp/dbget-bin/www_bget?xcc:XCC1949) | minus | 5 | flgF | [XCC1948](http://www.genome.jp/dbget-bin/www_bget?xcc:XCC1948) | minus | 110 |
| 94 | GAGAATCGGGAATCGGGAATCGGGAATC | plus | 28 | 4 | 2288244 | 2288271 | 94 | 86 | intragenic: flgA (directly after start codon) | [XCC1954](http://www.genome.jp/dbget-bin/www_bget?xcc:XCC1954) | plus |  | cheV | [XCC1953](http://www.genome.jp/dbget-bin/www_bget?xcc:XCC1953) | minus | 45 |
| 95 | GGGAATCGGGAGTGGGGAATCGGGAATC | minus | 28 | 4 | 2288385 | 2288358 | 95 | 86 | intragenic: flgA | [XCC1954](http://www.genome.jp/dbget-bin/www_bget?xcc:XCC1954) | plus |  | cheV | [XCC1953](http://www.genome.jp/dbget-bin/www_bget?xcc:XCC1953) | minus | 158 |
| 96 | GGGAATGGGGAATCGGGAATCGGGAATGGGGAATC | plus | 35 | 5 | 2291127 | 2291161 | 97 | 84 | histidine kinase | [XCC1957](http://www.genome.jp/dbget-bin/www_bget?xcc:XCC1957) | plus | 11 | pdeA | [XCC1958](http://www.genome.jp/dbget-bin/www_bget?xcc:XCC1958) | minus | 104 |
| 97 | GGGAATAGGGAATCGGGAGTGGGGAATC | minus | 28 | 4 | 2291273 | 2291246 | 96 | 84 | pdeA | [XCC1958](http://www.genome.jp/dbget-bin/www_bget?xcc:XCC1958) | minus | -8 | histidine kinase | [XCC1957](http://www.genome.jp/dbget-bin/www_bget?xcc:XCC1957) | plus | 11 |
| 98 | GGGAATCGGGAATCGAGAGTGGGGAATG | plus | 28 | 4 | 2397567 | 2397594 | no | n.a. | sensor histidine kinase | [XCC2030](http://www.genome.jp/dbget-bin/www_bget?xcc:XCC2030) | minus | 272 | conserved | [XCC2031](http://www.genome.jp/dbget-bin/www_bget?xcc:XCC2031) | plus | 362 |
| 99 | GGGAATGGGGAATCGAGAGTGGGGAACG | plus | 28 | 4 | 2615715 | 2615742 | 100 | 76 | hydrolase | [XCC2217](http://www.genome.jp/dbget-bin/www_bget?xcc:XCC2217) | minus | 9 | ybdL | [XCC2218](http://www.genome.jp/dbget-bin/www_bget?xcc:XCC2218) | minus | 110 |
| 100 | GGGATTCGGGAATCGGGATTGGGGATTC | minus | 28 | 4 | 2615846 | 2615819 | 99 | 76 | ybdL | [XCC2218](http://www.genome.jp/dbget-bin/www_bget?xcc:XCC2218) | minus | 6 | hydrolase | [XCC2217](http://www.genome.jp/dbget-bin/www_bget?xcc:XCC2217) | minus | 113 |
| 101 | GGGAATGGGGAGTGGGGAATCGGGAATG | plus | 28 | 4 | 2637783 | 2637810 | 102 | 69 | proA | [XCC2238](http://www.genome.jp/dbget-bin/www_bget?xcc:XCC2238) | minus | 11 | proB | [XCC2239](http://www.genome.jp/dbget-bin/www_bget?xcc:XCC2239) | minus | 99 |
| 102 | GCGAATCGGGAATCGGGACTTGGGAATC | minus | 28 | 4 | 2637907 | 2637880 | 101 | 69 | proB | [XCC2239](http://www.genome.jp/dbget-bin/www_bget?xcc:XCC2239) | minus | 2 | proA | [XCC2238](http://www.genome.jp/dbget-bin/www_bget?xcc:XCC2238) | minus | 108 |
| 103 | GGGAATGGGGAATCGGGATTCGGGAATC | minus | 28 | 4 | 2669627 | 2669600 | no | n.a. | ubiG | [XCC2269](http://www.genome.jp/dbget-bin/www_bget?xcc:XCC2269) | minus | 6 | cbbZ | [XCC2268](http://www.genome.jp/dbget-bin/www_bget?xcc:XCC2268) | minus | 78 |
| 104 | GGGAATCGGGAATGGTGAATCGGGAATG | plus | 28 | 4 | 2683661 | 2683688 | 105 | 18 | uup | [XCC2281](http://www.genome.jp/dbget-bin/www_bget?xcc:XCC2281) | minus | 13 | dbpA | [XCC2282](http://www.genome.jp/dbget-bin/www_bget?xcc:XCC2282) | plus | 59 |
| 105 | GGGAATCGGGAGTGGTGAATCGGGAATC | minus | 28 | 4 | 2683734 | 2683707 | 104 | 18 | dbpA | [XCC2282](http://www.genome.jp/dbget-bin/www_bget?xcc:XCC2282) | plus | 13 | uup | [XCC2281](http://www.genome.jp/dbget-bin/www_bget?xcc:XCC2281) | minus | 59 |
| 106 | GGGAATCGGGAGTTGGGAATCGGGAATGGGGGGTC | plus | 35 | 5 | 2782275 | 2782309 | no | n.a. | two | [XCC2361](http://www.genome.jp/dbget-bin/www_bget?xcc:XCC2361) | plus | 5 | yieO | [XCC2362](http://www.genome.jp/dbget-bin/www_bget?xcc:XCC2362) | plus | 288 |
| 107 | GGGAATGGGGAATGGGGAATCGGGAGTCGGGAATC | plus | 35 | 5 | 2812110 | 2812144 | 108 | 44 | tgt | [XCC2378](http://www.genome.jp/dbget-bin/www_bget?xcc:XCC2378) | minus | 9 | queA | [XCC2379](http://www.genome.jp/dbget-bin/www_bget?xcc:XCC2379) | minus | 40 |
| 108 | GGGAGTGGGGAATCGGGAATGGGGAATC | minus | 28 | 4 | 2812217 | 2812190 | 107 | 44 | intragenic: queA | [XCC2379](http://www.genome.jp/dbget-bin/www_bget?xcc:XCC2379) | minus |  | tgt | [XCC2378](http://www.genome.jp/dbget-bin/www_bget?xcc:XCC2378) | minus | 89 |
| 109 | GGGAATCGGGAGTGGGGAATCGGGAATC | minus | 28 | 4 | 2813554 | 2813527 | no | n.a. | AsnC family transcriptional regulator | [XCC2380](http://www.genome.jp/dbget-bin/www_bget?xcc:XCC2380) | minus | 6 | queA | [XCC2379](http://www.genome.jp/dbget-bin/www_bget?xcc:XCC2379) | minus | 270 |
| 110 | GGGAGCCGGGAAAAGAGAATAGGGAATCGGGAATC | plus | 35 | 5 | 2921142 | 2921176 | 111 | 60 | pheT | [XCC2458](http://www.genome.jp/dbget-bin/www_bget?xcc:XCC2458) | minus | 1 | pheS | [XCC2459](http://www.genome.jp/dbget-bin/www_bget?xcc:XCC2459) | minus | 93 |
| 111 | GGAAATCGGGAATAGGGAATAGAGAATC | minus | 28 | 4 | 2921264 | 2921237 | 110 | 60 | pheS | [XCC2459](http://www.genome.jp/dbget-bin/www_bget?xcc:XCC2459) | minus | 5 | pheT | [XCC2458](http://www.genome.jp/dbget-bin/www_bget?xcc:XCC2458) | minus | 96 |
| 112 | GAGATTCGGGAATCGGGAAGCGGGAAGCGGGAAGCGGGAAGCGGGAAGCGGGAATC | plus | 56 | 8 | 2927336 | 2927391 | no | n.a. | maltose transport gene repressor | [XCC2464](http://www.genome.jp/dbget-bin/www_bget?xcc:XCC2464) | plus | -2 | cgt | [XCC2465](http://www.genome.jp/dbget-bin/www_bget?xcc:XCC2465) | minus | 146 |
| 113 | GGGAATCGGGAGAA | minus | 14 | 2 | 3051301 | 3051288 | no | n.a. | oar | [XCC2573](http://www.genome.jp/dbget-bin/www_bget?xcc:XCC2573) | plus | 17 | btuB | [XCC2572](http://www.genome.jp/dbget-bin/www_bget?xcc:XCC2572) | plus | 331 |
| 114 | GGGAATCGGGAATCGGGAATGGGGAATT | minus | 28 | 4 | 3089518 | 3089491 | gleich mit 115 | 59 | tldD | [XCC2606](http://www.genome.jp/dbget-bin/www_bget?xcc:XCC2606) | minus | 93 | conserved | [XCC2605](http://www.genome.jp/dbget-bin/www_bget?xcc:XCC2605) | plus | 48 |
| 115 | GGGAATCGGGAATCGGGAGTGGGGAATC | minus | 28 | 4 | 3089605 | 3089578 | gleich mit 114 | 59 | tldD | [XCC2606](http://www.genome.jp/dbget-bin/www_bget?xcc:XCC2606) | minus | 6 | conserved | [XCC2605](http://www.genome.jp/dbget-bin/www_bget?xcc:XCC2605) | plus | 135 |
| 116 | GGGAATCGGGAGTCGGGAGTCGGGAGTC | plus | 28 | 4 | 3095823 | 3095850 | 117 | 94 | conserved | [XCC2608](http://www.genome.jp/dbget-bin/www_bget?xcc:XCC2608) | minus | 8 | rng | [XCC2609](http://www.genome.jp/dbget-bin/www_bget?xcc:XCC2609) | minus | 133 |
| 117 | GAGAGTCGGGAATCGGGAATGGGGGAAG | minus | 28 | 4 | 3095972 | 3095945 | 116 | 94 | rng | [XCC2609](http://www.genome.jp/dbget-bin/www_bget?xcc:XCC2609) | minus | 11 | conserved | [XCC2608](http://www.genome.jp/dbget-bin/www_bget?xcc:XCC2608) | minus | 130 |
| 118 | GGGAATCGGGAATCGGGAATCGGGAGTC | plus | 28 | 4 | 3103834 | 3103861 | other |  | intragenic: nadD | [XCC2616](http://www.genome.jp/dbget-bin/www_bget?xcc:XCC2616) | minus |  |  |  |  |  |
| 119 | GTGAATGGGGAATCGGGAATGGGGAATC | plus | 28 | 4 | 3112642 | 3112669 | no | n.a. | peptidyl-prolyl cis-trans isomerase | [XCC2625](http://www.genome.jp/dbget-bin/www_bget?xcc:XCC2625) | plus | -4 | conserved | [XCC2626](http://www.genome.jp/dbget-bin/www_bget?xcc:XCC2626) | minus | 41 |
| 120 | TGGAATTGGGAATTGGGAATTGGGAATTGGGAATTGGGAATTGGGAATTGGGAATCGGGAATTGGGAATTGGGAATTGGGAATTGGGAATTGGGAATTGGGAATTGGGAATCGGGAATCGGGAATCGGGAATCGGGAATCGGGAATCGGGAATCGGGAATC | plus | 161 | 23 | 3257022 | 3257182 | no | n.a. | osmC | [XCC2745](http://www.genome.jp/dbget-bin/www_bget?xcc:XCC2745) | minus | 64 | pyrB | [XCC2746](http://www.genome.jp/dbget-bin/www_bget?xcc:XCC2746) | minus | 219 |
| 121 | GGGAATAGGGAATCGAGAATCGGGAATT | plus | 28 | 4 | 3266373 | 3266400 | 122 | 44 | pilU | [XCC2754](http://www.genome.jp/dbget-bin/www_bget?xcc:XCC2754) | minus | 19 | pilT | [XCC2755](http://www.genome.jp/dbget-bin/www_bget?xcc:XCC2755) | minus | 75 |
| 122 | GTGACTCGGGAATAGGGAATCGGGAATGGGGAATT | minus | 35 | 5 | 3266445 | 3266479 | 121 | 44 | pilT | [XCC2755](http://www.genome.jp/dbget-bin/www_bget?xcc:XCC2755) | minus | -4 | pilU | [XCC2754](http://www.genome.jp/dbget-bin/www_bget?xcc:XCC2754) | minus | 91 |
| 123 | GGGAATCGGGAATGGAGAATC | plus | 21 | 3 | 3279648 | 3279668 | 124 | 70 | nifS | [XCC2769](http://www.genome.jp/dbget-bin/www_bget?xcc:XCC2769) | plus | 8 | acetyltransferase | [XCC2770](http://www.genome.jp/dbget-bin/www_bget?xcc:XCC2770) | plus | 183 |
| 124 | GGGAATCGGGAATCGGGAATCGGGAATCGGGAGTCGGGAGTCGGGAATCGGGAATCGGGAATCGGGAGTCGGGAATCGGGAATCGGGAATCGGGAAAA | minus | 98 | 14 | 3279836 | 3279739 | 123 | 70 | acetyltransferase | [XCC2770](http://www.genome.jp/dbget-bin/www_bget?xcc:XCC2770) | plus | 15 | nifS | [XCC2769](http://www.genome.jp/dbget-bin/www_bget?xcc:XCC2769) | plus | 99 |
| 125 | GGGATTGGGGAATCGGGATTCGGGAATCGGGAATCGGGAATCGGGAATC | plus | 49 | 7 | 3312014 | 3312062 | no | n.a. | conserved | [XCC2806](http://www.genome.jp/dbget-bin/www_bget?xcc:XCC2806) | plus | 5 | conserved | [XCC2807](http://www.genome.jp/dbget-bin/www_bget?xcc:XCC2807) | plus | 453 |
| 126 | GGGTGGAGGGAATC | minus | 14 | 2 | 3384125 | 3384112 | no | n.a. | intragenic: conserved | [XCC2861](http://www.genome.jp/dbget-bin/www_bget?xcc:XCC2861) | plus |  |  |  |  |  |
| 127 | GGGATTGGGGAATCGGGAGTCGGGAATC | plus | 28 | 4 | 3567831 | 3567858 | no | n.a. | intragenic: conserved | [XCC3022](http://www.genome.jp/dbget-bin/www_bget?xcc:XCC3022) | minus |  | ruvB | [XCC3023](http://www.genome.jp/dbget-bin/www_bget?xcc:XCC3023) | minus | 61 |
| 128 | GGGCGCCGGGAATCGGGAATCGGGAATGGGGAATC | plus | 35 | 5 | 3572831 | 3572865 | 129 | 29 | ruvA | [XCC3025](http://www.genome.jp/dbget-bin/www_bget?xcc:XCC3025) | minus | 15 | ruvC | [XCC3026](http://www.genome.jp/dbget-bin/www_bget?xcc:XCC3026) | minus | 62 |
| 129 | GAGAATCGGGAATCGGGAATCGGGAATCGGGAATC | minus | 35 | 5 | 3572929 | 3572895 | 128 | 29 | ruvC | [XCC3026](http://www.genome.jp/dbget-bin/www_bget?xcc:XCC3026) | minus | -2 | ruvA | [XCC3025](http://www.genome.jp/dbget-bin/www_bget?xcc:XCC3025) | minus | 79 |
| 130 | GGGAGTGGGGAATCGGGAATCGGGAATG | plus | 28 | 4 | 3573462 | 3573489 | 131 | 59 | ruvC | [XCC3026](http://www.genome.jp/dbget-bin/www_bget?xcc:XCC3026) | minus | 9 | conserved | [XCC3027](http://www.genome.jp/dbget-bin/www_bget?xcc:XCC3027) | minus | 92 |
| 131 | GGGAATCGGGAATGGAGAATCGGGAATC | minus | 28 | 4 | 3573576 | 3573549 | 130 | 59 | conserved | [XCC3027](http://www.genome.jp/dbget-bin/www_bget?xcc:XCC3027) | minus | 5 | ruvC | [XCC3026](http://www.genome.jp/dbget-bin/www_bget?xcc:XCC3026) | minus | 96 |
| 132 | GGGAATCGGGAATCGGGGATGGGGAATC | minus | 28 | 4 | 3594334 | 3594307 | no | n.a. | intragenic: yheS | [XCC3042](http://www.genome.jp/dbget-bin/www_bget?xcc:XCC3042) | minus |  |  |  |  |  |
| 133 | GGGAATTGGGAGTGGGGAATCGGGAATC | plus | 28 | 4 | 3671168 | 3671195 | 134 | 349 | pilR | [XCC3096](http://www.genome.jp/dbget-bin/www_bget?xcc:XCC3096) | plus | 7 | pilB | [XCC3097](http://www.genome.jp/dbget-bin/www_bget?xcc:XCC3097) | minus | 383 |
| 134 | GGGAATTGGGAATCGGGAGTGGGGAATC | minus | 28 | 4 | 3671572 | 3671545 | 133 | 349 | pilB | [XCC3097](http://www.genome.jp/dbget-bin/www_bget?xcc:XCC3097) | minus | 6 | pilR | [XCC3096](http://www.genome.jp/dbget-bin/www_bget?xcc:XCC3096) | plus | 384 |
| 135 | GGGCGATGGGAATC | plus | 14 | 2 | 3728588 | 3728601 | no | n.a. | intS | [XCC3146](http://www.genome.jp/dbget-bin/www_bget?xcc:XCC3146) | minus | 237 | estA | [XCC3148](http://www.genome.jp/dbget-bin/www_bget?xcc:XCC3148) | minus | 176 |
| 136 | GGGAATCGGGAATGGAGAATCGGGAATC | plus | 28 | 4 | 3732666 | 3732693 | 137 | 61 | intragenic: micA | [XCC3151](http://www.genome.jp/dbget-bin/www_bget?xcc:XCC3151) | plus |  | sac1 | [XCC3152](http://www.genome.jp/dbget-bin/www_bget?xcc:XCC3152) | plus | 97 |
| 137 | GGGAATGGGGAGTCGGGAATGGGGAATG | minus | 28 | 4 | 3732782 | 3732755 | 136 | 61 | sac1 | [XCC3152](http://www.genome.jp/dbget-bin/www_bget?xcc:XCC3152) | plus | 8 | micA / trmB | [XCC3151](http://www.genome.jp/dbget-bin/www_bget?xcc:XCC3151) | plus | 30 |
| 138 | GGGATTGGGGAGTGGGGAATCGGGAATC | plus | 28 | 4 | 3765772 | 3765799 | 139 | 48 | intragenic: nodQ | [XCC3171](http://www.genome.jp/dbget-bin/www_bget?xcc:XCC3171) | minus |  | cysD | [XCC3172](http://www.genome.jp/dbget-bin/www_bget?xcc:XCC3172) | minus | 82 |
| 139 | GTGAATCGGGAATCGGGAATGGGGAATC | minus | 28 | 4 | 3765875 | 3765848 | 138 | 48 | cysD | [XCC3172](http://www.genome.jp/dbget-bin/www_bget?xcc:XCC3172) | minus | 6 | intragenisch: nodQ | [XCC3171](http://www.genome.jp/dbget-bin/www_bget?xcc:XCC3171) | minus |  |
| 140 | GGGAATCGGGAGTGGGGAATCGGGAATG | plus | 28 | 4 | 3768821 | 3768848 | 141 | 41 | cysJ | [XCC3173](http://www.genome.jp/dbget-bin/www_bget?xcc:XCC3173) | plus | 4 | cysI | [XCC3174](http://www.genome.jp/dbget-bin/www_bget?xcc:XCC3174) | plus | 76 |
| 141 | GGGTCATGGGAATCGGGAATCGGGAAAGGGGAATCGTGAAAA | minus | 42 | 6 | 3768931 | 3768890 | 140 | 41 | cysI | [XCC3174](http://www.genome.jp/dbget-bin/www_bget?xcc:XCC3174) | plus | -7 | cysJ | [XCC3173](http://www.genome.jp/dbget-bin/www_bget?xcc:XCC3173) | plus | 73 |
| 142 | GGGAATGGGGAATGGGGAATGGGGAATGGGGAATGGGGAATC | plus | 42 | 6 | 3788317 | 3788358 | no | n.a. | pgk | [XCC3188](http://www.genome.jp/dbget-bin/www_bget?xcc:XCC3188) | minus | 24 | conserved | [XCC3189](http://www.genome.jp/dbget-bin/www_bget?xcc:XCC3189) | minus | 254 |
| 143 | GGGAATCAGCAGCCGGGAATGGAGAAACGGGAATCGGGAATC | plus | 42 | 6 | 3825514 | 3825555 | 144 | 106 | tktA | [XCC3220](http://www.genome.jp/dbget-bin/www_bget?xcc:XCC3220) | minus | 70 | gltP | [XCC3221](http://www.genome.jp/dbget-bin/www_bget?xcc:XCC3221) | minus | 126 |
| 144 | GGGAATCGGGAATCAGGAATC | minus | 21 | 3 | 3825682 | 3825664 | 143 | 106 | gltP | [XCC3221](http://www.genome.jp/dbget-bin/www_bget?xcc:XCC3221) | minus | -1 | tktA | [XCC3220](http://www.genome.jp/dbget-bin/www_bget?xcc:XCC3220) | minus | 218 |
| 145 | GGGAATCGAGAGTCGGGAATAGGGAATGGGGCGCC | plus | 35 | 5 | 3862480 | 3862514 | 146 | 42 | gmk | [XCC3249](http://www.genome.jp/dbget-bin/www_bget?xcc:XCC3249) | minus | 4 | conserved | [XCC3250](http://www.genome.jp/dbget-bin/www_bget?xcc:XCC3250) | minus | 72 |
| 146 | GGAAATAGGCAATCGGGAATGGGGAATC | minus | 28 | 4 | 3862584 | 3862557 | 145 | 42 | conserved | [XCC3250](http://www.genome.jp/dbget-bin/www_bget?xcc:XCC3250) | minus | 2 | gmk | [XCC3249](http://www.genome.jp/dbget-bin/www_bget?xcc:XCC3249) | minus | 81 |
| 147 | GGGATTAGGGAATCGGGATTGGGGAATC | minus | 28 | 4 | 3876313 | 3876286 | other | 31 | rpiA | [XCC3265](http://www.genome.jp/dbget-bin/www_bget?xcc:XCC3265) | plus | 12 | conserved | [XCC3264](http://www.genome.jp/dbget-bin/www_bget?xcc:XCC3264) | plus | 61 |
| 148 | GGGAATCGAGAGTGGGGAATCGGGAATG | minus | 28 | 4 | 3901096 | 3901069 | other | 63 | serine/threonine protein kinase conserved | [XCC3286](http://www.genome.jp/dbget-bin/www_bget?xcc:XCC3286) | minus | 6 | short chain dehydrogenase | [XCC3285](http://www.genome.jp/dbget-bin/www_bget?xcc:XCC3285) | minus | 91 |
| 149 | GGGAACCGGGAATC | plus | 14 | 2 | 3943111 | 3943124 | no | n.a. | intragenic: thiC | [XCC3319](http://www.genome.jp/dbget-bin/www_bget?xcc:XCC3319) | minus |  |  |  |  |  |
| 150 | GGGAATCGAGACTCGGGAATAGGGAATC | minus | 28 | 4 | 3959681 | 3959654 | other | 28 | leuC | [XCC3331](http://www.genome.jp/dbget-bin/www_bget?xcc:XCC3331) | minus | -22 | leuD | [XCC3330](http://www.genome.jp/dbget-bin/www_bget?xcc:XCC3330) | minus | 58 |
| 151 | GGGAATCGTGAATAGGGAATGGGGAATC | plus | 28 | 4 | 3992698 | 3992725 | 152 | 91 | tctD | [XCC3352](http://www.genome.jp/dbget-bin/www_bget?xcc:XCC3352) | minus | 7 | oprO | [XCC3353](http://www.genome.jp/dbget-bin/www_bget?xcc:XCC3353) | plus | 231 |
| 152 | GGGATTGGGGAGTCGGGATTGGGGAATC | minus | 28 | 4 | 3992844 | 3992817 | 151 | 91 | oprO | [XCC3353](http://www.genome.jp/dbget-bin/www_bget?xcc:XCC3353) | plus | 112 | tctD | [XCC3352](http://www.genome.jp/dbget-bin/www_bget?xcc:XCC3352) | minus | 126 |
| 153 | GGGAATGGGGAATCGGGAATGGGGAATC | plus | 28 | 4 | 3995713 | 3995740 | 154 | 76 | citM | [XCC3354](http://www.genome.jp/dbget-bin/www_bget?xcc:XCC3354) | plus | 9 | phbB | [XCC3355](http://www.genome.jp/dbget-bin/www_bget?xcc:XCC3355) | plus | 125 |
| 154 | GGGAATGGGGAATCGGGAGTGGGGAATC | minus | 28 | 4 | 3995844 | 3995817 | 153 | 76 | phbB / fabG | [XCC3355](http://www.genome.jp/dbget-bin/www_bget?xcc:XCC3355) | plus | 21 | citM | [XCC3354](http://www.genome.jp/dbget-bin/www_bget?xcc:XCC3354) | plus | 113 |
| 155 | GGGATTCGGGATTCGGGATTCGGGATTCGGGATTCGGGATTCGGGATTCGGGATCGGGAATCGGGAATCGGGAATCGGGAATCGGGAATCGGGAATCGGGAATCGGGAATCGGGAATCGGGAATCGGGAATC | plus | 132 | 19 | 3998009 | 3998140 | no | n.a. | cebR | [XCC3356](http://www.genome.jp/dbget-bin/www_bget?xcc:XCC3356) | plus | 3 | suc1 | [XCC3357](http://www.genome.jp/dbget-bin/www_bget?xcc:XCC3357) | minus | 92 |
| 156 | GGGAGTGGAGAATCGGGAATC | minus | 21 | 3 | 4112428 | 4112408 | other | 30 | dacC | [XCC3456](http://www.genome.jp/dbget-bin/www_bget?xcc:XCC3456) | minus | 38 | conserved | [XCC3455](http://www.genome.jp/dbget-bin/www_bget?xcc:XCC3455) | plus | 63 |
| 157 | GGGAATCGGGAGTCGGGAGTCGGGAATC | plus | 28 | 4 | 4130504 | 4130531 | other | 35 | mreD | [XCC3468](http://www.genome.jp/dbget-bin/www_bget?xcc:XCC3468) | minus | 25 | intragenisch: mreC | [XCC3469](http://www.genome.jp/dbget-bin/www_bget?xcc:XCC3469) | minus |  |
| 158 | GGGATTGGGGAATTGGGAGTGGGGAATC | plus | 28 | 4 | 4131835 | 4131862 | 159 | 259 | mreC | [XCC3469](http://www.genome.jp/dbget-bin/www_bget?xcc:XCC3469) | minus | 10 | mreB | [XCC3470](http://www.genome.jp/dbget-bin/www_bget?xcc:XCC3470) | minus | 294 |
| 159 | GGGAATCGGGAATCGGGAGTGGGGAATC | minus | 28 | 4 | 4132149 | 4132122 | 158 | 259 | mreB | [XCC3470](http://www.genome.jp/dbget-bin/www_bget?xcc:XCC3470) | minus | 7 | mreC | [XCC3469](http://www.genome.jp/dbget-bin/www_bget?xcc:XCC3469) | minus | 297 |
| 160 | GGGAATCGGGAATCGGGAATCGGGAATC | plus | 28 | 4 | 4223226 | 4223253 | 161 | 46 | pheC | [XCC3537](http://www.genome.jp/dbget-bin/www_bget?xcc:XCC3537) | plus | 18 | conserved | [XCC3538](http://www.genome.jp/dbget-bin/www_bget?xcc:XCC3538) | minus | 94 |
| 161 | GGGAATGGGGAGTCGGGAATCGGGAATC | minus | 28 | 4 | 4223327 | 4223300 | 160 | 46 | conserved | [XCC3538](http://www.genome.jp/dbget-bin/www_bget?xcc:XCC3538) | minus | 20 | pheC | [XCC3537](http://www.genome.jp/dbget-bin/www_bget?xcc:XCC3537) | plus | 92 |
| 162 | GGGAGTCGGGATTGGGGATTGGGGAATC | plus | 28 | 4 | 4330647 | 4330674 | other | 42 | abc, metN | [XCC3630](http://www.genome.jp/dbget-bin/www_bget?xcc:XCC3630) | minus | 52 | conserved | [XCC3631](http://www.genome.jp/dbget-bin/www_bget?xcc:XCC3631) | minus | 73 |
| 163 | GGGAATCGGGATTGGGGAATCGGGATTC | plus | 28 | 4 | 4350196 | 4350223 | no | n.a. | lrp | [XCC3649](http://www.genome.jp/dbget-bin/www_bget?xcc:XCC3649) | plus | 11 | conserved | [XCC3650](http://www.genome.jp/dbget-bin/www_bget?xcc:XCC3650) | minus | 366 |
| 164 | GGGATTGGGGAGTTGGGAATCGGGATCG | plus | 28 | 4 | 4355523 | 4355550 | no | n.a. | conserved | [XCC3656](http://www.genome.jp/dbget-bin/www_bget?xcc:XCC3656) | plus | 7 | conserved | [XCC3657](http://www.genome.jp/dbget-bin/www_bget?xcc:XCC3657) | minus | 1167 |
| 165 | GGGAATTGGGAATTGGGAGTGGGGAATC | minus | 28 | 4 | 43600266 | 4360239 | no | n.a. | yjcE | [XCC3661](http://www.genome.jp/dbget-bin/www_bget?xcc:XCC3661) | minus | 17 | yadG | [XCC3660](http://www.genome.jp/dbget-bin/www_bget?xcc:XCC3660) | minus | 367 |
| 166 | GGGAATGGGGAGTCGGGAATGGGAAATGGGGAATCGGGAATGGGGAATCGGGATTC | minus | 56 | 8 | 4414853 | 4414798 | no | n.a. | conserved | [XCC3710](http://www.genome.jp/dbget-bin/www_bget?xcc:XCC3710) | plus | 85 | yagR | [XCC3709](http://www.genome.jp/dbget-bin/www_bget?xcc:XCC3709) | minus | 359 |
| 167 | GAGGCTGGAGAAGCGGGAAGCGGGAATC | minus | 28 | 4 | 4500642 | 4500615 | no | n.a. | amaA | [XCC3786](http://www.genome.jp/dbget-bin/www_bget?xcc:XCC3786) | minus | -2 | queF, conserved | [XCC3785](http://www.genome.jp/dbget-bin/www_bget?xcc:XCC3785) | minus | 148 |
| 168 | GGGAATTGGGAATCGGGAATCGGGAGTG | minus | 28 | 4 | 4506900 | 4506873 | no | n.a. | acrA | [XCC3789](http://www.genome.jp/dbget-bin/www_bget?xcc:XCC3789) | plus | 12 | acrD | [XCC3788](http://www.genome.jp/dbget-bin/www_bget?xcc:XCC3788) | plus | 87 |
| 169 | GGGAATCGGGAGTTGGGAATAGGGAATAGGGAATG | plus | 35 | 5 | 4535056 | 4535090 | 170 | 57 | folB | [XCC3815](http://www.genome.jp/dbget-bin/www_bget?xcc:XCC3815) | minus | 6 | gcp | [XCC3816](http://www.genome.jp/dbget-bin/www_bget?xcc:XCC3816) | minus | 53 |
| 170 | GGGAGTCGGGAATCGGGAATCGGGAATC | minus | 28 | 4 | 4535175 | 4535148 | 169 | 57 | intragenic: gcp | [XCC3816](http://www.genome.jp/dbget-bin/www_bget?xcc:XCC3816) | minus | 0 | folB | [XCC3815](http://www.genome.jp/dbget-bin/www_bget?xcc:XCC3815) | minus | 98 |
| 171 | GGGAATCGAGAATCGGGAGTTGGGAATCGTGCAAA | plus | 35 | 5 | 4548084 | 4548118 | 172 | 22 | conserved | [XCC3828](http://www.genome.jp/dbget-bin/www_bget?xcc:XCC3828) | plus | 6 | cox3 | [XCC3829](http://www.genome.jp/dbget-bin/www_bget?xcc:XCC3829) | minus | 55 |
| 172 | GGGAATAGAGAGTGGGGAATGGGGAATC | minus | 28 | 4 | 4548141 | 4548168 | 171 | 22 | cox3 | [XCC3829](http://www.genome.jp/dbget-bin/www_bget?xcc:XCC3829) | minus | 5 | conserved | [XCC3828](http://www.genome.jp/dbget-bin/www_bget?xcc:XCC3828) | plus | 63 |
| 173 | GGTGCCTGGGAATC | minus | 14 | 2 | 4553225 | 4553212 | no | n.a. | putA | [XCC3835](http://www.genome.jp/dbget-bin/www_bget?xcc:XCC3835) | plus | 6 | conserved | [XCC3834](http://www.genome.jp/dbget-bin/www_bget?xcc:XCC3834) | minus | 303 |
| 174 | TGGAATGGGGAGTCGGGAATGGGGAATC | plus | 28 | 4 | 4570649 | 4570676 | 175 | 53 | exoA | [XCC3846](http://www.genome.jp/dbget-bin/www_bget?xcc:XCC3846) | minus | 6 | pyrE | [XCC3847](http://www.genome.jp/dbget-bin/www_bget?xcc:XCC3847) | plus | 86 |
| 175 | GGGAATGGGGAATAGGGAATCGGGAATG | minus | 28 | 4 | 4570757 | 4570730 | 174 | 53 | pyrE | [XCC3847](http://www.genome.jp/dbget-bin/www_bget?xcc:XCC3847) | plus | 5 | exoA | [XCC3846](http://www.genome.jp/dbget-bin/www_bget?xcc:XCC3846) | minus | 87 |
| 176 | GGGAATCGGGAATCGGGAATCGGGAGTTGGGAATCGGGAATC | plus | 42 | 6 | 4631079 | 4631120 | 177 | 25 | yhhT | [XCC3902](http://www.genome.jp/dbget-bin/www_bget?xcc:XCC3902) | plus | 2 | hydrolase | [XCC3903](http://www.genome.jp/dbget-bin/www_bget?xcc:XCC3903) | plus | 70 |
| 177 | GGGAATGGGGAGTCGGGAATCGAGAATG | minus | 28 | 4 | 4631173 | 4631146 | 176 | 25 | hydrolase | [XCC3903](http://www.genome.jp/dbget-bin/www_bget?xcc:XCC3903) | plus | 17 | yhhT | [XCC3902](http://www.genome.jp/dbget-bin/www_bget?xcc:XCC3902) | plus | 69 |
| 178 | GGGTTCGGGAATC | minus | 13 | 2 | 4715598 | 4715586 | no | n.a. | intragenic: apbA | [XCC3980](http://www.genome.jp/dbget-bin/www_bget?xcc:XCC3980) | plus |  |  |  |  |  |
| 179 | GGGAATGGGGAATCGAGAGTCGTGAATC | plus | 28 | 4 | 4718376 | 4718403 | 180 | 27 | conserved | [XCC3984](http://www.genome.jp/dbget-bin/www_bget?xcc:XCC3984) | minus | 9 | nrdF | [XCC3985](http://www.genome.jp/dbget-bin/www_bget?xcc:XCC3985) | minus | 61 |
| 180 | GGGAGTGGTGAATCGGGAATCGGGAATG | minus | 28 | 4 | 4718458 | 4718431 | 179 | 27 | nrdF | [XCC3985](http://www.genome.jp/dbget-bin/www_bget?xcc:XCC3985) | minus | 6 | conserved, flavodoxin | [XCC3984](http://www.genome.jp/dbget-bin/www_bget?xcc:XCC3984) | minus | 64 |
| 181 | GGGAGTGGGGAATCGGGAGTGGGGAATC | plus | 28 | 4 | 4818828 | 4818855 | 182 | 160 | acs | [XCC4060](http://www.genome.jp/dbget-bin/www_bget?xcc:XCC4060) | plus | 6 | tcsR | [XCC4061](http://www.genome.jp/dbget-bin/www_bget?xcc:XCC4061) | plus | 197 |
| 182 | GGGAATCGGGAGTGGGGAATCGGGAATT | minus | 28 | 4 | 4819043 | 4819016 | 181 | 160 | tcsR | [XCC4061](http://www.genome.jp/dbget-bin/www_bget?xcc:XCC4061) | plus | 9 | acs | [XCC4060](http://www.genome.jp/dbget-bin/www_bget?xcc:XCC4060) | plus | 194 |
| 183 | GGAAATCGGGAATCGGGAATCGGGAATCGGGAATCGGGAATGGGGAATGGGGAGTCGGGAATC | plus | 63 | 9 | 4841543 | 4841605 | no | n.a. | two | [XCC4076](http://www.genome.jp/dbget-bin/www_bget?xcc:XCC4076) | minus | 48 | ndvB | [XCC4077](http://www.genome.jp/dbget-bin/www_bget?xcc:XCC4077) | minus | 630 |
| 184 | GGGTGCCGGGAATC | plus | 14 | 2 | 4939174 | 4939187 | no | n.a. | tdk | [XCC4143](http://www.genome.jp/dbget-bin/www_bget?xcc:XCC4143) | plus | 31 | sensor histidine kinase | [XCC4144](http://www.genome.jp/dbget-bin/www_bget?xcc:XCC4144) | plus | 291 |
| 185 | GGGAATCGGGAATCGGGAGTGGGGAATC | minus | 28 | 4 | 5052404 | 5052377 | no | n.a. | intragenic: dipeptidase | [XCC4228](http://www.genome.jp/dbget-bin/www_bget?xcc:XCC4228) | minus | 0 | ttuB | [XCC4227](http://www.genome.jp/dbget-bin/www_bget?xcc:XCC4227) | minus | 343 |
| 186 | GGGATTGGGGAATCGGGATTGGGGATTC | plus | 28 | 4 | 5062192 | 5062165 | no | n.a. | fecA | [XCC4235](http://www.genome.jp/dbget-bin/www_bget?xcc:XCC4235) | minus | 4 | glpQ | [XCC4234](http://www.genome.jp/dbget-bin/www_bget?xcc:XCC4234) | minus | 350 |

S2 Table B: GGGAATC Repeats in Xanthomonas axonopodis pv. citri str. 306

Table shows the occurrence of GGGAATC repeat sequences in the *Xac* str. 306 genome (AE008923). Sequence of the repeat, its total length and the number of repeat units are stated. Mutations deviating from the consensus sequences are marked in red. G-tracts are underscored. The location on plus or minus strand as well as start and end point of the sequence on the genome is given. Participation in inverted repeat (inv rep) formation is stated with the number of the partnering repeat and the distance between the two repeats (dis inv reps). Other indicates occurrence of another G-rich sequence or another repeat type in place of the partnering GGGAATC repeat. The next upstream and downstream genes are listed, including locus tags, location on the plus or minus strand of the genome and the distance between the neighboring gene and the repeat (dis [nt]). Distances are negative in case a repeat overlaps with the ORF. [n.a. = not available].

| **#** | **sequence (5'-3')** | **strand** | **Length**  **[nt]** | **number units** | **start** | **end** | **inv rep** | **dis inv rep** | **upstream gene** | **locus tag** | **strand** | **dis [nt]** | **downstream gene** | **locus tag** | **strand** | **dis [nt]** |
| --- | --- | --- | --- | --- | --- | --- | --- | --- | --- | --- | --- | --- | --- | --- | --- | --- |
| 1 | GGGAATGGAGAATCGGGAATC | plus | 21 | 3 | 19357 | 19377 | 2 | 44 | hypothetical protein | [XAC0017](http://www.genome.jp/dbget-bin/www_bget?xac:XAC0017) | minus | 98 | hypothetical protein | [XAC0018](http://www.genome.jp/dbget-bin/www_bget?xac:XAC0018) | minus | 64 |
| 2 | GGGAATAGGGAATCGGGAATGGGGAATC | minus | 28 | 4 | 19449 | 19422 | 1 | 44 | hypothetical protein | [XAC0018](http://www.genome.jp/dbget-bin/www_bget?xac:XAC0018) | minus | -8 | hypothetical protein | [XAC0017](http://www.genome.jp/dbget-bin/www_bget?xac:XAC0017) | minus | 163 |
| 3 | GGCAATCGGGAATCGGGAATCGGGAATC | plus | 28 | 4 | 160596 | 160623 | no | n.a. | two-component system regulatory protein | [XAC0136](http://www.genome.jp/dbget-bin/www_bget?xac:XAC0136) | plus | 5 | hypothetical protein | [XAC0137](http://www.genome.jp/dbget-bin/www_bget?xac:XAC0137) | minus | 198 |
| 4 | GAGAATCGGGAATCGGGAATT | plus | 21 | 3 | 167597 | 167617 | other | 103 | trans-2-enoyl-CoA reductase | [XAC0141](http://www.genome.jp/dbget-bin/www_bget?xac:XAC0141) | plus | -2 | hypothetical protein | [XAC0142](http://www.genome.jp/dbget-bin/www_bget?xac:XAC0142) | plus | 98 |
| 5 | GTGAATCGGGAATTGGGAATC | minus | 21 | 3 | 168205 | 168185 | no | n.a. | kdgK | [XAC0143](http://www.genome.jp/dbget-bin/www_bget?xac:XAC0143) | minus | -4 | hypothetical protein | [XAC0142](http://www.genome.jp/dbget-bin/www_bget?xac:XAC0142) | plus | 142 |
| 6 | GGGATTCGGGACTCGGGAATC | plus | 21 | 3 | 386193 | 386213 | 7 | 103 | purU | [XAC0324](http://www.genome.jp/dbget-bin/www_bget?xac:XAC0324) | plus | 4 | smeR | [XAC0325](http://www.genome.jp/dbget-bin/www_bget?xac:XAC0325) | minus | 127 |
| 7 | GTGAATGGGGAATTGGGAATGGGGAATC | minus | 28 | 4 | 386344 | 386317 | 6 | 103 | smeR | [XAC0325](http://www.genome.jp/dbget-bin/www_bget?xac:XAC0325) | minus | -4 | purU | [XAC0324](http://www.genome.jp/dbget-bin/www_bget?xac:XAC0324) | plus | 128 |
| 8 | GAGAATCGGGAATCGGGAATCGGGAATCGGGAATCGGGAATCGGGAATC | plus | 49 | 7 | 565740 | 565788 | no | n.a. | lipid kinase | [XAC0475](http://www.genome.jp/dbget-bin/www_bget?xac:XAC0475) | minus | -2 | trpE | [XAC0476](http://www.genome.jp/dbget-bin/www_bget?xac:XAC0476) | plus | 521 |
| 9 | GTGAATCGGGAATCGGGAATC | minus | 21 | 3 | 614979 | 614959 | no | n.a. | PbsX family transcriptional regulator | [XAC0524](http://www.genome.jp/dbget-bin/www_bget?xac:XAC0524) | minus | -4 | hypothetical protein | [XAC0523](http://www.genome.jp/dbget-bin/www_bget?xac:XAC0523) | minus | 302 |
| 10 | GGGAATCGGGAGTGGAGAATGGGGAATC | minus | 28 | 4 | 621222 | 621249 | no | n.a. | bccP | [XAC0532](http://www.genome.jp/dbget-bin/www_bget?xac:XAC0532) | minus | 6 | hypothetical protein | [XAC0531](http://www.genome.jp/dbget-bin/www_bget?xac:XAC0531) | minus | -17 |
| 11 | GGGAATCGGGAGTTGGGAATCGGGAATG | plus | 28 | 4 | 632575 | 632602 | 12 | 61 | groES | [XAC0541](http://www.genome.jp/dbget-bin/www_bget?xac:XAC0541) | plus | 16 | groEL | [XAC0542](http://www.genome.jp/dbget-bin/www_bget?xac:XAC0542) | plus | 102 |
| 12 | GGGAATCGGGAGTGGAGAATGGGGAATC | minus | 28 | 4 | 632691 | 632664 | 64 | 61 | groEL | [XAC0542](http://www.genome.jp/dbget-bin/www_bget?xac:XAC0542) | plus | 13 | groES | [XAC0541](http://www.genome.jp/dbget-bin/www_bget?xac:XAC0541) | plus | 105 |
| 13 | GGGAATTGGGAATTGGGAATTGGGAATC | minus | 28 | 4 | 642213 | 642186 | no | n.a. | hypothetical protein | [XAC0546](http://www.genome.jp/dbget-bin/www_bget?xac:XAC0546) | plus | 49 | aroG | [XAC0545](http://www.genome.jp/dbget-bin/www_bget?xac:XAC0545) | plus | 711 |
| 14 | GTGAATCGGGAATCGGGAACG | minus | 21 | 3 | 701970 | 701950 | other | 45 | pheC | [XAC0598](http://www.genome.jp/dbget-bin/www_bget?xac:XAC0598) | minus | -4 | hypothetical protein | [XAC0597](http://www.genome.jp/dbget-bin/www_bget?xac:XAC0597) | plus | 71 |
| 15 | GTGAATCGGGAGTGGGGAATCGGGAATC | plus | 28 | 4 | 752397 | 752424 | 16 | 56 | hslV | [XAC0637](http://www.genome.jp/dbget-bin/www_bget?xac:XAC0637) | plus | -4 | hslU | [XAC0638](http://www.genome.jp/dbget-bin/www_bget?xac:XAC0638) | plus | 86 |
| 16 | GGCATTAGGGAATCGAGAATTGGGAAAGGGGAATC | minus | 35 | 5 | 762515 | 752481 | 15 | 56 | hslU | [XAC0638](http://www.genome.jp/dbget-bin/www_bget?xac:XAC0638) | plus | -5 | hslV | [XAC0637](http://www.genome.jp/dbget-bin/www_bget?xac:XAC0637) | plus | 80 |
| 17 | GGGAATGGGGAATCGGGAATGGGGAATC | plus | 28 | 4 | 773369 | 773396 | 18 | 27 | fepA | [XAC0653](http://www.genome.jp/dbget-bin/www_bget?xac:XAC0653) | plus | 4 | acoR | [XAC0654](http://www.genome.jp/dbget-bin/www_bget?xac:XAC0654) | minus | 2 |
| 18 | GGGAATCGGGAATCGGGAATCGGGAATCGGGAATG | minus | 35 | 5 | 773458 | 773424 | 17 | 27 | acoR | [XAC0654](http://www.genome.jp/dbget-bin/www_bget?xac:XAC0654) | minus | 3 | fepA | [XAC0653](http://www.genome.jp/dbget-bin/www_bget?xac:XAC0653) | plus | 59 |
| 19 | GGGAATCGGGAATCGGGAAAGGGGAATC | plus | 28 | 4 | 777914 | 777941 | 20 | 92 | mreB | [XAC0656](http://www.genome.jp/dbget-bin/www_bget?xac:XAC0656) | plus | 7 | mreC | [XAC0657](http://www.genome.jp/dbget-bin/www_bget?xac:XAC0657) | plus | 137 |
| 20 | GGGAATGGGGAGTCGGGAATGGGGAATCGGGGAGC | minus | 35 | 5 | 778068 | 778034 | 19 | 92 | mreC | [XAC0657](http://www.genome.jp/dbget-bin/www_bget?xac:XAC0657) | plus | 10 | mreB | [XAC0656](http://www.genome.jp/dbget-bin/www_bget?xac:XAC0656) | plus | 127 |
| 21 | TGGAATGGGGAATCGGGAATTGGGAATTGGGAATG | plus | 35 | 5 | 778930 | 778964 | no | n.a. | intragenic: mreC | [XAC0657](http://www.genome.jp/dbget-bin/www_bget?xac:XAC0657) | plus |  |  |  |  |  |
| 22 | GGGAATCGGGAATCGGGAATCGGGAATC | minus | 28 | 4 | 779402 | 779375 | no | n.a. | intragenic: mreC | [XAC0657](http://www.genome.jp/dbget-bin/www_bget?xac:XAC0657) | plus |  |  |  |  |  |
| 23 | GCGAATCGGGAATCGGGAATCGGGAATC | minus | 28 | 4 | 789413 | 789386 | no | n.a. | hypothetical protein | [XAC0665](http://www.genome.jp/dbget-bin/www_bget?xac:XAC0665) | minus | 2 | dacC | [XAC0664](http://www.genome.jp/dbget-bin/www_bget?xac:XAC0664) | plus | 90 |
| 24 | GGGAAATGGGGAGTCGGGAATGGGGAATC | plus | 29 | 4 | 891468 | 891496 | 25 | 40 | nusB | [XAC0751](http://www.genome.jp/dbget-bin/www_bget?xac:XAC0751) | plus | 7 | thiL | [XAC0752](http://www.genome.jp/dbget-bin/www_bget?xac:XAC0752) | plus | 75 |
| 25 | GGGAATCGGGAAAAGGGAATCGGGAATC | minus | 28 | 4 | 891564 | 891537 | 24 | 40 | thiL | [XAC0752](http://www.genome.jp/dbget-bin/www_bget?xac:XAC0752) | plus | 6 | nusB | [XAC0751](http://www.genome.jp/dbget-bin/www_bget?xac:XAC0751) | plus | 76 |
| 26 | GGGAATCGGGAATCGGGAATCGGGAATC | minus | 28 | 4 | 901252 | 901225 | other | 45 | kdpC | [XAC0758](http://www.genome.jp/dbget-bin/www_bget?xac:XAC0758) | plus | 23 | kdpD | [XAC0759](http://www.genome.jp/dbget-bin/www_bget?xac:XAC0759) | plus | 1 |
| 27 | GGGATTGGGGAATGGGGAATCGGGAATG | plus | 28 | 4 | 916138 | 916165 | 28 | 21 | intragenic: mraW | [XAC0772](http://www.genome.jp/dbget-bin/www_bget?xac:XAC0772) | plus |  |  |  |  |  |
| 28 | GGGAATGGGGAATCGGCAGTCGGGAATCGGGAAGC | minus | 35 | 5 | 916221 | 916187 | 27 | 21 | intragenic: mraW | [XAC0772](http://www.genome.jp/dbget-bin/www_bget?xac:XAC0772) | plus |  |  |  |  |  |
| 29 | GCGAATCGGGAATCGGGAATCGGGAATG | minus | 28 | 4 | 925126 | 925099 | other | 94 | intragenic: murG | [XAC0779](http://www.genome.jp/dbget-bin/www_bget?xac:XAC0779) | plus |  |  |  |  |  |
| 30 | GGGAATCGTGAATCGTGAATCGGGAATG | plus | 28 | 4 | 927538 | 927565 | 31 | 29 | ddl | [XAC0781](http://www.genome.jp/dbget-bin/www_bget?xac:XAC0781) | plus | 4 | ftsQ | [XAC0782](http://www.genome.jp/dbget-bin/www_bget?xac:XAC0782) | plus | 74 |
| 31 | GGGAATTGGGAGTCGGGAATAGGGAATC | minus | 28 | 4 | 927622 | 927595 | 30 | 29 | ftsQ | [XAC0782](http://www.genome.jp/dbget-bin/www_bget?xac:XAC0782) | plus | 17 | ddl | [XAC0781](http://www.genome.jp/dbget-bin/www_bget?xac:XAC0781) | plus | 61 |
| 32 | GGGAATCGGGAATG | plus | 14 | 2 | 928337 | 928350 | no | n.a. | intragenic: ftsQ | [XAC0782](http://www.genome.jp/dbget-bin/www_bget?xac:XAC0782) | plus |  |  |  |  |  |
| 33 | GGGAATCGGGATTCGAGAATT | plus | 21 | 3 | 929715 | 929735 | 34 | 125 | ftsA | [XAC0783](http://www.genome.jp/dbget-bin/www_bget?xac:XAC0783) | plus | 6 | ftsZ | [XAC0784](http://www.genome.jp/dbget-bin/www_bget?xac:XAC0784) | plus | 263 |
| 34 | GGGAATGGGAAGTTGGAAATGGGGAATC | minus | 28 | 4 | 929888 | 929861 | 33 | 125 | ftsZ | [XAC0784](http://www.genome.jp/dbget-bin/www_bget?xac:XAC0784) | plus | 110 | ftsA | [XAC0783](http://www.genome.jp/dbget-bin/www_bget?xac:XAC0783) | plus | 152 |
| 35 | GTGAATGGGGAATCGGGAATGGGGAATC | minus | 28 | 4 | 929976 | 929949 | no | n.a. | ftsZ | [XAC0784](http://www.genome.jp/dbget-bin/www_bget?xac:XAC0784) | plus | 22 | ftsA | [XAC0783](http://www.genome.jp/dbget-bin/www_bget?xac:XAC0783) | plus | 240 |
| 36 | GGGCATTGGGAATC | minus | 14 | 2 | 940480 | 940467 | no | n.a. | intragenic: hypothetical protein | [XAC0792](http://www.genome.jp/dbget-bin/www_bget?xac:XAC0792) | minus |  |  |  |  |  |
| 37 | GGGAATCGGGAATCGGGAATCGGAAATCGGAAATCGGAAATCGGAAATCGGAAATCGGAAATCGGAAATCGGAAATCGGAAAAG | minus | 84 | 12 | 1065898 | 1065815 | no | n.a. | oxyR | [XAC0905](http://www.genome.jp/dbget-bin/www_bget?xac:XAC0905) | minus | -1 | hypothetical protein | [XAC0904](http://www.genome.jp/dbget-bin/www_bget?xac:XAC0904) | minus | 172 |
| 38 | GGGAGACGGGAATC | minus | 14 | 2 | 1115901 | 1115888 | no | n.a. | tuf | [XAC0957](http://www.genome.jp/dbget-bin/www_bget?xac:XAC0957) | plus | 161 | ychF | [XAC0953](http://www.genome.jp/dbget-bin/www_bget?xac:XAC0953) | plus | 323 |
| 39 | GGGAATCGGGAATC | minus | 14 | 2 | 1121432 | 1121419 | no | n.a. | rpoB | [XAC0965](http://www.genome.jp/dbget-bin/www_bget?xac:XAC0965) | plus | 135 | rplL | [XAC0964](http://www.genome.jp/dbget-bin/www_bget?xac:XAC0964) | plus | 130 |
| 40 | GGGAATGGAGAATCGGGAATC | plus | 21 | 3 | 1197952 | 1197972 | 41 | 73 | gtrB | [XAC1038](http://www.genome.jp/dbget-bin/www_bget?xac:XAC1038) | minus | 84 | ppx | [XAC1039](http://www.genome.jp/dbget-bin/www_bget?xac:XAC1039) | minus | 118 |
| 41 | GGGAATCGGGAATCGGGAATCGGGAATA | minus | 28 | 4 | 1198073 | 1198046 | 40 | 73 | ppx | [XAC1039](http://www.genome.jp/dbget-bin/www_bget?xac:XAC1039) | minus | 17 | gtrB | [XAC1038](http://www.genome.jp/dbget-bin/www_bget?xac:XAC1038) | minus | 178 |
| 42 | GGGAATTGGGAATC | minus | 14 | 2 | 1208128 | 1208115 | no | n.a. | hypothetical protein | [XAC1047](http://www.genome.jp/dbget-bin/www_bget?xac:XAC1047) | minus | 3 | icd | [XAC1046](http://www.genome.jp/dbget-bin/www_bget?xac:XAC1046) | plus | 107 |
| 43 | GAGAATTGGGAATTGGGAATGGGGAATC | plus | 28 | 4 | 1262212 | 1262239 | 44 | 29 | hypothetical protein | [XAC1110](http://www.genome.jp/dbget-bin/www_bget?xac:XAC1110) | plus | 6 | recR | [XAC1111](http://www.genome.jp/dbget-bin/www_bget?xac:XAC1111) | plus | 63 |
| 44 | TGGAATCGGGAATCGGGAGTAGGGAATT | minus | 28 | 4 | 1262296 | 1262269 | 43 | 29 | recR | [XAC1111](http://www.genome.jp/dbget-bin/www_bget?xac:XAC1111) | plus | 6 | hypothetical protein | [XAC1110](http://www.genome.jp/dbget-bin/www_bget?xac:XAC1110) | plus | 63 |
| 45 | GGGAATAGGGAATCGGGATTGGGGAATC | minus | 28 | 4 | 1262995 | 1262968 | other | 44 | histidine triad-like protein | [XAC1112](http://www.genome.jp/dbget-bin/www_bget?xac:XAC1112) | plus | 5 | recR | [XAC1111](http://www.genome.jp/dbget-bin/www_bget?xac:XAC1111) | plus | 71 |
| 46 | GGGAATCGGGAATCGGGATTC | plus | 21 | 3 | 1349320 | 1349340 | no | n.a. | hypothetical protein | [XAC1183](http://www.genome.jp/dbget-bin/www_bget?xac:XAC1183) | plus | 19 | hypothetical protein | [XAC1184](http://www.genome.jp/dbget-bin/www_bget?xac:XAC1184) | minus | 117 |
| 47 | GGGAATCGGGAGTAGGGAATCGGGAATC | plus | 28 | 4 | 1426184 | 1426211 | 50 | 89 | rpmA | [XAC1249](http://www.genome.jp/dbget-bin/www_bget?xac:XAC1249) | plus | 94 | obgE, GTPase ObgE | [XAC1250](http://www.genome.jp/dbget-bin/www_bget?xac:XAC1250) | plus | 129 |
| 48 | GGGAATTGAGAATAGGGAATAGGGAATC | minus | 28 | 4 | 1426328 | 1426301 | 47 | 89 | obgE, GTPase ObgE | [XAC1250](http://www.genome.jp/dbget-bin/www_bget?xac:XAC1250) | plus | 12 | rpmA | [XAC1249](http://www.genome.jp/dbget-bin/www_bget?xac:XAC1249) | plus | 211 |
| 49 | GGGAACCGGGAATC | plus | 14 | 2 | 1434335 | 1434348 | no | n.a. | intragenic: lspA | [XAC1255](http://www.genome.jp/dbget-bin/www_bget?xac:XAC1255) | plus |  |  |  |  |  |
| 50 | CGGAATCGGGAATCGGGAATCGGGAATCGGGAATCGGGATTCGGGATTCGGGATTCGGGATTCGGGATTCGGGATTCGGGATTCGGGATTCGGGATTCGGGATTCGGGATTCGGGATTCGGGCAAT | minus | 126 | 18 | 1442084 | 1441959 | no | n.a. | radA | [XAC1263](http://www.genome.jp/dbget-bin/www_bget?xac:XAC1263) | plus | 72 | hypothetical protein | [XAC1262](http://www.genome.jp/dbget-bin/www_bget?xac:XAC1262) | plus | 135 |
| 51 | GGGATTGGGGATTGGCGAGTCGGGAATCGGGAATCGGGAATCGGGAATC | plus | 49 | 7 | 1477534 | 1477582 | no | n.a. | hypothetical protein | [XAC1288](http://www.genome.jp/dbget-bin/www_bget?xac:XAC1288) | minus | 25 | ffh | [XAC1289](http://www.genome.jp/dbget-bin/www_bget?xac:XAC1289) | plus | 167 |
| 52 | GGGAATCGGGAATCGGGAATCGGGAATCGGGAATCGGGAATC | minus | 42 | 6 | 1491857 | 1491898 | no | n.a. | mutS | [XAC1303](http://www.genome.jp/dbget-bin/www_bget?xac:XAC1303) | minus | 5 | hypothetical protein | [XAC1302](http://www.genome.jp/dbget-bin/www_bget?xac:XAC1302) | minus | 259 |
| 53 | GGGAATGGAGAATCGGGAATCGGGAATG | plus | 28 | 4 | 1527267 | 1527294 | 54 | 42 | mucD | [XAC1321](http://www.genome.jp/dbget-bin/www_bget?xac:XAC1321) | plus | 4 | lepA | [XAC1322](http://www.genome.jp/dbget-bin/www_bget?xac:XAC1322) | plus | 140 |
| 54 | GGGAATTGGGAATCGGGAGTGGGGAATC | minus | 28 | 4 | 1527364 | 1527337 | 53 | 42 | lepA | [XAC1322](http://www.genome.jp/dbget-bin/www_bget?xac:XAC1322) | plus | 70 | mucD | [XAC1321](http://www.genome.jp/dbget-bin/www_bget?xac:XAC1321) | plus | 74 |
| 55 | GGGAATGGTGAATCGGGAATCGGGAATC | plus | 28 | 4 | 1529243 | 1529270 | 56 | 28 | lepA | [XAC1322](http://www.genome.jp/dbget-bin/www_bget?xac:XAC1322) | plus | 2 | lepB | [XAC1323](http://www.genome.jp/dbget-bin/www_bget?xac:XAC1323) | plus | 73 |
| 56 | GGGAATCGAGAATCGCGAATGGGGAATA | minus | 28 | 4 | 1529326 | 1529326 | 55 | 28 | lepB | [XAC1323](http://www.genome.jp/dbget-bin/www_bget?xac:XAC1323) | plus | 17 | lepA | [XAC1322](http://www.genome.jp/dbget-bin/www_bget?xac:XAC1322) | plus | 58 |
| 57 | GTGAATCGGGAGTAGGCAGTAGGGAATC | plus | 28 | 4 | 1535687 | 1535714 | 58 | 27 | rumA | [XAC1329](http://www.genome.jp/dbget-bin/www_bget?xac:XAC1329) | plus | -5 | hypothetical protein | [XAC1330](http://www.genome.jp/dbget-bin/www_bget?xac:XAC1330) | plus | 62 |
| 58 | GGGAATCGGGAATCGGGAATCGGGAATCGGGAATG | minus | 35 | 5 | 1535776 | 1535742 | 57 | 27 | hypothetical protein | [XAC1330](http://www.genome.jp/dbget-bin/www_bget?xac:XAC1330) | plus | 0 | rumA | [XAC1329](http://www.genome.jp/dbget-bin/www_bget?xac:XAC1329) | plus | 51 |
| 59 | GGGATTGGGAGTTGGGAATCGGGAATC | minus | 27 | 4 | 1592373 | 1592347 | no | n.a. | metG | [XAC1386](http://www.genome.jp/dbget-bin/www_bget?xac:XAC1386) | minus | 1 | hypothetical protein | [XAC1385](http://www.genome.jp/dbget-bin/www_bget?xac:XAC1385) | minus | 278 |
| 60 | GGGAATAGGGAGTCGGGAATCGGGAATT | plus | 28 | 4 | 1594501 | 1594528 | no | n.a. | metG | [XAC1386](http://www.genome.jp/dbget-bin/www_bget?xac:XAC1386) | minus | 41 | hypothetical protein | [XAC1387](http://www.genome.jp/dbget-bin/www_bget?xac:XAC1387) | minus | 183 |
| 61 | GGGAATCGGGAGTCGGGAATC | plus | 21 | 3 | 1610940 | 1610960 | 62 | 41 | hypothetical protein | [XAC1397](http://www.genome.jp/dbget-bin/www_bget?xac:XAC1397) | minus | 40 | hypothetical protein | [XAC1398](http://www.genome.jp/dbget-bin/www_bget?xac:XAC1398) | plus | 145 |
| 62 | GTGAATCGGGAATTGGGAATCGGGCGAG | minus | 28 | 4 | 1611002 | 1611029 | 61 | 41 | hypothetical protein | [XAC1398](http://www.genome.jp/dbget-bin/www_bget?xac:XAC1398) | plus | 76 | hypothetical protein | [XAC1397](http://www.genome.jp/dbget-bin/www_bget?xac:XAC1397) | minus | 102 |
| 63 | CGGAATCGGGAATGGAGAATCGGGAATC | plus | 28 | 4 | 1617856 | 1617883 | 64 | 31 | accA | [XAC1405](http://www.genome.jp/dbget-bin/www_bget?xac:XAC1405) | minus | 73 | dnaE | [XAC1406](http://www.genome.jp/dbget-bin/www_bget?xac:XAC1406) | minus | 57 |
| 64 | GGGAATCGGGAATGGGGAATC | minus | 21 | 3 | 1617915 | 1617935 | 63 | 31 | dnaE | [XAC1406](http://www.genome.jp/dbget-bin/www_bget?xac:XAC1406) | minus | 5 | accA | [XAC1405](http://www.genome.jp/dbget-bin/www_bget?xac:XAC1405) | minus | 133 |
| 65 | GGGAATCGGGAATGGGGAATGGGGAATC | plus | 28 | 4 | 1622438 | 1622465 | 66 | 78 | intragenic: rnhB | [XAC1407](http://www.genome.jp/dbget-bin/www_bget?xac:XAC1407) | minus |  | lpxB | [XAC1408](http://www.genome.jp/dbget-bin/www_bget?xac:XAC1408) | minus | 59 |
| 66 | GCGAATCGGGAATGGAGAATCGGGAATC | minus | 28 | 4 | 1622544 | 1622571 | 65 | 78 | intragenic: lpxB | [XAC1408](http://www.genome.jp/dbget-bin/www_bget?xac:XAC1408) | minus |  | rnhB | [XAC1407](http://www.genome.jp/dbget-bin/www_bget?xac:XAC1407) | minus | 15 |
| 67 | GGGAATCGGGAGTGGGGAATCGGGAATC | plus | 28 | 4 | 1623731 | 1623758 | 68 | 53 | intragenic: lpxB | [XAC1408](http://www.genome.jp/dbget-bin/www_bget?xac:XAC1408) | minus |  | lpxA | [XAC1409](http://www.genome.jp/dbget-bin/www_bget?xac:XAC1409) | minus | 82 |
| 68 | GGGAATTGGGAATCGGGAGTCGGGAATC | minus | 28 | 4 | 1623839 | 1623812 | 67 | 53 | lpxA | [XAC1409](http://www.genome.jp/dbget-bin/www_bget?xac:XAC1409) | minus | 1 | lpxB | [XAC1408](http://www.genome.jp/dbget-bin/www_bget?xac:XAC1408) | minus | 0 |
| 69 | GGGAATCGGGATTGGGGAATCGGGAATC | minus | 28 | 4 | 1633876 | 1633849 | no | n.a. | pyrH | [XAC1419](http://www.genome.jp/dbget-bin/www_bget?xac:XAC1419) | minus | 4 | frr | [XAC1418](http://www.genome.jp/dbget-bin/www_bget?xac:XAC1418) | minus | 152 |
| 70 | GGGAATCGGGAATCGGGAATCGGGAATCGGGAATC | plus | 35 | 5 | 1638643 | 1638677 | other | 62 | rpsB | [XAC1422](http://www.genome.jp/dbget-bin/www_bget?xac:XAC1422) | minus | 285 | pili | [XAC1423](http://www.genome.jp/dbget-bin/www_bget?xac:XAC1423) | minus | 74 |
| 71 | GGGAATCGGGAATCGGGAATTGGGAATCGGGAATG | plus | 35 | 5 | 1684578 | 1684612 | 72 | 80 | fpr | [XAC1458](http://www.genome.jp/dbget-bin/www_bget?xac:XAC1458) | minus | 75 | msbA | [XAC1459](http://www.genome.jp/dbget-bin/www_bget?xac:XAC1459) | minus | 105 |
| 72 | GGGAATGGGGAATGGGGAATT | minus | 21 | 3 | 1684713 | 1684693 | 71 | 80 | msbA | [XAC1459](http://www.genome.jp/dbget-bin/www_bget?xac:XAC1459) | minus | 4 | fpr | [XAC1458](http://www.genome.jp/dbget-bin/www_bget?xac:XAC1458) | minus | 190 |
| 73 | GTGATTCGGGAAGCGGCAGTGGGGAGTCGGGAATC | plus | 35 | 5 | 1748649 | 1748683 | 74 | 83 | serine | [XAC1512](http://www.genome.jp/dbget-bin/www_bget?xac:XAC1512) | plus | -4 | smpB | [XAC1513](http://www.genome.jp/dbget-bin/www_bget?xac:XAC1513) | minus | 107 |
| 74 | GTGAATTGGGAATCGGGAATCGGGAATC | minus | 28 | 4 | 1748767 | 1748794 | 73 | 83 | smpB | [XAC1513](http://www.genome.jp/dbget-bin/www_bget?xac:XAC1513) | minus | -4 | serine | [XAC1512](http://www.genome.jp/dbget-bin/www_bget?xac:XAC1512) | plus | 114 |
| 75 | CGGAATCGGGAGTGGGGAATCGGGAATG | plus | 28 | 4 | 1770432 | 1770459 | 76 | 89 | hypothetical protein | [XAC1532](http://www.genome.jp/dbget-bin/www_bget?xac:XAC1532) | minus | 6 | ldp | [XAC1533](http://www.genome.jp/dbget-bin/www_bget?xac:XAC1533) | minus | 120 |
| 76 | GGGAATCGGGAGTGGGGAATCGGGAATC | minus | 28 | 4 | 1770576 | 1770549 | 75 | 89 | ldp | [XAC1533](http://www.genome.jp/dbget-bin/www_bget?xac:XAC1533) | minus | 3 | hypothetical protein | [XAC1532](http://www.genome.jp/dbget-bin/www_bget?xac:XAC1532) | minus | 123 |
| 77 | GGGAATGGGGAATAGGGAATGGGGAATC | plus | 28 | 4 | 1772030 | 1772057 | 78 | 72 | ldp | [XAC1533](http://www.genome.jp/dbget-bin/www_bget?xac:XAC1533) | minus | 13 | sucB | [XAC1534](http://www.genome.jp/dbget-bin/www_bget?xac:XAC1534) | minus | 105 |
| 78 | GGGAATCGGGAATGGAGATACGGGAATG | minus | 28 | 4 | 1772157 | 1772130 | 77 | 72 | sucB | [XAC1534](http://www.genome.jp/dbget-bin/www_bget?xac:XAC1534) | minus | 5 | ldp | [XAC1533](http://www.genome.jp/dbget-bin/www_bget?xac:XAC1533) | minus | 113 |
| 79 | GGGAATCGGGATTCGGGAGTC | plus | 21 | 3 | 1816998 | 1817018 | 80 | 112 | hypothetical protein | [XAC1570](http://www.genome.jp/dbget-bin/www_bget?xac:XAC1570) | plus | 5 | rnt | [XAC1571](http://www.genome.jp/dbget-bin/www_bget?xac:XAC1571) | minus | 144 |
| 80 | GGGAATCGGGAATCGGGAATCGGGAATC | minus | 28 | 4 | 1817158 | 1817131 | 79 | 112 | rnt | [XAC1571](http://www.genome.jp/dbget-bin/www_bget?xac:XAC1571) | minus | 4 | hypothetical protein | [XAC1570](http://www.genome.jp/dbget-bin/www_bget?xac:XAC1570) | plus | 138 |
| 81 | GGGATTCGGGAATCGGGATTTGGGAATC | minus | 28 | 4 | 1866117 | 1866090 | no | n.a. | rplI | [XAC1622](http://www.genome.jp/dbget-bin/www_bget?xac:XAC1622) | plus | 65 | rpsR | [XAC1621](http://www.genome.jp/dbget-bin/www_bget?xac:XAC1621) | plus | 94 |
| 82 | CTGAATCGGGAATCGGGAGAG | minus | 21 | 3 | 1988866 | 1988846 | other | 98 | hypothetical protein | [XAC1729](http://www.genome.jp/dbget-bin/www_bget?xac:XAC1729) | minus | -4 | nlpD | [XAC1728](http://www.genome.jp/dbget-bin/www_bget?xac:XAC1728) | plus | 129 |
| 83 | GGGATTGGGGAATCGGGATTCGGGATTCGGGATTC | minus | 35 | 5 | 2140045 | 2140011 | other | 106 | serA | [XAC1844](http://www.genome.jp/dbget-bin/www_bget?xac:XAC1844) | minus | 2 | hypothetical protein | [XAC1843](http://www.genome.jp/dbget-bin/www_bget?xac:XAC1843) | plus | -8 |
| 84 | GGGAATCGGGAGTAGGGAATGGGGAATC | plus | 28 | 4 | 2146282 | 2146309 | 85 | 42 | yeiP | [XAC1849](http://www.genome.jp/dbget-bin/www_bget?xac:XAC1849) | minus | 0 | hadH2 | [XAC1850](http://www.genome.jp/dbget-bin/www_bget?xac:XAC1850) | minus | 72 |
| 85 | GGGATTGGGGAATCGGGAATGGGGAATC | minus | 28 | 4 | 2146379 | 2146352 | 84 | 42 | hadH2 | [XAC1850](http://www.genome.jp/dbget-bin/www_bget?xac:XAC1850) | minus | 2 | yeiP | [XAC1849](http://www.genome.jp/dbget-bin/www_bget?xac:XAC1849) | minus | 70 |
| 86 | GGGAATCGGGAATCGGGAATCGGGAATC | minus | 28 | 4 | 2245962 | 2245935 | no | n.a. | intragenic: hypothetical protein | [XAC1919](http://www.genome.jp/dbget-bin/www_bget?xac:XAC1919) | plus |  |  |  |  |  |
| 87 | GGGATTCGGGATTCGGGATTCGGGATTCGGGATTCGGGATTGGGATAATCGGGAATCGGGAATCGGGAATC | minus | 71 | 10 | 2255458 | 2255388 | no | n.a. | cheA | [XAC1930](http://www.genome.jp/dbget-bin/www_bget?xac:XAC1930) | minus | 5 | ISxac1 | [XAC1929](http://www.genome.jp/dbget-bin/www_bget?xac:XAC1929) | minus | 276 |
| 88 | GGGAATCGGGATTGGGCATGGGGGAATC | plus | 28 | 4 | 2265446 | 2265473 | no | n.a. | diguanylate | [XAC1938](http://www.genome.jp/dbget-bin/www_bget?xac:XAC1938) | minus | 57 | flhB | [XAC1937](http://www.genome.jp/dbget-bin/www_bget?xac:XAC1937) | minus | 59 |
| 89 | GGGAATCGGGAATGGAGAATCGGGAATC | plus | 28 | 4 | 2315785 | 2315812 | 90 | 73 | flgF | [XAC1982](http://www.genome.jp/dbget-bin/www_bget?xac:XAC1982) | minus | 8 | flgE | [XAC1983](http://www.genome.jp/dbget-bin/www_bget?xac:XAC1983) | minus | 106 |
| 90 | GGGCATGGGGAATGGGGAGTAGGGAATC | minus | 28 | 4 | 2315913 | 2315886 | 89 | 73 | flgE | [XAC1983](http://www.genome.jp/dbget-bin/www_bget?xac:XAC1983) | minus | 5 | flgF | [XAC1982](http://www.genome.jp/dbget-bin/www_bget?xac:XAC1982) | minus | 109 |
| 91 | GGGAATGGGGAATGGGGAATC | plus | 21 | 3 | 2320019 | 2320039 | 92 | 126 | cheV | [XAC1987](http://www.genome.jp/dbget-bin/www_bget?xac:XAC1987) | minus | 65 | flgA | [XAC1988](http://www.genome.jp/dbget-bin/www_bget?xac:XAC1988) | plus | 284 |
| 92 | GGGAATCGGGAATCGGGAATCGGGAATCGGGAATCGGGAATCGGGAATCGGGAAGCGGGAATCGGGAAGCGGGAATCGGGAAGCGGGAAGCGGGAAGCGGGAAGC | minus | 105 | 15 | 2320270 | 2320166 | 91 | 126 | flgA | [XAC1988](http://www.genome.jp/dbget-bin/www_bget?xac:XAC1988) | plus | 53 | cheV | [XAC1987](http://www.genome.jp/dbget-bin/www_bget?xac:XAC1987) | minus | 212 |
| 93 | GGGAATGGGGAGTTGGGAAATGGGGAATCGGGAATC | plus | 36 | 5 | 2323100 | 2323135 | 94 | 74 | histidine | [XAC1991](http://www.genome.jp/dbget-bin/www_bget?xac:XAC1991) | plus | 12 | c-di-GMP phosphodiesterase A | [XAC1992](http://www.genome.jp/dbget-bin/www_bget?xac:XAC1992) | minus | 93 |
| 94 | GGGAATAGGGAATCGGGATTGGGGAATC | minus | 28 | 4 | 2323237 | 2323210 | 93 | 74 | c-di-GMP phosphodiesterase A | [XAC1992](http://www.genome.jp/dbget-bin/www_bget?xac:XAC1992) | minus | -8 | histidine | [XAC1991](http://www.genome.jp/dbget-bin/www_bget?xac:XAC1991) | plus | 122 |
| 95 | GGGAATGGGGAGAGGGGAATCGGGAATC | plus | 28 | 4 | 2355081 | 2355108 | 96 | 83 | fadA | [XAC2012](http://www.genome.jp/dbget-bin/www_bget?xac:XAC2012) | minus | 19 | fadB | [XAC2013](http://www.genome.jp/dbget-bin/www_bget?xac:XAC2013) | minus | 109 |
| 96 | GAGAATCGGGAGCGGGGAATCGGGAATG | minus | 28 | 4 | 2355219 | 2355192 | 95 | 83 | fadB | [XAC2013](http://www.genome.jp/dbget-bin/www_bget?xac:XAC2013) | minus | -28 | fadA | [XAC2012](http://www.genome.jp/dbget-bin/www_bget?xac:XAC2012) | minus | 130 |
| 97 | GGGAATCGAGAATCGGGATGAGGGAATC | plus | 28 | 4 | 2677017 | 2677044 | 98 | 56 | guaA | [XAC2287](http://www.genome.jp/dbget-bin/www_bget?xac:XAC2287) | minus | 8 | guaB | [XAC2288](http://www.genome.jp/dbget-bin/www_bget?xac:XAC2288) | minus | 78 |
| 98 | GGGTAACCGGGAATGGGGAATCGGGAATC | minus | 29 | 4 | 2677128 | 2677100 | 97 | 56 | guaB | [XAC2288](http://www.genome.jp/dbget-bin/www_bget?xac:XAC2288) | minus | -6 | guaA | [XAC2287](http://www.genome.jp/dbget-bin/www_bget?xac:XAC2287) | minus | 92 |
| 99 | GGGAATGGAGAATCGGGAATAGGGAATC | plus | 28 | 4 | 2716217 | 2716244 | 100 | 76 | hydrolase | [XAC2321](http://www.genome.jp/dbget-bin/www_bget?xac:XAC2321) | minus | 10 | ybdL | [XAC2322](http://www.genome.jp/dbget-bin/www_bget?xac:XAC2322) | minus | 103 |
| 100 | GGGAATCGGGATTCGGGAATC | minus | 21 | 3 | 2716341 | 2716321 | 99 | 76 | ybdL | [XAC2322](http://www.genome.jp/dbget-bin/www_bget?xac:XAC2322) | minus | 6 | hydrolase | [XAC2321](http://www.genome.jp/dbget-bin/www_bget?xac:XAC2321) | minus | 54 |
| 101 | GGGAATGGGGAATC | minus | 14 | 2 | 2733563 | 2733550 | no | n.a. | cynX | [XAC2340](http://www.genome.jp/dbget-bin/www_bget?xac:XAC2340) | plus | 183 | hypothetical protein | [XAC2338](http://www.genome.jp/dbget-bin/www_bget?xac:XAC2338) | plus | 23 |
| 102 | GAGAATCGGGAGTGGGGAATCGGGAATG | plus | 28 | 4 | 2738510 | 2738537 | other | 69 | proA | [XAC2342](http://www.genome.jp/dbget-bin/www_bget?xac:XAC2342) | minus | 2 | proB | [XAC2343](http://www.genome.jp/dbget-bin/www_bget?xac:XAC2343) | minus | 99 |
| 103 | CGGAATCGGGAATCGGGAATAGAGAATC | minus | 28 | 4 | 2747411 | 2747384 | other | 56 | argF | [XAC2352](http://www.genome.jp/dbget-bin/www_bget?xac:XAC2352) | minus | -22 | argG | [XAC2351](http://www.genome.jp/dbget-bin/www_bget?xac:XAC2351) | minus | 43 |
| 104 | GGGAATCGGGAATGGTGAATCGGGAATG | plus | 28 | 4 | 2784666 | 2784693 | 105 | 19 | uup | [XAC2389](http://www.genome.jp/dbget-bin/www_bget?xac:XAC2389) | minus | 13 | dbpA | [XAC2390](http://www.genome.jp/dbget-bin/www_bget?xac:XAC2390) | plus | 60 |
| 105 | GGGAATCGGGAGTGGGGAATCGGGAATC | minus | 28 | 4 | 2784740 | 2784713 | 104 | 19 | dbpA | [XAC2390](http://www.genome.jp/dbget-bin/www_bget?xac:XAC2390) | plus | 60 | uup | [XAC2389](http://www.genome.jp/dbget-bin/www_bget?xac:XAC2389) | minus | 13 |
| 106 | GAGAGTTGGGATAGTGAATCGGGAATC | minus | 14 | 2 | 2873398 | 2873385 | no | n.a. | gabD | [XAC2469](http://www.genome.jp/dbget-bin/www_bget?xac:XAC2469) | minus | -2 | corA | [XAC2468](http://www.genome.jp/dbget-bin/www_bget?xac:XAC2468) | plus | 129 |
| 107 | GGGAATGGGGAGTGGGGAGTGGGGAATCGGGAATG | plus | 35 | 5 | 2943397 | 2943431 | 108 | 48 | tgt | [XAC2513](http://www.genome.jp/dbget-bin/www_bget?xac:XAC2513) | minus | 9 | queA | [XAC2514](http://www.genome.jp/dbget-bin/www_bget?xac:XAC2514) | minus | 48 |
| 108 | GGGAATCGGGACTCGGGAATGGGGAATC | minus | 28 | 4 | 2943507 | 2943480 | 107 | 48 | intragenic: queA | [XAC2514](http://www.genome.jp/dbget-bin/www_bget?xac:XAC2514) | minus |  | tgt | [XAC2513](http://www.genome.jp/dbget-bin/www_bget?xac:XAC2513) | minus | 92 |
| 109 | GGGAATCGAGAATTGGGACTGGGGAATC | plus | 28 | 4 | 2944593 | 2944620 | 110 | 20 | queA | [XAC2514](http://www.genome.jp/dbget-bin/www_bget?xac:XAC2514) | minus | 46 | asnC | [XAC2515](http://www.genome.jp/dbget-bin/www_bget?xac:XAC2515) | minus | 44 |
| 110 | GTGAATGGAGAATCGGGAATAGGGAATC | minus | 28 | 4 | 2944668 | 2944641 | 109 | 20 | asnC | [XAC2515](http://www.genome.jp/dbget-bin/www_bget?xac:XAC2515) | minus | -4 | queA | [XAC2514](http://www.genome.jp/dbget-bin/www_bget?xac:XAC2514) | minus | 94 |
| 111 | GAGAATCGGGAATGGGGAATGGGGAATC | plus | 28 | 4 | 3051091 | 3051118 | 112 | 57 | pheT | [XAC2589](http://www.genome.jp/dbget-bin/www_bget?xac:XAC2589) | minus | 8 | pheS | [XAC2590](http://www.genome.jp/dbget-bin/www_bget?xac:XAC2590) | minus | 89 |
| 112 | GGGAATCAGGAGTTGGGAATCGGGAATC | minus | 28 | 4 | 3051203 | 3051176 | 111 | 57 | pheS | [XAC2590](http://www.genome.jp/dbget-bin/www_bget?xac:XAC2590) | minus | 4 | pheT | [XAC2589](http://www.genome.jp/dbget-bin/www_bget?xac:XAC2589) | minus | 93 |
| 113 | GGGAATCGGGAGTCGGGATTT | minus | 21 | 3 | 3240087 | 3240067 | other | 98 | tldD | [XAC2767](http://www.genome.jp/dbget-bin/www_bget?xac:XAC2767) | minus | 93 | hypothetical protein | [XAC2766](http://www.genome.jp/dbget-bin/www_bget?xac:XAC2766) | plus | 55 |
| 114 | GGGAATCGGGAATCGGGATAGGGGAATC | minus | 28 | 4 | 3240174 | 3240147 | no | n.a. | tldD | [XAC2767](http://www.genome.jp/dbget-bin/www_bget?xac:XAC2767) | minus | 6 | hypothetical protein | [XAC2766](http://www.genome.jp/dbget-bin/www_bget?xac:XAC2766) | plus | 135 |
| 115 | GGGAATGGGGAATGGGGAATAGGGAATC | plus | 28 | 4 | 3246423 | 3246450 | other | 101 | hypothetical protein | [XAC2769](http://www.genome.jp/dbget-bin/www_bget?xac:XAC2769) | minus | 8 | rng | [XAC2770](http://www.genome.jp/dbget-bin/www_bget?xac:XAC2770) | minus | 133 |
| 116 | GGGAATCGGGATTCGGGATTCGGGAATC | plus | 28 | 4 | 3257474 | 3257501 | other | 124 | intragenic: nadD | [XAC2778](http://www.genome.jp/dbget-bin/www_bget?xac:XAC2778) | plus |  |  |  |  |  |
| 117 | GGGAATCGGGAATC | plus | 14 | 2 | 3270728 | 3270741 | no | n.a. | peptidyl-prolyl cis-trans isomeras | [XAC2789](http://www.genome.jp/dbget-bin/www_bget?xac:XAC2789) | plus | 3 | hypothetical protein | [XAC2790](http://www.genome.jp/dbget-bin/www_bget?xac:XAC2790) | minus | 50 |
| 118 | CGGAATCGGGAATC | plus | 14 | 2 | 3423999 | 3424012 | no | n.a. | osmC | [XAC2915](http://www.genome.jp/dbget-bin/www_bget?xac:XAC2915) | minus | 62 | pyrB | [XAC2916](http://www.genome.jp/dbget-bin/www_bget?xac:XAC2916) | minus | 221 |
| 119 | GGGAATGGGGAATCGAGAAACGGGAATT | plus | 28 | 4 | 3430838 | 3430865 | 120 | 44 | pilU | [XAC2923](http://www.genome.jp/dbget-bin/www_bget?xac:XAC2923) | minus | 19 | pilT | [XAC2924](http://www.genome.jp/dbget-bin/www_bget?xac:XAC2924) | minus | 75 |
| 120 | GGGAATAGCGAATCGGGAATGGGGAATC | minus | 28 | 4 | 3430937 | 3430910 | 119 | 44 | pilT | [XAC2924](http://www.genome.jp/dbget-bin/www_bget?xac:XAC2924) | minus | 3 | pilU | [XAC2923](http://www.genome.jp/dbget-bin/www_bget?xac:XAC2923) | minus | 91 |
| 121 | GGGAATCGGGACGC | minus | 14 | 2 | 3434097 | 3434084 | no | n.a. | hup | [XAC2927](http://www.genome.jp/dbget-bin/www_bget?xac:XAC2927) | plus | 441 | proC | [XAC2926](http://www.genome.jp/dbget-bin/www_bget?xac:XAC2926) | plus | 103 |
| 122 | GGGAATCGGGAATCGGGAATGGAGAATC | plus | 28 | 4 | 3444119 | 3444146 | 123 | 69 | nifS | [XAC2938](http://www.genome.jp/dbget-bin/www_bget?xac:XAC2938) | plus | 1 | acetyltransferase | [XAC2939](http://www.genome.jp/dbget-bin/www_bget?xac:XAC2939) | plus | 112 |
| 123 | GGGAATGGGGAATGCGGAATGGGGGAAG | minus | 28 | 4 | 3444243 | 3444216 | 122 | 69 | acetyltransferase | [XAC2939](http://www.genome.jp/dbget-bin/www_bget?xac:XAC2939) | plus | 15 | nifS | [XAC2938](http://www.genome.jp/dbget-bin/www_bget?xac:XAC2938) | plus | 98 |
| 124 | GGGAATCGGGATGC | minus | 14 | 2 | 3470439 | 3470426 | no | n.a. | hypothetical protein | [XAC2966](http://www.genome.jp/dbget-bin/www_bget?xac:XAC2966) | minus | 47 | murA | [XAC2965](http://www.genome.jp/dbget-bin/www_bget?xac:XAC2965) | minus | 181 |
| 125 | GGGAATCGGGAATCGGGAATCGGGAATCGGGAATCGGGAATC | plus | 42 | 6 | 3659207 | 3659248 | no | n.a. | hypothetical protein | [XAC3112](http://www.genome.jp/dbget-bin/www_bget?xac:XAC3112) | minus | 190 | relA | [XAC3113](http://www.genome.jp/dbget-bin/www_bget?xac:XAC3113) | minus | 77 |
| 126 | GGGAATGGGGAATCGGGAATG | plus | 21 | 3 | 3700791 | 3700811 | 127 | 42 | ruvA | [XAC3149](http://www.genome.jp/dbget-bin/www_bget?xac:XAC3149) | mins | 22 | ruvC | [XAC3150](http://www.genome.jp/dbget-bin/www_bget?xac:XAC3150) | minus | 75 |
| 127 | GAGAATCGGGAATCGGCAATCGGGAATCGGGAATC | minus | 35 | 5 | 3700888 | 3700854 | 126 | 42 | ruvC | [XAC3150](http://www.genome.jp/dbget-bin/www_bget?xac:XAC3150) | minus | -2 | ruvA | [XAC3149](http://www.genome.jp/dbget-bin/www_bget?xac:XAC3149) | minus | 85 |
| 128 | GGGAATGGGAAGTCGGGAATCGGGAATG | plus | 28 | 4 | 3701421 | 3701448 | 129 | 59 | ruvC | [XAC3150](http://www.genome.jp/dbget-bin/www_bget?xac:XAC3150) | minus | 9 | hypothetical protein | [XAC3151](http://www.genome.jp/dbget-bin/www_bget?xac:XAC3151) | minus | 92 |
| 129 | GGGAATCGGGAATGGAGAATCGGGAATC | minus | 28 | 4 | 3701535 | 3701508 | 128 | 59 | hypothetical protein | [XAC3151](http://www.genome.jp/dbget-bin/www_bget?xac:XAC3151) | minus | 5 | ruvC | [XAC3150](http://www.genome.jp/dbget-bin/www_bget?xac:XAC3150) | minus | 96 |
| 130 | GGGAATGGGGAATGGGGAATCGGGAATT | plus | 28 | 4 | 3703666 | 3703693 | no | n.a. | acetyltransferase | [XAC3153](http://www.genome.jp/dbget-bin/www_bget?xac:XAC3153) | minus | 61 | aspS | [XAC3154](http://www.genome.jp/dbget-bin/www_bget?xac:XAC3154) | minus | 333 |
| 131 | GGGAGTCGGGAGTGGGGAATCGGGAATC | plus | 28 | 4 | 3816118 | 3816145 | 132 | 202 | pilR | [XAC3238](http://www.genome.jp/dbget-bin/www_bget?xac:XAC3238) | plus | 7 | pilB | [XAC3239](http://www.genome.jp/dbget-bin/www_bget?xac:XAC3239) | minus | 1099 |
| 132 | GGGAATCGGGGAGTCGGATCAGGGAATC | minus | 28 | 4 | 3816375 | 3816348 | 131 | 202 | pilB | [XAC3239](http://www.genome.jp/dbget-bin/www_bget?xac:XAC3239) | minus | 869 | pilR | [XAC3238](http://www.genome.jp/dbget-bin/www_bget?xac:XAC3238) | plus | 237 |
| 133 | GGGATTCGGGAATCGGGAATCGGGAATC | plus | 28 | 4 | 3881891 | 3881918 | other | 63 | intragenic: trmB | [XAC3303](http://www.genome.jp/dbget-bin/www_bget?xac:XAC3303) | plus |  | sac1 | [XAC3304](http://www.genome.jp/dbget-bin/www_bget?xac:XAC3304) | plus | 99 |
| 134 | GGGAATCGGGAATCGGGAATCGGGAATC | plus | 28 | 4 | 3921708 | 3921735 | 135 | 48 | intragenic: nodQ | [XAC3328](http://www.genome.jp/dbget-bin/www_bget?xac:XAC3328) | minus |  | cysD | [XAC3329](http://www.genome.jp/dbget-bin/www_bget?xac:XAC3329) | minus | 82 |
| 135 | GCGAATGGGGAATCGGGAATGGAGAATC | minus | 28 | 4 | 3921811 | 3921784 | 134 | 48 | cysD | [XAC3329](http://www.genome.jp/dbget-bin/www_bget?xac:XAC3329) | minus | 7 | intragenic: nodQ | [XAC3328](http://www.genome.jp/dbget-bin/www_bget?xac:XAC3328) | minus |  |
| 136 | GGGAATCGCGAGTGGGGAATCGGGAATG | plus | 28 | 4 | 3924759 | 3924786 | 137 | 48 | cysJ | [XAC3330](http://www.genome.jp/dbget-bin/www_bget?xac:XAC3330) | plus | 4 | cysI | [XAC3331](http://www.genome.jp/dbget-bin/www_bget?xac:XAC3331) | plus | 76 |
| 137 | GAGAATCGAGAATCGGGAATGGGGAATC | minus | 28 | 4 | 3924862 | 3924835 | 136 | 48 | cysI | [XAC3331](http://www.genome.jp/dbget-bin/www_bget?xac:XAC3331) | plus | 0 | cysJ | [XAC3330](http://www.genome.jp/dbget-bin/www_bget?xac:XAC3330) | plus | 80 |
| 138 | GGGAATCGGGAATC | plus | 14 | 2 | 3940497 | 3940510 | no | n.a. | fructose-biphosphate aldolase | [XAC3344](http://www.genome.jp/dbget-bin/www_bget?xac:XAC3344) | minus | 128 | pykA | [XAC3345](http://www.genome.jp/dbget-bin/www_bget?xac:XAC3345) | minus | 474 |
| 139 | GGGATTCGGGATTC | plus | 14 | 2 | 3951002 | 3951015 | no | n.a. | gapA | [XAC3352](http://www.genome.jp/dbget-bin/www_bget?xac:XAC3352) | minus | 101 | hypothetical protein | [XAC3353](http://www.genome.jp/dbget-bin/www_bget?xac:XAC3353) | plus | 334 |
| 140 | GGGAATCGGGAATGGAGAGTC | plus | 21 | 3 | 3975090 | 3975110 | other | 107 | tktA | [XAC3372](http://www.genome.jp/dbget-bin/www_bget?xac:XAC3372) | minus | 70 | gltP | [XAC3373](http://www.genome.jp/dbget-bin/www_bget?xac:XAC3373) | minus | 127 |
| 141 | GGGATTCGGGAATCGGGATTGGGGAATC | plus | 28 | 4 | 3993009 | 3993036 | no | n.a. | mrcA | [XAC3386](http://www.genome.jp/dbget-bin/www_bget?xac:XAC3386) | plus | 6 | hypothetical protein | [XAC3387](http://www.genome.jp/dbget-bin/www_bget?xac:XAC3387) | plus | 335 |
| 142 | GGGAATCGGATTGGGGAATC | minus | 20 | 3 | 3997171 | 3997153 | other | 76 | recG | [XAC3391](http://www.genome.jp/dbget-bin/www_bget?xac:XAC3391) | minus | 5 | inosine-uridine preferring nucleoside hydrolase | [XAC3390](http://www.genome.jp/dbget-bin/www_bget?xac:XAC3390) | minus | 104 |
| 143 | GGGAATAGGCAATCGGGAATGGGGAATC | minus | 28 | 4 | 4003396 | 4003369 | other | 52 | hypothetical protein | [XAC3396](http://www.genome.jp/dbget-bin/www_bget?xac:XAC3396) | minus | 2 | gmk | [XAC3395](http://www.genome.jp/dbget-bin/www_bget?xac:XAC3395) | minus | 85 |
| 144 | GGGAATCGGGAATGGGGAGTTGGGAATC | plus | 28 | 4 | 4025924 | 4025951 | no | n.a. | hemL | [XAC3420](http://www.genome.jp/dbget-bin/www_bget?xac:XAC3420) | plus | 6 | acoK | [XAC3421](http://www.genome.jp/dbget-bin/www_bget?xac:XAC3421) | plus | 497 |
| 145 | GGGAATCGGGAATCGGGAATCGGGAATG | plus | 28 | 4 | 4040393 | 4040420 | 146 | 65 | short chain dehydrogenase | [XAC3431](http://www.genome.jp/dbget-bin/www_bget?xac:XAC3431) | minus | 0 | serine/threonine protein kinase | [XAC3432](http://www.genome.jp/dbget-bin/www_bget?xac:XAC3432) | minus | 99 |
| 146 | GGGAATTGGGAATCGGGAATGGGGAATC | minus | 28 | 4 | 4040513 | 4040486 | 145 | 65 | serine/threonine protein kinase | [XAC3432](http://www.genome.jp/dbget-bin/www_bget?xac:XAC3432) | minus | 6 | short chain dehydrogenase | [XAC3431](http://www.genome.jp/dbget-bin/www_bget?xac:XAC3431) | minus | 93 |
| 147 | GGGAATCGAGAGTCGGGAATAGAGAATG | minus | 28 | 4 | 4080207 | 4080180 | other | 28 | leuC | [XAC3458](http://www.genome.jp/dbget-bin/www_bget?xac:XAC3458) | minus | -22 | leuD | [XAC3457](http://www.genome.jp/dbget-bin/www_bget?xac:XAC3457) | minus | 65 |
| 148 | GGGAATCGTGAATAGGGAATGGGGAATC | plus | 28 | 4 | 4116663 | 4116690 | 149 | 91 | tctD | [XAC3483](http://www.genome.jp/dbget-bin/www_bget?xac:XAC3483) | minus | 6 | oprO | [XAC3484](http://www.genome.jp/dbget-bin/www_bget?xac:XAC3484) | plus | 229 |
| 149 | GGGAAGGAGAATCGGGAATGGGGAATC | minus | 27 | 4 | 4116808 | 4116782 | 148 | 91 | oprO | [XAC3484](http://www.genome.jp/dbget-bin/www_bget?xac:XAC3484) | plus | 111 | tctD | [XAC3483](http://www.genome.jp/dbget-bin/www_bget?xac:XAC3483) | minus | 125 |
| 150 | GGGAATGGGGAATCGGGAGTGGGGAATC | plus | 28 | 4 | 4119677 | 4119704 | 151 | 83 | citM | [XAC3485](http://www.genome.jp/dbget-bin/www_bget?xac:XAC3485) | plus | 8 | fabG | [XAC3486](http://www.genome.jp/dbget-bin/www_bget?xac:XAC3486) | plus | 135 |
| 151 | GGGAATGGGGAATCGGGACTGGGGAATC | minus | 28 | 4 | 4119815 | 4119788 | 150 | 83 | fabG | [XAC3486](http://www.genome.jp/dbget-bin/www_bget?xac:XAC3486) | plus | 24 | citM | [XAC3485](http://www.genome.jp/dbget-bin/www_bget?xac:XAC3485) | plus | 119 |
| 152 | GGGAATCGGGAATCGGGAATCGGGAATCGGGAATCGGGAATCGGGAATCGGGAATC | plus | 56 | 8 | 4121857 | 4121912 | no | n.a. | cebR | [XAC3487](http://www.genome.jp/dbget-bin/www_bget?xac:XAC3487) | plus | 4 | suc1 | [XAC3488](http://www.genome.jp/dbget-bin/www_bget?xac:XAC3488) | minus | 93 |
| 153 | GGGAATCGGGAATC | plus | 14 | 2 | 4176929 | 4176942 | no | n.a. | transcriptional regulator | [XAC3532](http://www.genome.jp/dbget-bin/www_bget?xac:XAC3532) | plus | 4 | hypothetical protein | [XAC3533](http://www.genome.jp/dbget-bin/www_bget?xac:XAC3533) | minus | 388 |
| 154 | GGGATTGGGGAATCGGGAATCGGGAATCGGGAAAA | plus | 35 | 5 | 4187704 | 4187738 | no | n.a. | xpsF | [XAC3543](http://www.genome.jp/dbget-bin/www_bget?xac:XAC3543) | minus | 56 | xpsE | [XAC3544](http://www.genome.jp/dbget-bin/www_bget?xac:XAC3544) | minus | 126 |
| 155 | GGGAATCGGGAATCGGGAATCGGGAATG | minus | 28 | 4 | 4206388 | 4206361 | other | 28 | intragenic: purL | [XAC3549](http://www.genome.jp/dbget-bin/www_bget?xac:XAC3549) | minus |  |  |  |  |  |
| 156 | GGGAGCTGGGAATCGCGAGTCGGGAATGGGGAATG | plus | 35 | 5 | 4206963 | 4206997 | 157 | 38 | intragenic: purL | [XAC3549](http://www.genome.jp/dbget-bin/www_bget?xac:XAC3549) | minus |  |  |  |  |  |
| 157 | GGGAATCGGGAATCGGGAGTCGGGAATC | minus | 28 | 4 | 4207063 | 4207036 | 156 | 38 | intragenic: purL | [XAC3549](http://www.genome.jp/dbget-bin/www_bget?xac:XAC3549) | minus |  |  |  |  |  |
| 158 | GGGACTCGGGAATGGGAATC | plus | 20 | 3 | 4208899 | 4208918 | no | n.a. | purL | [XAC3549](http://www.genome.jp/dbget-bin/www_bget?xac:XAC3549) | minus | 80 | dsbC | [XAC3550](http://www.genome.jp/dbget-bin/www_bget?xac:XAC3550) | minus | 439 |
| 159 | GTGAATCGGGAATGGGGAATGGGGAATC | plus | 28 | 4 | 4217409 | 4217436 | 160 | 54 | holC | [XAC3558](http://www.genome.jp/dbget-bin/www_bget?xac:XAC3558) | plus | -4 | valS | [XAC3559](http://www.genome.jp/dbget-bin/www_bget?xac:XAC3559) | plus | 102 |
| 160 | GGGAATGGGGAGTCGGGAATAGGGAATAGGGAATAGGGAATG | minus | 42 | 6 | 4217532 | 4217491 | 159 | 54 | valS | [XAC3559](http://www.genome.jp/dbget-bin/www_bget?xac:XAC3559) | plus | 6 | holC | [XAC3558](http://www.genome.jp/dbget-bin/www_bget?xac:XAC3558) | plus | 78 |
| 161 | GGGAATGGGGAATCGTGAATCGGGAATT | plus | 28 | 4 | 4330091 | 4330118 | 162 | 23 | atpG | [XAC3650](http://www.genome.jp/dbget-bin/www_bget?xac:XAC3650) | minus | 24 | atpA | [XAC3651](http://www.genome.jp/dbget-bin/www_bget?xac:XAC3651) | minus | 61 |
| 162 | GGGAATCGGGAATAGAGAATAGGGAATC | minus | 28 | 4 | 4330142 | 4330169 | 161 | 23 | atpA | [XAC3651](http://www.genome.jp/dbget-bin/www_bget?xac:XAC3651) | minus | 10 | atpG | [XAC3650](http://www.genome.jp/dbget-bin/www_bget?xac:XAC3650) | minus | 75 |
| 163 | GGGAATCGGGAATC | minus | 14 | 2 | 4372692 | 4372679 | other | 393 | hypothetical protein | [XAC3697](http://www.genome.jp/dbget-bin/www_bget?xac:XAC3697) | minus | 9 | hypothetical protein | [XAC3696](http://www.genome.jp/dbget-bin/www_bget?xac:XAC3696) | plus | 426 |
| 164 | GGTAATCGGGAATCGGGATTGGGGAATC | minus | 28 | 4 | 4376052 | 4376025 | no | n.a. | yjcE | [XAC3701](http://www.genome.jp/dbget-bin/www_bget?xac:XAC3701) | minus | 16 | yadG | [XAC3700](http://www.genome.jp/dbget-bin/www_bget?xac:XAC3700) | minus | 252 |
| 165 | GGGAATGGGGAATC | minus | 14 | 2 | 4525854 | 4525841 | no | n.a. | amaA | [XAC3847](http://www.genome.jp/dbget-bin/www_bget?xac:XAC3847) | minus | 2 | queF | [XAC3846](http://www.genome.jp/dbget-bin/www_bget?xac:XAC3846) | minus | 142 |
| 166 | GGGAATCGGGAATCGGGAACTGGGATTCGGGAATCGGGAATC | minus | 42 | 6 | 4532156 | 4532115 | no | n.a. | acrA | [XAC3850](http://www.genome.jp/dbget-bin/www_bget?xac:XAC3850) | plus | 12 | acrD | [XAC3849](http://www.genome.jp/dbget-bin/www_bget?xac:XAC3849) | plus | 105 |
| 167 | GGGAATCGGGAGTCGGGAATAGGGAATT | plus | 28 | 4 | 4554768 | 4554795 | 168 | 49 | folB | [XAC3870](http://www.genome.jp/dbget-bin/www_bget?xac:XAC3870) | minus | 1 | gcp | [XAC3871](http://www.genome.jp/dbget-bin/www_bget?xac:XAC3871) | minus | 69 |
| 168 | GGGAGTGGGGAATCGGGAATAGTGAAGAGTGAAGAGTGAAGA | minus | 42 | 6 | 4554886 | 4554845 | 167 | 49 | gcp | [XAC3871](http://www.genome.jp/dbget-bin/www_bget?xac:XAC3871) | minus | -22 | folB | [XAC3870](http://www.genome.jp/dbget-bin/www_bget?xac:XAC3870) | minus | 78 |
| 169 | GGGAAAGGGGAATTGGGAGTAGGGAATC | plus | 28 | 4 | 4567309 | 4567336 | 170 | 29 | hypothetical protein | [XAC3883](http://www.genome.jp/dbget-bin/www_bget?xac:XAC3883) | plus | 6 | cox3 | [XAC3884](http://www.genome.jp/dbget-bin/www_bget?xac:XAC3884) | minus | 48 |
| 170 | GAGAATCGGGAGTCGGGAATC | minus | 21 | 3 | 4567386 | 4567366 | 169 | 29 | cox3 | [XAC3884](http://www.genome.jp/dbget-bin/www_bget?xac:XAC3884) | minus | -2 | hypothetical protein | [XAC3883](http://www.genome.jp/dbget-bin/www_bget?xac:XAC3883) | plus | 63 |
| 171 | GGGAATGGGGAAATGGGAATGGGGAATC | plus | 28 | 4 | 4589384 | 4589411 | 172 | 52 | exoA | [XAC3902](http://www.genome.jp/dbget-bin/www_bget?xac:XAC3902) | minus | 5 | pyrE | [XAC3903](http://www.genome.jp/dbget-bin/www_bget?xac:XAC3903) | plus | 86 |
| 172 | GGGTATGGGGAATGGGGAATCGGGAATG | minus | 28 | 4 | 4589492 | 4589465 | 171 | 52 | pyrE | [XAC3903](http://www.genome.jp/dbget-bin/www_bget?xac:XAC3903) | plus | 5 | exoA | [XAC3902](http://www.genome.jp/dbget-bin/www_bget?xac:XAC3902) | minus | 86 |
| 173 | GCGAATGGGGAATCGGGAGTTGGGAATC | plus | 28 | 4 | 4677889 | 4677916 | 174 | 25 | yhhT | [XAC3985](http://www.genome.jp/dbget-bin/www_bget?xac:XAC3985) | plus | 2 | hydrolase | [XAC3986](http://www.genome.jp/dbget-bin/www_bget?xac:XAC3986) | plus | 70 |
| 174 | GGGGATGGAGAGTCGGGAATCGAGAATG | minus | 28 | 4 | 4677969 | 4677942 | 173 | 25 | hydrolase | [XAC3986](http://www.genome.jp/dbget-bin/www_bget?xac:XAC3986) | plus | 17 | yhhT | [XAC3985](http://www.genome.jp/dbget-bin/www_bget?xac:XAC3985) | plus | 55 |
| 175 | GCGAATCGGGAATCGGGAATG | plus | 21 | 3 | 4691698 | 4691718 | no | n.a. | hypothetical protein | [XAC3998](http://www.genome.jp/dbget-bin/www_bget?xac:XAC3998) | plus | 17 | hypothetical protein | [XAC3999](http://www.genome.jp/dbget-bin/www_bget?xac:XAC3999) | plus | 144 |
| 176 | GGGAATGGGGAGTCGGGAATAGGGAATC | plus | 28 | 4 | 4768651 | 4768678 | 177 | 33 | flavodoxin | [XAC4073](http://www.genome.jp/dbget-bin/www_bget?xac:XAC4073) | minus | 7 | nrdF | [XAC4074](http://www.genome.jp/dbget-bin/www_bget?xac:XAC4074) | minus | 60 |
| 177 | GAGAATAGGGAATCGGGAATG | minus | 21 | 3 | 4768732 | 4768712 | 176 | 33 | nrdF | [XAC4074](http://www.genome.jp/dbget-bin/www_bget?xac:XAC4074) | minus | 6 | flavodoxin | [XAC4073](http://www.genome.jp/dbget-bin/www_bget?xac:XAC4073) | minus | 68 |
| 178 | GGGAATGGGGAATCGGGAGCAGGGAATC | plus | 28 | 4 | 4915957 | 4915984 | 179 | 210 | acs | [XAC4179](http://www.genome.jp/dbget-bin/www_bget?xac:XAC4179) | plus | 6 | tcsR | [XAC4180](http://www.genome.jp/dbget-bin/www_bget?xac:XAC4180) | plus | 254 |
| 179 | GGGAATCGGGAATCGGGAGTGGGGAATCGGGAATC | minus | 35 | 5 | 4916229 | 4916195 | 178 | 210 | tcsR | [XAC4180](http://www.genome.jp/dbget-bin/www_bget?xac:XAC4180) | plus | 9 | acs | [XAC4179](http://www.genome.jp/dbget-bin/www_bget?xac:XAC4179) | plus | 244 |
| 180 | GGGATTTGGGAATCGGGATTC | minus | 21 | 3 | 5158178 | 5158158 | no | n.a. | dipeptidase | [XAC4362](http://www.genome.jp/dbget-bin/www_bget?xac:XAC4362) | minus | 5 | ttuB | [XAC4361](http://www.genome.jp/dbget-bin/www_bget?xac:XAC4361) | minus | 381 |
| 181 | GGGAATGGGGAATCGGGAGTGGGGAATC | minus | 28 | 4 | 5158216 | 5158189 | no | n.a. | intragenic: dipeptidase | [XAC4362](http://www.genome.jp/dbget-bin/www_bget?xac:XAC4362) | minus |  | ttuB | [XAC4361](http://www.genome.jp/dbget-bin/www_bget?xac:XAC4361) | minus | 412 |
| 182 | GGGGATTGGGAATC | plus | 14 | 2 | 5162560 | 5162573 | no | n.a. | ygjT | [XAC4365](http://www.genome.jp/dbget-bin/www_bget?xac:XAC4365) | plus | 6 | hypothetical protein | [XAC4366](http://www.genome.jp/dbget-bin/www_bget?xac:XAC4366) | plus | 363 |
| 183 | GGGAATCGGGAATCGGGAATC | minus | 21 | 3 | 5165183 | 5165163 | no | n.a. | fecA | [XAC4368](http://www.genome.jp/dbget-bin/www_bget?xac:XAC4368) | minus | 4 | glpQ | [XAC4367](http://www.genome.jp/dbget-bin/www_bget?xac:XAC4367) | minus | 200 |

S2 Table C: GGGGA(C/T)T Repeats in Nostoc sp. PCC7120

Table shows the occurrence of GGGGA(C/T)T repeat sequences in the *Nostoc sp.* PCC 7120 genome (NC_003272). Sequence of the repeat, start and end point of the sequence on the genome, its total length, and location on plus (+) or minus (-) strand are given. Participation in inverted repeat (inv rep) formation is stated with the number of the partnering repeat, other indicates occurrence of another G-rich sequence or another repeat type in place of the partnering GGGGA(C/T)T repeat. The next upstream and downstream genes are listed by locus tags and the distance between the neighboring gene and the repeat (dis [nt]). Intragenic repeats and repeats overlapping with ORFs are showing in the last column, location on the coding or non-coding strand is stated.

| **#** | **Sequence [5’-3’]** | **start** | **end** | **Length[nt]** | **strand** | **inv rep** | **upstream gene locus tag** | **dis [nt]** | **downstream gene locus tag** | **dis [nt]** | **intragenic locus tag** |
| --- | --- | --- | --- | --- | --- | --- | --- | --- | --- | --- | --- |
| 1 | GGGGATTGGGGATTGGGGACTGGGGACTGGGG | 80969 | 80938 | 32 | (-) | no | alr0076 | 124 | alr0075 | 5 |  |
| 2 | GGGGACTGGGGACTGGGTATGGGGACTGGGGAAGTGGAGGG | 111837 | 111877 | 41 | (+) | no | all0109 (psaF) | 166 | alr0110 | 78 |  |
| 3 | GGGATTGGGGACTGGGGACTGGGGACTGGGGACTGGGAATTGGGAATTGGGG | 149871 | 149820 | 52 | (-) | no | all0144 | 139 | all0143 |  | all0143 (coding)  overlapping with stop codon, -1 |
| 4 | GGGGATTGGGGACTGGGGATTGGGGACTGGGGATTGGGGACTGGGGATTGGG | 169690 | 169639 | 52 | (-) |  | all0166 | 1290 | all0164 | 1404 | alr0165 (non-coding) |
| 5 | GGGGGATTGGGGACTGGGGACTGGGGAAGGTGGG | 254178 | 254145 | 34 | (-) | no | alr0237 | 108 | alr0236 | 70 |  |
| 6 | GGGGACTGGGGATCGGGGATTGGGAAGGTGGGGACTGGGG | 279040 | 279079 | 40 | (+) | no | asl0260 | 113 | all0261 | 45 |  |
| 7 | GGGGACTGGGGACTGGGGATTGGGGACTGGGGATTGGGGATTGGG | 287821 | 287777 | 45 | (-) | yes | all0268 | 126 | alr0267 | 4 |  |
| 8 | GGGGATTGGGGATTGGGGACTGGGGATTGGGGACTGGG | 290772 | 290809 | 38 | (+) | other | asl0272 | 57 | all0273 (prfB) | 46 |  |
| 9 | GGGACTGGGGACTGGGGACTGGGGACTGGGGATTGGGGACTGGGGATTGGGG | 353465 | 353414 | 52 | (-) |  | alr0309 | 3116 | all0307 | 1052 | alr0308 (non-coding) |
| 10 | GGGGATTGGGGATTGGGGATTGGGTGCTGGGGATTGGGGATTGGGGATTGGG | 377483 | 377534 | 52 | (+) | other | all0328 | 10 | all0329 (psaD) | 88 |  |
| 11 | GGGGATTGGGGAACCGGGATTGGGGACTGGGGAACGGGG | 573075 | 573037 | 39 | (-) |  | all0479 | 4470 | alr0477 | 749 | all0478 (coding) |
| 12 | GGGGATTGGGGATTGGGGATTGGGGATTGGGGATTGGGGATTGGGGATTGGGGATTGGGGATTGGGGATTGGGGATTGGGGATTGGGGATTGGGGATTGGGGATTGGGGATTGGGGATTGGGGATTGGGGATTGGGGATTGGGGATTGGGGATTGGGGATTGGGGATTGGGGATTGGGG | 619299 | 619477 | 179 | (+) | no | alr0526 (pecE) | 19 | alr0527 (pecF) | 219 |  |
| 13 | GGGGATAAGGGGTAATGGGGATTAGGGATTGGGTACTAGGGATTGGGGATTGGGATTAGGG | 641801 | 641741 | 61 | (-) | no | alr0548 | 61 | alr0547 | 50 |  |
| 14 | GGGGATTGGGGACTGGGGATTGGGGATTGGGGATTAGGGATTGGGGATTGGGGATTAGGGATTGGGGATTGGGGATTAGGGATTGGGGATTAGGGATTGGGGATTAGGGATTGGGGATTAGGGATTGGG | 643135 | 643263 | 129 | (+) | no | alr0548 | 1 | alr0549 | 55 |  |
| 15 | GGGGACTGGGGACTGGGGACTGGGG | 848109 | 848085 | 25 | (-) |  | alr0730 | 1093 | alr0728 | 4199 | all0729 (coding) |
| 16 | GGGGCTGGGGACTGGGGACTGGGGGCTGGG | 911056 | 911085 | 30 | (+) |  | alr0787 | 828 | alr0789 | 103 | all0788 (purN) (non-coding) |
| 17 | GGGACTGGGGATTGGGGACTGGGGATTGGGGACTGGGGATTGGGGACTGGG | 923197 | 923147 | 51 | (-) | no | asr0798 | 192 | all0797 | 71 |  |
| 18 | GGGATTGGGGATTGGGGGCTGGGGACTGGGG | 1102853 | 1102823 | 31 | (-) | no | all0949 | 135 | all0948 | 1 |  |
| 19 | GGGGATTGGGGATTGGGGATTGGGGACTGGGGATTGGGGACTGGGGGTTGGGGAATGGGGATTGGGG | 1107293 | 1107227 | 67 | (-) | other | alr0952 (coxC) | 88 | alr0951 (coxA) | 15 |  |
| 20 | GGGGGTTGGGGATTGGGGATTGGGGGTTGGGGATTGGGGATTGGGTACTGGGGATTGGGTACTGGGGATTGGGG | 1213183 | 1213110 | 74 | (-) | other | alr1042 (hemA) | 108 | alr1041 (glpX) | 38 |  |
| 21 | GGGGACTGGGGACTGGGGATTGGGGATTGGGGATTGGGATTGGGGATTGGGTACTGGGG | 1281938 | 1281880 | 59 | (-) | no | alr1094 | 197 | alr1093 | 142 |  |
| 22 | GGGGACTGGGGATTGGGGACTGGGGACTGGGG | 1284314 | 1284283 | 32 | (-) | no | alr1096 | 71 | alr1095 (gap3) | 1 |  |
| 23 | GGGGACTGGGGGGATTGGGGATGGGGGACTGGG | 1360437 | 1360405 | 33 | (-) | no | alr1155 | 374 | all1154 | 18 |  |
| 24 | GGGATTGGGGATTGGGTACTGGGGATTGGGTACTGGGGATTGGGTAATGGGGATTGGG | 1441553 | 1441611 | 58 | (+) | other | all1221 (btpA) | 18 | alr1222 | 658 |  |
| 25 | GGGACTGGGGACTGGGGACTGGGGATTGGGGGAGCAGGGG | 1470074 | 1470113 | 40 | (+) | other | alr1259 | 626 | alr1238 (clpP) | 127 |  |
| 26 | GGGGGTTGGGGATTGGGGACTGGGGACTGGGGACTGGGGATTGGGGACTGGGG | 1494743 | 1494691 | 53 | (-) | no | alr1260 | 39 | alr1259 | 4 |  |
| 27 | GGGGGTTGGGTATCGGGGATTGGGGGTTGGGGATTGGGGGTTGGGGATTGGGGATTGGGGACTGGGGATTAGGGACTAGGGATTGGGGATTGGGGATTGGGGACTGGGGATTGGGGACTGGGGATTGGGGGTTAGGGACTGGG | 1575364 | 1575506 | 143 | (+) | other | all1325 | 43 | all1326 | 50 |  |
| 28 | GGGGATGAGGGATTGGGGATTGGGGATGGGG | 1587606 | 1587576 | 31 | (-) | no | alr1337 | 37 | alr1336 | 80 |  |
| 29 | GGGATTAGGGACTGGGGACTGGGGACTAGGGACTGGGGACTAGGG | 1595025 | 1595069 | 45 | (+) | other | all1342 | 133 | alr1343 | 90 |  |
| 30 | GGGTTTTAGGGGGATTGGGGACTGGGGATTGGGGACTGGGGATTGGGGACTGGG | 1638008 | 1638061 | 54 | (+) | no | all1380 | 2332 | alr1382 | 225 | alr1381 (prcA) (coding)  overlapping with stop coding, -9 |
| 31 | GGGGACTGGGGACTGGGGACTGGGGG | 1997699 | 1997674 | 26 | (-) | no | alr1675 | 1451 | all1673 | 1076 | alr1674 (non-coding) |
| 32 | GGGGATTGGGGACTGGGGATTGGGGATTGGGGGAAGGG | 2191285 | 2191248 | 38 | (-) | no | all1826 | 36 | asl1825 | 2 |  |
| 33 | GGGGATTGGGGACTGGGGACTGGGGACTGGGGATTGGGGATTGGGGATAAGGGGTAATGGGGATTTTGGG | 2236798 | 2236867 | 70 | (+) | no | alr1874 | 2 | all1875 | 4 |  |
| 34 | GGGGATTGGGGACTGGGGATTGGGGATTGGGGACTGGGGACTGGGGATTGGGGACTGGGGATTGGGGATTGGGG | 2270552 | 2270479 | 74 | (-) | other | alr1901 | 66 | alr1900 (glmM) | 39 |  |
| 35 | GGGACTGGGGATTGGGGACTGGGGACTGGGGATTGGGGG | 2303778 | 2303740 | 39 | (-) | other | alr1921 | 71 | alr1920 | 28 |  |
| 36 | GGGGAATGGGGACTGGGGACTGGGGATTGGGGATTGGGTAATCGGG | 2401236 | 2401281 | 46 | (+) | yes (37) | all2007 | 3 | all2008 | 185 |  |
| 37 | GGGGTAGGGATTGGGTATTGGGGATTGGGGACTGGGGACTGGGG | 2401472 | 2401429 | 44 | (-) | yes (36) | alr2009 (dnaA) | 196 | all2007 | 1546 | all2008 (coding)  overlapping with stop codon, -7 |
| 38 | GGGATTGGGGATTGGGGATTGGGGACTGGG | 2521330 | 2521359 | 30 | (+) |  | asl2101 | 1334 | all2103 | 104 | all2102 (ama) (non-coding) |
| 39 | GGGATTAGGGATTGGGGATTAGGGATTGGGGATTAGGGATTGGGGATTAGGGATTGGGGATTGGGGACTGGGGAAGGGG | 2736570 | 2736492 | 79 | (-) |  | alr2274 (lpxB) | 90 | alr2272 | 11 | asr2273 (non-coding)  overlapping with stop codon, **-**47 |
| 40 | GGGGATTGGGGATTGGGGATTGAGGGTTGGGGATTGGGGGTTGGGGGTTGGGG | 2855593 | 2855541 | 53 | (-) | no | alr2373 | 57 | alr2372 | 7 |  |
| 41 | GGGATTAGGGATTAGGGATTGGGGATTGGGGACTGGGGATTGGGGATTGGGGATGAAGGGG | 2916857 | 2916917 | 61 | (+) | no | asr2427 | 5222 | alr2429 | 60 | alr2428 (coding) overlapping with stop codon, -8 |
| 42 | GGGGACTGGGGACTGGGGACTGGGGACTGGGGACTGGGG | 3096498 | 3096536 | 39 | (+) | no | alr2588 | 3 | alr2589 | 5 |  |
| 43 | GGGATTGGGGATTGGGGATTGGGGATTGGG | 3131530 | 3131559 | 30 | (+) | no | all2622 | 114 | all2623 | 86 |  |
| 44 | GGGATTGGGGATTGGGGATTGGGGACTGGGGATTGGGGATTGGGGATTGGGG | 3160483 | 3160534 | 52 | (+) | yes (45) | all2641 | 446 | all2643 | 2997 | all2642 (non-coding) |
| 45 | GGGACTGGGGACTGGGGACTGGGGACTGGGGACTGGG | 3160866 | 3160830 | 37 | (-) | yes  (44) | all2643 | 793 | all2641 | 2665 | all2642 (coding) |
| 46 | GGGGGACTGGGGACTGGGGACTGGGGACTGGGGACTGGGG | 3164328 | 3164289 | 40 | (-) |  | all2644 | 755 | all2642 | 2642 | all2643 (coding) |
| 47 | GGGGACTGGGGACTGGGGACTGGGGATTGGGGACTGGGGATTGGGGTGTCTAGGGAAATTATGGG | 3169803 | 3169867 | 65 | (+) |  | all2643 | 2964 | all2645 | 5059 | all2644 (non-coding) |
| 48 | GGGACTGGGGACTGGGGACTGGGGACTGGGGACTGGG | 3185044 | 3185080 | 37 | (+) |  | all2645 | 2739 | all2647 | 1811 | all2646 (non-coding) |
| 49 | GGGGACTGGGGACTGGGGACTGGGGACTGGG | 3192261 | 3192231 | 31 | (-) |  | all2649 | 2080 | all2647 | 5659 | all2648 (coding) |
| 50 | GGGCATGGGGTATGGGGCATGGGGCATGGGG | 3195367 | 3195397 | 31 | (+) |  | all2647 | 5216 | all2649 | 2523 | all2648 (non-coding) |
| 51 | GGGGACTGGGGACTGGGGACTGGGGACTGGGG | 3410753 | 3410722 | 32 | (-) |  | all2804 | 1385 | alr2802 | 2539 | alr2803 (nifJ) (non-coding) |
| 52 | GGGGATTGGGGATTGGGGATTGGGGACTGGGGACTGGGG | 3422138 | 3422100 | 39 | (-) |  | all2813 | 131 | alr2811 (avtA) | 680 | alr2812 (rimM) (non-coding) |
| 53 | GGGACTAGGGACTGGGGATTGGGGATTGGGGACTGGGGATTGGGGATTGGGGATTGGGG | 3422825 | 3422767 | 59 | (-) | other | alr2814 | 54 | alr2812 (rimM) | 747 | all2813 (coding)  overlapping with stop codon, -8 |
| 54 | GGGATTGGGGATTGGGGACTGGGGATTGGGGATTGGGGATTGGGGATTGGGG | 3456247 | 3456196 | 52 | (-) | no | alr2836 | 135 | alr2835 (hepA) | 1 |  |
| 55 | GGGGACTGGGGACTGGGGACTGGGGACTGGG | 3585960 | 3585990 | 31 | (+) |  | alr2947 | 146 | alr2948 | 179 |  |
| 56 | GGGTTGGGGATTGGGGACTGGGGACTGGGG | 3693093 | 3693064 | 30 | (-) | no | alr3050 | 822 | all3049 | 18 |  |
| 57 | GGGGATTGGGGATTGGGGACTGGGGATTGGGG | 3702801 | 3702770 | 32 | (-) | other | alr3057 | 69 | alr3056 (hisD) | 577 |  |
| 58 | GGGGATTGGGGATTGGGGATTGGGGATTGGG | 3853236 | 3853266 | 31 | (+) | no | alr3183 | 0 | all3184 (lrtA) | 79 |  |
| 59 | GGGGATTGGGGATTGGGGATTGGGGATTGGGGATTGGGGATTGGGG | 3878954 | 3878999 | 46 | (+) | no | all3209 | 7 | alr3210 | 26 |  |
| 60 | GGGAATTGGGGACTGGGGACTGGGGACGGGG | 3900907 | 3900877 | 31 | (-) | no | alr3230 | 34 | alr3229 (ksgA) | 6 |  |
| 61 | GGGGATTGGGGACTGGGGACTGGGTATTGGGGACTGGGGACTGGGTATTGGGTGTTGGG | 4038623 | 4038681 | 59 | (+) | no | asr3342 (acpP) | 10 | alr3343 | 153 |  |
| 62 | GGGATTGGGGACTGGGGATTGGGGATTGGGGATTGGGATTGGGGATTGGGGATTGGGGATTGGGGATTGGGGATTGGGGATTGGGGATTGGG | 4247500 | 4247409 | 92 | (-) |  | all3526 | 44 | alr3524 | 1350 | alr3525 (non-coding) |
| 63 | GGGACTGGGGATTAGGGACTGGGGACTGGGGACTGGGGACTGGGG | 4262942 | 4262898 | 45 | (-) | other | all3538 (eno) | 64 | alr3537 (argC) |  | alr3537 (coding)  overlapping with stop codon, -1 |
| 64 | GGGGATTGGGGATTGGGGATTGGGGACTGGGG | 4265346 | 4265377 | 32 | (+) | other | alr3539 | 8 | all3540 | 23 |  |
| 65 | GGGACAGGGATTGGGGATTGGGGACTGGGGATTGGGGACTGGGGACTGGG | 4302919 | 4302968 | 50 | (+) |  | alr3562 | 575 | all3564 | 1367 | all3563 (non-coding) |
| 66 | GGGATTGGGGGTTGGGGACTGGGGACTGGGGGTTGGGTAAAGGGGATTGGGGATTGGG | 4304910 | 4304967 | 58 | (+) |  | all3563 | 694 | alr3565 | 1138 | all3564 (non-coding) |
| 67 | GGGGAGTAGGGATTGGGGACTGGGGATTGGGGATTGGGGATTGGG | 4312093 | 4312049 | 45 | (-) | other | all3570 (ppa) | 114 | all3569 | 5 |  |
| 68 | GGGGATTAGGGATTAGGGATTGGGGATTGGGGATTGGGGATTGGGG | 4429252 | 4429297 | 46 | (+) | no | alr3671 | 1434 | all3673 | 114 | alr3672 (coding)  overlapping with stop codon, -1 |
| 69 | GGGGGCTGGGGACTGGGTAACGGGGACTGGGGACTGGG | 4609185 | 4609222 | 38 | (+) | no | alr3811 | 132 | alr3812 | 335 |  |
| 70 | GGGGACTGGGGATTGGGGGACTGGGGGCTGGGGATTGGG | 4687813 | 4687775 | 39 | (-) | no | alr3887 | 86 | asr3886 | 9 |  |
| 71 | GGGGGATGGGGGACTGGGGATTGGGGATTGGGGATTGGGGATTGGGGATTGGG | 4751751 | 4751699 | 53 | (-) | other | asr3935 | 1321 | all3933 | 1203 | alr3934 (non-coding) |
| 72 | GGGGACTGGGGACTAGGGGTTGGGGACTGGGG | 4950667 | 4950636 | 32 | (-) | no | all4109 | 336 | asr4108 | 189 | allrs04 (rnpB) RNA subunit of RNase P |
| 73 | GGGGATTGGGTACTGGGGACTGGGGATTGGG | 5036094 | 5036064 | 31 | (-) | no | all4187 (rpsI) | 74 | asl4186 (rpmE) | 4 |  |
| 74 | GGGACTGGGGATTGGGGATTGGGGACTGGGGATTGGG | 5083856 | 5083820 | 37 | (-) | no | alr4240 | 10 | alr4239 | 16 |  |
| 75 | GGGGATTGGGGATTGGGGATTGGGGATTGGGGATTGGGGACTGGG | 5117175 | 5117131 | 45 | (-) |  | alr4268 | 1422 | asl4266 | 1082 | alr4267 (non-coding) |
| 76 | GGGATTGGGGATTGGGGACTGGGGATTGGGGACTGGGGACTGGGGATTGGGGATTGGGGACTGGGGACTGGGGACTGGG | 5219352 | 5219430 | 79 | (+) |  | all4355 | 114 | all4357 (clpP) | 81 | asl4356 (non-coding) |
| 77 | GGGATTGGGGACTGGGGATTGGGGACTGGGGACTAGGGATTGGGGACTAGGGATTGGGGACTGGGGATTGGGGACTGGGGATTGGGGACTGGGGACTGGGGACTAGGGATTGGGGACTGGG | 5238394 | 5238514 | 121 | (+) | other | asl4372 | 484 | all4374 | 102 | alr4373 (coding)  overlapping with stop codon, -1 |
| 78 | GGGGATTGGGGACTGGGGATTGGGG | 5263152 | 5263176 | 25 | (+) | other | all4389 | 103 | all4390 (hisB) | 60 |  |
| 79 | GGGATTGGGGATTAGGGATTGGGGATTGGGGGTTGGGGACTGGGGATTGGGGATTGGGTATTGGGGATTGGG | 5330538 | 5330609 | 72 | (+) | no | all4445 | 1 | all4446 | 63 |  |
| 80 | GGGGACTGGGGATTGGGGACTGGGGATTGGGG | 5431034 | 5431003 | 32 | (-) | no | all4538 | 78 | alr4537 | 9 |  |
| 81 | GGGGACTGGGGACTGGGGACTGGGGACTGGGG | 5514081 | 5514050 | 32 | (-) |  | all4613 (ilvG) | 183 | alr4611 | 91 | asr4612 (non-coding) |
| 82 | GGGGACTGGGAACTGGGAATTGGGGATTGGGGACTGGGGG | 5551214 | 5551253 | 40 | (+) | no | alr4646 | 6 | all4647 | 122 |  |
| 83 | GGGGATTGGGGATTGGGGATTGGGGACTGGGGATTGGGGACTGGGGATTGGGGACTGGGGATTGGGGATTGGG | 5658801 | 5658729 | 73 | (-) | no | alr4745 | 130 | all4744 | 88 |  |
| 84 | GGGGATTGGGGATTGGGGATTTGGGATTGGGCAGGGGGCAGGGGGCAGGGGG | 5660419 | 5660368 | 52 | (-) | other | alr4746 (trpC) | 52 | alr4745 | 5 |  |
| 85 | GGGGACTGGGGGCTGGGGATTGGGG | 5789425 | 5789449 | 25 | (+) |  | asl4860 | 1193 | asl4862 | 2038 | all4861 (pepC) (non-coding) |
| 86 | GGGGATTGGGGACTGGGGATTGGGGATTGGGGACTGGGGACTGGGGACTGGGG | 5826050 | 5825998 | 53 | (-) |  | alr4890 | 58 | alr4888 | 149 | asl4889 (coding) |
| 87 | GGGGATTGGGGATTGGGGACTGGGGATTGGGGACTGGGGATTGGGGATTAGGGACTGGGG | 5995539 | 5995480 | 60 | (-) | no | all5023 | 114 | all5022 | 2 |  |
| 88 | GGGATTGGGGACTGGGGATTGGGGACTGGGGG | 6134817 | 6134786 | 32 | (-) | no | all5138 (sat) | 110 | all5137 | 7 |  |
| 89 | GGGATTAGGGATTGGGGACTGGGGATTGGGGATAAGGGGTAATAAGGACTGGGGGTTGGGGATTGGGGACTGGGG | 6327021 | 6327059 | 39 | (+) | other | all5305 | 9 | all5306 | 131 |  |
