# Supplementary material for: Investigation of a Quadruplex-Forming Repeat Sequence Highly Enriched in Xanthomonas and Nostoc sp
Source: PLoS One. 2015 Dec 22;10(12):e0144275. doi: 10.1371/journal.pone.0144275 (PMC4692102; doi:10.1371/journal.pone.0144275)
Supplement: S3 File — DNA Oligonucleotides (Table A). Longest Repeats in Xcc (Table B ). Longest Repeats in Xac (Table C ). CD spectra of (GGGAATC)3GGG variants with G to T mutations in G-tract (Fig A). Melting temperatures of G-quadruplex structures (Table D). Melting profiles of G-rich repeat oligos at pH 7.5 (Fig B) Melting temperatures of structures formed by C-rich repeat oligonucleotides (Table E). CD spectra of C-rich repeat oligos in Na-acetate buffer (Fig C). Melting profiles of C-rich repeat oligos in Na-acetate buffer (Fig D). Distance of repeats to neighboring ORFs for potential quadruplex forming sequences in Xcc (Fig E). (DOCX) [file pone.0144275.s003.docx]

**Supporting Information 3:**

**Contents:**

**S3 Table A**: **DNA Oligonucleotides**

**S3 Table B**: **Longest Repeats in *Xcc***

**S3 Table C: Longest Repeats in *Xac***

**S3 Figure A**: **CD spectra of (GGGAATC)_3_GGG variants with G to T mutations in G-tract.**

**S3 Table D**: **Melting temperatures of G-quadruplex structures**

**S3 Figure B**: **Melting profiles of G-rich repeat oligos at pH 7.5.**

**S3 Table E: Melting temperatures of structures formed by C-rich repeat oligonucleotides**

**S3 Figure C**: **CD spectra of C-rich repeat oligos in Na-acetate buffer**

**S3 Figure D: Melting profiles of C-rich repeat oligos in Na-acetate buffer**

**S3 Figure E: Distance of repeats to neighboring ORFs for potential quadruplex forming sequences in *Xcc***

**S3 Table A**: **DNA Oligonucleotides**

| Name | Sequence (5‘-3‘) |
| --- | --- |
| (GGGAATC)_3_GGG | GGGAATCGGGAATCGGGAATCGGG |
| (GGGAATC)_4_ | GGGAATCGGGAATCGGGAATCGGGAATC |
| GTG1 | GTGAATCGGGAATCGGGAATCGGG |
| GTG2 | GGGAATCGTGAATCGGGAATCGGG |
| GTG3 | GGGAATCGGGAATCGTGAATCGGG |
| GTG4 | GGGAATCGGGAATCGGGAATCGTG |
| (GGGGACT)_3_GGGG | GGGGACTGGGGACTGGGGACTGGGG |
| (GGGGATT)_3_GGGG | GGGGATTGGGGATTGGGGATTGGGG |
| (GGGACTG)_3_GGG | GGGACTGGGGACTGGGGACTGGGG |
| (GGGATTG)_3_GGG | GGGATTGGGGATTGGGGATTGGGG |
| (GGGGACT)_4_ | GGGGACTGGGGACTGGGGACTGGGGACT |
| (GGGGATT)_4_ | GGGGATTGGGGATTGGGGATTGGGGATT |
| (CCCGATT)_3_CCC | CCCGATTCCCGATTCCCGATTCCC |
| (GATTCCC)_4_ | GATTCCCGATTCCCGATTCCCGATTCCC |
| (CCCCAGT)_3_CCCC | CCCCAGTCCCCAGTCCCCAGTCCCC |
| (CCCCAAT)_3_CCCC | CCCCAATCCCCAATCCCCAATCCCC |
| (CCCCAGT)_3_CCC | CCCCAGTCCCCAGTCCCCAGTCCC |
| (CCCCAAT)_3_CCC | CCCCAATCCCCAATCCCCAATCCC |
| (AGTCCCC)_4_ | AGTCCCCAGTCCCCAGTCCCCAGTCCCC |
| (AATCCCC)_4_ | AATCCCCAATCCCCAATCCCCAATCCCC |

**S3 Table B**: **Longest Repeats in *Xcc***

Sequences of the longest repeats (≥ 7 units) in Xcc with G-tracts underscored. Total length, number of repeat units, locus tags and description of upstream and downstream neighboring genes are given. Occurrence of a repeat as part of the inverted repeat is shown (inv rep), and number of partnering repeat is listed.

| **#** | **sequence (5’-3’)** | **length (nt)** | **units** | **inv rep** |
| --- | --- | --- | --- | --- |
|  | **upstream gene / downstream gene** |  |  |  |
| 014 | (GGGATTC)_5_(GGGAATT)_4_GGGAATCGGGAGTC(GGGAATC)_2_(GGGAGTC)_2_GGGAATC(GGGAGTC)_3_(GGGAATC)_3_GGGAGTCGGCGATTGGGGATTTGGGATTC | 183 | 26 | no |
|  | conserved, hypothetical protein (xcc0513) / *prmA*, 50S ribosomal protein L11 methyltransferase (xcc0512) |  |  |  |
| 120 | TGGAATT(GGGAATT)_6_GGGAATC(GGGAATT)_7_(GGGAATC)_8_ | 161 | 23 | no |
|  | *osmC*, osmotically inducible protein (xcc2745) / *pyrB*, aspartate carbamoyltransferase (xcc2746) |  |  |  |
| 155 | (GGGATTC)_6_GGGATC(GGGAATC)_11_ | 132 | 19 | no |
|  | *cebR*, transcriptional regulator (xcc3356) / *suc1*, sugar transporter(xcc3357) |  |  |  |
| 008 | (GGGAATC)_14_ | 98 | 14 | no |
|  | conserved, hypothetical protein (xcc0176) / *cls*, cardiolipin synthase (xcc0177) |  |  |  |
| 124 | (GGGAATC)_4_(GGGAGTC)_2_(GGGAATC)_3_GGGAGTC(GGGAATC)_3_GGGAAAA | 98 | 14 | 123 |
|  | acetyltransferase (xcc2770) / *nifS*, cysteine desulfurase (xcc2769) |  |  |  |
| 006 | (GGGGATT)_3_(GGGAATC)_8_ | 77 | 11 | no |
|  | *lldD*, L-lactate dehydrogenase (xcc0106) / ATP-dependent DNA ligase (xcc0105) |  |  |  |
| 034 | (GGGAATC)_10_ | 70 | 20 | no |
|  | *kdpD*, two-component system sensor protein (xcc0705) / *kdpC*, potassium-transporting ATPase subunit C (xcc0704) |  |  |  |
| 044 | (GGGATTG)_4_(GGGAATC)_5_GGGTGCA | 70 | 10 | no |
|  | voltage-gated potassium channel beta subunit (xcc0766) / *yeiM*, nucleoside transporter (xcc0765) |  |  |  |
| 183 | GGAAATC(GGGAATC)_4_(GGGAATG)_2_GGGAGTCGGGAATC | 63 | 9 | no |
|  | two-component system sensor protein (xcc4076) / *ndvB*, NdvB protein (xcc4077) |  |  |  |
| 053 | (GGGAATC)_7_GGGTAGA | 56 | 8 | no |
|  | *acnA*, aconitate hydratase (xcc1033) / *prpC*, methylcitrate synthase (xcc1032) |  |  |  |
| 112 | GAGATTCGGGAATC(GGGAAGC)_5_GGGAATC | 56 | 8 | no |
|  | maltose transporter gene repressor (xcc2464) / *cgt*, cyclomaltodextrin glucanotransferase (xcc2465) |  |  |  |
| 166 | GGGAATGGGGAGTCGGGAATGGGAAATGGGGAATCGGGAATGGGGAATCGGGATTC | 56 | 8 | no |
|  | conserved, hypothetical protein (xcc3710) / *yagR*, oxidoreductase (xcc3709) |  |  |  |

**S3 Table C: Longest Repeats in *Xac***

Sequence of the longest repeats (≥ 7 units) in Xac with G-tracts underscored. Total length, number of repeat units, locus tags and description of upstream and downstream neighboring genes are given. Occurrence of a repeat as part of the inverted repeat is shown (inv rep), and number of partnering repeat is listed.

| **#** | **sequence (5’-3’)** | **length (nt)** | **units** | **inv rep** |
| --- | --- | --- | --- | --- |
|  | **upstream gene / downstream gene** |  |  |  |
| 050 | CGGAATC(GGGAATC)_4_(GGGATTC)_12_GGGCAAT | 126 | 18 | no |
|  | *radA*, DNA repair protein (xac1263) / hypothetical protein (xac1262) |  |  |  |
| 092 | (GGGAATC)_7_GGGAAGCGGGAATCGGGAAGCGGGAATC(GGGAAGC)_4_ | 105 | 15 | 091 |
|  | *flgA*, flagellar basal body P-ring biosynthesis protein FlgA (xac1988) / *cheV*, chemotaxis protein (xac1987) |  |  |  |
| 037 | (GGGAATC)_3_(GGAAATC)_8_GGAAAAG | 84 | 12 | no |
|  | *oxyR*, oxidative stress transcriptional regulator (xac0905) / hypothetical protein (xac0904) |  |  |  |
| 087 | (GGGATTC)_5_GGGATTGGGATAATC(GGGAATC)_3_ | 71 | 10 | no |
|  | *cheA*, chemotaxis protein (xac1930) / *ISxac1*, ISxac1 transposase (xac1929) |  |  |  |
| 152 | (GGGAATC)_8_ | 56 | 8 | no |
|  | *cebR*, transcriptional regulator (xac3487) / *suc1*, sugar transporter (xac3488) |  |  |  |
| 008 | GAGAATC(GGGAATC)_6_ | 49 | 7 | no |
|  | lipid kinase (xac0475) / *trpE*, anthranilate synthase component I (xac0476) |  |  |  |
| 051 | (GGGATTG)_2_GCGAGTC(GGGAATC)_4_ | 49 | 7 | no |
|  | hypothetical protein (xac1288) / *ffh*, signal recognition particle protein (xac1289) |  |  |  |

**
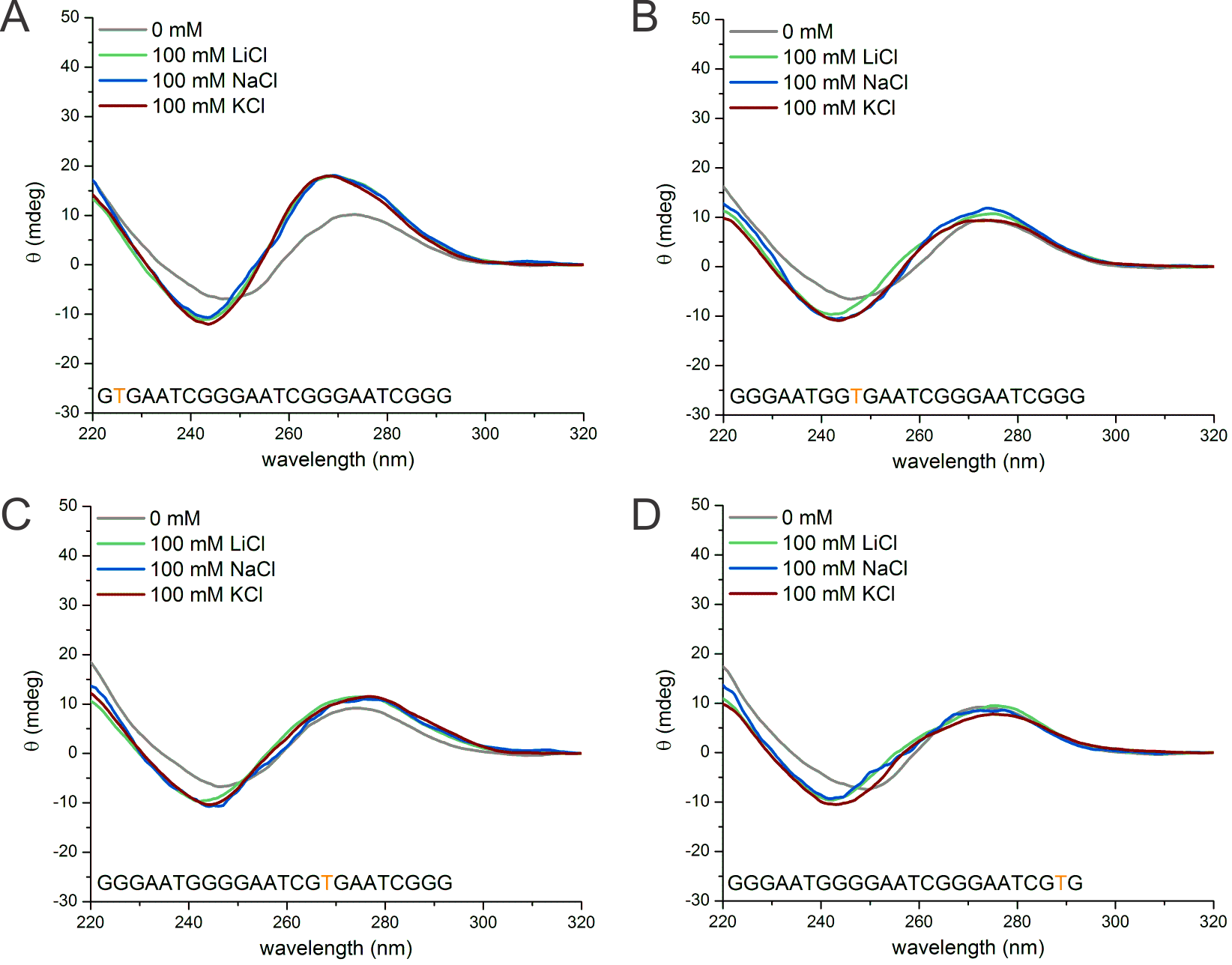
**

**S3 Figure A**: **CD spectra of (GGGAATC)_3_GGG variants with G to T mutations in G-tract.**

CD spectra were recorded from 220 to 320 nm of 5 μM oligonucleotide in 10 mM Tris-HCl (pH 7.5) in the presence of 100 mM LiCl (green), 100 mM NaCl (blue), 100 mM KCl (red) or tris buffer only (gray). Oligonucleotide sequences are shown in graphs. G to T muations for G-tract mutations are shown in orange. (**A)** (GGGAATC)_3_GGG with G to T mutation in first G-tract. (**B)** (GGGAATC)_3_GGG with G to T mutation in second G-tract (**C)** (GGGAATC)_3_GGG with G to T mutation in third G-tract. (**D)** (GGGAATC)_3_GGG with G to T mutation in fourth G-tract.

**S3 Table D**: **Melting temperatures of G-quadruplex structures**.
Temperature of the half-maximal decay of ellipticity T_1/2_ was determined at 290 nm unless otherwise indicated. Error shown is the error of the fit.

| **Sequence (5‘-3‘)** | **T_1/2_ 50 mM KCl** | **T_1/2_ 100 mM KCl** | **T_1/2_ 100 mM NaCl** |
| --- | --- | --- | --- |
| **(GGGAATC)_3_GGG** | 44.4°C ∓ 0.1 | 50.4°C ∓ 0.1 | 39.3°C ∓ 1.3 (260 nm) |
|  | 45.5°C ∓ 0.4 (270 nm) | 51.1°C ∓ 0.4 (270 nm) |  |
| **(GGGAATC)_4_** | 34.0°C ∓ 0.2 | 40.1°C∓ 0.1 | < 20°C |
|  | 35.9°C ∓ 0.5 (270 nm) | 40.5°C ∓ 0.5 (267 nm) |  |
| **(GGGGACT)_3_GGGG** | 92.6°C ∓ 0.3 | > 98°C | 59.1°C ∓ 0.2 |
| **(GGGGATT)_3_GGGG** | 88.6°C ∓ 0.4 | > 98°C | 59.7°C ∓ 0.1 |
| **(GGGACTG)_3_GGG** | 71.0°C ∓ 0.1 | 76.8°C ∓ 0.1 | 59.9°C ∓ 0.1 |
| **(GGGATTG)_3_GGG** | 69.3°C ∓ 0.1 | 74.6°C ∓ 0.1 | 58.9°C ∓ 0.1 |
| **(GGGGACT)_4_** | > 95°C | > 95°C | 56.6°C ∓ 0.2 |
| **(GGGGATT)_4_** | 87.9°C ∓ 0.1 | 92.9°C∓ 0.1 | 55.4°C∓ 0.1 |


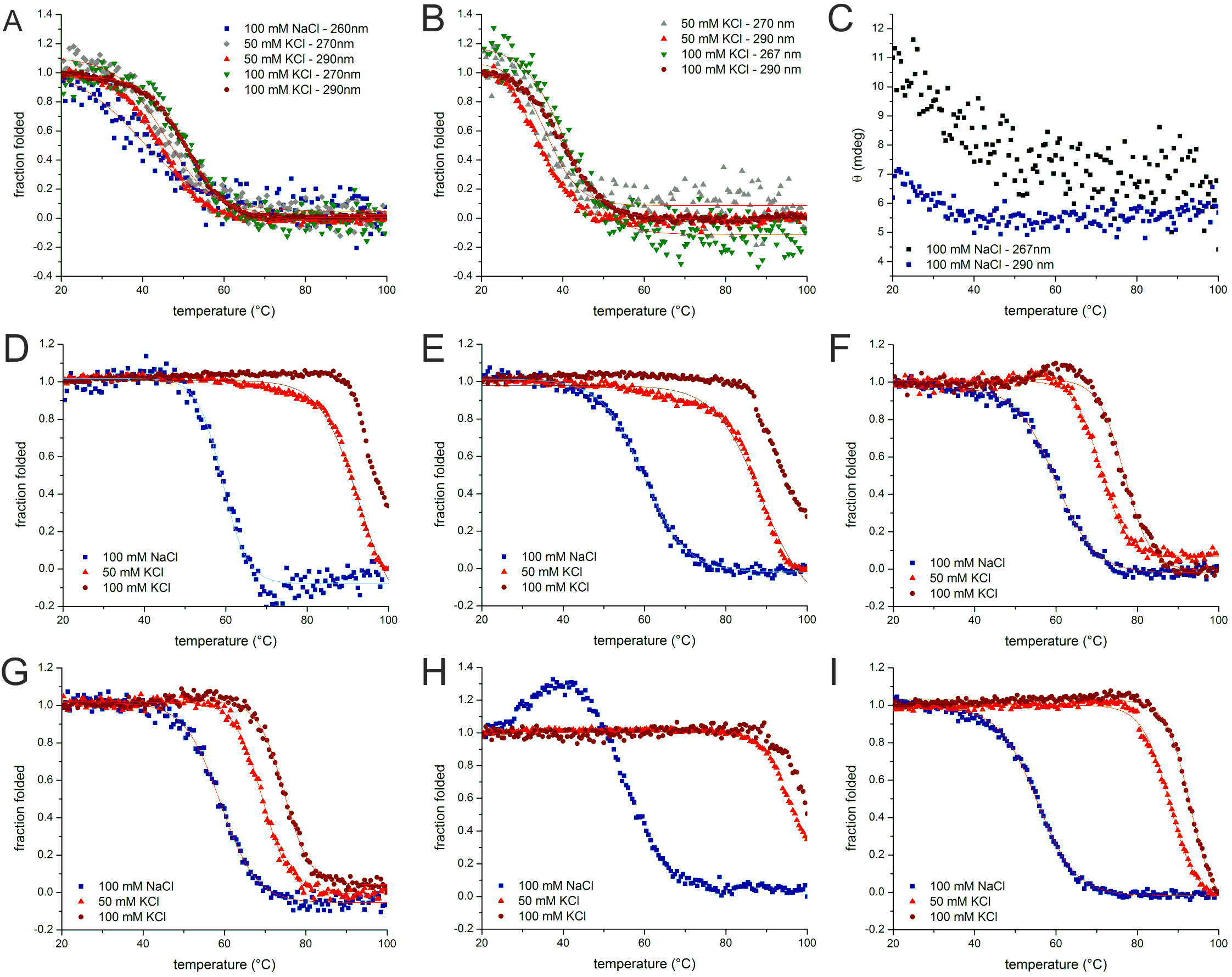


**S3 Figure B**: **Melting profiles of G-rich repeat oligos at pH 7.5.**

Melting profiles of 5 µM G-rich repeat oligonucleotides that showed G-quadruplex formation in CD in 10 mM Tris-HCl pH 7.5 in the prescence of different cations: 100 mM NaCl (blue squares), 50 mM KCl (light red triangles), 100 mM KCl (dark red circles). Samples were heated from 20°C to 100°C with a heating rate of 0.5°C/min. The CD signal was recorded every 0.5°C at 290 nm unless otherwise indicated. The temperature of the half-maximal decay of ellipticity T_1/2_ was obtained from the normalized ellipticity decrease using a Boltzmann sigmoidal fit. Fits are shown. **A**: (GGGAATC)_3_GGG; additionally shows melting profile in the presence of 100 mM KCl at 270 nm (black triangles). **B**: (GGGAATC)_4_; additionally shows melting profile in the presence of 100 mM KCl at 270 nm (black triangles). **C**: (GGGAATC)_4_; Melting profile in the presence of 100 mM NaCl recorded at 267 nm (black triangles) and 290 nm (blue squares). Structure melted immediately and T_1/2_ could not be determined accurately by sigmoidal fitting. **D**: (GGGGACT)_3_GGGG. **E:** (GGGGAAT)_3_GGGG. **F:** (GGGACTG)_3_GGG **G:** (GGGATTG)_3_GGG **H:** (GGGGACT)_4_; melting profile in the presence of 100 mM NaCl shows denaturation of a second less table structure between 20 and 40°C. Fit for determatination of T_1/2_ for the more stable structure listed in Table 3 was performed using data from 40-100°C only. **I:** (GGGGATT)_4_

**S3 Table E: Melting temperatures of structures formed by C-rich repeat oligonucleotides**

Melting temperatures were determined in 10 mM Na-acetate buffer at the indicated pH with or without additional salt. Temperature of the half-maximal decay of ellipticity T_1/2_ was determined at 285 nm unless otherwise indicated. Error shown is the error of the fit.

|  | **T_1/2_ [°C]** | | | | |
| --- | --- | --- | --- | --- | --- |
|  | **pH 4.5** | | | **pH 6.5** | |
| **Sequence** | **10 mM Na^+^** | **110 mM Na^+^** | **10 mM Na^+^ 100 mM K^+^** | **10 mM Na^+^** | **110 mM Na^+^** |
| **(CCCGATT)_3_CCC (280 nm)** | 64.04 ∓ 0.3 | 72.6 ∓ 0.3 | 72.1 ∓ 0.3 | 44.9 ∓ 0.2 | 37.0 ∓ 0.1 |
| **(GATTCCC)_4_ (280 nm)** | 60.9 ∓ 0.3 | 73.3 ∓ 0.3 | 73.4 ∓ 0.3 | 45.4 ∓ 0.1 | 40.5 ∓ 0.1 |
| **(CCCCAGT)_3_CCCC** | 70.0 ∓ 0.3 | 74.7 ∓ 0.2 | 74.5 ∓ 0.3 | 41.2 ∓ 0.1 | 30.1 ∓ 0.1 |
| **(CCCCAAT)_3_CCCC** | 71.8 ∓ 0.3 | 76.4 ∓ 0.2 | 76.7 ∓ 0.2 | 43.7 ∓ 0.1 | 33.9 ∓ 0.2 |
| **(CCCCAGT)_3_CCC** | 63.5 ∓ 0.2 | 67.7 ∓ 0.2 | 67.9 ∓ 0.2 | 36.4 ∓ 0.1 | 26.1 ∓ 0.5 |
| **(CCCCAAT)_3_CCC** | 65.4 ∓ 0.2 | 69.7 ∓ 0.2 | 69.6 ∓ 0.3 | 36.0 ∓ 0.1 | 24.9 ∓ 1.1 |
| **(AGTCCCC)_4_** | 67.7 ∓ 0.4 | 74.7 ∓ 0.5 | 73.8 ∓ 0.4 | 63.7 ∓ 0.3 | 30.7 ∓ 0.2 |
| **(AATCCCC)_4_** | 70.3 ∓ 0.5 | 78.4 ∓ 0.3 | 77.7 ∓ 0.2 | 48.3 ∓ 0.1 | 37.1 ∓ 0.1 |

**
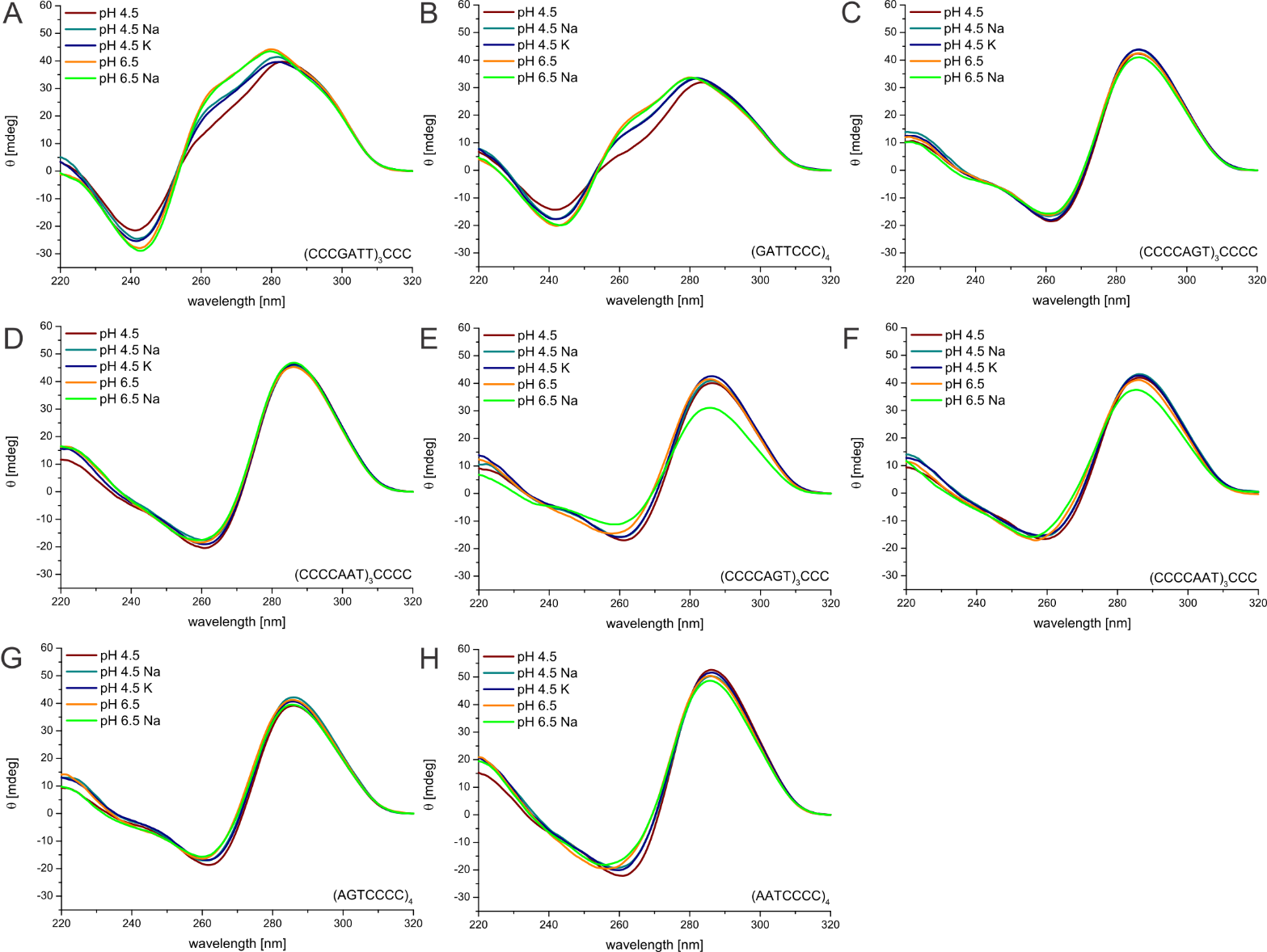
**

**S3 Figure C**: **CD spectra of C-rich repeat oligos in Na-acetate buffer**

CD spectra recorded from 220 to 320 nm of 5 μM oligonucleotide: (CCCGATT)_3_CCC (**A**), (GATTCCC)_4_ (**B**), (CCCCAGT)_3_CCCC (**C**), (CCCCAAT)_3_CCCC (**D**), (CCCCAGT)_3_CCC (**E**), (CCCCAAT)_3_CCC (**F**), (AGTCCCC)_4_ (**G**) and (AATCCCC)_4_ (**H**).Samples were prepared in 10 mM Na-acetate buffer pH 4.5 (red), pH 4.5 with additional 100 mM NaCl (light blue), pH 4.5 with additional 100 mM KCl (blue), pH 6.5 (orange), pH 6.5 with additional 100 mM NaCl (green).


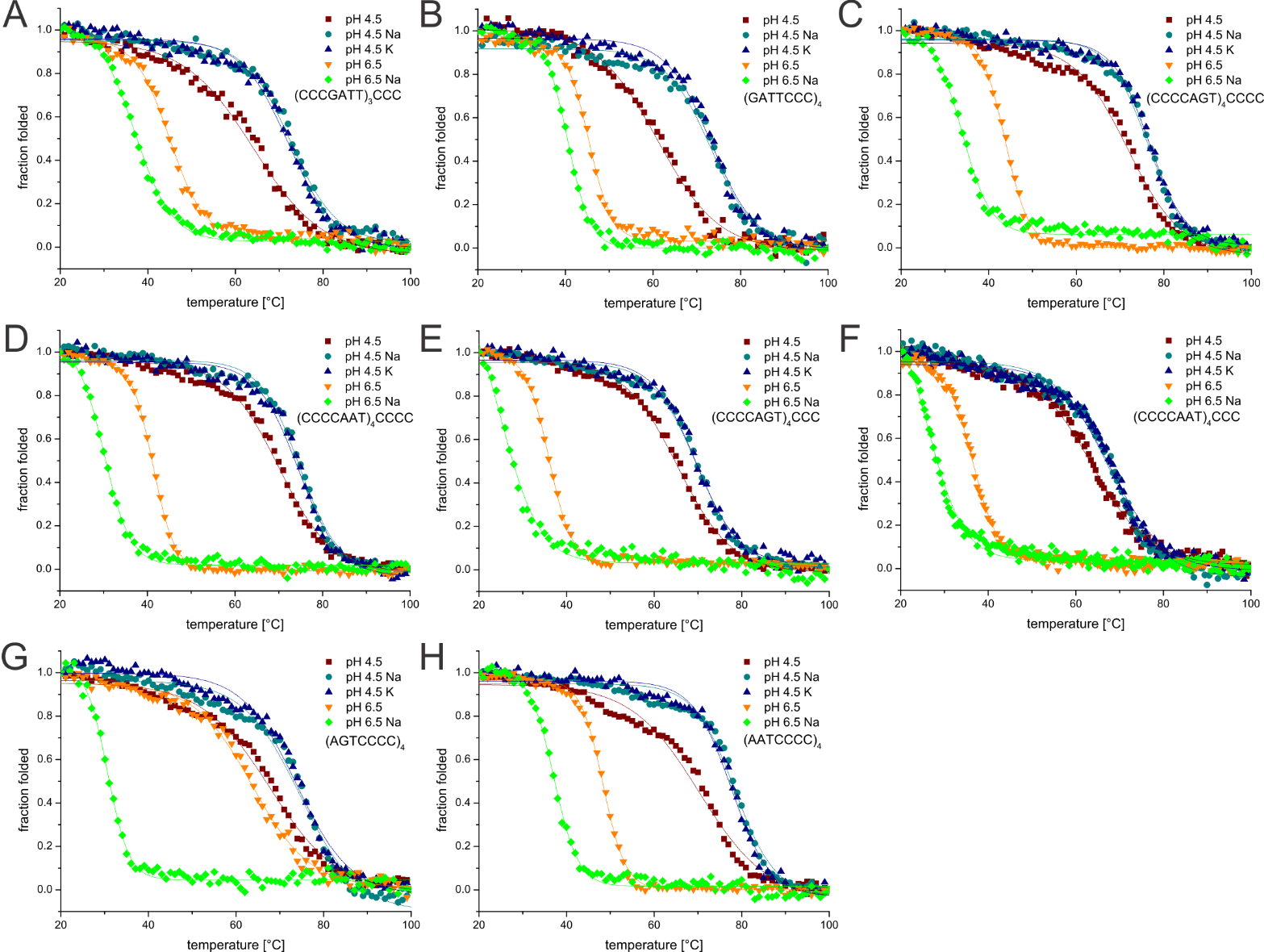


**S3 Figure D: Melting profiles of C-rich repeat oligos in Na-acetate buffer**

Melting profiles of 5 µM complementary C-rich oligonucleotides that showed i-motif formation in CD at pH 4.5: (CCCGATT)_3_CCC (**A**), (GATTCCC)_4_ (**B**), (CCCCAGT)_3_CCCC (**C**), (CCCCAAT)_3_CCCC (**D**), (CCCCAGT)_3_CCC (**E**), (CCCCAAT)_3_CCC (**F**), (AGTCCCC)_4_ (**G**) and (AATCCCC)_4_ (**H**).Samples were prepared in 10 mM Na-acetate buffer pH 4.5 (red), pH 4.5 with additional 100 mM NaCl (light blue), pH 4.5 with additional 100 mM KCl (blue), pH 6.5 (orange), pH 6.5 with additional 100 mM NaCl (green). All samples were heated from 20°C to 100°C with a heating rate of 0.5°C/min. The CD signal was recorded every 1.0°C at the indicated wavelength. The temperature of the half-maximal decay of ellipticity T_1/2_ was obtained from the normalized ellipticity decrease using a Boltzmann sigmoidal fit, ellipticity was recorded at 285 nm for C-H and 280 nm for A, B. Melting temperatures are shown in **SI3 Table S3.5**.

**
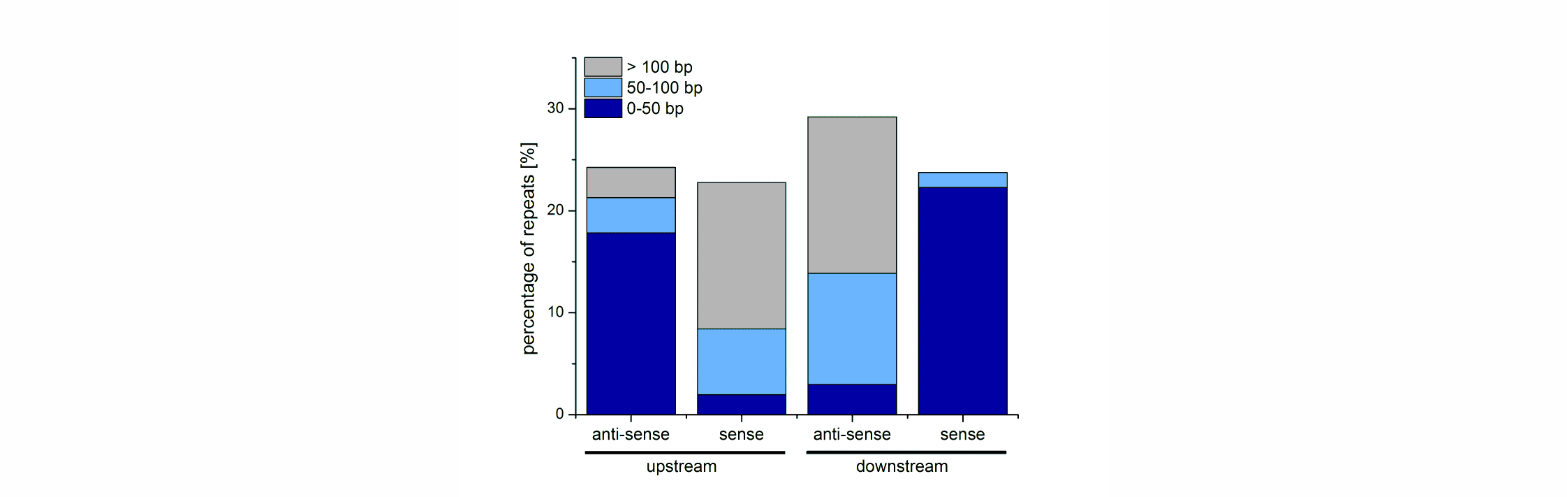
**

**S3 Figure E: Distance of repeats to neighboring ORFs for potential quadruplex forming sequences in Xcc**

Analysis of the distance of repeat sequences relative to adjacent ORFs for *Xcc* considering only repeats with ≥4 iterations and no point mutations in the G-tract. Repeats can be either located upstream or downstream of the next neighboring ORF. Repeats were grouped into three categories according to increasing distance from the respective: distance of >100 bp (gray), 50-100 bp (light blue) and repeats overlapping with ORFs or located in a distance of up to 50 bp from the respective start or stop codon are grouped together (dark blue).
