# Supplementary material for: Investigation of a Quadruplex-Forming Repeat Sequence Highly Enriched in Xanthomonas and Nostoc sp
Source: PLoS One. 2015 Dec 22;10(12):e0144275. doi: 10.1371/journal.pone.0144275 (PMC4692102; doi:10.1371/journal.pone.0144275)
Supplement: S4 File — (DOCX) [file pone.0144275.s004.docx]

**Supporting Information 4:**

**S4 Table**: Classification of repeat associated genes according to KEGG Pathways

(--, category not available)

|  | ***Xcc*** | ***Xac*** | ***Ana*** |
| --- | --- | --- | --- |
| **protein coding genes (total)** | 4170 | 4427 | 6129 |
| **RNA genes (total)** | 61 | 62 | 83 |
| **Repeat associated genes (total)** | 242 | 226 | 191 |
| **Not assigned** | 141 | 125 | 133 |
| **Assigned** | 101 | 101 | 58 |
| **KEGG pathway** |  |  |  |
| **Metabolism** | **71** | **66** | **49** |
| Global and overview maps | 67 | 59 | 45 |
| Carbohydrate metabolism | 24 | 18 | 10 |
| Energy metabolism | 13 | 10 | 14 |
| Lipid metabolism | 3 | 5 | 2 |
| Nucleotide metabolism | 15 | 18 | 4 |
| Amino acid metabolism | 15 | 16 | 15 |
| Metabolism of other amino acids | 12 | 5 | 5 |
| Glycan biosynthesis and metabolism | 5 | 7 | 3 |
| Metabolism of cofactors and vitamins | 11 | 8 | 13 |
| Metabolism of terpenoids and polyketides | 1 | 2 | 3 |
| Biosynthesis of other secondary metabolites | 1 | 0 | 0 |
| Xenobiotics biodegradation and metabolism | 0 | 1 | 1 |
| **Genetic Information Processing** | **20** | **24** | **5** |
| Transcription | 0 | 1 | 0 |
| Translation | 7 | 10 | 2 |
| Folding, sorting and degradation | 4 | 4 | 1 |
| Replication and repair | 9 | 9 | 2 |
| **Environmental Information Processing** | **12** | **12** | **7** |
| Membrane transport | 5 | 3 | 4 |
| Signal transduction | 7 | 9 | 3 |
| **Cellular Processes** | **5** | **6** | **0** |
| Cell motility | 5 | 6 | 0 |
| **Organismal Systems** | **1** | **0** | **--** |
| Environmental adaptation | 1 | 0 |  |
| **Human Diseases** | **0** | **1** | **0** |
| Drug resistance | 0 | 1 | 0 |

**Classification of repeat associated genes of Xcc according to KEGG Pathways**

Locus tag = intragenic, Locus tag = overlap with ORF

**XCC ATCC 33913: protein genes: 4170, RNA genes: 61 (4240)**

**186 repeats with 242 associated genes**

**Not found: 141 genes (55%)**

XCC0015, XCC0016, XCC0092, XCC0093, XCC0117, XCC0177, XCC0178, XCC0225, XCC0226, XCC0458, XCC0507, XCC0508, XCC0512, XCC0513, XCC0516, XCC0522, XCC0579, XCC0602, XCC0603, XCC0671, XCC0672, XCC0691, XCC0698, XCC0700, XCC0718, XCC0728, XCC0729, XCC0730, XCC0735, XCC0736, XCC0765, XCC0766, XCC0815, XCC0816, XCC0876, XCC0959, XCC0968, XCC1002, XCC1004, XCC1079, XCC1085, XCC1086, XCC1151, XCC1193, XCC1200, XCC1201, XCC1269, XCC1270, XCC1338, XCC1350, XCC1351, XCC1414, XCC1415, XCC1465, XCC1466, XCC1475, XCC1484, XCC1513, XCC1568, XCC1709, XCC1710, XCC1717, XCC1718, XCC1830, XCC1831, XCC1899, XCC1900, XCC1911, XCC1957, XCC1958, XCC2030, XCC2031, XCC2217, XCC2218, XCC2281, XCC2282, XCC2361, XCC2362, XCC2378, XCC2379, XCC2380, XCC2464, XCC2465, XCC2572, XCC2573, XCC2605, XCC2606, XCC2608, XCC2609, XCC2625, XCC2626, XCC2745, XCC2754, XCC2755, XCC2770, XCC2806, XCC2861, XCC3022, XCC3027, XCC3042, XCC3097, XCC3146, XCC3148, XCC3151, XCC3152, XCC3189, XCC3221, XCC3250, XCC3264, XCC3286, XCC3353, XCC3354, XCC3356, XCC3357, XCC3455, XCC3468, XCC3469, XCC3470, XCC3538, XCC3631, XCC3649, XCC3650, XCC3656, XCC3657, XCC3660, XCC3661, XCC3710, XCC3786, XCC3816, XCC3828, XCC3834, XCC3902, XCC3903, XCC3984, XCC4061, XCC4076, XCC4077, XCC4144, XCC4227, XCC4228, XCC4235

**HITS (101 genes):**

**Metabolism (71)**

Global and overview maps (67)

01100  [Metabolic pathways](http://www.genome.jp/kegg-bin/show_pathway?xcc01100) (63/629)

XCC0031, XCC0032, XCC0106, XCC0118, XCC0411, XCC0412, XCC0453, XCC0459,

XCC0517, XCC0552, XCC0553, XCC0554, XCC0555, XCC0648, XCC0649, XCC0656,

XCC0690, XCC0699, XCC0725, XCC0727, XCC0967, XCC1033, XCC1154, XCC1357,

XCC1358, XCC1360, XCC1361, XCC1485, XCC1486, XCC1512, XCC1569, XCC1575,

XCC2238, XCC2239, XCC2268, XCC2269, XCC2616, XCC2746, XCC2769, XCC2807,

XCC3171, XCC3172, XCC3173, XCC3174, XCC3188, XCC3220, XCC3249, XCC3265,

XCC3319, XCC3330, XCC3331, XCC3456, XCC3537, XCC3709, XCC3785, XCC3815,

XCC3829, XCC3835, XCC3847, XCC3980, XCC3985, XCC4060, XCC4143
01110  [Biosynthesis of secondary metabolites](http://www.genome.jp/kegg-bin/show_pathway?xcc01110) (28/270)

XCC0031, XCC0032, XCC0412, XCC0453, XCC0459, XCC0517, XCC0656, XCC0690,

XCC0967, XCC1033, XCC1357, XCC1485, XCC1486, XCC1512, XCC1569, XCC1575,

XCC2268, XCC2269, XCC3188, XCC3220, XCC3265, XCC3285, XCC3330, XCC3331,

XCC3537, XCC3835, XCC3980, XCC4060
01120  [Microbial metabolism in diverse environments](http://www.genome.jp/kegg-bin/show_pathway?xcc01120) (21/186)

XCC0031, XCC0032, XCC0118, XCC0517, XCC0690, XCC0967, XCC1033, XCC1357,

XCC1485, XCC1486, XCC2807, XCC3171, XCC3172, XCC3173, XCC3174, XCC3188,

XCC3220, XCC3265, XCC3355, XCC3709, XCC4060
01200  [Carbon metabolism](http://www.genome.jp/kegg-bin/show_pathway?xcc01200) (12/92)

XCC0118, XCC0517, XCC0690, XCC0967, XCC1033, XCC1357, XCC1485, XCC1486,

XCC3188, XCC3220, XCC3265, XCC4060
01210  [2-Oxocarboxylic acid metabolism](http://www.genome.jp/kegg-bin/show_pathway?xcc01210) (4/22)

XCC0967, XCC1033, XCC3330, XCC3331
01212  [Fatty acid metabolism](http://www.genome.jp/kegg-bin/show_pathway?xcc01212) (2/27)

XCC0517, XCC1357
01230  [Biosynthesis of amino acids](http://www.genome.jp/kegg-bin/show_pathway?xcc01230) (16/111)

XCC0031, XCC0032, XCC0459, XCC0690, XCC0967, XCC1033, XCC1512, XCC1569,

XCC2238, XCC2239, XCC3188, XCC3220, XCC3265, XCC3330, XCC3331, XCC3537
01220  [Degradation of aromatic compounds](http://www.genome.jp/kegg-bin/show_pathway?xcc01220) (0/15)

Carbohydrate metabolism (24)

00010  [Glycolysis / Gluconeogenesis](http://www.genome.jp/kegg-bin/show_pathway?xcc00010) (3/32)

XCC4060, XCC3188, XCC1485
00020  [Citrate cycle (TCA cycle)](http://www.genome.jp/kegg-bin/show_pathway?xcc00020) (4/25)

XCC0967, XCC1033, XCC1485, XCC1486
00030  [Pentose phosphate pathway](http://www.genome.jp/kegg-bin/show_pathway?xcc00030) (4/26)

XCC0118, XCC1575, XCC3220, XCC3265
00040  [Pentose and glucuronate interconversions](http://www.genome.jp/kegg-bin/show_pathway?xcc00040) (0/20)
00051  [Fructose and mannose metabolism](http://www.genome.jp/kegg-bin/show_pathway?xcc00051) (0/23)
00052  [Galactose metabolism](http://www.genome.jp/kegg-bin/show_pathway?xcc00052) (0/20)
00053  [Ascorbate and aldarate metabolism](http://www.genome.jp/kegg-bin/show_pathway?xcc00053) (1/12)

XCC2807
00500  [Starch and sucrose metabolism](http://www.genome.jp/kegg-bin/show_pathway?xcc00500) (2/48)

XCC0411, XCC0412
00520  [Amino sugar and nucleotide sugar metabolism](http://www.genome.jp/kegg-bin/show_pathway?xcc00520) (0/29)
00620  [Pyruvate metabolism](http://www.genome.jp/kegg-bin/show_pathway?xcc00620) (5/33)

XCC0106, XCC0517, XCC1357, XCC1485, XCC4060
00630  [Glyoxylate and dicarboxylate metabolism](http://www.genome.jp/kegg-bin/show_pathway?xcc00630) (4/27)

XCC0690, XCC1033, XCC2268, XCC3355
00640  [Propanoate metabolism](http://www.genome.jp/kegg-bin/show_pathway?xcc00640) (4/18)

XCC0517, XCC1032, XCC1357, XCC4060
00650  [Butanoate metabolism](http://www.genome.jp/kegg-bin/show_pathway?xcc00650) (1/24)

XCC3355
00660  [C5-Branched dibasic acid metabolism](http://www.genome.jp/kegg-bin/show_pathway?xcc00660) (2/8)

XCC3330, XCC3331
00562  [Inositol phosphate metabolism](http://www.genome.jp/kegg-bin/show_pathway?xcc00562) (0/7)

Energy metabolism (13)

00190  [Oxidative phosphorylation](http://www.genome.jp/kegg-bin/show_pathway?xcc00190) (5/51)

XCC0552, XCC0553, XCC0554, XCC0555, XCC3829
00680  [Methane metabolism](http://www.genome.jp/kegg-bin/show_pathway?xcc00680) (2/20)

XCC0690, XCC4060
00910  [Nitrogen metabolism](http://www.genome.jp/kegg-bin/show_pathway?xcc00910) (2/16)

XCC0031, XCC0032
00920  [Sulfur metabolism](http://www.genome.jp/kegg-bin/show_pathway?xcc00920) (4/19)

XCC3171, XCC3172, XCC3173, XCC3174

Lipid metabolism (3)

00061  [Fatty acid biosynthesis](http://www.genome.jp/kegg-bin/show_pathway?xcc00061) (2/18)

XCC0517, XCC1357
00071  [Fatty acid degradation](http://www.genome.jp/kegg-bin/show_pathway?xcc00071) (0/13)
00072  [Synthesis and degradation of ketone bodies](http://www.genome.jp/kegg-bin/show_pathway?xcc00072) (0/6)
00561  [Glycerolipid metabolism](http://www.genome.jp/kegg-bin/show_pathway?xcc00561) (0/10)
00564  [Glycerophospholipid metabolism](http://www.genome.jp/kegg-bin/show_pathway?xcc00564) (1/10)

XCC4234
00565  [Ether lipid metabolism](http://www.genome.jp/kegg-bin/show_pathway?xcc00565) (0/3)
00600  [Sphingolipid metabolism](http://www.genome.jp/kegg-bin/show_pathway?xcc00600) (0/4)
00590  [Arachidonic acid metabolism](http://www.genome.jp/kegg-bin/show_pathway?xcc00590) (0/7)
00591  [Linoleic acid metabolism](http://www.genome.jp/kegg-bin/show_pathway?xcc00591) (0/3)
00592  [alpha-Linolenic acid metabolism](http://www.genome.jp/kegg-bin/show_pathway?xcc00592) (0/2)
01040  [Biosynthesis of unsaturated fatty acids](http://www.genome.jp/kegg-bin/show_pathway?xcc01040) (0/9)

Nucleotide metabolism (15)

00230  [Purine metabolism](http://www.genome.jp/kegg-bin/show_pathway?xcc00230) (10/65)

XCC0453, XCC0648, XCC0656, XCC0960, XCC1358, XCC3171, XCC3172, XCC3249,

XCC3709, XCC3985
00240  [Pyrimidine metabolism](http://www.genome.jp/kegg-bin/show_pathway?xcc00240) (6/40)

XCC4143, XCC1358, XCC2746, XCC3847, XCC3985, XCC4143

Amino acid metabolism (15)

00250  [Alanine, aspartate and glutamate metabolism](http://www.genome.jp/kegg-bin/show_pathway?xcc00250) (4/23)

XCC0031, XCC0032, XCC2746, XCC3835
00260  [Glycine, serine and threonine metabolism](http://www.genome.jp/kegg-bin/show_pathway?xcc00260) (2/35)

XCC0690, XCC1485
00270  [Cysteine and methionine metabolism](http://www.genome.jp/kegg-bin/show_pathway?xcc00270) (1/29)

XCC1512
00280  [Valine, leucine and isoleucine degradation](http://www.genome.jp/kegg-bin/show_pathway?xcc00280) (1/21)

XCC1485
00290  [Valine, leucine and isoleucine biosynthesis](http://www.genome.jp/kegg-bin/show_pathway?xcc00290) (2/13)

XCC3330, XCC3331
00300  [Lysine biosynthesis](http://www.genome.jp/kegg-bin/show_pathway?xcc00300) (0/15)
00310  [Lysine degradation](http://www.genome.jp/kegg-bin/show_pathway?xcc00310) (1/10)

XCC1486

00330  [Arginine and proline metabolism](http://www.genome.jp/kegg-bin/show_pathway?xcc00330) (3/32)

XCC2238, XCC2239, XCC3835

00340  [Histidine metabolism](http://www.genome.jp/kegg-bin/show_pathway?xcc00340) (1/18)

XCC1569
00350  [Tyrosine metabolism](http://www.genome.jp/kegg-bin/show_pathway?xcc00350) (1/13)

XCC1569
00360  [Phenylalanine metabolism](http://www.genome.jp/kegg-bin/show_pathway?xcc00360) (1/13)

XCC1569
00380  [Tryptophan metabolism](http://www.genome.jp/kegg-bin/show_pathway?xcc00380) (0/19)
00400  [Phenylalanine, tyrosine and tryptophan biosynthesis](http://www.genome.jp/kegg-bin/show_pathway?xcc00400) (3/27)

XCC0459, XCC1569, XCC3537

Metabolism of other amino acids (12)

00410  [beta-Alanine metabolism](http://www.genome.jp/kegg-bin/show_pathway?xcc00410) (0/9)
00430  [Taurine and hypotaurine metabolism](http://www.genome.jp/kegg-bin/show_pathway?xcc00430) (0/5)

00440  [Phosphonate and phosphinate metabolism](http://www.genome.jp/kegg-bin/show_pathway?xcc00440) (0/3)
00450  [Selenocompound metabolism](http://www.genome.jp/kegg-bin/show_pathway?xcc00450) (5/11)

XCC1339, XCC1512, XCC2769, XCC3171, XCC3172
00460  [Cyanoamino acid metabolism](http://www.genome.jp/kegg-bin/show_pathway?xcc00460) (1/11)

XCC0690
00471  [D-Glutamine and D-glutamate metabolism](http://www.genome.jp/kegg-bin/show_pathway?xcc00471) (0/2)
00473  [D-Alanine metabolism](http://www.genome.jp/kegg-bin/show_pathway?xcc00473) (1/3)

XCC0727
00480  [Glutathione metabolism](http://www.genome.jp/kegg-bin/show_pathway?xcc00480) (1/26)

XCC0649

Glycan biosynthesis and metabolism (5)

00540  [Lipopolysaccharide biosynthesis](http://www.genome.jp/kegg-bin/show_pathway?xcc00540) (2/13)

XCC1360, XCC1361
00550  [Peptidoglycan biosynthesis](http://www.genome.jp/kegg-bin/show_pathway?xcc00550) (3/20)

XCC0725, XCC0727, XCC3456
00511  [Other glycan degradation](http://www.genome.jp/kegg-bin/show_pathway?xcc00511) (0/9)

Metabolism of cofactors and vitamins (11)

00730  [Thiamine metabolism](http://www.genome.jp/kegg-bin/show_pathway?xcc00730) (3/7)

XCC0699, XCC2769, XCC3319
00740  [Riboflavin metabolism](http://www.genome.jp/kegg-bin/show_pathway?xcc00740) (1/10)

XCC1154
00750  [Vitamin B6 metabolism](http://www.genome.jp/kegg-bin/show_pathway?xcc00750) (0/6)
00760  [Nicotinate and nicotinamide metabolism](http://www.genome.jp/kegg-bin/show_pathway?xcc00760) (1/11)

XCC2616
00770  [Pantothenate and CoA biosynthesis](http://www.genome.jp/kegg-bin/show_pathway?xcc00770) (1/14)

XCC3980
00780  [Biotin metabolism](http://www.genome.jp/kegg-bin/show_pathway?xcc00780) (0/16)
00785  [Lipoic acid metabolism](http://www.genome.jp/kegg-bin/show_pathway?xcc00785) (0/2)
00790  [Folate biosynthesis](http://www.genome.jp/kegg-bin/show_pathway?xcc00790) (2/18)

XCC3785, XCC3815
00670  [One carbon pool by folate](http://www.genome.jp/kegg-bin/show_pathway?xcc00670) (2/15)

XCC0690, XCC1512
00860  [Porphyrin and chlorophyll metabolism](http://www.genome.jp/kegg-bin/show_pathway?xcc00860) (0/27)
00130  [Ubiquinone and other terpenoid-quinone biosynthesis](http://www.genome.jp/kegg-bin/show_pathway?xcc00130) (1/11)

XCC2269

Metabolism of terpenoids and polyketides (1)

00900  [Terpenoid backbone biosynthesis](http://www.genome.jp/kegg-bin/show_pathway?xcc00900) (0/13)
00903  [Limonene and pinene degradation](http://www.genome.jp/kegg-bin/show_pathway?xcc00903) (0/5)
00281  [Geraniol degradation](http://www.genome.jp/kegg-bin/show_pathway?xcc00281) (1/7)

XCC3285
00523  [Polyketide sugar unit biosynthesis](http://www.genome.jp/kegg-bin/show_pathway?xcc00523) (0/5)
01054  [Nonribosomal peptide structures](http://www.genome.jp/kegg-bin/show_pathway?xcc01054) (0/1)
01053  [Biosynthesis of siderophore group nonribosomal peptides](http://www.genome.jp/kegg-bin/show_pathway?xcc01053) (0/2)

Biosynthesis of other secondary metabolites (1)

00311  [Penicillin and cephalosporin biosynthesis](http://www.genome.jp/kegg-bin/show_pathway?xcc00311) (0/4)
00521  [Streptomycin biosynthesis](http://www.genome.jp/kegg-bin/show_pathway?xcc00521) (0/11)

00401  [Novobiocin biosynthesis](http://www.genome.jp/kegg-bin/show_pathway?xcc00401) (1/5)

XCC1569

Xenobiotics biodegradation and metabolism (0)

00362  [Benzoate degradation](http://www.genome.jp/kegg-bin/show_pathway?xcc00362) (0/16)
00627  [Aminobenzoate degradation](http://www.genome.jp/kegg-bin/show_pathway?xcc00627) (0/15)
00364  [Fluorobenzoate degradation](http://www.genome.jp/kegg-bin/show_pathway?xcc00364) (0/3)
00625  [Chloroalkane and chloroalkene degradation](http://www.genome.jp/kegg-bin/show_pathway?xcc00625) (0/7)
00361  [Chlorocyclohexane and chlorobenzene degradation](http://www.genome.jp/kegg-bin/show_pathway?xcc00361) (0/4)
00623  [Toluene degradation](http://www.genome.jp/kegg-bin/show_pathway?xcc00623) (0/4)
00622  [Xylene degradation](http://www.genome.jp/kegg-bin/show_pathway?xcc00622) (0/2)
00633  [Nitrotoluene degradation](http://www.genome.jp/kegg-bin/show_pathway?xcc00633) (0/1)
00643  [Styrene degradation](http://www.genome.jp/kegg-bin/show_pathway?xcc00643) (0/6)
00930  [Caprolactam degradation](http://www.genome.jp/kegg-bin/show_pathway?xcc00930) (0/5)
00363  [Bisphenol degradation](http://www.genome.jp/kegg-bin/show_pathway?xcc00363) (0/3)
00626  [Naphthalene degradation](http://www.genome.jp/kegg-bin/show_pathway?xcc00626) (0/2)
00624  [Polycyclic aromatic hydrocarbon degradation](http://www.genome.jp/kegg-bin/show_pathway?xcc00624) (0/5)

**Genetic Information Processing (20)**

Transcription (0)

03020  [RNA polymerase](http://www.genome.jp/kegg-bin/show_pathway?xcc03020) (0/4)

Translation (7)

03010  [Ribosome](http://www.genome.jp/kegg-bin/show_pathway?xcc03010) (3/60)

XCC1150, XCC1564, XCC1565
00970  [Aminoacyl-tRNA biosynthesis](http://www.genome.jp/kegg-bin/show_pathway?xcc00970) (4/78)

XCC1155, XCC1339, XCC2458, XCC2459

Folding, sorting and degradation (4)

03060  [Protein export](http://www.genome.jp/kegg-bin/show_pathway?xcc03060) (2/16)

XCC1194, XCC1271
04122  [Sulfur relay system](http://www.genome.jp/kegg-bin/show_pathway?xcc04122) (0/7)
03018  [RNA degradation](http://www.genome.jp/kegg-bin/show_pathway?xcc03018) (2/15)

XCC0523, XCC1474

Replication and repair (9)

03030  [DNA replication](http://www.genome.jp/kegg-bin/show_pathway?xcc03030) (3/14)

XCC0648, XCC1358, XCC1359
03410  [Base excision repair](http://www.genome.jp/kegg-bin/show_pathway?xcc03410) (2/18)

XCC0105, XCC3846
03420  [Nucleotide excision repair](http://www.genome.jp/kegg-bin/show_pathway?xcc03420) (1/11)

XCC0105
03430  [Mismatch repair](http://www.genome.jp/kegg-bin/show_pathway?xcc03430) (3/19)

XCC0105, XCC0648, XCC1358
03440  [Homologous recombination](http://www.genome.jp/kegg-bin/show_pathway?xcc03440) (6/22)

XCC0648, XCC1003, XCC1358, XCC3023, XCC3025, XCC3026

03450  [Non-homologous end-joining](http://www.genome.jp/kegg-bin/show_pathway?xcc03450) (1/4)

XCC0105

**Environmental Information Processing (12)**

Membrane transport (5)

02010  [ABC transporters](http://www.genome.jp/kegg-bin/show_pathway?xcc02010) (1/47)

XCC3630
02060  [Phosphotransferase system (PTS)](http://www.genome.jp/kegg-bin/show_pathway?xcc02060) (1/5)

XCC2807
03070  [Bacterial secretion system](http://www.genome.jp/kegg-bin/show_pathway?xcc03070) (3/62)
 XCC0660, XCC0661, XCC1194

Signal transduction (7)

02020  [Two-component system](http://www.genome.jp/kegg-bin/show_pathway?xcc02020) (7/121)

XCC0704, XCC0705, XCC1953, XCC3096, XCC3352, XCC3788, XCC3789

04152  [AMPK signaling pathway](http://www.genome.jp/kegg-bin/show_pathway?xcc04152) (0/0)

**Cellular Processes (5)**

Cell motility (5)

02030  [Bacterial chemotaxis](http://www.genome.jp/kegg-bin/show_pathway?xcc02030) (1/49)

XCC1953
02040  [Flagellar assembly](http://www.genome.jp/kegg-bin/show_pathway?xcc02040) (4/34)

XCC1910, XCC1948, XCC1949, XCC1954

**Organismal Systems (1)**

Environmental adaptation (1)

04626  [Plant-pathogen interaction](http://www.genome.jp/kegg-bin/show_pathway?xcc04626) (1/8)

XCC0880

**Human Diseases (0)**

Drug resistance (-)

00312  [beta-Lactam resistance](http://www.genome.jp/kegg-bin/show_pathway?xcc00312) (0/15)

**Classification of repeat associated genes of Xac according to KEGG Pathways**

Locustag = intragenic, Locustag = overlapping with orf

**XAC strain 306: protein genes = 4427, RNA genes = 62 (4489)**

**183 repeats with 226 associated genes**

**Not found: 125 genes (55%)**

XAC0017, XAC0018, XAC0136, XAC0137, XAC0142, XAC0475, XAC0523, XAC0524, XAC0531, XAC0541, XAC0546, XAC0597, XAC0637, XAC0638, XAC0653, XAC0654, XAC0656, XAC0657, XAC0665, XAC0751, XAC0772, XAC0782, XAC0783, XAC0784, XAC0792, XAC0904, XAC0905, XAC0953, XAC0957, XAC1038, XAC1047, XAC1110, XAC1112, XAC1183, XAC1184, XAC1250, XAC1262, XAC1263, XAC1288, XAC1302, XAC1321, XAC1322, XAC1329, XAC1385, XAC1387, XAC1397, XAC1398, XAC1418, XAC1423, XAC1458, XAC1459, XAC1512, XAC1513, XAC1532, XAC1570, XAC1571, XAC1728, XAC1729, XAC1843, XAC1849, XAC1850, XAC1919, XAC1929, XAC1938, XAC1991, XAC1992, XAC2321, XAC2322, XAC2338, XAC2340, XAC2389, XAC2390, XAC2468, XAC2513, XAC2514, XAC2515, XAC2766, XAC2767, XAC2769, XAC2770, XAC2789, XAC2790, XAC2915, XAC2923, XAC2924, XAC2927, XAC2939, XAC2966, XAC3112, XAC3151, XAC3153, XAC3239, XAC3303, XAC3304, XAC3353, XAC3373, XAC3387, XAC3396, XAC3421, XAC3432, XAC3484, XAC3485, XAC3487, XAC3488, XAC3532, XAC3533, XAC3550, XAC3696, XAC3697, XAC3700, XAC3701, XAC3847, XAC3871, XAC3883, XAC3985, XAC3986, XAC3998, XAC3999, XAC4073, XAC4180, XAC4361, XAC4362, XAC4365, XAC4366, XAC4368

**HITS (101 genes):**

**Metabolism (66)**

Global and overview maps (59)

01100  [Metabolic pathways](http://www.genome.jp/kegg-bin/show_pathway?xcc01100) (56/632)

XAC0143, XAC0476, XAC0532, XAC0545, XAC0598, XAC0664, XAC0752,

XAC0779, XAC0781, XAC0965, XAC1046, XAC1405, XAC1406, XAC1408,

XAC1409, XAC1419, XAC1533, XAC1534, XAC1844, XAC2012, XAC2013,

XAC2287, XAC2288, XAC2342, XAC2343, XAC2351, XAC2469, XAC2778,

XAC2916, XAC2926, XAC2938, XAC2965, XAC3328, XAC3329, XAC3330,

XAC3331, XAC3344, XAC3345, XAC3352, XAC3372, XAC3386, XAC3390,

XAC3395, XAC3420, XAC3457, XAC3458, XAC3549, XAC3558, XAC3650,

XAC3651, XAC3846, XAC3870, XAC3884, XAC3903, XAC4074, XAC4179
01110  [Biosynthesis of secondary metabolites](http://www.genome.jp/kegg-bin/show_pathway?xcc01110) (22/273)

XAC0476, XAC0532, XAC0545, XAC0598, XAC1046, XAC1405, XAC1533,

XAC1534, XAC2012, XAC2288, XAC2351, XAC2926, XAC3344, XAC3345,

XAC3352, XAC3372, XAC3420, XAC3431, XAC3457, XAC3458, XAC3549,

XAC4179,
01120  [Microbial metabolism in diverse environments](http://www.genome.jp/kegg-bin/show_pathway?xcc01120) (20/184)

XAC0143, XAC0532, XAC1046, XAC1405, XAC1533, XAC1534, XAC1844,

XAC2012, XAC2013, XAC2469, XAC3328, XAC3329, XAC3330, XAC3331,

XAC3344, XAC3345, XAC3352, XAC3372, XAC3486, XAC4179
01200  [Carbon metabolism](http://www.genome.jp/kegg-bin/show_pathway?xcc01200) (14/89)

XAC0141, XAC0143, XAC0532, XAC1046, XAC1405, XAC1533, XAC1534,

XAC1844, XAC2013, XAC3344, XAC3345, XAC3352, XAC3372, XAC4179
01210  [2-Oxocarboxylic acid metabolism](http://www.genome.jp/kegg-bin/show_pathway?xcc01210) (3/22)

XAC1046, XAC3457, XAC3458

01212  [Fatty acid metabolism](http://www.genome.jp/kegg-bin/show_pathway?xcc01212) (4/29)

XAC0532, XAC1405, XAC2012, XAC2013

01230  [Biosynthesis of amino acids](http://www.genome.jp/kegg-bin/show_pathway?xcc01230) (15/110)

XAC0476, XAC0545, XAC0598, XAC1046, XAC1844, XAC2342, XAC2343,

XAC2351, XAC2926, XAC3344, XAC3345, XAC3352, XAC3372, XAC3457,

XAC3458
01220  [Degradation of aromatic compounds](http://www.genome.jp/kegg-bin/show_pathway?xcc01220) (0/15)

Carbohydrate metabolism (18)

00010  [Glycolysis / Gluconeogenesis](http://www.genome.jp/kegg-bin/show_pathway?xcc00010) (5/34)

XAC1533, XAC3344, XAC3345, XAC3352, XAC4179
00020  [Citrate cycle (TCA cycle)](http://www.genome.jp/kegg-bin/show_pathway?xcc00020) (3/24)

XAC1046, XAC1533, XAC1534

00030  [Pentose phosphate pathway](http://www.genome.jp/kegg-bin/show_pathway?xcc00030) (3/26)

XAC0143, XAC3344, XAC3372
00040  [Pentose and glucuronate interconversions](http://www.genome.jp/kegg-bin/show_pathway?xcc00040) (0/21)
00051  [Fructose and mannose metabolism](http://www.genome.jp/kegg-bin/show_pathway?xcc00051) (1/24)

XAC3344
00052  [Galactose metabolism](http://www.genome.jp/kegg-bin/show_pathway?xcc00052) (0/23)
00053  [Ascorbate and aldarate metabolism](http://www.genome.jp/kegg-bin/show_pathway?xcc00053) (0/11)

00500  [Starch and sucrose metabolism](http://www.genome.jp/kegg-bin/show_pathway?xcc00500) (0/48)
00520  [Amino sugar and nucleotide sugar metabolism](http://www.genome.jp/kegg-bin/show_pathway?xcc00520) (1/30)

XAC2965
00620  [Pyruvate metabolism](http://www.genome.jp/kegg-bin/show_pathway?xcc00620) (5/33)

XAC0532, XAC1405, XAC1533, XAC3345, XAC4179
00630  [Glyoxylate and dicarboxylate metabolism](http://www.genome.jp/kegg-bin/show_pathway?xcc00630) (2/27)

XAC0324, XAC3486
00640  [Propanoate metabolism](http://www.genome.jp/kegg-bin/show_pathway?xcc00640) (3/19)

XAC0532, XAC1405, XAC4179
00650  [Butanoate metabolism](http://www.genome.jp/kegg-bin/show_pathway?xcc00650) (3/24)

XAC0141, XAC2469, XAC3486
00660  [C5-Branched dibasic acid metabolism](http://www.genome.jp/kegg-bin/show_pathway?xcc00660) (2/8)

XAC3457, XAC3458
00562  [Inositol phosphate metabolism](http://www.genome.jp/kegg-bin/show_pathway?xcc00562) (0/9)

Energy metabolism (10)

00190  [Oxidative phosphorylation](http://www.genome.jp/kegg-bin/show_pathway?xcc00190) (3/50)

XAC3650, XAC3651, XAC3884
00680  [Methane metabolism](http://www.genome.jp/kegg-bin/show_pathway?xcc00680) (3/18)

XAC1844, XAC3344, XAC4179
00910  [Nitrogen metabolism](http://www.genome.jp/kegg-bin/show_pathway?xcc00910) (0/11)

00920  [Sulfur metabolism](http://www.genome.jp/kegg-bin/show_pathway?xcc00920) (4/27)

XAC3328, XAC3329, XAC3330, XAC3331

Lipid metabolism (5)

00061  [Fatty acid biosynthesis](http://www.genome.jp/kegg-bin/show_pathway?xcc00061) (2/20)

XAC0532, XAC1405
00071  [Fatty acid degradation](http://www.genome.jp/kegg-bin/show_pathway?xcc00071) (2/15)

XAC2012, XAC2013
00072  [Synthesis and degradation of ketone bodies](http://www.genome.jp/kegg-bin/show_pathway?xcc00072) (0/6)
00561  [Glycerolipid metabolism](http://www.genome.jp/kegg-bin/show_pathway?xcc00561) (0/11)
00564  [Glycerophospholipid metabolism](http://www.genome.jp/kegg-bin/show_pathway?xcc00564) (1/23)

XAC4367
00565  [Ether lipid metabolism](http://www.genome.jp/kegg-bin/show_pathway?xcc00565) (0/3)
00600  [Sphingolipid metabolism](http://www.genome.jp/kegg-bin/show_pathway?xcc00600) (0/3)
00590  [Arachidonic acid metabolism](http://www.genome.jp/kegg-bin/show_pathway?xcc00590) (0/7)
00591  [Linoleic acid metabolism](http://www.genome.jp/kegg-bin/show_pathway?xcc00591) (0/4)
00592  [alpha-Linolenic acid metabolism](http://www.genome.jp/kegg-bin/show_pathway?xcc00592) (1/2)

XAC2012
01040  [Biosynthesis of unsaturated fatty acids](http://www.genome.jp/kegg-bin/show_pathway?xcc01040) (0/10)

Nucleotide metabolism (18)

00230  [Purine metabolism](http://www.genome.jp/kegg-bin/show_pathway?xcc00230) (15/64)

XAC0965, XAC1039, XAC1330, XAC1406, XAC2287, XAC2288, XAC3113,

XAC3328, XAC3329, XAC3345, XAC3390, XAC3395, XAC3549, XAC3558,

XAC4074
00240  [Pyrimidine metabolism](http://www.genome.jp/kegg-bin/show_pathway?xcc00240) (7/39)

XAC0965, XAC1406, XAC1419, XAC2916, XAC3558, XAC3903, XAC4074

Amino acid metabolism (16)

00250  [Alanine, aspartate and glutamate metabolism](http://www.genome.jp/kegg-bin/show_pathway?xcc00250) (3/25)

XAC2351, XAC2469, XAC2916
00260  [Glycine, serine and threonine metabolism](http://www.genome.jp/kegg-bin/show_pathway?xcc00260) (0/33)
00270  [Cysteine and methionine metabolism](http://www.genome.jp/kegg-bin/show_pathway?xcc00270) (2/30)

XAC1533, XAC1844
00280  [Valine, leucine and isoleucine degradation](http://www.genome.jp/kegg-bin/show_pathway?xcc00280) (2/22)

XAC1533, XAC2012

00290  [Valine, leucine and isoleucine biosynthesis](http://www.genome.jp/kegg-bin/show_pathway?xcc00290) (2/13)

XAC3457, XAC3458
00300  [Lysine biosynthesis](http://www.genome.jp/kegg-bin/show_pathway?xcc00300) (0/15)

00310  [Lysine degradation](http://www.genome.jp/kegg-bin/show_pathway?xcc00310) (2/11)

XAC1534, XAC2469
00330  [Arginine and proline metabolism](http://www.genome.jp/kegg-bin/show_pathway?xcc00330) (5/35)

XAC2342, XAC2343, XAC2351, XAC2352, XAC2926
00340  [Histidine metabolism](http://www.genome.jp/kegg-bin/show_pathway?xcc00340) (0/21)

00350  [Tyrosine metabolism](http://www.genome.jp/kegg-bin/show_pathway?xcc00350) (1/16)

XAC2469
00360  [Phenylalanine metabolism](http://www.genome.jp/kegg-bin/show_pathway?xcc00360) (0/13)

00380  [Tryptophan metabolism](http://www.genome.jp/kegg-bin/show_pathway?xcc00380) (0/20)
00400  [Phenylalanine, tyrosine and tryptophan biosynthesis](http://www.genome.jp/kegg-bin/show_pathway?xcc00400) (3/27)

XAC0476, XAC0545, XAC0598

Metabolism of other amino acids (5)

00410  [beta-Alanine metabolism](http://www.genome.jp/kegg-bin/show_pathway?xcc00410) (0/10)
00430  [Taurine and hypotaurine metabolism](http://www.genome.jp/kegg-bin/show_pathway?xcc00430) (0/5)

00440  [Phosphonate and phosphinate metabolism](http://www.genome.jp/kegg-bin/show_pathway?xcc00440) (0/3)
00450  [Selenocompound metabolism](http://www.genome.jp/kegg-bin/show_pathway?xcc00450) (4/11)

XAC1386, XAC2938, XAC3328, XAC3329
00460  [Cyanoamino acid metabolism](http://www.genome.jp/kegg-bin/show_pathway?xcc00460) (0/10)

00471  [D-Glutamine and D-glutamate metabolism](http://www.genome.jp/kegg-bin/show_pathway?xcc00471) (0/2)
00473  [D-Alanine metabolism](http://www.genome.jp/kegg-bin/show_pathway?xcc00473) (1/2)

XAC0781
00480  [Glutathione metabolism](http://www.genome.jp/kegg-bin/show_pathway?xcc00480) (0/29)

Glycan biosynthesis and metabolism (7)

00540  [Lipopolysaccharide biosynthesis](http://www.genome.jp/kegg-bin/show_pathway?xcc00540) (2/13)

XAC1408, XAC1409
00550  [Peptidoglycan biosynthesis](http://www.genome.jp/kegg-bin/show_pathway?xcc00550) (5/19)

XAC0664, XAC0779, XAC0781, XAC2965, XAC3386
00511  [Other glycan degradation](http://www.genome.jp/kegg-bin/show_pathway?xcc00511) (0/10)

Metabolism of cofactors and vitamins (8)

00730  [Thiamine metabolism](http://www.genome.jp/kegg-bin/show_pathway?xcc00730) (2/7)

XAC0752, XAC2938
00740  [Riboflavin metabolism](http://www.genome.jp/kegg-bin/show_pathway?xcc00740) (0/13)

00750  [Vitamin B6 metabolism](http://www.genome.jp/kegg-bin/show_pathway?xcc00750) (0/6)
00760  [Nicotinate and nicotinamide metabolism](http://www.genome.jp/kegg-bin/show_pathway?xcc00760) (1/15)

XAC2778, XAC3390
00770  [Pantothenate and CoA biosynthesis](http://www.genome.jp/kegg-bin/show_pathway?xcc00770) (0/14)

00780  [Biotin metabolism](http://www.genome.jp/kegg-bin/show_pathway?xcc00780) (0/17)
00785  [Lipoic acid metabolism](http://www.genome.jp/kegg-bin/show_pathway?xcc00785) (0/2)
00790  [Folate biosynthesis](http://www.genome.jp/kegg-bin/show_pathway?xcc00790) (2/19)

XAC3846, XAC3870
00670  [One carbon pool by folate](http://www.genome.jp/kegg-bin/show_pathway?xcc00670) (1/15)

XAC0324
00860  [Porphyrin and chlorophyll metabolism](http://www.genome.jp/kegg-bin/show_pathway?xcc00860) (1/28)

XAC3420
00130  [Ubiquinone and other terpenoid-quinone biosynthesis](http://www.genome.jp/kegg-bin/show_pathway?xcc00130) (0/12)

Metabolism of terpenoids and polyketides (2)

00900  [Terpenoid backbone biosynthesis](http://www.genome.jp/kegg-bin/show_pathway?xcc00900) (0/12)
00903  [Limonene and pinene degradation](http://www.genome.jp/kegg-bin/show_pathway?xcc00903) (0/6)
00281  [Geraniol degradation](http://www.genome.jp/kegg-bin/show_pathway?xcc00281) (2/7)

XAC2012, XAC3431
00523  [Polyketide sugar unit biosynthesis](http://www.genome.jp/kegg-bin/show_pathway?xcc00523) (0/4)
01054  [Nonribosomal peptide structures](http://www.genome.jp/kegg-bin/show_pathway?xcc01054) (0/1)
01053  [Biosynthesis of siderophore group nonribosomal peptides](http://www.genome.jp/kegg-bin/show_pathway?xcc01053) (0/0)

Biosynthesis of other secondary metabolites (0)

00311  [Penicillin and cephalosporin biosynthesis](http://www.genome.jp/kegg-bin/show_pathway?xcc00311) (0/4)
00521  [Streptomycin biosynthesis](http://www.genome.jp/kegg-bin/show_pathway?xcc00521) (0/10)
00401  [Novobiocin biosynthesis](http://www.genome.jp/kegg-bin/show_pathway?xcc00401) (0/5)

Xenobiotics biodegradation and metabolism (1)

00362  [Benzoate degradation](http://www.genome.jp/kegg-bin/show_pathway?xcc00362) (1/16)

XAC2012
00627  [Aminobenzoate degradation](http://www.genome.jp/kegg-bin/show_pathway?xcc00627) (0/18)
00364  [Fluorobenzoate degradation](http://www.genome.jp/kegg-bin/show_pathway?xcc00364) (0/1)
00625  [Chloroalkane and chloroalkene degradation](http://www.genome.jp/kegg-bin/show_pathway?xcc00625) (0/10)
00361  [Chlorocyclohexane and chlorobenzene degradation](http://www.genome.jp/kegg-bin/show_pathway?xcc00361) (0/)2
00623  [Toluene degradation](http://www.genome.jp/kegg-bin/show_pathway?xcc00623) (0/2)
00622  [Xylene degradation](http://www.genome.jp/kegg-bin/show_pathway?xcc00622) (0/2)
00633  [Nitrotoluene degradation](http://www.genome.jp/kegg-bin/show_pathway?xcc00633) (0/1)
00643  [Styrene degradation](http://www.genome.jp/kegg-bin/show_pathway?xcc00643) (0/6)
00930  [Caprolactam degradation](http://www.genome.jp/kegg-bin/show_pathway?xcc00930) (0/4)
00363  [Bisphenol degradation](http://www.genome.jp/kegg-bin/show_pathway?xcc00363) (0/3)
00626  [Naphthalene degradation](http://www.genome.jp/kegg-bin/show_pathway?xcc00626) (0/3)
00624  [Polycyclic aromatic hydrocarbon degradation](http://www.genome.jp/kegg-bin/show_pathway?xcc00624) (0/7)

**Genetic Information Processing (24)**

Transcription (1)

03020  [RNA polymerase](http://www.genome.jp/kegg-bin/show_pathway?xcc03020) (1/4)

XAC0965

Translation (10)

03010  [Ribosome](http://www.genome.jp/kegg-bin/show_pathway?xcc03010) (5/60)

XAC0964, XAC1249, XAC1422, XAC1621, XAC1622
00970  [Aminoacyl-tRNA biosynthesis](http://www.genome.jp/kegg-bin/show_pathway?xcc00970) (5/79)

XAC1386, XAC2589, XAC2590, XAC3154, XAC3559

Folding, sorting and degradation (4)

03060  [Protein export](http://www.genome.jp/kegg-bin/show_pathway?xcc03060) (3/16)

XAC1255, XAC1289, XAC1323
04122  [Sulfur relay system](http://www.genome.jp/kegg-bin/show_pathway?xcc04122) (0/7)
03018  [RNA degradation](http://www.genome.jp/kegg-bin/show_pathway?xcc03018) (1/15)

XAC0542

Replication and repair (9)

03030  [DNA replication](http://www.genome.jp/kegg-bin/show_pathway?xcc03030) (3/15)

XAC1406, XAC1407, XAC3558
03410  [Base excision repair](http://www.genome.jp/kegg-bin/show_pathway?xcc03410) (1/17)

XAC3902
03420  [Nucleotide excision repair](http://www.genome.jp/kegg-bin/show_pathway?xcc03420) (0/10)

03430  [Mismatch repair](http://www.genome.jp/kegg-bin/show_pathway?xcc03430) (3/19)

XAC1303, XAC1406, XAC3558
03440  [Homologous recombination](http://www.genome.jp/kegg-bin/show_pathway?xcc03440) (6/23)

XAC1111, XAC1406, XAC3149, XAC3150, XAC3391, XAC3558
03450  [Non-homologous end-joining](http://www.genome.jp/kegg-bin/show_pathway?xcc03450) (0/3)

**Environmental Information Processing (12)**

Membrane transport (3)

02010  [ABC transporters](http://www.genome.jp/kegg-bin/show_pathway?xcc02010) (0/56)

02060  [Phosphotransferase system (PTS)](http://www.genome.jp/kegg-bin/show_pathway?xcc02060) (0/5)

03070  [Bacterial secretion system](http://www.genome.jp/kegg-bin/show_pathway?xcc03070) (3/76)
 XAC1289, XAC3543, XAC3544

Signal transduction (9)

02020  [Two-component system](http://www.genome.jp/kegg-bin/show_pathway?xcc02020) (9/125)

XAC0325, XAC0758, XAC0759, XAC1930, XAC1987, XAC3238, XAC3483,

XAC3849, XAC3850

04152  [AMPK signaling pathway](http://www.genome.jp/kegg-bin/show_pathway?xcc04152) (0/0)

**Cellular Processes (6)**

Cell motility (6)

02030  [Bacterial chemotaxis](http://www.genome.jp/kegg-bin/show_pathway?xcc02030) (2/48)

XAC1930, XAC1987

02040  [Flagellar assembly](http://www.genome.jp/kegg-bin/show_pathway?xcc02040) (4/34)

XAC1937, XAC1982, XAC1983, XAC1988

**Organismal Systems (0)**

Environmental adaptation (0)

04626  [Plant-pathogen interaction](http://www.genome.jp/kegg-bin/show_pathway?xcc04626) (0/0)

**Human Diseases (1)**

Drug resistance (1)

00312  [beta-Lactam resistance](http://www.genome.jp/kegg-bin/show_pathway?xcc00312) (1/14)

XAC3386

**Classification of repeat associated genes of Ana according to KEGG Pathways**

Locustag = intragenic, Locustag = overlapping with ORF

**Nostoc sp. PCC7120: protein genes: 6129, RNA genes: 83 (6212)**

**89 repeats with 191 associated genes**

**Not found: 133 genes (69%)**

all0144, all0268, all0479, all1221, all1325, all1342, all1380, all1826, all2007, all2641, all2643, all2644, all2645, all2647, all2649, all2804, all2813, all3209, all3526, all3563, all4109, all4355, all4389, all4445, all5023, all5305, alr0076, alr0309, alr0548, alr0730, alr0787, alr1096, all1237, alr1259, alr1337, alr1675, alr1874, alr1901, alr2373, alr2588, alr2814, alr2836, alr2947, alr3057, alr3183, alr3539, alr3562, alr3671, alr3811, alr4240, alr4268, alr4646, alr4890, asl0260, asl0272, asl2101, asl4372, asl4860, asr0798, asr2427, asr3342, asr3935, all0164, all0261, all0273, all0307, all1154, all1326, all1673, all1875, all2008, all2103, all2642, all3049, all3184, all3564, all3673, all3933, all4357, all4374, all4446, all4647, all4744, all5022, all5137, alr0075, alr0110, alr0236, alr0267, alr0547, alr0549, alr0728, alr0789, alr1093, alr1222, alr1238, alr1336, alr1920, alr2372, alr2429, alr2589, alr2802, alr2812, alr2835, alr2948, alr3210, alr3229, alr3565, alr3812, alr4239, alr4537, alr4611, alr4888, asl1825, asl4266, asl4862, asr3886, asr4108, all0478, all0729, all2102, all2646, all2648, allrs04, alr1381, alr1674, alr2428, alr4267, alr4373, asl4356, asl4889, asr2273, asr4612

**HITS (58 genes)**

**Metabolism (49)**

Global and overview maps (45)

01100  [Metabolic pathways](http://www.kegg.jp/kegg-bin/show_pathway?ana01100) (45/667)
[all0109](http://www.kegg.jp/dbget-bin/www_bget?ana:all0109), [all0143](http://www.kegg.jp/dbget-bin/www_bget?ana:all0143), [all0166](http://www.kegg.jp/dbget-bin/www_bget?ana:all0166), [all0328](http://www.kegg.jp/dbget-bin/www_bget?ana:all0328), [all0329](http://www.kegg.jp/dbget-bin/www_bget?ana:all0329), [all0788](http://www.kegg.jp/dbget-bin/www_bget?ana:all0788), [all0797](http://www.kegg.jp/dbget-bin/www_bget?ana:all0797), [all0948](http://www.kegg.jp/dbget-bin/www_bget?ana:all0948), [all0949](http://www.kegg.jp/dbget-bin/www_bget?ana:all0949), [all3538](http://www.kegg.jp/dbget-bin/www_bget?ana:all3538), [all3569](http://www.kegg.jp/dbget-bin/www_bget?ana:all3569), [all4390](http://www.kegg.jp/dbget-bin/www_bget?ana:all4390), [all4538](http://www.kegg.jp/dbget-bin/www_bget?ana:all4538), [all4613](http://www.kegg.jp/dbget-bin/www_bget?ana:all4613), [all4861](http://www.kegg.jp/dbget-bin/www_bget?ana:all4861), [all5138](http://www.kegg.jp/dbget-bin/www_bget?ana:all5138), [alr0237](http://www.kegg.jp/dbget-bin/www_bget?ana:alr0237), [alr0308](http://www.kegg.jp/dbget-bin/www_bget?ana:alr0308), [alr0477](http://www.kegg.jp/dbget-bin/www_bget?ana:alr0477), [alr0951](http://www.kegg.jp/dbget-bin/www_bget?ana:alr0951), [alr0952](http://www.kegg.jp/dbget-bin/www_bget?ana:alr0952), [alr1041](http://www.kegg.jp/dbget-bin/www_bget?ana:alr1041), [alr1042](http://www.kegg.jp/dbget-bin/www_bget?ana:alr1042), [alr1095](http://www.kegg.jp/dbget-bin/www_bget?ana:alr1095), [alr1155](http://www.kegg.jp/dbget-bin/www_bget?ana:alr1155), [alr1343](http://www.kegg.jp/dbget-bin/www_bget?ana:alr1343), [alr1900](http://www.kegg.jp/dbget-bin/www_bget?ana:alr1900), [alr1921](http://www.kegg.jp/dbget-bin/www_bget?ana:alr1921), [alr2272](http://www.kegg.jp/dbget-bin/www_bget?ana:alr2272), [alr2274](http://www.kegg.jp/dbget-bin/www_bget?ana:alr2274), [alr2803](http://www.kegg.jp/dbget-bin/www_bget?ana:alr2803), [alr2811](http://www.kegg.jp/dbget-bin/www_bget?ana:alr2811), [alr3050](http://www.kegg.jp/dbget-bin/www_bget?ana:alr3050), [alr3056](http://www.kegg.jp/dbget-bin/www_bget?ana:alr3056), [alr3230](http://www.kegg.jp/dbget-bin/www_bget?ana:alr3230), [alr3343](http://www.kegg.jp/dbget-bin/www_bget?ana:alr3343), [alr3524](http://www.kegg.jp/dbget-bin/www_bget?ana:alr3524), [alr3525](http://www.kegg.jp/dbget-bin/www_bget?ana:alr3525), [alr3537](http://www.kegg.jp/dbget-bin/www_bget?ana:alr3537), [alr3672](http://www.kegg.jp/dbget-bin/www_bget?ana:alr3672), [alr3887](http://www.kegg.jp/dbget-bin/www_bget?ana:alr3887), [alr3934](http://www.kegg.jp/dbget-bin/www_bget?ana:alr3934), [alr4745](http://www.kegg.jp/dbget-bin/www_bget?ana:alr4745), [alr4746](http://www.kegg.jp/dbget-bin/www_bget?ana:alr4746)

01110  [Biosynthesis of secondary metabolites](http://www.kegg.jp/kegg-bin/show_pathway?ana01110) (25/291)
[all0328](http://www.kegg.jp/dbget-bin/www_bget?ana:all0328), [all0788](http://www.kegg.jp/dbget-bin/www_bget?ana:all0788), [all0797](http://www.kegg.jp/dbget-bin/www_bget?ana:all0797), [all0948](http://www.kegg.jp/dbget-bin/www_bget?ana:all0948), [all0949](http://www.kegg.jp/dbget-bin/www_bget?ana:all0949), [all3538](http://www.kegg.jp/dbget-bin/www_bget?ana:all3538), [all3569](http://www.kegg.jp/dbget-bin/www_bget?ana:all3569), [all4390](http://www.kegg.jp/dbget-bin/www_bget?ana:all4390), [all4538](http://www.kegg.jp/dbget-bin/www_bget?ana:all4538), [all4613](http://www.kegg.jp/dbget-bin/www_bget?ana:all4613), [alr0308](http://www.kegg.jp/dbget-bin/www_bget?ana:alr0308), [alr1041](http://www.kegg.jp/dbget-bin/www_bget?ana:alr1041), [alr1042](http://www.kegg.jp/dbget-bin/www_bget?ana:alr1042), [alr1095](http://www.kegg.jp/dbget-bin/www_bget?ana:alr1095), [alr2811](http://www.kegg.jp/dbget-bin/www_bget?ana:alr2811), [alr3050](http://www.kegg.jp/dbget-bin/www_bget?ana:alr3050), [alr3056](http://www.kegg.jp/dbget-bin/www_bget?ana:alr3056), [alr3230](http://www.kegg.jp/dbget-bin/www_bget?ana:alr3230), [alr3524](http://www.kegg.jp/dbget-bin/www_bget?ana:alr3524), [alr3525](http://www.kegg.jp/dbget-bin/www_bget?ana:alr3525), [alr3537](http://www.kegg.jp/dbget-bin/www_bget?ana:alr3537), [alr3672](http://www.kegg.jp/dbget-bin/www_bget?ana:alr3672), [alr3887](http://www.kegg.jp/dbget-bin/www_bget?ana:alr3887), [alr4745](http://www.kegg.jp/dbget-bin/www_bget?ana:alr4745), [alr4746](http://www.kegg.jp/dbget-bin/www_bget?ana:alr4746)

01120  [Microbial metabolism in diverse environments](http://www.kegg.jp/kegg-bin/show_pathway?ana01120) (8/151)
[all3538](http://www.kegg.jp/dbget-bin/www_bget?ana:all3538), [all4861](http://www.kegg.jp/dbget-bin/www_bget?ana:all4861), [all5138](http://www.kegg.jp/dbget-bin/www_bget?ana:all5138), [alr1041](http://www.kegg.jp/dbget-bin/www_bget?ana:alr1041), [alr1095](http://www.kegg.jp/dbget-bin/www_bget?ana:alr1095), [alr2803](http://www.kegg.jp/dbget-bin/www_bget?ana:alr2803), [alr3672](http://www.kegg.jp/dbget-bin/www_bget?ana:alr3672), [alr4745](http://www.kegg.jp/dbget-bin/www_bget?ana:alr4745)

01200  [Carbon metabolism](http://www.kegg.jp/kegg-bin/show_pathway?ana01200) (6/87)
[all3538](http://www.kegg.jp/dbget-bin/www_bget?ana:all3538), [all4861](http://www.kegg.jp/dbget-bin/www_bget?ana:all4861), [alr1041](http://www.kegg.jp/dbget-bin/www_bget?ana:alr1041), [alr1095](http://www.kegg.jp/dbget-bin/www_bget?ana:alr1095), [alr2803](http://www.kegg.jp/dbget-bin/www_bget?ana:alr2803), [alr4745](http://www.kegg.jp/dbget-bin/www_bget?ana:alr4745)

01210  [2-Oxocarboxylic acid metabolism](http://www.kegg.jp/kegg-bin/show_pathway?ana01210) (2/24)
[all4613](http://www.kegg.jp/dbget-bin/www_bget?ana:all4613), [alr3537](http://www.kegg.jp/dbget-bin/www_bget?ana:alr3537)

01212  [Fatty acid metabolism](http://www.kegg.jp/kegg-bin/show_pathway?ana01212) (1/16)
[alr3343](http://www.kegg.jp/dbget-bin/www_bget?ana:alr3343)

01230  [Biosynthesis of amino acids](http://www.kegg.jp/kegg-bin/show_pathway?ana01230) (12/121)
[all0328](http://www.kegg.jp/dbget-bin/www_bget?ana:all0328), [all0797](http://www.kegg.jp/dbget-bin/www_bget?ana:all0797), [all3538](http://www.kegg.jp/dbget-bin/www_bget?ana:all3538), [all4390](http://www.kegg.jp/dbget-bin/www_bget?ana:all4390), [all4613](http://www.kegg.jp/dbget-bin/www_bget?ana:all4613), [alr0308](http://www.kegg.jp/dbget-bin/www_bget?ana:alr0308), [alr1095](http://www.kegg.jp/dbget-bin/www_bget?ana:alr1095), [alr3050](http://www.kegg.jp/dbget-bin/www_bget?ana:alr3050), [alr3056](http://www.kegg.jp/dbget-bin/www_bget?ana:alr3056), [alr3537](http://www.kegg.jp/dbget-bin/www_bget?ana:alr3537), [alr3887](http://www.kegg.jp/dbget-bin/www_bget?ana:alr3887), [alr4746](http://www.kegg.jp/dbget-bin/www_bget?ana:alr4746)

01220  [Degradation of aromatic compounds](http://www.kegg.jp/kegg-bin/show_pathway?ana01220) (0/3)

Carbohydrate metabolism (10)

00010  [Glycolysis / Gluconeogenesis](http://www.kegg.jp/kegg-bin/show_pathway?ana00010) (5/33)

[all3538](http://www.kegg.jp/dbget-bin/www_bget?ana:all3538), [alr1041](http://www.kegg.jp/dbget-bin/www_bget?ana:alr1041), [alr1095](http://www.kegg.jp/dbget-bin/www_bget?ana:alr1095), [alr3672](http://www.kegg.jp/dbget-bin/www_bget?ana:alr3672), [alr4745](http://www.kegg.jp/dbget-bin/www_bget?ana:alr4745)

00020  [Citrate cycle (TCA cycle)](http://www.kegg.jp/kegg-bin/show_pathway?ana00020) (1/15)

[alr4745](http://www.kegg.jp/dbget-bin/www_bget?ana:alr4745)

00030  [Pentose phosphate pathway](http://www.kegg.jp/kegg-bin/show_pathway?ana00030) (1/23)

[alr1041](http://www.kegg.jp/dbget-bin/www_bget?ana:alr1041)

00040  [Pentose and glucuronate interconversions](http://www.kegg.jp/kegg-bin/show_pathway?ana00040) (1/7)

[alr3672](http://www.kegg.jp/dbget-bin/www_bget?ana:alr3672)

00051  [Fructose and mannose metabolism](http://www.kegg.jp/kegg-bin/show_pathway?ana00051) (2/20)

[all4538](http://www.kegg.jp/dbget-bin/www_bget?ana:all4538), [alr1041](http://www.kegg.jp/dbget-bin/www_bget?ana:alr1041)

00052  [Galactose metabolism](http://www.kegg.jp/kegg-bin/show_pathway?ana00052) (0/10)

00053  [Ascorbate and aldarate metabolism](http://www.kegg.jp/kegg-bin/show_pathway?ana00053) (1/3)

[alr3672](http://www.kegg.jp/dbget-bin/www_bget?ana:alr3672)

00500  [Starch and sucrose metabolism](http://www.kegg.jp/kegg-bin/show_pathway?ana00500) (1/27)

[all0166](http://www.kegg.jp/dbget-bin/www_bget?ana:all0166)

00520  [Amino sugar and nucleotide sugar metabolism](http://www.kegg.jp/kegg-bin/show_pathway?ana00520) (2/33)

[all4538](http://www.kegg.jp/dbget-bin/www_bget?ana:all4538), [alr1900](http://www.kegg.jp/dbget-bin/www_bget?ana:alr1900)

00620  [Pyruvate metabolism](http://www.kegg.jp/kegg-bin/show_pathway?ana00620) (3/32)

[all4861](http://www.kegg.jp/dbget-bin/www_bget?ana:all4861), [alr3672](http://www.kegg.jp/dbget-bin/www_bget?ana:alr3672), [alr4745](http://www.kegg.jp/dbget-bin/www_bget?ana:alr4745)

00630  [Glyoxylate and dicarboxylate metabolism](http://www.kegg.jp/kegg-bin/show_pathway?ana00630) (0/20)

00640  [Propanoate metabolism](http://www.kegg.jp/kegg-bin/show_pathway?ana00640) (0/15)

00650  [Butanoate metabolism](http://www.kegg.jp/kegg-bin/show_pathway?ana00650) (1/8)

[all4613](http://www.kegg.jp/dbget-bin/www_bget?ana:all4613)

00660  [C5-Branched dibasic acid metabolism](http://www.kegg.jp/kegg-bin/show_pathway?ana00660) (1/8)

[all4613](http://www.kegg.jp/dbget-bin/www_bget?ana:all4613)

00562  [Inositol phosphate metabolism](http://www.kegg.jp/kegg-bin/show_pathway?ana00562) (0/5)

Energy metabolism (14)

00190  [Oxidative phosphorylation](http://www.kegg.jp/kegg-bin/show_pathway?ana00190) (5/56)

[all0948](http://www.kegg.jp/dbget-bin/www_bget?ana:all0948), [all0949](http://www.kegg.jp/dbget-bin/www_bget?ana:all0949), [all3570](http://www.kegg.jp/dbget-bin/www_bget?ana:all3570), [alr0951](http://www.kegg.jp/dbget-bin/www_bget?ana:alr0951), [alr0952](http://www.kegg.jp/dbget-bin/www_bget?ana:alr0952)

00195  [Photosynthesis](http://www.kegg.jp/kegg-bin/show_pathway?ana00195) (2/75)

[all0109](http://www.kegg.jp/dbget-bin/www_bget?ana:all0109), [all0329](http://www.kegg.jp/dbget-bin/www_bget?ana:all0329)

00196  [Photosynthesis - antenna proteins](http://www.kegg.jp/kegg-bin/show_pathway?ana00196) (2/22)

[alr0526](http://www.kegg.jp/dbget-bin/www_bget?ana:alr0526), [alr0527](http://www.kegg.jp/dbget-bin/www_bget?ana:alr0527)

00710  [Carbon fixation in photosynthetic organisms](http://www.kegg.jp/kegg-bin/show_pathway?ana00710) (3/19)

[all4861](http://www.kegg.jp/dbget-bin/www_bget?ana:all4861), [alr1041](http://www.kegg.jp/dbget-bin/www_bget?ana:alr1041), [alr1095](http://www.kegg.jp/dbget-bin/www_bget?ana:alr1095)

00680  [Methane metabolism](http://www.kegg.jp/kegg-bin/show_pathway?ana00680) (3/30)

[all3538](http://www.kegg.jp/dbget-bin/www_bget?ana:all3538), [all4861](http://www.kegg.jp/dbget-bin/www_bget?ana:all4861), [alr1041](http://www.kegg.jp/dbget-bin/www_bget?ana:alr1041)

00910  [Nitrogen metabolism](http://www.kegg.jp/kegg-bin/show_pathway?ana00910) (0/20)

00920  [Sulfur metabolism](http://www.kegg.jp/kegg-bin/show_pathway?ana00920) (1/18)

[all5138](http://www.kegg.jp/dbget-bin/www_bget?ana:all5138)

Lipid metabolism (2)

00061  [Fatty acid biosynthesis](http://www.kegg.jp/kegg-bin/show_pathway?ana00061) (1/12)

[alr3343](http://www.kegg.jp/dbget-bin/www_bget?ana:alr3343)

00071  [Fatty acid degradation](http://www.kegg.jp/kegg-bin/show_pathway?ana00071) (1/5)

[alr3672](http://www.kegg.jp/dbget-bin/www_bget?ana:alr3672)

00100  [Steroid biosynthesis](http://www.kegg.jp/kegg-bin/show_pathway?ana00100) (0/1)

00561  [Glycerolipid metabolism](http://www.kegg.jp/kegg-bin/show_pathway?ana00561) (1/12)

[alr3672](http://www.kegg.jp/dbget-bin/www_bget?ana:alr3672)

00564  [Glycerophospholipid metabolism](http://www.kegg.jp/kegg-bin/show_pathway?ana00564) (0/10)

01040  [Biosynthesis of unsaturated fatty acids](http://www.kegg.jp/kegg-bin/show_pathway?ana01040) (0/5)

Nucleotide metabolism (4)

00230  [Purine metabolism](http://www.kegg.jp/kegg-bin/show_pathway?ana00230) (3/63)

[all0788](http://www.kegg.jp/dbget-bin/www_bget?ana:all0788), [all5138](http://www.kegg.jp/dbget-bin/www_bget?ana:all5138), [alr3525](http://www.kegg.jp/dbget-bin/www_bget?ana:alr3525)

00240  [Pyrimidine metabolism](http://www.kegg.jp/kegg-bin/show_pathway?ana00240) (1/43)

[alr1155](http://www.kegg.jp/dbget-bin/www_bget?ana:alr1155)

Amino acid metabolism (15)

00250  [Alanine, aspartate and glutamate metabolism](http://www.kegg.jp/kegg-bin/show_pathway?ana00250) (2/43)

[alr1155](http://www.kegg.jp/dbget-bin/www_bget?ana:alr1155), [alr3887](http://www.kegg.jp/dbget-bin/www_bget?ana:alr3887)

00260  [Glycine, serine and threonine metabolism](http://www.kegg.jp/kegg-bin/show_pathway?ana00260) (1/30)

[alr4745](http://www.kegg.jp/dbget-bin/www_bget?ana:alr4745)

00270  [Cysteine and methionine metabolism](http://www.kegg.jp/kegg-bin/show_pathway?ana00270) (1/26)

[alr0308](http://www.kegg.jp/dbget-bin/www_bget?ana:alr0308)

00280  [Valine, leucine and isoleucine degradation](http://www.kegg.jp/kegg-bin/show_pathway?ana00280) (2/6)

[alr3672](http://www.kegg.jp/dbget-bin/www_bget?ana:alr3672), [alr4745](http://www.kegg.jp/dbget-bin/www_bget?ana:alr4745)

00290  [Valine, leucine and isoleucine biosynthesis](http://www.kegg.jp/kegg-bin/show_pathway?ana00290) (2/14)

[all4613](http://www.kegg.jp/dbget-bin/www_bget?ana:all4613), [alr2811](http://www.kegg.jp/dbget-bin/www_bget?ana:alr2811)

00300  [Lysine biosynthesis](http://www.kegg.jp/kegg-bin/show_pathway?ana00300) (0/14)

00310  [Lysine degradation](http://www.kegg.jp/kegg-bin/show_pathway?ana00310) (1/6)

[alr3672](http://www.kegg.jp/dbget-bin/www_bget?ana:alr3672)

00330  [Arginine and proline metabolism](http://www.kegg.jp/kegg-bin/show_pathway?ana00330) (3/33)

[alr3537](http://www.kegg.jp/dbget-bin/www_bget?ana:alr3537), [alr3672](http://www.kegg.jp/dbget-bin/www_bget?ana:alr3672), [alr3887](http://www.kegg.jp/dbget-bin/www_bget?ana:alr3887)

00340  [Histidine metabolism](http://www.kegg.jp/kegg-bin/show_pathway?ana00340) (3/14)

[all4390](http://www.kegg.jp/dbget-bin/www_bget?ana:all4390), [alr3056](http://www.kegg.jp/dbget-bin/www_bget?ana:alr3056), [alr3672](http://www.kegg.jp/dbget-bin/www_bget?ana:alr3672)

00350  [Tyrosine metabolism](http://www.kegg.jp/kegg-bin/show_pathway?ana00350) (0/8)

00360  [Phenylalanine metabolism](http://www.kegg.jp/kegg-bin/show_pathway?ana00360) (0/8)

00380  [Tryptophan metabolism](http://www.kegg.jp/kegg-bin/show_pathway?ana00380) (1/4)

[alr3672](http://www.kegg.jp/dbget-bin/www_bget?ana:alr3672)

00400  [Phenylalanine, tyrosine and tryptophan biosynthesis](http://www.kegg.jp/kegg-bin/show_pathway?ana00400) (4/31)

[all0328](http://www.kegg.jp/dbget-bin/www_bget?ana:all0328), [all0797](http://www.kegg.jp/dbget-bin/www_bget?ana:all0797), [alr3050](http://www.kegg.jp/dbget-bin/www_bget?ana:alr3050), [alr4746](http://www.kegg.jp/dbget-bin/www_bget?ana:alr4746)

Metabolism of other amino acids (5)

00410  [beta-Alanine metabolism](http://www.kegg.jp/kegg-bin/show_pathway?ana00410) (2/6)

[all3569](http://www.kegg.jp/dbget-bin/www_bget?ana:all3569), [alr3672](http://www.kegg.jp/dbget-bin/www_bget?ana:alr3672)

00430  [Taurine and hypotaurine metabolism](http://www.kegg.jp/kegg-bin/show_pathway?ana00430) (0/3)

00440  [Phosphonate and phosphinate metabolism](http://www.kegg.jp/kegg-bin/show_pathway?ana00440) (0/8)

00450  [Selenocompound metabolism](http://www.kegg.jp/kegg-bin/show_pathway?ana00450)

[all5138](http://www.kegg.jp/dbget-bin/www_bget?ana:all5138), [alr0308](http://www.kegg.jp/dbget-bin/www_bget?ana:alr0308)

00460  [Cyanoamino acid metabolism](http://www.kegg.jp/kegg-bin/show_pathway?ana00460) (0/4)

00471  [D-Glutamine and D-glutamate metabolism](http://www.kegg.jp/kegg-bin/show_pathway?ana00471) (0/5)
00473  [D-Alanine metabolism](http://www.kegg.jp/kegg-bin/show_pathway?ana00473) (0/2)
00480  [Glutathione metabolism](http://www.kegg.jp/kegg-bin/show_pathway?ana00480) (1/17)

[alr0237](http://www.kegg.jp/dbget-bin/www_bget?ana:alr0237)

Glycan biosynthesis and metabolism (3)

00540  [Lipopolysaccharide biosynthesis](http://www.kegg.jp/kegg-bin/show_pathway?ana00540) (2/5)

[alr2272](http://www.kegg.jp/dbget-bin/www_bget?ana:alr2272), [alr2274](http://www.kegg.jp/dbget-bin/www_bget?ana:alr2274)

00550  [Peptidoglycan biosynthesis](http://www.kegg.jp/kegg-bin/show_pathway?ana00550) (1/17)

[alr0477](http://www.kegg.jp/dbget-bin/www_bget?ana:alr0477)

00511  [Other glycan degradation](http://www.kegg.jp/kegg-bin/show_pathway?ana00511) (0/2)

Metabolism of cofactors and vitamins (13)

00730  [Thiamine metabolism](http://www.kegg.jp/kegg-bin/show_pathway?ana00730) (2/10)

[all3540](http://www.kegg.jp/dbget-bin/www_bget?ana:all3540), [alr1343](http://www.kegg.jp/dbget-bin/www_bget?ana:alr1343)

00740  [Riboflavin metabolism](http://www.kegg.jp/kegg-bin/show_pathway?ana00740) (0/7)
00750  [Vitamin B6 metabolism](http://www.kegg.jp/kegg-bin/show_pathway?ana00750) (0/6)
00760  [Nicotinate and nicotinamide metabolism](http://www.kegg.jp/kegg-bin/show_pathway?ana00760) (0/17)
00770  [Pantothenate and CoA biosynthesis](http://www.kegg.jp/kegg-bin/show_pathway?ana00770) (2/14)

[all3569](http://www.kegg.jp/dbget-bin/www_bget?ana:all3569), [all4613](http://www.kegg.jp/dbget-bin/www_bget?ana:all4613)

00780  [Biotin metabolism](http://www.kegg.jp/kegg-bin/show_pathway?ana00780) (2/12)

[alr1921](http://www.kegg.jp/dbget-bin/www_bget?ana:alr1921), [alr3343](http://www.kegg.jp/dbget-bin/www_bget?ana:alr3343)

00785  [Lipoic acid metabolism](http://www.kegg.jp/kegg-bin/show_pathway?ana00785) (0/4)

00790  [Folate biosynthesis](http://www.kegg.jp/kegg-bin/show_pathway?ana00790) (1/19)

[alr1260](http://www.kegg.jp/dbget-bin/www_bget?ana:alr1260)

00670  [One carbon pool by folate](http://www.kegg.jp/kegg-bin/show_pathway?ana00670) (2/11)

[all0788](http://www.kegg.jp/dbget-bin/www_bget?ana:all0788), [alr0308](http://www.kegg.jp/dbget-bin/www_bget?ana:alr0308)

00860  [Porphyrin and chlorophyll metabolism](http://www.kegg.jp/kegg-bin/show_pathway?ana00860) (4/55)

[all0948](http://www.kegg.jp/dbget-bin/www_bget?ana:all0948), [all0949](http://www.kegg.jp/dbget-bin/www_bget?ana:all0949), [alr1042](http://www.kegg.jp/dbget-bin/www_bget?ana:alr1042), [alr3934](http://www.kegg.jp/dbget-bin/www_bget?ana:alr3934)

00130  [Ubiquinone and other terpenoid-quinone biosynthesis](http://www.kegg.jp/kegg-bin/show_pathway?ana00130) (0/19)

Metabolism of terpenoids and polyketides (3)

00900  [Terpenoid backbone biosynthesis](http://www.kegg.jp/kegg-bin/show_pathway?ana00900) (1/13)

[alr3230](http://www.kegg.jp/dbget-bin/www_bget?ana:alr3230)

00909  [Sesquiterpenoid and triterpenoid biosynthesis](http://www.kegg.jp/kegg-bin/show_pathway?ana00909) (0/3)

00906  [Carotenoid biosynthesis](http://www.kegg.jp/kegg-bin/show_pathway?ana00906) (1/11)

[alr3524](http://www.kegg.jp/dbget-bin/www_bget?ana:alr3524)

00903  [Limonene and pinene degradation](http://www.kegg.jp/kegg-bin/show_pathway?ana00903) (1/5)

[alr3672](http://www.kegg.jp/dbget-bin/www_bget?ana:alr3672)

00523  [Polyketide sugar unit biosynthesis](http://www.kegg.jp/kegg-bin/show_pathway?ana00523)(0/7)

Biosynthesis of other secondary metabolites (0)

00311  [Penicillin and cephalosporin biosynthesis](http://www.kegg.jp/kegg-bin/show_pathway?ana00311) (0/4)
00521  [Streptomycin biosynthesis](http://www.kegg.jp/kegg-bin/show_pathway?ana00521) (0/12)
00401  [Novobiocin biosynthesis](http://www.kegg.jp/kegg-bin/show_pathway?ana00401) (0/3)

Xenobiotics biodegradation and metabolism (1)

00627  [Aminobenzoate degradation](http://www.kegg.jp/kegg-bin/show_pathway?ana00627) (0/9)
00364  [Fluorobenzoate degradation](http://www.kegg.jp/kegg-bin/show_pathway?ana00364) (0/4)
00625  [Chloroalkane and chloroalkene degradation](http://www.kegg.jp/kegg-bin/show_pathway?ana00625) (1/9)

[alr3672](http://www.kegg.jp/dbget-bin/www_bget?ana:alr3672)

00361  [Chlorocyclohexane and chlorobenzene degradation](http://www.kegg.jp/kegg-bin/show_pathway?ana00361) (0/6)
00623  [Toluene degradation](http://www.kegg.jp/kegg-bin/show_pathway?ana00623) (0/4)
00633  [Nitrotoluene degradation](http://www.kegg.jp/kegg-bin/show_pathway?ana00633) (0/3)
00643  [Styrene degradation](http://www.kegg.jp/kegg-bin/show_pathway?ana00643) (0/2)
00363  [Bisphenol degradation](http://www.kegg.jp/kegg-bin/show_pathway?ana00363) (0/3)
00626  [Naphthalene degradation](http://www.kegg.jp/kegg-bin/show_pathway?ana00626) (0/2)
00624  [Polycyclic aromatic hydrocarbon degradation](http://www.kegg.jp/kegg-bin/show_pathway?ana00624) (0/4)

**Genetic Information Processing** (5)

Transcription (0)

03020  [RNA polymerase](http://www.kegg.jp/kegg-bin/show_pathway?ana03020) (0/5)

Translation (2)

03010  [Ribosome](http://www.kegg.jp/kegg-bin/show_pathway?ana03010) (2/68)

[all4187](http://www.kegg.jp/dbget-bin/www_bget?ana:all4187). [asl4186](http://www.kegg.jp/dbget-bin/www_bget?ana:asl4186)

00970  [Aminoacyl-tRNA biosynthesis](http://www.kegg.jp/kegg-bin/show_pathway?ana00970) (0/89)

Folding, sorting and degradation (1)

03060  [Protein export](http://www.kegg.jp/kegg-bin/show_pathway?ana03060) (0/16)
04122  [Sulfur relay system](http://www.kegg.jp/kegg-bin/show_pathway?ana04122) (0/12)
03018  [RNA degradation](http://www.kegg.jp/kegg-bin/show_pathway?ana03018) (1/18)

[all3538](http://www.kegg.jp/dbget-bin/www_bget?ana:all3538)

Replication and repair (2)

03030  [DNA replication](http://www.kegg.jp/kegg-bin/show_pathway?ana03030) (0/19)
03410  [Base excision repair](http://www.kegg.jp/kegg-bin/show_pathway?ana03410) (1/7)

[all5306](http://www.kegg.jp/dbget-bin/www_bget?ana:all5306)

03420  [Nucleotide excision repair](http://www.kegg.jp/kegg-bin/show_pathway?ana03420) (1/9)

[alr0165](http://www.kegg.jp/dbget-bin/www_bget?ana:alr0165)

03430  [Mismatch repair](http://www.kegg.jp/kegg-bin/show_pathway?ana03430) (1/24)

[alr0165](http://www.kegg.jp/dbget-bin/www_bget?ana:alr0165)

03440  [Homologous recombination](http://www.kegg.jp/kegg-bin/show_pathway?ana03440) (0/25)

**Environmental Information Processing** (7)

Membrane transport (4)

02010  [ABC transporters](http://www.kegg.jp/kegg-bin/show_pathway?ana02010) (4/137)
[all2622](http://www.kegg.jp/dbget-bin/www_bget?ana:all2622), [all2623](http://www.kegg.jp/dbget-bin/www_bget?ana:all2623), [alr1094](http://www.kegg.jp/dbget-bin/www_bget?ana:alr1094), [alr1382](http://www.kegg.jp/dbget-bin/www_bget?ana:alr1382)

03070  [Bacterial secretion system](http://www.kegg.jp/kegg-bin/show_pathway?ana03070) (0/12)

Signal transduction (3)
02020  [Two-component system](http://www.kegg.jp/kegg-bin/show_pathway?ana02020) (3/59)

[all0949](http://www.kegg.jp/dbget-bin/www_bget?ana:all0949), [alr1094](http://www.kegg.jp/dbget-bin/www_bget?ana:alr1094), [alr2009](http://www.kegg.jp/dbget-bin/www_bget?ana:alr2009)

**Cellular Processes (0)**

Cell motility (0)

02030  [Bacterial chemotaxis](http://www.kegg.jp/kegg-bin/show_pathway?ana02030) (0/3)

**Human Diseases (0)**

Drug resistance (0)

00312  [beta-Lactam resistance](http://www.kegg.jp/kegg-bin/show_pathway?ana00312) (0/7)
