# Supplementary material for: Investigation of a Quadruplex-Forming Repeat Sequence Highly Enriched in Xanthomonas and Nostoc sp
Source: PLoS One. 2015 Dec 22;10(12):e0144275. doi: 10.1371/journal.pone.0144275 (PMC4692102; doi:10.1371/journal.pone.0144275)
Supplement: S5 File — Sequence comparison between repeat containing regions in Xcc and Xac (Table A). Control sequences used for sequence comparison between Xcc and Xac (Table B). (DOCX) [file pone.0144275.s005.docx]

**Supporting Information 5:**

Contents:

S5 Table A: Sequence comparison between repeat containing regions in *Xcc* and *Xac*

S5 Table B: Control sequences used for sequence comparison between *Xcc* and *Xac*

S5 Table A: Sequence comparison between repeat containing regions in *Xcc* and *Xac*

Sequence comparison of repeat containing regions between *Xcc* and *Xac* are shown. Arrows represent the orientation of the neighboring genes relative to the repeat in Xcc. ITR is short for intergenic region.

| **#** | **inv**  **rep** | **orient genes** | **blastn: ORF-ITR-ORF Xac** | **Repeat present?** | **Change in flanking genes?** |
| --- | --- | --- | --- | --- | --- |
| 1 | 2 | ►R◄ | insertion | repeats are present, ITR in Xac is longer | no |
| 2 | 1 | ►R◄ | insertion | repeats are present, ITR in Xac is longer | no |
| 3 | 4 | ◄R◄ | no change, less homology in ITR | repeats present, less homology in ITR | no |
| 4 | 3 | ►R► | no change, less homology in ITR | repeats present, less homology in ITR | no |
| 5 | no | ►R► | no change | repeats present, no change | no |
| 6 | no | ►R◄ | flanking region missing | no repeat | lctD (Xcc) is homologous to lldD (Xac) but ITR and other gene not homologous |
| 7 | no | ►R► | flanking region missing | repeat present, ITR not homologous | flanking region not homologous |
| 8 | no | ◄R◄ | insertion | repeat present, but GGGAATG type, ITR longer in Xac | no |
| 9 | no | ►R◄ | gap | repeat present, but more mutations, ITR shorter in Xac | no |
| 10 | no | ◄R\| | no change, intragenic repeat | no change, intragenic repeat (probably no real repeat) | no |
| 11 | no | ◄R\| | no change, intragenic repeat | repeat present | no |
| 12 | no | ►R► | insertion | repeat present, but ITR is longer in Xac | no |
| 13 | no | ►R◄ | gap | repeat present, but gene ice gene is missing in Xac, | in Xcc: cdsA- ice - fis; in Xac: cdsA - fis |
| 14 | no | ◄R► | no homology in ITR | no repeat in Xac ITR, no significant similarity, ITR slightly shorter | no |
| 15 | no | \|R► | no change | repeat present, no change | no |
| 16 | 17 | ►R► | no change | repeat present, no change | no |
| 17 | 16 | ◄R◄ | no change | repeat present, no change | no |
| 18 | 19 | ►R► | no change | repeat present, no change | no |
| 19 | 18 | ◄R◄ | no change | repeat present, no change | no |
| 20 | 21 | ►R► | no change, less homology in ITR | repeat present, ITR a little shorter in Xac | no |
| 21 | 20 | ◄R◄ | no change, less homology in ITR | repeat present, ITR a little shorter in Xac | no |
| 22 | no | ◄R\| | no change | repeat mutated | no |
| 23 | no | ►R► | no alignment possible |  |  |
| 24 | 25 | ◄R◄ | repeat missing, insertion | repeat missing, instead another gene is inserted | yes, gene inserted |
| 25 | 24 | ►R► | repeat missing, insertion | repeat missing, instead another gene is inserted | yes, gene inserted |
| 26 | no | \|R► | no change, intragenic repeat | repeat present | no |
| 27 | 28 | \|R► | no change, intragenic repeat | repeat present | no |
| 28 | 27 | ◄R\| | no change, intragenic repeat | repeat present | no |
| 29 | no | ◄R◄ | no change, less homolog in ITR | repeat present | no |
| 30 | no | ►R◄ | insertion | repeat present, but ITR is longer in Xac | no |
| 31 | other | ◄R◄ | no change | repeat present, no change | no |
| 32 | other | ◄R◄ | no change | repeat present, no change | no |
| 33 | no | ◄R◄ | no change, less homology in ITR | repeat present but mutated, ITR same length | no |
| 34 | no | ◄R◄ | gap | repeat present but shorter, ITR in Xcc is longer | end of kdpC less homologous because of 2nd repeat in Xac |
| 35 | 36 | \|R► | no change, intragenic repeat | repeat present, no change | no |
| 36 | 35 | ◄R\| | no change, intragenic repeat | repeat present, no change | no |
| 37 | other | ◄R\| | no change, intragenic repeat | repeat present, no change | no |
| 38 | 39 | ►R► | no change | repeat present, no change | no |
| 39 | 38 | ◄R◄ | no change | repeat present, no change | no |
| 40 | other | \|R► | no change, intragenic repeat | repeat present, no change | no |
| 41 | other | ►R► | no change | repeat present, no change | no |
| 42 | no | ◄R◄ | no change | repeat present, no change | no |
| 43 | other | ◄R◄ | no change | repeat present, no change | no |
| 44 | no | ◄R► | gap | no repeat, ITR in Xac is shorter with less homology | no |
| 45 | no | ►R◄ | insertion | no repeat, ITR in Xac is longer, but less homology | no |
| 46 | no | ◄R◄ | no change | repeat present | no |
| 47 | other | ◄R◄ | no change | repeat present, but less homologous | no |
| 48 | no | ►R◄ | gap (minor) | repeat present, but shorter | no |
| 49 | 50 | ►R► | no change | repeat present, no change | no |
| 50 | 49 | ◄R◄ | no change | repeat present, no change | no |
| 51 | 52 | ►R► | no change | repeat present, no change | no |
| 52 | 51 | ◄R◄ | no change | repeat present, no change | no |
| 53 | no | ◄R◄ | gap | no repeat, G-rich ITR, ITR in Xac is shorter | no |
| 54 | no | ◄R\| | flanking region missing | repeat present, but intragenic (real repeat?) | next gene is different and differently oriented (hypothetical gene) |
| 55 | no | ►R◄ | gap | repeat present, ITR in Xac is shorter | no |
| 56 | 57 | ►R► | no change | repeat present, no change | no |
| 57 | 56 | ◄R◄ | no change | repeat present, no change | no |
| 58 | no | ◄R◄ | no gap, less homology in flanking gene | no repeat | C-terminal part of ribF is not homologous |
| 59 | no | ◄R► | no change, less homology in ITR | repeat present, longer repeat in Xac, but shorter ITR | no |
| 60 | no | ◄R◄ | no change | repeat mutated, ITR same length | no |
| 61 | 62 | ►R► | no change, less homology in ITR | repeats present | no |
| 62 | 61 | ◄R◄ | no change, less homology in ITR | repeats present | no |
| 63 | 64 | ►R► | no change | repeat present, no change | no |
| 64 | 63 | ◄R◄ | no change | repeat present, no change | no |
| 65 | 66 | ◄R◄ | insertion | only one repeat present, ITR in Xac is longer | no |
| 66 | 65 | ►R► | insertion | only one repeat present, ITR in Xac is longer | no |
| 67 | 68 | ◄R► | gap | repeats present, but distance between repeats longer in Xcc, ITR is shorter in Xac | hypothetical gene starts at different start codon |
| 68 | 67 | ◄R► | gap | repeats present, but distance between repeats longer in Xcc, ITR is shorter in Xac | hypothetical gene starts at different start codon |
| 69 | 70 | ◄R◄ | no change | repeats present | no |
| 70 | 69 | ►R► | no change | repeats present | no |
| 71 | 72 | ◄R◄ | no change | repeats present, intragenic repeats | no |
| 72 | 71 | ►R► | no change | repeats present, intragenic repeats | no |
| 73 | 74 | ◄R◄ | no change | repeats present | no |
| 74 | 73 | ►R► | no change | repeats present | no |
| 75 | 76 | ◄R◄ | no change | repeats present | no |
| 76 | 75 | ►R► | no change | repeats present | no |
| 77 | other | ►R◄ | gap | repeats present, ITR between repeats is shorter in Xac,  repeats overlap with stop codons in Xac but not in Xcc | no |
| 78 | other | ◄R◄ | gap / less homology in ITR | repeat present but other type, ITR in Xac is slightly longer | no |
| 79 | other | ►R► | no change | repeats present | no |
| 80 | 81 | ◄R◄ | no change | repeats present | no |
| 81 | 80 | ►R► | no change | repeats present | no |
| 82 | other | \|R► | no change, intragenic repeat | repeats present but mutated | no |
| 83 | no | ◄R◄ | gap | repeat present, but ITR is shorter in Xac | no |
| 84 | no | ►R◄ | no change, less homology in ITR | repeat mutated, less homology in ITR | no |
| 85 | no | \|R► | no change, intragenic repeat | repeat present, intragenic | no |
| 86 | other | ►R◄ | no change | repeats present, less homology in ITR | no |
| 87 | no | ◄R◄ | no change, less homology in ITR | no repeat, ITR same length but less homology | no |
| 88 | 89 | ◄R◄ | no change | repeat present | no |
| 89 | 88 | ►R► | no change | repeat present | no |
| 90 | no | ◄R◄ | flanking region missing | repeat present | hypothetical gene and repeat in different genomic region than ISxac2 |
| 91 | no | ◄R◄ | gap | repeats present, less homology in ITR, ITR in Xac shorter | no |
| 92 | 93 | ◄R◄ | no change | repeats present | no |
| 93 | 92 | ►R► | no change | repeats present | no |
| 94 | 94 | ►R◄ | Insertion | repeats present, but intragenic in Xcc, but intergenic in Xac and ITR longer | no (annotation wrong?) |
| 95 | 95 | ◄R► | insertion | repeats present, but intragenic in Xcc, but intergenic in Xac and ITR longer | no (annotation wrong?) |
| 96 | 97 | ►R◄ | no change, less homology in ITR | repeats present, less homology in ITR but similar distance | no |
| 97 | 96 | ►R◄ | no change, less homology in ITR | repeats present, less homology in ITR but similar distance | no |
| 98 | no | ◄R► | flanking region missing | repeat missing, in Xac also long ITR, but other neighboring gene | hypothetical gene is homologous, sensor is not |
| 99 | 100 | ◄R◄ | no change | repeat present, less homology in ITR | no |
| 100 | 99 | ►R► | no change | repeat present, less homology in ITR | no |
| 101 | 102 | ◄R◄ | no change, less homolog in ITR | repeat present, less homology in ITR | no |
| 102 | 101 | ►R► | no change, less homolog in ITR | repeat present, less homology in ITR | no |
| 103 | no | ►R► | no change | repeats present | no |
| 104 | 105 | ◄R► | no change | repeats present | no |
| 105 | 104 | ◄R► | no change | repeats present | no |
| 106 | no | ►R► | insertion | no repeat, ITR longer in Xac | no |
| 107 | 108 | ◄R◄ | no change | repeats present | no |
| 108 | 107 | ►R► | no change | repeats present | no |
| 109 | no | ►R► | gap | repeat present, but ITR longer in Xcc and in Xac second repeat | no |
| 110 | 111 | ◄R◄ | no change | repeats present | no |
| 111 | 110 | ►R► | no change | repeats present | no |
| 112 | no | ►R◄ | gap | repeat present but shorter and mutated, ITR shorter in Xac | no |
| 113 | no | ◄R◄ | no change, less homology in ITR | no repeat, ITR same length but less homology in part of ITR | no |
| 114 | gleich mit 115 | ►R◄ | no change | repeats present | no |
| 115 | gleich mit 114 | ►R◄ | no change | repeats present | no |
| 116 | 117 | ◄R◄ | no change | repeats present | no |
| 117 | 116 | ►R► | no change | repeats present | no |
| 118 | other | ◄R\| | less homology, intragenic repeat | repeats present, but less homology after first repeat in n-terminal part of nadD | n-terminal part of nadD less homologous (annotation wrong?) |
| 119 | no | ►R◄ | no change | repeats present | no |
| 120 | no | ◄R◄ | gap | repeat present, but mutated, ITR much shorter in Xac | no |
| 121 | 122 | ◄R◄ | no change | repeats present | no |
| 122 | 121 | ►R► | no change | repeats present | no |
| 123 | 124 | ►R► | gap | repeat present, ITR shorter in Xac | no |
| 124 | 123 | ◄R◄ | gap | repeat present, repeat longer in Xcc, ITR shorter in Xac | no |
| 125 | no | ►R► | no change, less homology in ITR | repeat present but mutated, ITR same length | no |
| 126 | no | ◄R\| | no change | short intragenic repeat, mutated (probably no repeat) | no |
| 127 | no | ◄R◄ | no change | intragenic repeat present but other type | no |
| 128 | 129 | ◄R◄ | no change | repeats present, but less homology in ITR | no |
| 129 | 128 | ►R► | no change | repeats present, but less homology in ITR | no |
| 130 | 131 | ◄R◄ | no change | repeats present | no |
| 131 | 130 | ►R► | no change | repeats present | no |
| 132 | no | \|R► | no change, intragenic repeat | no repeat, less homology around repeat | no |
| 133 | 134 | ►R◄ | insertion | repeats present, but insertion after repeats, ITR longer in Xac | no |
| 134 | 133 | ►R◄ | insertion | repeats present, but insertion after repeats, ITR longer in Xac | no |
| 135 | no | ◄R◄ | flanking region missing | repeat present | no homology for intS in Xac |
| 136 | 137 | ►R► | no change | repeat present, intragenic | no |
| 137 | 136 | ◄R\| | no change | repeat present, less homology in ITR | no |
| 138 | 139 | \|R► | no change, intragenic repeat | repeats present | no |
| 139 | 138 | ►R► | no change, intragenic repeat | repeats present | no |
| 140 | 141 | ►R► | no change | repeats present | no |
| 141 | 140 | ◄R◄ | no change | repeats present | no |
| 142 | no | ◄R◄ | no change | repeat mutated | no |
| 143 | 144 | ◄R◄ | no change, less homology in ITR | repeat present, less homology in ITR | no |
| 144 | 143 | ►R► | no change, less homology in ITR | repeat present, less homology in ITR | no |
| 145 | 146 | ◄R◄ | no change | repeats present, but other type | no |
| 146 | 145 | ►R► | no change | repeats present | no |
| 147 | other | ◄R◄ | no change | repeats mutated | no |
| 148 | other | ►R► | no change | repeat present | no |
| 149 | no | ◄R\| | flanking region missing | repeat present, short intragenic | no, no change in thiC, flanking region changes in following ITR |
| 150 | other | ►R► | no change | repeat present | no |
| 151 | 152 | ◄R► | no change | repeat present | no |
| 152 | 151 | ◄R► | no change | repeat present | no |
| 153 | 154 | ►R► | no change | repeat present | no |
| 154 | 153 | ◄R◄ | no change | repeat present | no |
| 155 | no | ►R◄ | gap | repeat not so long in Xac, less homology in ITR after repeat | no |
| 156 | other | ►R◄ | no change | repeat present | no |
| 157 | other | ◄R◄ | no change | repeat present | no |
| 158 | 159 | ◄R◄ | gap | repeat present, ITR shorter in Xac | no |
| 159 | 158 | ►R► | gap | repeat present, ITR shorter in Xac | no |
| 160 | 161 | ►R◄ | no change, less homology in ITR | repeat present, less homology in ITR | no |
| 161 | 160 | ►R◄ | no change, less homology in ITR | repeat present, less homology in ITR | no |
| 162 | other | ◄R◄ | insertion | repeat mutated, ITR in Xac longer | no |
| 163 | gleich mit 163 | ►R◄ | gap, less homology in ITR | repeat mutated, ITR in Xac is shorter | no |
| 164 | no | ►R◄ | gap | repeat + other type repeat in Xac, ITR in Xac is shorter | no |
| 165 | no | ►R► | gap | repeat present, ITR in Xac is shorter | no |
| 166 | no | ◄R► | gap, less homology in ITR | no repeat or mutated, ITR in Xac is shorter | no |
| 167 | no | ►R► | no change | repeat present, less homology | no |
| 168 | no | ◄R◄ | no change | repeat longer in Xac, less homology | no |
| 169 | 170 | ◄R◄ | no change | repeat present, less homology in ITR | no |
| 170 | 169 | ►R► | no change | repeat present, less homology in ITR | no |
| 171 | 172 | ►R◄ | no change | repeat present, less homology | no |
| 172 | 171 | ►R◄ | no change | repeat present, less homology | no |
| 173 | no | ◄R► | no change | repeat mutated | no |
| 174 | 175 | ◄R► | no change | repeat present | no |
| 175 | 174 | ◄R► | no change | repeat present | no |
| 176 | 177 | ►R► | no change | repeat present but slightly shorter, less homology | no |
| 177 | 176 | ◄R◄ | no change | repeat present | no |
| 178 | no | ◄R\| | no change | repeat present | no |
| 179 | 180 | ◄R◄ | no change | repeat present | no |
| 180 | 179 | ►R► | no change | repeat present | no |
| 181 | 182 | ►R► | insertion | repeat present, ITR in Xac is longer, less homology | no |
| 182 | 181 | ◄R◄ | insertion | repeat present, ITR in Xac is longer, less homology | no |
| 183 | no | ◄R◄ | insertion | repeat not present, insertion of hypothetical protein | gene inserted, xac4194 |
| 184 | no | ►R► | insertion | repeat not present, ITR in Xac is longer | no |
| 185 | no | ►R► | insertion | repeat present, +second or longer repeat, ITR in Xac is longer | no |
| 186 | no | ◄R◄ | gap | repeat present, ITR in Xac is shorter, less homology | no |

S5 Table B: Control sequences used for sequence comparison between *Xcc* and *Xac*

Sequence comparison of non-repeat containing regions between *Xcc* and *Xac* are shown. Arrows represent the orientation of the neighboring genes relative to the repeat in *Xcc*. ITR is short for intergenic region. The column controls indicates which alignment is used in which control set (1-5). Sequences were chosen by random generation of a numerical between 1 and 5076188 (total length of the *Xcc* genome), the next neighboring ITR region that did not contain a GGGAATC repeat was chosen for analysis.

| **controls** | | | | | **random generator** | **gene orientation** | **start  pos ITR** | **ORF-ITR-ORF** | **What happens in intergenic region (ITR)?** | **Change in flanking genes?** |
| --- | --- | --- | --- | --- | --- | --- | --- | --- | --- | --- |
| 1 | 2 |  |  |  | 82 | ►ITR► | 1371 | 1328 bp dnaA - 275 bp ITR - 1101 bp dnaN | no change | no |
| 1 | 2 | 3 | 4 |  | 17113 | ◄ITR► | 16696 | 1461 bp cls - 285 bp ITR - 1095 bp conserved | flanking region changes | yes, cls is homologous, but hypothetical gene is not, two more hypothetical genes in Xac and next homologous part is reversed in orientation |
|  |  | 3 |  |  | 22965 | ►ITR◄ | 23102 | 987 bp serA - 934 bp ITR - 1581 bp ctp | less homology in ITR, ITR in Xac is shorter | no |
| 1 | 2 |  | 4 | 5 | 23854 | ►ITR► | 25617 | 1581 bp ctp - 164 bp ITR - 1218 bp conserved | less homology in ITR, ITR in Xac is longer | no |
|  | 2 |  |  |  | 54335 | ◄ITR► | 54553 | 570 bp transcriptional - 260 bp ITR - 387 bp mecI | flanking region changing | yes, mecI present, transcriptional not homologous |
|  |  | 3 | 4 | 5 | 64595 | ►ITR► | 64810 | 1158 bp xylR - 138 bp ITR - 2157 bp avrBs2 | less homology in ITR, ITR in Xac is longer | no |
| 1 |  |  |  | 5 | 85581 | ►ITR◄ | 85388 | 627 bp conserved - 397 bp ITR - 189 bp conserved | less homology in ITR, different genes inserted | y/n, 189 bp conserved is homologous, region further downstream of 627 bp conserved is homologous again |
|  | 2 |  | 4 |  | 95404 | ►ITR► | 96184 | 990 bp esterase - 105 bp ITR - 399 bp atsE | flanking region changing | yes, atsE is present, esterase is not homologous |
|  |  | 3 |  | 5 | 201545 | ►ITR► | 201132 | 756 bp kduD - 159 bp ITR - 657 bp sugar | less homology in ITR, ITR is longer in Xac | no, hypothetical gene in Xac is homologous to sugar in Xcc |
| 1 |  |  | 4 | 5 | 208194 | ►ITR◄ | 208899 | 2241 bp fpvA - 392 bp ITR - 1038 bp conserved | less homology in ITR, same length | no |
| 1 | 2 |  |  |  | 247548 | ►ITR► | 247779 | 1431 bp ntrC - 1276 bp ITR - 480 bp yojM | not homologous, ITR in Xac is shorter | no |
|  |  | 3 | 4 |  | 262616 | ◄ITR► | 262859 | 597 bp conserved - 348 bp ITR - 1728 bp poxB | less homology in ITR, same length | no |
| 1 | 2 |  |  |  | 284916 | ◄ITR► | 284896 | 543 bp conserved - 94 bp ITR - 432 bp peptidyl | no change | no |
|  | 2 | 3 | 4 | 5 | 291002 | ►ITR► | 291840 | 2202 bp conserved - 369 bp ITR - 1053 bp conserved | flanking region changes within ITR | yes, 1053 bp conserved present, 2202 bp conserved missing |
|  |  | 3 |  |  | 322609 | ►ITR◄ | 322192 | 1326 bp nitrile - 166 bp ITR - 1236 bp conserved | not homologous | yes, nitrile homologous, except last 9nt, flanking region changes |
| 1 |  |  |  |  | 329537 | ►ITR► | 328418 | 429 bp ohr - 56 bp ITR - 276 bp lipase | flanking region changes within ITR | yes, ohr homologous, but lipase not |
|  | 2 |  |  |  | 330780 | ►ITR► | 329470 | 618 bp lipase - 503 bp ITR - 501 bp conserved | part of intergenic region is homologous | yes, lipase not homologous, conserved gene present |
|  |  | 3 |  | 5 | 379465 | ►ITR► | 379622 | 393 bp conserved - 200 bp ITR - 870 bp purU | less homology in ITR, ITR in Xac is longer | no |
| 1 |  |  |  |  | 387184 | ►ITR► | 387308 | 906 bp metR - 109 bp ITR - 582 bp sflA | less homology in ITR, ITR in Xac is longer | no |
| 1 | 2 |  |  |  | 452570 | ►ITR► | 452239 | 1905 bp gidA - 552 bp ITR - 471 bp conserved | no change | no |
|  |  | 3 |  | 5 | 464556 | ◄ITR► | 464920 | 906 bp ubiA - 500 bp ITR - 447 bp int | less homology in ITR, ITR in Xac is shorter | y/n, ubiA is homologous, int is partly homologous to hypothetical protein in Xac |
| 1 | 2 | 3 |  |  | 471240 | ◄ITR► | 470818 | 705 bp sodM - 54 bp ITR - 381 bp conserved | no homology | no homology |
|  | 2 |  |  | 5 | 495784 | ►ITR► | 493655 | 1425 bp glgA - 41 bp ITR - 2190 bp glgB2 | no change | no |
|  |  | 3 |  |  | 504071 | ►ITR► | 502373 | 2619 bp glgY/189 bp conserved (overlapping) - 42 bp ITR - 2163 bp glgX | less homology in ITR, conserved gene present | no |
| 1 |  |  |  | 5 | 504071 | ►ITR◄ | 505227 | 681 bp conserved - 852 bp ITR - 759 bp 3 | less homology in ITR, ITR in Xac is shorter | no |
|  | 2 | 3 |  |  | 509190 | ►ITR◄ | 509331 | 432 bp virK - 194 bp ITR - 1473 bp oprN | less homology in ITR, ITR in Xac is longer | y/n, virK is homologous, oprN is homologous except for part adjacent to ITR |
|  |  | 3 |  | 5 | 532484 | ►ITR► | 532666 | 1521 bp yhiP- 11 bp ITR - 591 bp conserved | no change | no |
| 1 |  |  | 4 |  | 538342 | ►ITR► | 536154 | 393 bp conserved - 36 bp ITR - 1368 bp hmgA | no change | no |
| 1 | 2 |  |  |  | 559297 | ►ITR► | 560521 | 1074 bp threonine - 13 bp ITR - 366 bp hypothetical | flanking region changes | yes, threonine is present, but hypothetical gene is not |
|  |  | 3 |  | 5 | 564409 | ►ITR► | 560900 | 366 bp hypothetical - 124 bp ITR - 2985 bp hsdR | no homology | no homology |
| 1 | 2 |  |  |  | 567931 | ►ITR► | 569015 | 1617 bp type -145 bp ITR - 579 bp trpG | flanking region changes | yes, trpG is homologous, type is not |
|  | 2 |  |  | 5 | 595514 | ►ITR► | 597722 | 2244 bp conserved - 87 bp ITR - 1296 bp purD | less homology in ITR, ITR in Xac is longer | no |
|  |  | 3 | 4 | 5 | 618733 | ►ITR◄ | 617852 | 534 bp conserved - 1 bp ITR - 921 bp prmA | flanking region changes with end of prmA | yes, conserved protein is not homologous |
| 1 |  |  | 4 | 5 | 654107 | ►ITR◄ | 653996 | 606 bp conserved - 154 bp ITR - 678 bp ompW | flanking region changes | yes, ompW is homologous, conserved is not |
|  | 2 |  | 4 | 5 | 658573 | ►ITR► | 658976 | 648 bp conserved - 16 bp ITR - 1848 bp lpdA | no change | no |
|  |  | 3 | 4 |  | 673634 | ◄ITR► | 673209 | 414 bp conserved - 118 bp ITR - 1365 bp glmU | less homology in ITR, ITR in Xac is longer | no |
| 1 |  | 3 | 4 |  | 686283 | ◄ITR► | 685292 | 1272 bp ybjY - 203 bp ITR - 1830 bp glmS | no change | no |
| 1 | 2 |  | 4 |  | 693691 | ►ITR► | 692317 | 927 bp conserved - 1051 bp ITR - 759 bp conserved | no change | no |
|  |  | 3 | 4 | 5 | 724255 | ►ITR► | 723969 | 1287 bp wzt - 40 bp ITR - 2295 bp wxcB | no homology | no |
| 1 | 2 |  | 4 |  | 757318 | ►ITR► | 758111 | 927 bp conserved - 2 bp ITR - 831 bp yrbF | no change | no |
|  | 2 |  | 4 | 5 | 761868 | ►ITR◄ | 761564 | 1260 bp conserved - 62 bp ITR - 468 bp DNA | no change | no |
|  |  | 3 | 4 |  | 780316 | ►ITR◄ | 781241 | overlap with conserved gene/555 bp hypothetical - 207 bp ITR - 468bp conserved | less homology in ITR, different genes inserted | y/n, in xcc und xac different hypothetical genes inserted between homologous conserved genes |
| 1 |  |  | 4 | 5 | 786281 | ►ITR► | 784021 | 795 bp dsbC - 331 bp ITR - 4046 bp purL | less homology in ITR, ITR in Xac is longer and  contains repeat#158 | no |
|  | 2 |  | 4 |  | 792330 | ►ITR► | 796954 | 7056 bp xadA - 158 bp ITR - 1881 bp protease | less homoloty in ITR, ITR in Xac is shorter | y/n xadA of different length, parts without homology, protease present |
|  |  | 3 | 4 |  | 820495 | ►ITR► | 819036 | 909 bp conserved - 577 bp ITR - 1344 bp conserved | no change | no |
| 1 |  | 3 |  |  | 824176 | ►ITR► | 824972 | 1197 bp pncB - 51 bp ITR - 828 bp cellulose | several genes inserted between homologous genes | no, several genes inserted in ITR, cellulose is homologous to bcsC in Xac |
| 1 | 2 |  | 4 | 5 | 848119 | ►ITR► | 848050 | 2049 bp kdpB -30 bp ITR - 630 bp kdpC | no change | no |
|  |  | 3 | 4 | 5 | 858445 | ◄ITR► | 856884 | 1731 bp conserved - 82 bp ITR - 822 bp conserved | no change | no |
| 1 | 2 |  | 4 |  | 871057 | ►ITR► | 876767 | 1230 bp ftsZ - 226 bp ITR - 912 bp lpxC | less homology in ITR, same length | no |
|  |  |  | 4 |  | 882540 | ►ITR► | 882382 | 2739 bp secA - 469 bp ITR - 369 bp conserved | flanking region changes within ITR | yes, secA homologous, conserved gene missing |
|  |  | 3 | 4 | 5 | 910827 | ►ITR► | 909614 | 2064 bp conserved - 101 bp ITR - 1296 bp conserved | less homology in ITR | y/n, conserved (2064) is homologous, conserved (1296) is longer in Xcc, C-terminal part is homologous |
| 1 |  |  |  |  | 958397 | ►ITR► | 959720 | 1182 bp visC - 102 bp ITR - 717 bp hydrolase | less homology in ITR | no, but genes less homologous close to ITR |
|  | 2 | 3 |  | 5 | 969783 | ►ITR► | 970064 | 828 bp fadB - 33 bp ITR - 471 bp transcriptional | no change | no |
|  |  | 3 | 4 |  | 977844 | ►ITR► | 977993 | 930 bp integral - 125 bp ITR - 744 bp transcriptional | flanking region changing | yes, integral is present (hypothetical in Xac), transcriptional not homologous |
| 1 |  |  |  | 5 | 987544 | ►ITR► | 987434 | 570 bp conserved - 276 bp ITR - 969 bp talB | less homology in ITR, ITR is longer in Xac | no |
| 1 | 2 |  |  |  | 990586 | ►ITR► | 990343 | 942 bp oxyR - 90 bp ITR - 1590 bp ahpF | no change | no |
|  |  | 3 |  |  | 1020000 | ◄ITR► | 1019495 | 597 bp tryptophan - 112 bp ITR - 1277 bp rbn | no change | no |
| 1 | 2 |  | 4 | 5 | 1031610 | ►ITR► | 1032338 | 888 bp ipk - 264 bp ITR - 960 bp prsA | less homology in ITR, same length | no |
|  | 2 |  |  |  | 1044510 | ►ITR► | 1046235 | 4164 bp rpoB - 100 bp ITR - 4218 bp rpoC | less homology in ITR, ITR is longer in Xac | no |
|  |  | 3 |  |  | 1052872 | ►ITR► | 1053886 | 2118 bp fusA - 48 bp ITR - 1191 bp tufB | no change | no |
| 1 |  |  |  | 5 | 1065799 | ►ITR► | 1066459 | 1368 bp secY- 332 bp ITR - 357 bp rpsM | no change | no |
|  | 2 |  |  |  | 1072019 | ►ITR► | 1072346 | 1437 bp dhs1 - 226 bp ITR - 1128 bp membrane | gap, ITR shorter in ITR | no |
|  |  | 3 |  | 5 | 1096569 | ►ITR► | 1096631 | 1032 bp cysA - 293 bp ITR - 717 bp conserved | less homology in ITR, same length | no |
| 1 |  |  | 4 |  | 1124755 | ◄ITR► | 1124718 | 1647 bp conserved - 27 bp ITR - 429 bp grxC | no change | no |
| 1 | 2 | 3 | 4 |  | 1138888 | ►ITR► | 1137789 | 971 bp ppiD - 540 bp ITR - 486 bp hypothetical | flanking region changing | yes, ppiD present but hypothetical gene no |
|  |  | 3 | 4 |  | 1145826 | ◄ITR► | 1145920 | 1046 bp opsX - 20 bp ITR - 750 bp conserved | no change | no |
| 1 | 2 |  | 4 | 5 | 1157555 | ►ITR► | 1157662 | 957 bp conserved - 4 bp ITR - 954 bp moxR | no change | no |
|  | 2 |  |  |  | 1183850 | ►ITR► | 1183888 | 2596 bp acnA - 35 bp ITR - 294 bp conserved | no change | no |
|  |  | 3 | 4 |  | 1185429 | ►ITR► | 1185824 | 1200 bp conserved - 254 bp ITR - 2217 bp fyuA | less homology in ITR, ITR is shorter in Xac | no, N-terminal part of conserved is not homologous |
| 1 |  | 3 | 4 | 5 | 1217669 | ►ITR◄ | 1217146 | 855 bp hypothetical - 197 bp ITR - 1197 bp hypothetical | no homology | no homology |
|  | 2 |  | 4 | 5 | 1231241 | ►ITR► | 1230585 | 3123 bp type -1 bp ITR - 2919 bp possible | no homology | no homology |
|  |  | 3 | 4 |  | 1283139 | ►ITR► | 1284786 | 1970 bp oliA - 363 bp ITR - 1022 bp conserved | less homology in ITR, ITR is longer in Xac | y/n, genes are homologous, except for n-terminal part of hypothetical protein |
| 1 |  |  | 4 |  | 1325053 | ►ITR◄ | 1324695 | 1203 bp purT - 265 bp ITR - 1056 bp conserved | flanking region changing | yes, purT is homologous, conserved gene is not homologous |
| 1 | 2 |  | 4 | 5 | 1359297 | ►ITR► | 1360661 | 1431 bp hrpXct - 300 bp ITR - 1977 bp hsp90xc | less homology in ITR, ITR in Xac is shorter | no |
|  |  | 3 |  | 5 | 1445236 | ►ITR◄ | 1446036 | 1817 bp hrcC - 78 bp ITR - 366 bp hpa1 | less homology in ITR, ITR in Xac is longer  (hpa1 is present but very little homology) | y/n, hrcC is homologous, but hpa1 has very little homology in Xac |
| 1 | 2 |  |  |  | 1451016 | ►ITR► | 1450655 | 429 bp conserved - 309 bp ITR - 999 bp conserved | no homology in ITR | yes, part of 429 bp conserved is homologous to two copies of IS1389 ITR inbetween, ITR and 999 bp conserved is not homologous |
|  | 2 | 3 |  |  | 1452679 | ►ITR► | 1454722 | 2241 bp conserved - 84 bp ITR - bp IS1479 | no homology | no homology |
|  |  | 3 | 4 | 5 | 1467392 | ►ITR► | 1469697 | 1208 bp galA - 167 bp ITR - 2684 bp btuB | no change | no |
| 1 |  |  | 4 |  | 1486659 | ►ITR► | 1485474 | 861 bp regulatory - 109 bp ITR - 1577 bp mucD | no change | no, only part of regulatory protein is less homologous |
|  | 2 |  | 4 |  | 1587008 | ◄ITR► | 1587615 | 1014 bp lpxD - 61 bp ITR - 267 bp conserved | no change | no |
|  |  | 3 | 4 |  | 1589559 | ►ITR◄ | 1587943 | 267 bp conserved - 82 bp ITR - 2457 bp oma | less homology in ITR, same length | no |
| 1 |  |  | 4 |  | 1593927 | ►ITR► | 1593042 | 1190 bp dxr - 2 bp ITR - 828 bp cdsA | no change | no |
| 1 | 2 |  | 4 |  | 1614712 | ►ITR◄ | 1616095 | 1695 bp asnB - 203 bp ITR - 1161 bp conserved | less homology in ITR, ITR in Xac is shorter | no |
|  |  | 3 | 4 | 5 | 1620158 | ►ITR► | 1619827 | 2141 bp fhuA - 426 bp ITR - 2486 bp acyII | less homology in ITR | no |
| 1 | 2 |  |  | 5 | 1629925 | ►ITR► | 1630015 | 1515 bp yjcP - 10 bp ITR - 1194 bp pmrA | no change | no |
|  | 2 |  |  |  | 1643392 | ►ITR► | 1643689 | 843 bp conserved - 64 bp ITR - 501 bp transcriptional | no change | no, but far end of hypothetical protein is not homologous |
|  |  | 3 | 4 | 5 | 1647656 | ►ITR◄ | 1647339 | 486 bp gpo - 158 bp ITR - 780 bp fpr | less homology in ITR, same length | no |
| 1 |  |  |  |  | 1648827 | ◄ITR► | 1650426 | 1926 bp msbA - 148 bp ITR - 1554 bp fumB | less homology in ITR, ITR in Xac is longer | no |
|  | 2 | 3 |  | 5 | 1653553 | ►ITR► | 1653411 | 636 bp gst - 139 bp ITR - 684 bp cynT | less homology in ITR, ITR in Xac is shorter | no |
|  |  | 3 |  |  | 1660182 | ►ITR► | 1660588 | 777 bp conserved - 176 bp ITR - 1164 bp gcdH | no change | no |
| 1 |  |  |  | 5 | 1677863 | ►ITR► | 1679656 | 3170 bp mexF - 33 bp ITR - 732 bp short | no change | no |
| 1 | 2 |  | 4 | 5 | 1696786 | ►ITR► | 1696137 | 3198 bp helicase - 383 bp ITR - 1426 bp IS1478 | flanking region changes | yes, IS1478 present, helicase not homologous |
|  |  | 3 | 4 |  | 1725352 | ►ITR► | 1723987 | 1131 bp dnaJ - 374 bp ITR - 909 bp pdxY | less homology in ITR, ITR in Xac is longer | no |
| 1 | 2 | 3 | 4 | 5 | 1753240 | ◄ITR► | 1754452 | 363 bp transcriptional - 385 bp ITR - 558 bp btuE | no change | no |
|  | 2 |  | 4 | 5 | 1754550 | ►ITR► | 1756449 | 894 bp fkpA - 24 bp ITR - 1346 bp ugd | no change | no |
|  |  | 3 |  | 5 | 1763808 | ►ITR◄ | 1764698 | 1206 bp conserved - 415 bp ITR - 2756 bp metH2 | less homology in ITR, ITR in Xac is shorter | no, but part of metH is less homologous |
| 1 |  |  |  |  | 1767157 | ►ITR► | 1767870 | 2756 bp metH2 - 82 bp ITR - 1139 bp metH1 | less homology in ITR, ITR in Xac is longer | no |
|  | 2 |  |  |  | 1770494 | ◄ITR► | 1770141 | 1053 bp transcriptional - 66 bp ITR - 1149 bp acdA | no change | no |
|  |  | 3 |  | 5 | 1775492 | ►ITR► | 1777341 | 2423 bp vacB - 126 bp ITR - 474 bp conserved | less homology in ITR, ITR in Xac is longer | no |
| 1 |  |  |  |  | 1781668 | ►ITR◄ | 1782287 | 3153 bp conserved - 178 bp ITR - 651 bp rnt | less homology in ITR, repeats in ITR in Xac  but mutated in Xcc, same length | no |
| 1 | 2 | 3 |  |  | 1796329 | ◄ITR► | 1796227 | 783 bp crt - 164 bp - 699 bp peptidyl | no change | no |
|  |  | 3 | 4 |  | 1826884 | ►ITR► | 1826584 | 1182 bp conserved - 5 bp ITR - 3505 bp smc | flanking region changes within beginning of smc | yes, N-terminal part of smc is not homologous, conserved protein is not present |
| 1 | 2 |  | 4 | 5 | 1844213 | ►ITR► | 1844401 | 849 bp conserved - 63 bp ITR - 1682 bp hutU | less homology in ITR, | no, but conserved gene much longer in Xcc, in Xac ITR is longer |
|  | 2 |  |  |  | 1858170 | ►ITR► | 1858301 | 1085 bp serC - 48 bp ITR - 1220 bp pheA | less homology in ITR, ITR in Xac shorter | no, but n-terminal part of pheA is not homologous |
|  |  | 3 |  | 5 | 1861753 | ►ITR► | 1861582 | 597 bp TonB - 80 bp ITR - 750 bp conserved | less homology in ITR, ITR in Xac is longer | no, but less homology in TonB |
| 1 |  |  |  | 5 | 1867961 | ►ITR► | 1866815 | 1467 bp phage - 86 bp ITR - 1287 bp mbtG | no homology | y/n, parts of phage homologous to integrase |
|  | 2 |  |  |  | 1881980 | ►ITR► | 1888183 | 480 bp RadC - 15 bp ITR - 729 bp conserved | flanking region changes | y/n, radC is homologous, conserved gene is less homologous and further away |
|  |  | 3 |  |  | 1896670 | ►ITR► | 1896829 | 624 bp conserved - 934 bp ITR - 267 bp IS1477 | not homologous | yes, IS1477 not homologous, conserved gene present |
| 1 |  |  |  | 5 | 1932874 | ►ITR◄ | 1932019 | 2103 bp cvgSY - 37 bp ITR - 729 bp conserved | less homology in ITR, ITR in Xac is longer | no |
| 1 | 2 |  |  | 5 | 1936253 | ►ITR► | 1935767 | 1997 bp cycK - 66 bp ITR - 471 bp cycJ | no change | no |
|  |  | 3 |  |  | 1943809 | ◄ITR► | 1943266 | 534 bp cytochrome - 390 bp ITR - 423 bp conserved | no change | no |
| 1 | 2 |  |  |  | 1945202 | ►ITR► | 1944079 | 423 bp conserved - 145 bp ITR - 1143 bp aminotransferase | less homology in ITR, ITR in Xac is longer,  two genes inserted | yes, two more hypothetical genes inserted in ITR |
|  | 2 | 3 |  | 5 | 1947384 | ►ITR► | 1949582 | 906 bp conserved - 161 bp ITR - 846 bp methyltransferase | less homology in ITR, ITR in Xac is longer | no |
|  |  | 3 | 4 |  | 1995470 | ►ITR► | 1997096 | 2663 bp TonB - 424 bp ITR - 1676 bp aarF | flanking region changes | yes, aarF present, TonB not homologous |
| 1 |  |  | 4 |  | 2005838 | ►ITR◄ | 2007996 | 1965 bp mcpA - 690 bp ITR - 702 bp conserved | not homologous, ITR in Xac is shorter | no |
|  | 2 |  |  |  | 2018738 | ►ITR◄ | 2018815 | 519 bp conserved - 76 bp ITR - 324 bp ferredoxin | less homology in ITR, ITR in Xac is longer | no |
|  |  | 3 |  | 5 | 2056230 | ►ITR► | 2055941 | 855 bp panC - 107 bp ITR - 381 bp panD | less homology in ITR, ITR in Xac is longer | no |
| 1 |  | 3 |  | 5 | 2056794 | ►ITR► | 2057940 | 1515 bp pgi - 136 bp ITR - 282 bp hypothetical | insertion of another hypothetical gene | y/n, additional gene in ITR |
| 1 | 2 |  |  |  | 2080747 | ►ITR► | 2079569 | 285 bp conserved - 329 bp - 1532 bp aldehyde | less homology in ITR, ITR is longer in Xac | no |
|  |  | 3 |  |  | 2092617 | ►ITR► | 2084226 | 1701 bp fhaC - 69 bp ITR - 8756 bp fhaB | no change | no, middle part of fhaB is not homologous |
| 1 | 2 |  |  |  | 2103622 | ◄ITR► | 2103554 | 423 bp - 53 bp ITR - 1308 bp thrC | less homology in ITR, ITR in Xac is longer | no |
|  | 2 |  |  | 5 | 2106942 | ◄ITR► | 2106608 | 762 bp fnr - 108 bp ITR - 1434 bp hisS | no change | no |
|  |  | 3 | 4 | 5 | 2114663 | ►ITR► | 2108712 | 327 bp conserved - 9 bp ITR - 915 bp hisG | no change | no |
| 1 |  |  | 4 |  | 2126235 | ◄ITR► | 2125728 | 1481 bp yhdG - 164 bp ITR - 552 bp conserved | no change | no |
|  | 2 |  | 4 |  | 2129931 | ►ITR► | 2129911 | 1418 bp oxidoreductase - 34 bp ITR - 294 bp conserved | no change | no |
|  |  | 3 | 4 | 5 | 2183422 | ►ITR► | 2181900 | 486 bp cheW - 111 bp ITR - 798 bp conserved | no homology | no homology |
| 1 |  |  | 4 | 5 | 2191206 | ►ITR► | 2191971 | 2019 bp tsr - 29 bp ITR - 516 bp hypothetical | no change | no |
| 1 | 2 | 3 | 4 |  | 2276471 | ►ITR► | 2278496 | 1874 bp flgK - 11 bp ITR - 1085 bp flgJ | no change | no |
|  |  | 3 | 4 | 5 | 2393711 | ►ITR◄ | 2392227 | 594 bp W78 - 216 bp ITR - 1965 bp conserved | no homology | only middle part of conserved gene homologous |
| 1 | 2 |  |  |  | 2408611 | ◄ITR► | 2409037 | 1290 bp transport - 161 bp ITR - 417 bp conserved | no homology | no homology |
|  | 2 |  |  | 5 | 2410926 | ►ITR► | 2411287 | 1176 bp conserved - 177 bp ITR - 1143 bp conserved | no homology | no homology |
|  |  | 3 |  |  | 2435355 | ◄ITR► | 2436150 | 555 bp phage - 219 bp ITR - 1041 bp gII | no homology | no homology |
| 1 |  |  |  |  | 2458612 | ►ITR◄ | 2458206 | 3563 bp rne - 567 bp ITR - 647 bp gacA | less homology in ITR and c-term part of rne | no, but c-terminal part of rne is not homologous in xcc/xac |
|  | 2 |  |  |  | 2498702 | ►ITR► | 2496640 | 1937 bp intS - 633 bp ITR - 633 bp pgsA | flanking region changes | yes, pgsA is homologous and half of ITR, intS is not |
|  |  | 3 |  |  | 2523194 | ►ITR► | 2523334 | 720 bp pgl - 145 bp ITR - 1917 bp edd | less homology in ITR, ITR in Xac is shorter | no |
| 1 |  |  | 4 | 5 | 2641834 | ►ITR► | 2641083 | 1296 bp argH - 75 bp ITR - 951 bp argC | no change | no |
| 1 | 2 | 3 | 4 |  | 2644449 | ►ITR► | 2644066 | 1344 bp argB - 48 bp ITR - 1101 bp argE | no change | no |
|  |  | 3 | 4 | 5 | 2701453 | ►ITR► | 2699804 | 900 bp conserved - 38 bp ITR - 1875 bp mutL | no change | no |
| 1 | 2 |  | 4 |  | 2703242 | ►ITR► | 2703560 | 1692 bp amiC - 52 bp ITR - 501 bp conserved | no change | no, middle part of amiC is not homologous |
|  | 2 |  |  |  | 2716809 | ◄ITR► | 2715439 | 177 bp conserved - 221 bp ITR - 405 bp xrvA | no change | no |
|  |  | 3 |  | 5 | 2748504 | ►ITR► | 2748806 | 867 bp potH - 189 bp ITR - 1151bp potG | less homology in ITR, no ITR in Xac,  genes overlap | no, genes overlap |
| 1 |  |  |  |  | 2758192 | ►ITR► | 2759378 | 386 bp glnA - 6 bp ITR - 756 bp guaA | no change | no |
|  | 2 | 3 |  | 5 | 2774419 | ►ITR► | 2773990 | 1353 bp omega - 10 bp ITR - 1094 bp conserved | less homology in ITR, ITR in Xac is longer | no |
|  |  | 3 |  |  | 2792036 | ►ITR► | 2792125 | 660 bp transcriptional - 14 bp ITR - 1310 bp acrA | no change | no |
| 1 |  |  | 4 | 5 | 2820696 | ◄ITR► | 2821774 | 2519 bp cirA - 193 bp ITR - 633 bp upp | no change | no |
| 1 | 2 |  | 4 | 5 | 2838993 | ►ITR► | 2839500 | 1487 bp avrXccA2 - 474 bp ITR - 2342 bp peptidase | less homology in ITR, avrXccA2 is inserted  in Xcc but not Xac | yes, peptidase is homologous, but avrXcc is inserted in ITR in Xcc, but not present in Xac, next gene btuB is homologous again |
|  |  | 3 | 4 | 5 | 2857744 | ►ITR► | 2858312 | 1631 bp conserved - 127 bp ITR - 2210 bp peptidase | no change | no |
| 1 | 2 |  | 4 |  | 2881594 | ◄ITR► | 2881509 | 333 bp qacE - 235 bp ITR - 672 bp conserved | no homology | no homology |
|  | 2 |  | 4 |  | 2901670 | ►ITR► | 2901829 | 1317 bp fabH - 50 bp ITR - 1029 bp gumN | less homology in ITR, ITR in Xac is longer | no |
|  |  | 3 |  | 5 | 2908194 | ►ITR► | 2906015 | 888 bp gumK - 379 bp ITR - 1499 bp gumJ | no change | no |
| 1 |  |  |  |  | 2930763 | ◄ITR► | 2933008 | 2322 bp conserved - 113 bp ITR - 1079 bp aglA | no change | no |
|  | 2 |  |  |  | 2967124 | ►ITR► | 2967701 | 1346 bp conserved - 144 bp ITR - 3257 bp oar | no change | no, but intragenic part of oar is not homologous |
|  |  | 3 |  |  | 2985170 | ►ITR► | 2985751 | 1511 bp infB - 53 bp ITR - 2750 bp nusA | no change | no |
| 1 |  |  |  | 5 | 3030838 | ►ITR◄ | 3031194 | 990 bp hypothetical - 90 bp ITR - 1416 bp dniR | flanking region changes with end of dniR | yes, dniR present, hypothetical gene is not homologous |
| 1 | 2 | 3 | 4 | 5 | 3054894 | ►ITR► | 3054367 | 3048 bp oar - 339 bp ITR - 2145 bp metallopeptidase | less homology in ITR, gene inserted in ITR  in Xac | y/n, further gene inserted in ITR in Xac |
|  |  | 3 | 4 |  | 3058441 | ►ITR◄ | 3061517 | 2012 bp metallopeptidase - 202 bp ITR - 687 bp integral | less homology in ITR, ITR in Xac is longer | no |
| 1 | 2 |  | 4 |  | 3090724 | ◄ITR► | 3091058 | 1445 bp tldD - 98 bp ITR - 756 bp conserved | no change | no |
|  | 2 |  | 4 | 5 | 3107132 | ►ITR► | 3108871 | 2514 bp leuS - 9 bp ITR - 627 bp conserved | no change | no |
|  |  | 3 | 4 | 5 | 3119824 | ►ITR◄ | 3120218 | 1802 bp conserved - 668 bp ITR - 1832 bp bapA | less homology in ITR, ITR in Xac is shorter | no |
| 1 |  |  |  |  | 3127065 | ◄ITR► | 3126736 | 501 bp menG - 194 bp ITR - 828 bp conserved | less homology in ITR, ITR in Xac is longer | no |
|  | 2 |  |  | 5 | 3147823 | ◄ITR► | 3147328 | 417 bp conserved - 161 bp ITR - 2067 bp phuR | no change | no |
|  |  | 3 |  |  | 3179000 | ►ITR► | 3180318 | 3134 bp mexB - 15 bp ITR - 1199 bp mexA | no change | no |
| 1 |  |  |  | 5 | 3186613 | ►ITR► | 3187198 | 885 bp nthA - 59 bp ITR - 597 bp conserved | less homology in ITR, ITR in Xac is longer | no |
| 1 | 2 |  |  | 5 | 3198751 | ►ITR► | 3199935 | 3467 bp mfd - 751 bp ITR - 330 bp conserved | less homology in ITR, ITR in Xac is longer | no |
|  |  | 3 |  |  | 3236366 | ►ITR► | 3236418 | 669 bp yagT - 333 bp ITR - 1007 bp alcohol | less homology in ITR, ITR in Xac is longer | no |
| 1 | 2 |  | 4 |  | 3239684 | ◄ITR► | 3237759 | 1007 bp alcohol - 776 bp ITR - 1019 bp response | less homology in ITR, ITR in Xac is shorter | no, but end of alcohol is less homologous |
|  | 2 | 3 | 4 | 5 | 3274947 | ►ITR► | 3273531 | 543 bp pfpI - 107 bp ITR - 420 bp conserved | no change | no |
|  |  | 3 | 4 |  | 3274682 | ►ITR► | 3274738 | 459 bp conserved - 15 bp ITR - 1458 bp ynhE | no change | no |
| 1 |  |  |  | 5 | 3329264 | ◄ITR► | 3327505 | 777 bp ahyR - 189 bp ITR - 1380 bp amino | no change | no |
|  | 2 |  | 4 |  | 3346141 | ►ITR► | 3347780 | 1167 bp synthetase - 1 bp ITR - 576 bp conserved | no change | no |
|  |  | 3 |  |  | 3352597 | ◄ITR► | 3352581 | 600 bp pdxH - 194 bp ITR - 543 bp aroK | no change | no |
| 1 |  |  |  | 5 | 3362052 | ►ITR► | 3363026 | 3585 bp histidine - 483 bp ITR - 3543 bp histidine | another histidine gene inserted in ITR  in Xac | y/n, both genes present, but second is shorter in Xac |
| 1 | 2 |  |  |  | 3369893 | ◄ITR► | 3370694 | 3561 bp histidine - 96 bp ITR - 678 bp conserved | no change | y/n both genes present, but part of histidine after start codon in not homologous in Xac |
|  |  | 3 |  |  | 3398331 | ◄ITR► | 3399108 | 1364 bp conserved - 339 bp ITR - 1379 bp bla | no change | no |
| 1 | 2 |  |  |  | 3401569 | ►ITR◄ | 3402816 | 603 bp conserved / histone - 1026 bp ITR - 396 bp gcvH | less homology in ITR, ITR in Xac is shorter | yes, gcvH is homologous, n-terminal part (near ITR) of hypothetical protein is less homologous |
|  | 2 |  |  |  | 3404497 | ►ITR► | 3404239 | 396bp gcvH - 139 bp ITR - 1109 bp gcvT | less homology in ITR, same length | no |
|  |  | 3 |  | 5 | 3417167 | ►ITR► | 3417108 | 2742 bp iroN - 202 bp ITR - 1686 bp fucA1 | less homology, same length | no, less homology in middle part of iroN |
| 1 |  |  |  | 5 | 3423346 | ►ITR► | 3425382 | 2682 bp beta - 261 bp ITR - 2667 bp glucan | less homology in ITR | no, but end of beta and beginning of glucan less homologous |
|  | 2 | 3 |  | 5 | 3439323 | ►ITR► | 3441263 | 2529 bp conserved - 566 bp ITR - 261 bp hypothetical | no homology | yes, part of conserved gene is homologous, but other gene and rest of conserved gene is not |
|  |  | 3 |  |  | 3442264 | ◄ITR► | 3442090 | 261 bp hypothetical - 160 bp ITR - 597 bp hypothetical | no homology | no homology |
| 1 |  |  |  |  | 3443961 | ►ITR► | 3442847 | 597 bp hypothetical - 17 bp ITR - 1509 bp hsdM | no homology | no homology |
| 1 | 2 |  | 4 | 5 | 3460680 | ►ITR► | 3460510 | 1857 bp conserved - 87 bp ITR - 573 bp RNA | no homology | no homology |
|  |  | 3 | 4 |  | 3514543 | ►ITR► | 3514560 | 426 bp conserved - 165 bp ITR - 627 bp yggA | not homologous | yes, conserved is not homologous, C-terminal part of yggA is not homologous |
| 1 | 2 |  | 4 | 5 | 3593047 | ►ITR◄ | 3592824 | 876 bp ampR - 22 bp ITR - 501 bp conserved | no change | no |
|  | 2 |  |  |  | 3599201 | ►ITR► | 3601149 | 2420 bp bfeA - 447 bp ITR - 2252 bp bfeA | less homology in ITR, ITR in Xac is shorter | no |
|  |  | 3 |  | 5 | 3616176 | ►ITR► | 3619321 | 960 bp transcriptional - 40 bp ITR - 735 bp cobS | flanking region changes | yes, cobS is homologous, transcriptional is not homologous |
| 1 |  |  |  |  | 3630526 | ►ITR► | 3628796 | 537 bp conserved - 179 bp ITR - 1881 bp btuB | less homology in ITR, ITR in Xac is longer | no |
|  | 2 | 3 |  |  | 3649805 | ►ITR► | 3648379 | 759 bp ostB - 49 bp ITR - 779 bp conserved | no change | no |
|  |  | 3 |  | 5 | 3664245 | ►ITR► | 3661739 | 882 bp comL - 450 bp ITR - 987 bp conserved | less homology in ITR, ITR in Xac is shorter | no |
| 1 |  |  | 4 |  | 3673576 | ►ITR► | 3675915 | 1257 bp pilC - 6 bp ITR - 864 bp pilD | no change | no |
| 1 | 2 |  |  | 5 | 3676041 | ►ITR◄ | 3679208 | 492 bp conserved - 142 bp ITR - 1157 bp colS | flanking region changes | yes, conserved is not homologous, colS is homologous |
|  |  | 3 |  |  | 3680766 | ►ITR► | 3680508 | 1157 bp colS - 169 bp ITR - 678 bp colR | no change | no |
| 1 | 2 |  |  | 5 | 3682729 | ►ITR► | 3681355 | 678 bp colR - 649 bp ITR - 876 bp rimK | less homology in ITR, gene inserted in ITR in Xac | y/n, genes are homologous, but gene inserted in ITR |
|  | 2 |  |  | 5 | 3683792 | ►ITR◄ | 3685502 | 2129 bp glgX - 538 bp ITR - 393 bp xrvA | gene inserted in ITR in Xac | y/n, genes are homologous, but gene inserted in ITR |
|  |  | 3 |  | 5 | 3698885 | ►ITR◄ | 3698634 | 1023 bp conserved - 358 bp ITR - 1368 bp IS1478 | several genes inserted between homologous  genes | gene homologous and part of ITR, but distance between homologous regions is 300000bp in Xac! |
| 1 |  |  |  | 5 | 3711041 | ►ITR► | 3713831 | 3263 bp DNA - 324 bp ITR - 267 bp truncated | no homology | no homology |
|  | 2 |  | 4 | 5 | 3723415 | ►ITR◄ | 3723679 | 885 bp regulatory - 27 bp ITR - 294 bp conserved | no homology | no homology |
|  |  | 3 | 4 |  | 3730151 | ◄ITR► | 3730563 | 785 bp estA - 188 bp ITR - 201 bp conserved | no change | no |
| 1 |  |  |  |  | 3731067 | ►ITR► | 3730952 | 201 bp conserved - 48 bp ITR - 795 bp thiG | no change | no |
| 1 | 2 | 3 |  | 5 | 3747071 | ►ITR► | 3745625 | 2936 bp iroN - 137 bp ITR - 2672 bp glycosyl | no change | no |
|  |  | 3 |  | 5 | 3748525 | ◄ITR► | 3748435 | 2673 bp glycosyl - 964 bp ITR - 1965 bp susB | no change | no |
| 1 | 2 |  | 4 |  | 3763454 | ►ITR► | 3763644 | 1086 bp RND - 262 bp ITR - 1976 bp nodQ | no change | no |
|  | 2 |  | 4 |  | 3800050 | ◄ITR► | 3799877 | 813 bp endonuclease - 410 bp ITR - 519 bp IS1480 | flanking region changes | yes, endonuclease is homologous, but IS is not |
|  |  | 3 |  |  | 3815080 | ◄ITR► | 3815394 | 831 bp reductase - 34 bp ITR - 912 bp transcriptional | no homology | no homology |
| 1 |  | 3 | 4 | 5 | 3832222 | ►ITR► | 3833464 | 939 bp - 53 bp ITR - 1020 bp | no change | no |
|  | 2 |  |  |  | 3840936 | ◄ITR► | 3839824 | 1082 bp pilM - 95 bp ITR - 2522 bp mrcA | no change | no |
|  |  | 3 |  |  | 3849215 | ►ITR► | 3849234 | 381 bp yjgF - 138 bp ITR - 1545 bp D | flanking region changes | yes, yjgF present, D not homologous |
| 1 |  |  |  | 5 | 3851405 | ►ITR► | 3852220 | 1271 bp porin - 77 bp ITR - 1355 bp tcbD | no homology | no homology |
| 1 | 2 |  | 4 |  | 3865876 | ►ITR► | 3865271 | 624 bp Ham1 - 16 bp ITR - 1158 bp oxidoreductase | no change | no |
|  | 2 | 3 | 4 |  | 3879927 | ►ITR◄ | 3879693 | 785 bp estA - 188 bp ITR - 201 bp sulfur | no change | no |
| 1 |  |  | 4 | 5 | 3910360 | ►ITR► | 3909705 | 1256 bp pfkA - 580 bp ITR - 762 bp hypothetical | flanking region changes | yes, pfkA is homologous, hypothetical is not |
|  | 2 | 3 |  |  | 3916141 | ►ITR► | 3915376 | 1346 bp conserved - 97 bp ITR - 1091 bp virB6 | no homology | yes, only part of hypothetical protein is homologous |
|  |  | 3 | 4 |  | 3959764 | ◄ITR► | 3961100 | 1440 bp leuC - 124 bp ITR - 903 bp transcriptional | no change | no |
| 1 |  |  |  |  | 3982535 | ►ITR► | 3982359 | 2328 bp maeB - 147 bp ITR - 1374 bp dctA | less homology in ITR, ITR in Xac is shorter | no |
|  | 2 |  | 4 | 5 | 3986689 | ►ITR► | 3985315 | 1169 bp oprO - 115bp ITR - 2645 bp sensor | less homology in ITR, ITR in Xac is longer | no |
|  |  | 3 | 4 |  | 4047496 | ►ITR► | 4047435 | 1979 bp betT - 40 bp ITR - 1472 bp betB | no change | no |
| 1 |  |  | 4 | 5 | 4073457 | ►ITR► | 4073380 | 474 bp xcsH - 60 bp ITR - 465 bp xcsG | less homology in ITR | no |
| 1 | 2 |  |  | 5 | 4075273 | ►ITR► | 4076614 | 1499 bp xcsE - 4 bp ITR - 2072 bp xcsD | no change | no, but less homology in nterm part of xcsH |
|  |  | 3 |  |  | 4089082 | ►ITR► | 4087878 | 507 bp folK - 96 bp ITR - 1502 bp histidine | no change | no |
| 1 | 2 | 3 | 4 | 5 | 4098153 | ►ITR► | 4096790 | 378 bp conserved - 125 bp ITR - 864 bp conserved | no change | no |
|  | 2 |  | 4 |  | 4100505 | ►ITR► | 4101362 | 1407 bp pbeF - 59 bp ITR - 1056 bp bifunctional | less homology in ITR, ITR in Xac is longer | no |
|  |  | 3 | 4 | 5 | 4147161 | ►ITR► | 4147037 | 279 bp ISxac3 - 35 bp ITR - 1739 bp mxaF | flanking region changes | yes, genes are homologous but in different genomic regions |
| 1 |  |  | 4 |  | 4239615 | ►ITR► | 4237889 | 609 bp conserved - 166 bp ITR - 1080 bp hypothetical | flanking region changes | y/n, conserved gene is homologous, hypothetical not |
|  | 2 | 3 |  |  | 4282067 | ►ITR◄ | 4282543 | 2447 bp conserved - 210 bp ITR - 918 bp GNL | less homology in ITR, ITR in Xac is longer | no, but less homology in hypothetical protein |
|  |  | 3 |  | 5 | 4295357 | ►ITR◄ | 4294595 | 2814 bp glnE - 82 bp ITR - 1575 bp hypothetical | flanking region changing | yes, conserved gene not homologous, glnE homologous except for C-terminal part |
| 1 |  |  | 4 | 5 | 4304233 | ◄ITR► | 4303258 | 534 bp conserved - 197 bp ITR - 1646 bp mdcA | less homology in ITR, ITR in Xac is longer | no |
| 1 | 2 |  | 4 | 5 | 4312716 | ►ITR► | 4311802 | 1356 bp matC - 79 bp ITR - 720 bp mdcY | no change | no |
|  |  | 3 |  |  | 4315964 | ◄ITR► | 4317415 | 2082 bp icfG - 209 bp ITR - 699 bp conserved | less homology in ITR, ITR in Xac is shorter | no, but less homology in beginning of icfG |
| 1 | 2 |  |  |  | 4343187 | ►ITR► | 4342809 | 1335 bp sndH - 88 bp ITR - 306 bp conserved | no change | no |
|  | 2 |  |  |  | 4349143 | ◄ITR► | 4349555 | 1289 bp dadA - 150 bp ITR - 480 bp lrp | no change | no |
|  |  | 3 | 4 |  | 4385908 | ►ITR► | 4385836 | 900 bp conversed - 81 bp ITR - 345 bp conserved | no change | no |
| 1 |  |  | 4 | 5 | 4399638 | ►ITR► | 4400509 | 1152 bp UDP - 35 bp ITR - 1185 bp conserved | no homology | no homology |
|  | 2 |  |  | 5 | 4446760 | ►ITR► | 4447492 | 1134 bp ribA - 139 bp ITR - 477 bp conserved | no change | no |
|  |  | 3 |  |  | 4464087 | ►ITR► | 4462764 | 651 bp conserved - 180 bp ITR - 2496 bp topA | no change | no |
| 1 |  |  | 4 | 5 | 4484780 | ►ITR► | 4485661 | 795 bp response - 56 bp ITR - 951 bp ftsX | no change | no |
| 1 | 2 | 3 |  | 5 | 4490044 | ►ITR◄ | 4491770 | 1280 bp rho - 225 bp ITR - 924 bp hypothetical | less homology in ITR, hypothetical gene  inserted in Xcc | y/n, rho is homologous, but gene inserted in ITR in Xcc, next gene homologous again aceK |
|  |  | 3 | 4 |  | 4534139 | ►ITR► | 4534441 | 2171 bp bglX - 255 bp ITR - 354 bp folB | less homology in ITR, ITR in Xac is shorter | no |
| 1 | 2 |  | 4 |  | 4579421 | ►ITR◄ | 4577906 | 447 bp conserved - 313 bp ITR - 1395 bp algC | less homology | no, but hypothetical gene is less homologous |
|  | 2 |  | 4 | 5 | 4585398 | ►ITR► | 4584842 | 1689 bp argS - 46 bp ITR - 861 bp conserved | less homology in ITR, ITR in Xac is longer | no |
|  |  | 3 |  | 5 | 4620201 | ►ITR◄ | 4620593 | 891 bp sulA - 47 bp ITR - 1383 bp pyridine | flanking region changes | yes, sulA is homologous, pyridine is not |
| 1 |  | 3 | 4 |  | 4623283 | ►ITR► | 4624618 | 1434 bp ygiY - 100 bp ITR - 288 bp conserved | no homology | y/n, parts of ygiY are homologous, conserved gene is not |
|  | 2 |  | 4 | 5 | 4633991 | ►ITR► | 4633354 | 1371 bp leucine - 242 bp ITR - 1092 bp srpA | insertion, prtR and prtI inserted in Xac | no |
|  |  | 3 | 4 | 5 | 4651400 | ►ITR► | 4653133 | 1611 bp peptidase - 206 bp ITR - 954 bp beta | less homology in ITR, ITR is same length | no |
| 1 |  |  |  | 5 | 4708778 | ◄ITR► | 4708466 | 273 bp conserved - 65 bp ITR - 996 bp czcD | no change | no, less homologous part in czcD, both genes present |
| 1 | 2 |  | 4 | 5 | 4721754 | ►ITR► | 4719509 | 1044 bp nrdB - 179 bp ITR - 2484 bp nrdA | no change | no |
|  |  | 3 | 4 |  | 4723570 | ►ITR► | 4724292 | 1044 bp integral - 106 bp ITR - 2709 bp conserved | no change | no |
| 1 | 2 |  |  | 5 | 4812813 | ►ITR► | 4811401 | 969 bp peptidyl - 3 bp ITR - 1167 bp conserved | no change | no, but less homology in beginning of peptidyl |
|  | 2 | 3 | 4 | 5 | 4828936 | ►ITR◄ | 4829768 | 1326 bp RTS - 1064 bp ITR - 378 bp conserved | less homology, ITR in Xac shorter | no |
|  |  | 3 | 4 |  | 4866835 | ►ITR► | 4866149 | 228 bp tatA - 72 bp ITR - 923 bp conserved | no change | no |
| 1 |  |  |  |  | 4875340 | ◄ITR► | 4875350 | 1086 bp salR - 152 bp ITR - 2220 bp aguA | no change | no |
|  | 2 |  |  | 5 | 4955743 | ►ITR◄ | 4952686 | 1184 bp benE - 311 bp ITR - 1904 bp aphB | less homology in ITR, ITR in Xac is shorter | no |
|  |  | 3 | 4 | 5 | 4957179 | ►ITR► | 4956850 | 1262 bp tetA - 554 bp ITR - 2159 bp transglycolase | less homology in ITR, ITR in Xac is shorter | no |
| 1 |  |  |  |  | 4984224 | ►ITR◄ | 4984226 | 1218 bp cls - 31 bp ITR - 888 bp hypothetical | no change | no |
| 1 | 2 |  |  |  | 5016712 | ►ITR► | 5016567 | 3405 bp recC - 295 bp ITR - 5228 bp hemaglutinin | less homology in ITR | yes, recC is homologous, beginning of hemaglutinin is homologous to hypothetical gene, but then flanking region changes |
|  |  | 3 |  | 5 | 5043346 | ►ITR► | 5042221 | 2475 bp conserved - 97 bp ITR - 2931 bp conserved | no homology | yes, first conserved gene is present, but less homology in beginning and different flanking region |
| 1 | 2 |  | 4 | 5 | 5056051 | ►ITR◄ | 5055798 | 1871 bp avrXccA1 - 222 bp ITR - 318 bp conserved | flanking region changing | yes, conserved gene is homologous, avr is not |
|  | 2 |  |  |  | 5072157 | ◄ITR► | 5064549 | 2351 bp fecA - 159 bp ITR - 843 bp phoC | less homology in ITR, ITR in Xac is longer | no |
|  |  | 3 | 4 |  | 5069676 | ►ITR◄ | 5069375 | 3045 bp oar - 91 bp ITR - 1341 bp thdF | flanking region changes | yes, thdF in Xcc is homologous to trmE in Xac, other gene is not present |
| 1 |  |  |  |  | 5071853 | ►ITR► | 5070807 | 1340 bp thdF - 22 bp ITR - 2858 bp polysaccharide | less homology in ITR, ITR in Xac is longer | no |
| 1 | 2 | 3 |  | 5 | 5073484 | ►ITR► | 5075346 | 1721 bp 60kDa - 15 bp ITR - 447 bp rnpA | no change | no |
