# Supplementary material for: Investigation of a Quadruplex-Forming Repeat Sequence Highly Enriched in Xanthomonas and Nostoc sp
Source: PLoS One. 2015 Dec 22;10(12):e0144275. doi: 10.1371/journal.pone.0144275 (PMC4692102; doi:10.1371/journal.pone.0144275)
Supplement: S6 File — (DOCX) [file pone.0144275.s006.docx]

**Supporting Information 6:**

**Contents:**

S6 File. Analysis of RNA sequencing data by Jalan et al. for repeat containing transcripts

Analysis of RNA sequencing data by Jalan et al. for repeat containing transcripts

Table shows the analysis of RNA sequencing data of *Xac* made publicly available by Jalan et al. (Jalan, N., Kumar, D., Andrade, M.O., Yu, F., Jones, J.B., Graham, J.H., White, F.F., Setubal, J.C. and Wang, N. (2013) Comparative genomic and transcriptome analyses of pathotypes of Xanthomonas citri subsp. citri provide insights into mechanisms of bacterial virulence and host range. *BMC genomics*, **14**, 551.) for repeat-containing transcripts. Sequences of the respective repeats are listed and formation of inverted repeats (inv rep) is indicated with the number of the partnering repeat. If a repeat was found to be part of an assembled transcript is shown by yes/no/partial; which strand of the repeat was found on the transcript is indicated by G or C. Start and end point of an assembled transcript on the *Xac* genome is stated, its location on the plus or minus strand of the genome and its respective expression level in FPKM. If several repeats were found on one transcript is indicated by the repeat number of the respective repeat. Repeats were sorted according to the following criteria: 1) the transcript starts within the repeat and is a) sense (1A) or b) anti-sense to the following gene (1B), 2) the transcript ends within the repeat sequence or shortly thereafter (max. 30 nt) and is a) sense (2A) or b) anti-sense (2B) to the last gene transcribed and 3) the repeat sequences is located somewhere in the middle of the transcript and it is a) sense (3A) or b) anti-sense (3B) to the neighboring genes or c) the transcript covers convergent genes (3C). [n.a. = not available]

| **#** | **length [nt]** | **sequence** | **inv  rep** | **transcribed** | **G or C** | **RNA start** | **RNA stop** | **expr level** | **on same RNA** | **comments** |
| --- | --- | --- | --- | --- | --- | --- | --- | --- | --- | --- |
| 1 | 21 | GGGAATGGAGAATCGGGAATC | 2 | yes | c | 19220 | 19435 | 0 | 2 | starts within 2, sense with both neighboring genes |
| 2 | 28 | GGGAATAGGGAATCGGGAATGGGGAATC | 1 | yes (partial) | g | 19220 | 19435 | 0 | 1 | see above |
| 3 | 28 | GGCAATCGGGAATCGGGAATCGGGAATC | no | no |  |  |  |  |  | n.a. |
| 4 | 21 | GAGAATCGGGAATCGGGAATT | other | yes | c | 166377 | 168093 | 338.65 | other (g) | in middle of long transcript, *anti-sense* to both neighboring genes |
| 5 | 21 | GTGAATCGGGAATTGGGAATC | no | yes | c | 168109 | 168543 | 14.28 |  | 5 is at beginning of transcript, *anti-sense* to *kdgK*, transcript stops within *kdgK* |
| 6 | 21 | GGGATTCGGGACTCGGGAATC | 7 | yes | c | 385348 | 386512 | 43.65 | 7 | repeats are in middle of long transcript over convergent genes |
| 7 | 28 | GTGAATGGGGAATTGGGAATGGGGAATC | 6 | yes | g | 385348 | 386512 | 43.65 | 6 | see above |
| 8 | 49 | GAGAATCGGGAATCGGGAATCGGGAATCGGGAATCGGGAATCGGGAATC | no | yes (partial) | g | 565424 | 565764 | 21.63 |  | transcript stops within the middle of 8, sense with neighboring gene, repeat overlaps with stop codon |
| 9 | 21 | GTGAATCGGGAATCGGGAATC | no | yes | g | 614829 | 615058 | 0 |  | in middle of short transcript, sense with neighboring genes, does not contain whole gene |
| 10 | 28 | GGGAATCGGGAGTGGAGAATGGGGAATC | no | yes | g | 619400 | 621796 | 305.56 |  | repeat is in the middle of long transcript spanning three genes, repeat and another G-rich sequence are at the beginning and end of second gene (hypothetical), sense with neighboring genes |
| 11 | 28 | GGGAATCGGGAGTTGGGAATCGGGAATG | 12 | yes | c | 632157 | 634311 | 3832.87 | 12 | repeats are in the middle of long transcript, *anti-sense* to both neighboring genes |
| 12 | 28 | GGGAATCGGGAGTGGAGAATGGGGAATC | 64 | yes | g | 632157 | 634311 | 3832.87 | 11 | see above |
| 13 | 28 | GGGAATTGGGAATTGGGAATTGGGAATC | no | no |  |  |  |  |  | n.a. |
| 14 | 21 | GTGAATCGGGAATCGGGAACG | other | yes | c | 700961 | 702476 | 38.85 | other (g) | repeats are in the middle of long transcript, neighboring genes are convergent, RNA stops within second gene (*anti-sense*) |
| 15 | 28 | GTGAATCGGGAGTGGGGAATCGGGAATC | 16 | yes (partial) | g | 752404 | 753922 | 280.19 | 16 | repeats are at the beginning of transcript, transcript starts within 15, sense with *hslU* |
| 16 | 35 | GGCATTAGGGAATCGAGAATTGGGAAAGGGGAATC | 15 | yes | c | 752404 | 753922 | 280.19 | 15 | see above |
| 17 | 28 | GGGAATGGGGAATCGGGAATGGGGAATC | 18 | no |  |  |  |  |  | n.a. |
| 18 | 35 | GGGAATCGGGAATCGGGAATCGGGAATCGGGAATG | 17 | no |  |  |  |  |  | n.a. |
| 19 | 28 | GGGAATCGGGAATCGGGAAAGGGGAATC | 20 | yes | c | 776803 | 778251 | 150.85 | 20 | repeats are in the middle of transcript, *anti-sense* to both neighboring genes |
| 20 | 35 | GGGAATGGGGAGTCGGGAATGGGGAATCGGGGAGC | 19 | yes | g | 776803 | 778251 | 150.85 | 19 | see above |
| 21 | 35 | TGGAATGGGGAATCGGGAATTGGGAATTGGGAATG | no | yes | g | 778230 | 778988 | 18.29 |  | repeats are near the end of transcript, sense with *mreC*, transcript stops within *mreC*, shortly after repeat (intragenic repeat) |
| 22 | 28 | GGGAATCGGGAATCGGGAATCGGGAATC | no | yes | g | 779376 | 779670 | 51.3 |  | repeats are at the end of transcript, *anti-sense* to *mreC* and *mreD* (intragenic repeat) |
| 23 | 28 | GCGAATCGGGAATCGGGAATCGGGAATC | no | yes (partial) | g | 789389 | 789703 | 110.74 |  | transcript stops within repeat, sense with hypothetical gene but does not cover whole gene |
| 24 | 29 | GGGAAATGGGGAGTCGGGAATGGGGAATC | 25 | yes | g | 890250 | 892171 | 92.3 | 25 | repeats are in the middle of transcript spanning three genes, between second and third, sense with neighboring genes |
| 25 | 28 | GGGAATCGGGAAAAGGGAATCGGGAATC | 24 | yes | c | 890250 | 892171 | 92.3 | 24 | see above |
| 26 | 28 | GGGAATCGGGAATCGGGAATCGGGAATC | other | yes (partial) | c | 901232 | 901926 | 16.32 | other is not transcribed | starts within 26, sense with *kpdD* |
| 27 | 28 | GGGATTGGGGAATGGGGAATCGGGAATG | 28 | yes | c | 915707 | 920007 | 100.09 | 28 | repeats are in the middle of transcript spanning several genes, *anti-sense* to all genes (intragenic repeats) |
| 28 | 35 | GGGAATGGGGAATCGGCAGTCGGGAATCGGGAAGC | 27 | yes | g | 915707 | 920007 | 100.09 | 27 | see above |
| 29 | 28 | GCGAATCGGGAATCGGGAATCGGGAATG | other | yes (partial) | c | 924225 | 925123 | 149.58 | other (g) | transcript stops within 29, sense with gene; transcript containing 31, 30, 32 also starts with 29, sense (could this be one transcript?) (intragenic repeat) |
| 30 | 28 | GGGAATCGTGAATCGTGAATCGGGAATG | 31 | yes | g | 925100 | 929317 | 134.92 | 31 | repeats are in the middle of transcript, sense with both neighboring genes |
| 31 | 28 | GGGAATTGGGAGTCGGGAATAGGGAATC | 30 | yes | c | 925100 | 929317 | 134.92 | 30 | see above |
| 32 | 14 | GGGAATCGGGAATG | no | yes | g | 925100 | 929317 | 134.92 |  | see above (intragenic repeat) partial 29, 30, 31, 32 are all on same transcript |
| 33 | 21 | GGGAATCGGGATTCGAGAATT | 34 | yes | g | 929311 | 931148 | 513.43 | 34 | repeats are in the middle of transcript, sense with genes |
| 34 | 28 | GGGAATGGGAAGTTGGAAATGGGGAATC | 33 | yes | c | 929311 | 931148 | 513.43 | 33 | see above |
| 35 | 28 | GTGAATGGGGAATCGGGAATGGGGAATC | no | yes | c | 929311 | 931148 | 513.43 |  | see above  33 34 35 are all on same transcript |
| 36 | 14 | GGGCATTGGGAATC | no | yes | g | 940227 | 940565 | 18.28 |  | repeat is in the middle of short transcript, sense with gene, transcript does not cover whole gene (intragenic repeat) |
| 37 | 84 | GGGAATCGGGAATCGGGAATCGGAAATCGGAAATCGGAAATCGGAAATCGGAAATCGGAAATCGGAAATCGGAAATCGGAAAAG | no | yes (partial) | g | 1065874 | 1066954 | 41.12 |  | stops within 37, sense with gene |
| 38 | 14 | GGGAGACGGGAATC | no | yes | c | 1114414 | 1116122 | 955.06 |  | repeat is in the middle of transcript, sense with both neighboring genes |
| 39 | 14 | GGGAATCGGGAATC | no | yes | c | 1117190 | 1133520 | 1893.06 |  | repeat is in the middle of transcript, sense with both neighboring genes |
| 40 | 21 | GGGAATGGAGAATCGGGAATC | 41 | no |  |  |  |  |  | n.a. |
| 41 | 28 | GGGAATCGGGAATCGGGAATCGGGAATA | 40 | no |  |  |  |  |  | n.a. |
| 42 | 14 | GGGAATTGGGAATC | no | yes | g | 1208044 | 1209034 | 274.31 |  | repeat is near the end of transcript, sense with hypothetical gene |
| 43 | 28 | GAGAATTGGGAATTGGGAATGGGGAATC | 44 | yes | g | 1261534 | 1262990 | 239.91 | 44 | repeats are in the middle of long transcript, sense with genes |
| 44 | 28 | TGGAATCGGGAATCGGGAGTAGGGAATT | 43 | yes | c | 1261534 | 1262990 | 239.91 | 43 | see above |
| 45 | 28 | GGGAATAGGGAATCGGGATTGGGGAATC | other | yes | c | 1261534 | 1262990 | 239.91 | other (g) | 43 44 45 and other are all on same transcript |
| 46 | 21 | GGGAATCGGGAATCGGGATTC | no | yes | g | 1347587 | 1351123 | 40.49 |  | repeat is in the middle of long transcript, covering convergent genes, hypothetical gene and 46 are sense with transcript |
| 47 | 28 | GGGAATCGGGAGTAGGGAATCGGGAATC | 48 | yes | c | 1421783 | 1426393 | 453.88 | 48 | repeats are close to beginning of long transcript spanning several genes, neighboring genes are *anti-sense* to transcript |
| 48 | 28 | GGGAATTGAGAATAGGGAATAGGGAATC | 47 | yes | g | 1421783 | 1426393 | 453.88 | 47 | see above |
| 49 | 14 | GGGAACCGGGAATC | no | yes | c | 1431407 | 1439830 | 642.75 |  | repeat is in the middle of a really long transcript, *anti-sense* to everything (intragenic repeat) |
| 50 | 126 | CGGAATCGGGAATCGGGAATCGGGAATCGGGAATCGGGATTCGGGATTCGGGATTCGGGATTCGGGATTCGGGATTCGGGATTCGGGATTCGGGATTCGGGATTCGGGATTCGGGATTCGGGCAAT | no | yes (partial) | c | 1442060 | 1443644 | 71.55 |  | transcript starts within 50, sense to *radA* |
| 51 | 49 | GGGATTGGGGATTGGCGAGTCGGGAATCGGGAATCGGGAATCGGGAATC | no | yes (partial) | g | 1477552 | 1530627 | 519.33 | 52;  53,55 | transcript starts within 51, sense to *ffh* |
| 52 | 42 | GGGAATCGGGAATCGGGAATCGGGAATCGGGAATCGGGAATC | no | yes | g and c | 1477552  1491655  1491875 | 1530627  1491872  1492129 | 519.33  30.59  28.04 | 51  --  -- | part of three transcripts: in the middle of transcript with 51, *anti-sense* to both neighboring genes, second RNA ends within 52, *anti-sense* to neighboring gene; third RNA ends within 52, but relatively short and sense with *mutS* |
| 53 | 28 | GGGAATGGAGAATCGGGAATCGGGAATG | 54 | yes | g | 1477552  1525220 | 1530627  1527696 | 519.33  162.91 | 52  54 | part of two transcripts:first with 52, sense to both neighboring genes; second with 54, sense to both neighboring genes |
| 54 | 28 | GGGAATTGGGAATCGGGAGTGGGGAATC | 53 | yes | c | 1525220 | 1527696 | 162.91 | 53 | see above |
| 55 | 28 | GGGAATGGTGAATCGGGAATCGGGAATC | 56 | yes | g | 1477552  1527677 | 1530627  1529275 | 519.33  433.4 | 51,52,53 56 | part of two transcripts: on long transcript with 51-53 (see above), sense to both neighboring genes; second transcript ends after 55, sense to *lepA* |
| 56 | 28 | GGGAATCGAGAATCGCGAATGGGGAATA | 55 | yes | c | 1527677 | 1529275 | 433.4 | 55 | see above |
| 57 | 28 | GTGAATCGGGAGTAGGCAGTAGGGAATC | 58 | yes (partial) | g | 1534340  1535754 | 1535764  1536899 | 42.77  77.21 | 58 | first RNA stops within 58, sense with *rumA*; second transcript also stops within 58, but is anti-sense to hypotherical and following gene |
| 58 | 35 | GGGAATCGGGAATCGGGAATCGGGAATCGGGAATG | 57 | yes | c | 1534340  1535754 | 1535764  1536899 | 42.77  77.21 | 57 | see above |
| 59 | 27 | GGGATTGGGAGTTGGGAATCGGGAATC | no | yes | c | 1592161 | 1594488 | 215.47 |  | repeat is near beginning of transcript; transcript is *anti-sense* to *metG*; stops shortly before 60 |
| 60 | 28 | GGGAATAGGGAGTCGGGAATCGGGAATT | no | yes | g | 1594473 | 1595348 | 211.34 |  | repeat is at the beginning of transcript, is *anti-sense* to following gene |
| 61 | 21 | GGGAATCGGGAGTCGGGAATC | 62 | no |  |  |  |  |  | n.a. |
| 62 | 28 | GTGAATCGGGAATTGGGAATCGGGCGAG | 61 | yes (partial) | g | 1610982 | 1612477 | 20.74 | alone, without 61 | transcript stops between 62 and 61, but is *anti-sense* to all genes |
| 63 | 28 | CGGAATCGGGAATGGAGAATCGGGAATC | 64 | yes (partial) | c | 1617860 | 1618196 | 71.56 | 64 | transcript stops within 63, sense with *dnaE* (not all of *dnaE* on transcript) |
| 64 | 21 | GGGAATCGGGAATGGGGAATC | 63 | yes | g | 1617860 | 1618196 | 71.56 | 63 | see above |
| 65 | 28 | GGGAATCGGGAATGGGGAATGGGGAATC | 66 | no |  |  |  |  |  | n.a. (intragenic repeat) |
| 66 | 28 | GCGAATCGGGAATGGAGAATCGGGAATC | 65 | no |  |  |  |  |  | n.a. (intragenic repeat) |
| 67 | 28 | GGGAATCGGGAGTGGGGAATCGGGAATC | 68 | yes (partial) | g | 1623735 | 1626497 | 156.57 | 68 | long transcript starts within 67, is *anti-sense* to *lpxB* and *lpxA* |
| 68 | 28 | GGGAATTGGGAATCGGGAGTCGGGAATC | 67 | yes | c | 1623735 | 1626497 | 156.57 | 67 | see above |
| 69 | 28 | GGGAATCGGGATTGGGGAATCGGGAATC | no | yes | g | 1631181 | 1635010 | 241.62 |  | repeat is in the middle of transcript, sense with neighboring genes |
| 70 | 35 | GGGAATCGGGAATCGGGAATCGGGAATCGGGAATC | other | yes (partial) | c | 1638654 | 1638910 | 17.89 | other (g) | stops within 70, sense with pili gene (not all of gene on transcript) |
| 71 | 35 | GGGAATCGGGAATCGGGAATTGGGAATCGGGAATG | 72 | yes (partial) | c | 1684581 | 1684786 | 54.82 | 72 | stops within 71, sense with *msbA* (not all of gene on transcript) |
| 72 | 21 | GGGAATGGGGAATGGGGAATT | 71 | yes | g | 1684581 | 1684786 | 54.82 | 71 | see above |
| 73 | 35 | GTGATTCGGGAAGCGGCAGTGGGGAGTCGGGAATC | 74 | yes | g | 1748303 | 1751304 | 160.49 | 74 | repeats are in the middle of a long transcript spanning several genes, repeat between convergent genes, sense to serine gene |
| 74 | 28 | GTGAATTGGGAATCGGGAATCGGGAATC | 73 | yes | c | 1748303 | 1751304 | 160.49 | 73 | see above |
| 75 | 28 | CGGAATCGGGAGTGGGGAATCGGGAATG | 76 | yes (partial) | g | 1770436 | 1773063 | 485.08 | 76 | transcript starts within 75 *anti-sense* to *ldp*, and following gene |
| 76 | 28 | GGGAATCGGGAGTGGGGAATCGGGAATC | 75 | yes | c | 1770436 | 1773063 | 485.08 | 75 | see above |
| 77 | 28 | GGGAATGGGGAATAGGGAATGGGGAATC | 78 | yes | g | 1770538 | 1773063 | 112.61 | 78 | repeats are in the middle of transcript, *anti-sense* to both neighboring genes |
| 78 | 28 | GGGAATCGGGAATGGAGATACGGGAATG | 77 | yes | c | 1770538 | 1773063 | 112.61 | 77 | see above |
| 79 | 21 | GGGAATCGGGATTCGGGAGTC | 80 | yes | g | 1816626  1817134 | 1817146  1818046 | 22.69  75.81 | 80 | 80 is part of several transcripts: first stops within 80, sense to hypothetical gene (not all of gene on transcript); second starts within 80 but *anti-sense* to following gene |
| 80 | 28 | GGGAATCGGGAATCGGGAATCGGGAATC | 79 | yes | c | 1816626  1817134 | 1817146  1818046 | 22.69  75.81 | 79 | see above |
| 81 | 28 | GGGATTCGGGAATCGGGATTTGGGAATC | no | yes | c | 1864622 | 1866661 | 1664.72 |  | repeat is in the middle of transcript, sense to neighboring genes |
| 82 | 21 | CTGAATCGGGAATCGGGAGAG | other | yes | c | 1988381 | 1989570 | 32.66 | other (g) | repeats are in the middle of transcript, between convergent genes |
| 83 | 35 | GGGATTGGGGAATCGGGATTCGGGATTCGGGATTC | other | yes | c | 2139316 | 2141316 | 66.91 | other (g) | repeats are in the middle of transcript between convergent genes |
| 84 | 28 | GGGAATCGGGAGTAGGGAATGGGGAATC | 85 | yes (partial)yes | c | 2145035 | 2146374 | 217.33 | 85 | starts within 85, sense to *yeiP* |
| 85 | 28 | GGGATTGGGGAATCGGGAATGGGGAATC | 84 | yes | g | 2145035 | 2146374 | 217.33 | 84 | see above |
| 86 | 28 | GGGAATCGGGAATCGGGAATCGGGAATC | no | no |  |  |  |  |  | n.a. (intragenic repeat) |
| 87 | 71 | GGGATTCGGGATTCGGGATTCGGGATTCGGGATTCGGGATTGGGATAATCGGGAATCGGGAATCGGGAATC | no | no |  |  |  |  |  | n.a. |
| 88 | 28 | GGGAATCGGGATTGGGCATGGGGGAATC | no | yes | g | 2264298 | 2265483 | 40.27 |  | transcripts stops after 88, but *anti-sense* to *flhB* |
| 89 | 28 | GGGAATCGGGAATGGAGAATCGGGAATC | 90 |  |  |  |  |  | 90 |  |
| 90 | 28 | GGGCATGGGGAATGGGGAGTAGGGAATC | 89 |  |  |  |  |  | 89 | see above |
| 91 | 21 | GGGAATGGGGAATGGGGAATC | 92 | no |  |  |  |  |  | n.a. |
| 92 | 105 | GGGAATCGGGAATCGGGAATCGGGAATCGGGAATCGGGAATCGGGAATCGGGAAGCGGGAATCGGGAAGCGGGAATCGGGAAGCGGGAAGCGGGAAGCGGGAAGC | 91 | no |  |  |  |  |  | n.a. |
| 93 | 36 | GGGAATGGGGAGTTGGGAAATGGGGAATCGGGAATC | 94 | yes | c | 2320317 | 2326651 | 129.83 | 94 | repeats are in the middle of transcript, between convergent genes |
| 94 | 28 | GGGAATAGGGAATCGGGATTGGGGAATC | 93 | yes | g | 2320317 | 2326651 | 129.83 | 93 | see above |
| 95 | 28 | GGGAATGGGGAGAGGGGAATCGGGAATC | 96 | yes | g | 2353738 | 2358259 | 165.43 | 96 | repeats are in the middle of transcript, *anti-sense* to both neighboring genes |
| 96 | 28 | GAGAATCGGGAGCGGGGAATCGGGAATG | 95 | yes | c | 2353738 | 2358259 | 165.43 | 95 | see above |
| 97 | 28 | GGGAATCGAGAATCGGGATGAGGGAATC | 98 | yes | g | 2676741 | 2678642 | 256.83 | 98 | repeats are in the middle of transcript, *anti-sense* to both neighboring genes |
| 98 | 29 | GGGTAACCGGGAATGGGGAATCGGGAATC | 97 | yes | c | 2676741 | 2678642 | 256.83 | 97 | see above |
| 99 | 28 | GGGAATGGAGAATCGGGAATAGGGAATC | 100 | yes | c | 2716002 | 2717093 | 53.22 | 100 | see above |
| 100 | 21 | GGGAATCGGGATTCGGGAATC | 99 | yes | g | 2716002 | 2717093 | 53.22 | 99 | repeats are in the middle of transcript, sense to neighboring genes |
| 101 | 14 | GGGAATGGGGAATC | no | yes | c | 2730506 | 2733683 | 2432.3 |  | repeat is near the end of transcript, sense to gene |
| 102 | 28 | GAGAATCGGGAGTGGGGAATCGGGAATG | other | yes | c | 2738512 | 2738948 | 28.29 | other (g) | transcript stops within 102, sense with *proB* |
| 103 | 28 | CGGAATCGGGAATCGGGAATAGAGAATC | other | yes | c | 2746688 | 2748532 | 113.68 | other (g) | repeats are in the middle of transcript, *anti-sense* to both neighboring genes |
| 104 | 28 | GGGAATCGGGAATGGTGAATCGGGAATG | 105 | no |  |  |  |  |  | n.a. |
| 105 | 28 | GGGAATCGGGAGTGGGGAATCGGGAATC | 104 | yes (partial) | g | 2784723 | 2785239 | 33.3 | alone, without 104 | transcript stops within 105, but *anti-sense* to *dbpA* (does not contain whole gene) |
| 106 | 14 | GAGAGTTGGGATAGTGAATCGGGAATC | no | yes | c | 2872322 | 2874153 | 56.07 |  | repeat is in the middle of transcript, between convergent genes |
| 107 | 35 | GGGAATGGGGAGTGGGGAGTGGGGAATCGGGAATG | 108 | yes | c | 2942240 | 2943816 | 64.37 | 108 | repeats are in the middle of transcript, sense to neighboring genes (intragenic repeat) |
| 108 | 28 | GGGAATCGGGACTCGGGAATGGGGAATC | 107 | yes | g | 2942240 | 2943816 | 64.37 | 107 | see above |
| 109 | 28 | GGGAATCGAGAATTGGGACTGGGGAATC | 110 | no |  |  |  |  |  | n.a. |
| 110 | 28 | GTGAATGGAGAATCGGGAATAGGGAATC | 109 | no |  |  |  |  |  | n.a. |
| 111 | 28 | GAGAATCGGGAATGGGGAATGGGGAATC | 112 | yes | g | 3050380 | 3052446 | 99.75 | 112 | middle of transcript, *anti-sense* to both neighboring genes |
| 112 | 28 | GGGAATCAGGAGTTGGGAATCGGGAATC | 111 | yes | c | 3050380 | 3052446 | 99.75 | 111 | see above |
| 113 | 21 | GGGAATCGGGAGTCGGGATTT | other | yes | g and c | 3240044 | 3240859 | 160.76 | other (g and c) | other is part of two transcripts: one starts between inv rep but anti-sense to hypothetical gene; 113 and 114 part of same transcript both repeats have same orientation, but transcript is *anti-sense* to following gene |
| 114 | 28 | GGGAATCGGGAATCGGGATAGGGGAATC | no | yes | c | 3240044 | 3240859 | 160.76 |  | see above |
| 115 | 28 | GGGAATGGGGAATGGGGAATAGGGAATC | other | no |  |  |  |  |  | n.a. |
| 116 | 28 | GGGAATCGGGATTCGGGATTCGGGAATC | other | yes | g | 3257474 | 3257995 | 54.58 | other ( c) | starts within 116, *anti-sense* to *nadD* and following gene (intragenic repeat) |
| 117 | 14 | GGGAATCGGGAATC | no | yes | c | 3269772 | 3271041 | 57.25 |  | repeat is in the middle of transcript between convergent genes |
| 118 | 14 | CGGAATCGGGAATC | no | yes | g | 3422697 | 3424286 | 116.24 |  | repeat is in the middle of transcript, *anti-sense* to both neighboring genes |
| 119 | 28 | GGGAATGGGGAATCGAGAAACGGGAATT | 120 | yes | c | 3429646 | 3432034 | 356.44 | 120 | repeats are in the middle of transcript, sense to neighboring genes |
| 120 | 28 | GGGAATAGCGAATCGGGAATGGGGAATC | 119 | yes | g | 3429646 | 3432034 | 356.44 | 119 | see above |
| 121 | 14 | GGGAATCGGGACGC | no | yes | g | 3434069 | 3434270 | 38.82 |  | short transcript, no genes on transcript |
| 122 | 28 | GGGAATCGGGAATCGGGAATGGAGAATC | 123 | yes | c | 3442296 | 3445314 | 115.29 | 123 | repeats are in the middle of transcript, *anti-sense* to both neighboring genes |
| 123 | 28 | GGGAATGGGGAATGCGGAATGGGGGAAG | 122 | yes | g | 3442296 | 3445314 | 115.29 | 122 | see above |
| 124 | 14 | GGGAATCGGGATGC | no | yes | c | 3468558 | 3470719 | 102.82 |  | repeat is in the middle of transcript, *anti-sense* to both neighboring genes |
| 125 | 42 | GGGAATCGGGAATCGGGAATCGGGAATCGGGAATCGGGAATC | no | yes (partial) | g | 3659228 | 3659637 | 26.13 |  | starts within 125, *anti-sense* to *relA* |
| 126 | 21 | GGGAATGGGGAATCGGGAATG | 127 | yes | g | 3700181 | 3701530 | 50.38 | 127 | 126, 127 are in the middle of transcript, *anti-sense* to *ruvA* and *ruvC* |
| 127 | 35 | GAGAATCGGGAATCGGCAATCGGGAATCGGGAATC | 126 | yes | c | 3700181 | 3701530 | 50.38 | 126 | transcript stops within 129, *anti-sense* to *ruvC* |
| 128 | 28 | GGGAATGGGAAGTCGGGAATCGGGAATG | 129 | yes | g | 3700181 | 3701530 | 50.38 | 129 | see above |
| 129 | 28 | GGGAATCGGGAATGGAGAATCGGGAATC | 128 | yes | c | 3700181 | 3701530 | 50.38 | 128 | 126, 127, 128, 129 all together on one transcript |
| 130 | 28 | GGGAATGGGGAATGGGGAATCGGGAATT | no | yes | g | 3703667 | 3704264 | 42.92 |  | transcript starts within 130, anti-sense to following gene |
| 131 | 28 | GGGAGTCGGGAGTGGGGAATCGGGAATC | 132 | yes | g | 3813752 | 3816151 | 81.32 | alone, without 132 | transcript stops after 131, sense to *pilR* |
| 132 | 28 | GGGAATCGGGGAGTCGGATCAGGGAATC | 131 | no |  |  |  |  |  | n.a. |
| 133 | 28 | GGGATTCGGGAATCGGGAATCGGGAATC | other | yes (partial) | g | 3881898 | 3883285 | 68.29 | other ( c) | starts within 133, sense to both neighboring genes (intragenic repeat) |
| 134 | 28 | GGGAATCGGGAATCGGGAATCGGGAATC | 135 | yes | c | 3921718 | 3922317 | 22.89 | 135 | transcript stops within 134 (intragenic in *nodQ*), sense to *nodQ* and *cysD* (not all of *cysD* on transcript) |
| 135 | 28 | GCGAATGGGGAATCGGGAATGGAGAATC | 134 | yes | g | 3921718 | 3922317 | 22.89 | 134 | (intragenic repeats) |
| 136 | 28 | GGGAATCGCGAGTGGGGAATCGGGAATG | 137 | yes | g | 3924770 | 3925315 | 23.1 | 137 | starts within 136, sense to following gene |
| 137 | 28 | GAGAATCGAGAATCGGGAATGGGGAATC | 136 | yes | c | 3924770 | 3925315 | 23.1 | 136 | see above |
| 138 | 14 | GGGAATCGGGAATC | no | yes | c | 3939311 | 3940580 | 988.76 |  | near beginning of transcript, sense to following gene |
| 139 | 14 | GGGATTCGGGATTC | no | yes | g | 3949844 | 3951353 | 1073.4 |  | repeat is in the middle of transcript, *anti-sense* to both neighboring genes |
| 140 | 21 | GGGAATCGGGAATGGAGAGTC | other | yes | g | 3972987 | 3976631 | 181.72 | other ( c) | repeats are in the middle of transcript between convergent genes |
| 141 | 28 | GGGATTCGGGAATCGGGATTGGGGAATC | no | yes (partial) | g | 3992491 | 3993014 | 113.86 |  | ends within 141, sense with *mrcA* (not all of mrcA on transcript) |
| 142 | 20 | GGGAATCGGATTGGGGAATC | other | yes | g | 3995764 | 3997509 | 285.71 | other ( c) | repeats are in the middle of transcript, sense to neighboring genes |
| 143 | 28 | GGGAATAGGCAATCGGGAATGGGGAATC | other | yes | c | 4002969 | 4004270 | 70.48 | other (g) | repeats are in the middle of transcript, *anti-sense* to both neighboring genes |
| 144 | 28 | GGGAATCGGGAATGGGGAGTTGGGAATC | no | yes | c | 4025471 | 4026549 | 54.33 |  | repeat is in the middle of transcript, anti-sense to both neighboring genes |
| 145 | 28 | GGGAATCGGGAATCGGGAATCGGGAATG | 146 | yes | c | 4040404 | 4040617 | 32.44 |  | stops within 145; sense to neighboring genes |
| 146 | 28 | GGGAATTGGGAATCGGGAATGGGGAATC | 145 | no |  |  |  |  |  | n.a. |
| 147 | 28 | GGGAATCGAGAGTCGGGAATAGAGAATG | other | yes | g | 4078171 | 4080293 | 100.62 | other ( c) | repeats are in the middle of transcript, sense to neighboring genes |
| 148 | 28 | GGGAATCGTGAATAGGGAATGGGGAATC | 149 | no |  |  |  |  |  | n.a. |
| 149 | 27 | GGGAAGGAGAATCGGGAATGGGGAATC | 148 | no |  |  |  |  |  | n.a. |
| 150 | 28 | GGGAATGGGGAATCGGGAGTGGGGAATC | 151 | yes (partial) | g | 4119685 | 4119906 | 14.43 | 151 | transcript starts within 150, sense to following gene |
| 151 | 28 | GGGAATGGGGAATCGGGACTGGGGAATC | 150 | yes | c | 4119685 | 4119906 | 14.43 | 150 | see above |
| 152 | 56 | GGGAATCGGGAATCGGGAATCGGGAATCGGGAATCGGGAATCGGGAATCGGGAATC | no | yes (partial) | g and c | 4120706  4121896 | 4121879  4122462 | 72.89  27.8 |  | two transcripts found: first starts within 152, but is *anti-sense* to following *suc1*; second also starts within 152, but is *anti-sense* to following *cebR*, divergent transcripts |
| 153 | 14 | GGGAATCGGGAATC | no | yes | c | 4175381 | 4177299 | 47.54 |  | repeat is in the middle of transcript, *anti-sense* to transcriptional something gene |
| 154 | 35 | GGGATTGGGGAATCGGGAATCGGGAATCGGGAAAA | no | yes (partial) | c | 4186352 | 4187731 | 46.04 |  | transcript starts within 154, sense with *xpsF* |
| 155 | 28 | GGGAATCGGGAATCGGGAATCGGGAATG | other | yes (partial) | g | 4204733 | 4206381 | 71.78 | other ( c) | transcript starts within 155, sense with *purL* but middle of gene (intragenic repeat) |
| 156 | 35 | GGGAGCTGGGAATCGCGAGTCGGGAATGGGGAATG | 157 | yes | c | 4206502 | 4207134 | 55.2 | 157 | repeats are in the middle of transcript, sense (intragenic repeats) |
| 157 | 28 | GGGAATCGGGAATCGGGAGTCGGGAATC | 156 | yes | g | 4206502 | 4207134 | 55.2 | 156 | see above |
| 158 | 20 | GGGACTCGGGAATGGGAATC | no | no |  |  |  |  |  | n.a. |
| 159 | 28 | GTGAATCGGGAATGGGGAATGGGGAATC | 160 | yes | c | 4215629 | 4217434 | 82.23 | alone, without 160 | transcript starts within 159, but *anti-sense* to *holC* |
| 160 | 42 | GGGAATGGGGAGTCGGGAATAGGGAATAGGGAATAGGGAATG | 159 | yes (partial) | g | 4217508 | 4219648 | 78.6 | alone, without 159 | transcript ends within 160, but *anti-sense* to *valS* |
| 161 | 28 | GGGAATGGGGAATCGTGAATCGGGAATT | 162 | yes | c | 4330058 | 4334591 | 844.23 |  | near end, sense to both neighboring genes |
| 162 | 28 | GGGAATCGGGAATAGAGAATAGGGAATC | 161 | yes | g | 4330058 | 4334591 | 844.23 |  | see above |
| 163 | 14 | GGGAATCGGGAATC | other | yes | g | 4372557 | 4373356 | 95.09 | other ( c) | transcript stops between 163 and other, long intergenic region between convergent genes, sense with hypothetical gene |
| 164 | 28 | GGTAATCGGGAATCGGGATTGGGGAATC | no | yes | g | 4376023 | 4377671 | 34.03 |  | transcript stops 2nt after 164, sense with *yjcE* (but does not contain whole gene) |
| 165 | 14 | GGGAATGGGGAATC | no | yes | g | 4524352 | 4527168 | 81.46 |  | repeat is in the middle of transcript, sense to neighboring genes |
| 166 | 42 | GGGAATCGGGAATCGGGAACTGGGATTCGGGAATCGGGAATC | no | yes | g | 4532044 | 4533154 | 54.45 |  | transcript ends shortly after 166, *anti-sense* to *acrA* (does not contain whole gene) |
| 167 | 28 | GGGAATCGGGAGTCGGGAATAGGGAATT | 168 | yes | c | 4554331 | 4554890 | 125.95 | 168 | at the beginning of transcript, sense to following gene *folB* |
| 168 | 42 | GGGAGTGGGGAATCGGGAATAGTGAAGAGTGAAGAGTGAAGA | 167 | yes | g | 4554331 | 4554890 | 125.95 | 167 | see above |
| 169 | 28 | GGGAAAGGGGAATTGGGAGTAGGGAATC | 170 | yes | c | 4566669 | 4570761 | 132.66 | 170 | in the middle of transcript, between convergent genes, sense to *cox3* |
| 170 | 21 | GAGAATCGGGAGTCGGGAATC | 169 | yes | g | 4566669 | 4570761 | 132.66 | 169 | see above |
| 171 | 28 | GGGAATGGGGAAATGGGAATGGGGAATC | 172 | yes | g | 4585837 | 4589412 | 39.04 | alone, without 172 | transcript ends directly after 171, long transcript spanning several genes *anti-sense* to all |
| 172 | 28 | GGGTATGGGGAATGGGGAATCGGGAATG | 171 | yes (partial) | g | 4589485 | 4589773 | 69.87 | alone, without 171 | transcript ends within 172, *anti-sense* to *pyrE* (does not contain whole gene) |
| 173 | 28 | GCGAATGGGGAATCGGGAGTTGGGAATC | 174 | yes | g | 4677317 | 4680323 | 95.89 | 174 | repeats are in the middle of transcript, sense to neighboring genes |
| 174 | 28 | GGGGATGGAGAGTCGGGAATCGAGAATG | 173 | yes | c | 4677317 | 4680323 | 95.89 | 173 | see above |
| 175 | 21 | GCGAATCGGGAATCGGGAATG | no | no |  |  |  |  |  | n.a. |
| 176 | 28 | GGGAATGGGGAGTCGGGAATAGGGAATC | 177 | yes | c | 4767453 | 4769937 | 440.1 | 177 | middle of transcript, *anti-sense* to neighboring genes |
| 177 | 21 | GAGAATAGGGAATCGGGAATG | 176 | yes | g | 4767453 | 4769937 | 440.1 | 176 | see above |
| 178 | 28 | GGGAATGGGGAATCGGGAGCAGGGAATC | 179 | yes | c | 4916223 | 4916592 | 66.41 | 179 | transcripts stops within 179, *anti-sense* to *tcsR* (does not contain whole gene) |
| 179 | 35 | GGGAATCGGGAATCGGGAGTGGGGAATCGGGAATC | 178 | yes (partial) | g | 4916223 | 4916592 | 66.41 | 178 | see above |
| 180 | 21 | GGGATTTGGGAATCGGGATTC | no | yes | g | 5158032 | 5159162 | 30.87 | 181 | near the end of transcript, sense with dipeptidase (does not contain whole gene); on same transcript as 181 but repeats face same direction (10 nt between repeats) |
| 181 | 28 | GGGAATGGGGAATCGGGAGTGGGGAATC | no | yes | g | 5158032 | 5159162 | 30.87 | 180 | see above |
| 182 | 14 | GGGGATTGGGAATC | no | no |  |  |  |  |  | n.a. |
| 183 | 21 | GGGAATCGGGAATCGGGAATC | no | yes | g | 5165156 | 5165456 | 10.84 |  | transcript stops after 183, sense to *fecA* (does not cover whole gene) |
